# Two-fold hidden diversity of diminutive horned frogs (Megophryinae: *Jingophrys*) highlights overlooked *in situ* diversification in the Himalayas

**DOI:** 10.1098/rsos.261030

**Published:** 2026-11-11

**Authors:** Sonali Garg, S. D. Biju, Tage Tajo, Radhakrishna Upadhyaya K., James Hanken

**Affiliations:** ^1^ Department of Organismic and Evolutionary Biology and Museum of Comparative Zoology, Harvard University, Cambridge, MA, USA; ^2^ Radcliffe Institute for Advanced Study, Harvard University, Cambridge, MA, USA; ^3^ Systematics Lab, Department of Environmental Studies, University of Delhi, Delhi, India

**Keywords:** amphibians, Asia, behaviour, biogeography, endemism, integrative taxonomy, Megophryidae, molecular evolution, new species, Northeast India

## Abstract

*Jingophrys* is a genus of Asian horned frogs known from six species with disjunct distributions in the Himalayas and Indo-Burma. By integrating evidence from molecular phylogeny, morphology and behaviour, we uncover five new species, recognize two distinct species groups, report two new country records for India and identify additional unnamed lineages. These additions constitute a seven-fold increase in the diversity of Indian *Jingophrys* and a more than two-fold increase for the genus overall. We also describe the first vocal repertoires of all eight Indian members, provide accounts of umbelliform tadpoles for three members and report a previously unknown snail-eating behaviour for the family Megophryidae. Present-day patterns of diversity and endemism may have been shaped by interplay between major rivers that subdivide the Eastern Himalayas from west to east and elevational range divergence at regional scales, which yields up to four congeners within a 10 km radius. The Himalayas are an overlooked and important centre of *in situ* diversification for frogs in the subfamily Megophryinae. Divergence timing, rapid diversification rates and biogeographical patterns across the large megophryine radiation are coincident with active Himalayan–Tibetan orogeny through the Late Oligocene and Miocene and intensification of the South Asian monsoon system starting around 10 Ma, in Late Miocene.

## 1. Introduction

The Asian horned frog family Megophryidae, with a wide distribution across present-day South, Southeast and East Asia, is the largest extant radiation of ‘primitive’ frogs (‘Archaeobatrachia’), which are phylogenetically basal to ‘modern’ frogs (‘Neobatrachia’) [1]. The currently recognized diversity of 375 species, which comprise two subfamilies and 14 genera, dwarfs that of any other non-neobatrachian group [2,3]. New megophryid species continue to be discovered and formally described, with a significant addition of 265 species—a 240% increase—to the 110 species known less than three decades ago at the beginning of the twenty-first century. The most profound increases have occurred in genera such as *Leptobrachella* (96 species) and *Leptobrachium* (26 species) of the subfamily Leptobrachiinae, and *Boulenophrys* (67 species) and *Xenophrys* (23 species) of the subfamily Megophryinae. Yet, several species-depauperate lineages remain poorly understood from a single or handful of poorly known taxa. This complicates efforts to satisfactorily resolve higher-level classification schemes, especially regarding the treatment of major groups at genus or subgenus ranks (e.g. [4–7]). This is particularly true for Megophryinae, in which the previous single-genus (*Megophrys*) classification scheme for a large subfamily became increasingly challenging with accumulating knowledge of complex phylogenetic relationships. Nevertheless, there is growing consensus regarding the major lineages recognized as distinct taxonomic groups, either subgenera or genera (e.g. [4–19]). Recent comprehensive and deep phylogenomic-scale investigations have also shed light on the evolutionary history of megophryine frogs. Using various divergence time estimation approaches, Mahony *et al.* [5] concluded that the crown group ages of the family Megophryidae (57.7–51.5 Ma, in the Late Paleocene and Early Eocene) and the subfamily Megophryinae (28.9–24.5 Ma, during the Late Oligocene) are congruent with both the anuran fossil record and the historical biogeographical events that could explain divergence-dispersal scenarios. They further suggested that Megophryinae originated in Southeast Asia, followed by range expansion through a complex interplay between dispersal and vicariance events that unfolded after the India–Eurasia collision and the primary Himalayan–Tibetan uplift (e.g. [20–22]).

Many groups of large megophryine frogs are widely distributed [5]. However, several small-sized and species-depauperate groups have narrow geographical ranges, possibly owing to insufficient study. Extensive field surveys and basic taxonomic studies in many understudied regions are, therefore, essential for unlocking a more complete understanding of megophryid evolution and biogeography. One such region includes the Himalayas and Indo-Burma, which delineate the western limits of megophryine distribution and contain two genera with the westernmost ranges. *Xenophrys* extends as far west as the Central Himalayas in Nepal—beyond the Eastern Himalayas, which encompasses the Northeastern Region of India (NER India, including Sikkim), Bhutan and Southern China—and is both species-rich (32 species) and widely distributed (eastward to Cambodia) (e.g. [4,12,15,18,23–28]). *Jingophrys*, the second megophryine taxon found in the Himalayas, was recently recognized as a distinct genus [15]. As currently understood, it comprises six species—*Jingophrys pachyproctus* (Huang, 1981), *Jingophrys vegrandis* (Mahony, Teeling and Biju, 2013), *Jingophrys feii* (Yang, Wang and Wang, 2018), *Jingophrys yeae* (Shi, Zhang, Xie, Jiang, Liu, Ding, Luan and Wang, 2020), *Jingophrys zhouii* (Shi, Zhang, Xie, Jiang, Liu, Ding, Luan and Wang, 2020) and *Jingophrys zhaoermii* Huang, Liu, Chen and Yuan, 2026—all restricted to the Eastern Himalayas and adjacent Indo-Burma [15,19].

The fragmented geographic distribution of *Jingophrys*, as currently understood, highlights the need to study intervening regions in the Eastern Himalayas. The six recognized species are known with certainty from just three localities where they are highly restricted: (i) Sessa Village in West Kameng District, Arunachal Pradesh State, India (*J. vegrandis*) [4], (ii) Nabang Town in Yingjiang County and Husa in Dehong Dai and Jingpo Autonomous Prefecture, Yunnan Province, People's Republic of China (*J. feii*, as well as an unnamed population) [6,29], and (iii) multiple adjacent villages and towns in Medog County in Xizang Autonomous Region (Tibet), People's Republic of China (*J. pachyproctus*, *J. yeae*, *J. zhaoermii* and *J. zhoui*) [19,30,31]. A lone exception is a proposed record of *J. pachyproctus* from a fourth locality (Tale Valley Wildlife Sanctuary (WLS) in Lower Subansiri District, Arunachal Pradesh State, India) distant from its type locality (Medog County, People's Republic of China) [32]. All these localities are separated by minimum distances that range from 150 to 680 km, which likely reflect the absence of dedicated surveys in intervening regions. In light of the many recent discoveries of new megophryid populations and species from these regions (e.g. [4,12,19,20,27,31,33–35]), we carried out extensive field surveys across the suspected fuller range of *Jingophrys* in NER India over 5 years (2021–2025). These surveys included both previously explored and many understudied, rarely accessed remote areas. Here, we report our surprising discovery of far more species of *Jingophrys* from India than were previously known, along with systematic, evolutionary, biogeographic and natural history insights that significantly improve our understanding of this unique and long-overlooked group of Asian horned frogs. In addition, we report broader insights into the timing and patterns of diversification and geographic distribution, both historical and present-day, at the wider subfamily-level scale.

## 2. Material and methods

### 2.1. Field surveys and sampling

Field surveys were conducted across the known range of megophryid frogs in the Northeastern Region of India (NER India) that encompasses the Himalaya and Indo-Burma biodiversity hotspots [36]. Sampling was carried out largely during the peak breeding season of frogs in the region (June–September), but also during the pre- and post-monsoon seasons when megophryid frogs were found to be active. During the study period (2021–2025), we located multiple populations of *Jingophrys* only in the Himalayan state of Arunachal Pradesh, and specifically in six districts—West Kameng, Lower Subansiri, East Siang, Lower Dibang Valley, Dibang Valley and Anjaw—where we focused subsequent sampling efforts (supplementary material, tables S1 and S2). Adults were found through opportunistic searches or by locating calling males. Tadpoles were sampled from slow-flowing stream edges or adjacent stagnant pools using scoop nets. Male advertisement calls were recorded in the field at night during active calling hours (18.00–04.00 hours). Ambient air temperatures at the calling sites, both dry and wet bulb, were noted to the nearest 0.1°C. Recorded individuals were captured, weighed and measured for snout-to-vent length (SVL) before being released, except when collected for laboratory studies. Additional microhabitat characteristics and other natural history observations were noted for all studied populations. Geographical coordinates and elevation at surveyed localities were recorded in the World Geodetic System 1984 datum, using Garmin handheld Global Positioning System receivers. Distribution maps were prepared in QGIS v. 2.6.1 (http://www.qgis.org).

Sampled individuals were photographed to capture external morphology and body colouration in life, followed by euthanization in an aqueous solution of tricaine methanesulfonate (MS-222). Prior to preservation, tissue samples were obtained from the thigh muscle of adults or the tail muscle of tadpoles, preserved in absolute ethanol and later stored at −20°C for molecular studies. Adult voucher specimens were fixed in 4% formalin for a few days, washed under running water and preserved in 70% ethanol. Tadpoles were preserved in 10% neutral-buffered formalin. Type specimens are deposited in the amphibian collection of the Zoological Survey of India (ZSI A), Kolkata, India; additional referred specimens (RS) are deposited at the Systematics Lab, University of Delhi (SDBDU), India. Field surveys, including collection of specimens, were conducted with the permission of and following guidelines from the responsible authorities in the State Forest Department of the Government of Arunachal Pradesh, India.

### 2.2. Molecular phylogenetics

Genomic DNA was extracted from 33 field-collected tissue samples representing all previously known and new *Jingophrys* populations from India (supplementary material, table S1) using a Qiagen DNeasy blood and tissue kit (Qiagen, Valencia, CA, USA). A short fragment of the mitochondrial 16S rRNA gene was PCR-amplified for all samples using previously published primers [37]. Based on phylogenetic analyses using the 16S sequences (described below), a subset of samples representing the major recovered lineages were sequenced for additional genetic markers. The following six gene fragments were selected for multigene concatenation-based phylogenetic analyses depending upon data availability from previous studies: four mitochondrial—16S rRNA (16S, approx. 540 bp [37]), 12S rRNA + tRNA-VAL + 16S rRNA (12SV16S, approx. 740 bp [38]) and cytochrome oxidase I (COI, approx. 650 bp [39]); and two nuclear—recombination activating gene 1 (RAG-1, approx. 1100 bp [40,41]) and exon 2 of C-X-C motif chemokine receptor 4 (CXCR-4, approx. 675 bp [42]). The PCR products were sequenced using the BigDye terminator cycle sequencing kit on an ABI 3730 automated DNA sequencer (Applied Biosystems). Newly generated sequences were checked in ChromasPro v1.34 (Technelysium Pty Ltd.) and deposited in the National Centre for Biotechnology Information (NCBI) GenBank under accession numbers PZ484275–PZ484307, PZ485464–PZ485475 and PZ488134–PZ488175.

Additional taxa were sampled by retrieving homologous sequences from NCBI GenBank. These included all previously published sequences of *Jingophrys* and one representative sample of every species of the subfamily Megophriynae recognized at the time of this study [2,3,15] except *Xenophrys damrei* (Mahony, 2011) and *Ophryophryne pachyproctus* Kou, 1985, which lack nucleotide sequence data. Additionally, three species of the subfamily Leptobrachiinae were included to root the subfamily, and a member of the family Pelobatidae was used as the outgroup taxon (supplementary material, table S1). Sequences of each gene were assembled and aligned using the MUSCLE tool [43] in MEGA 7.2 [44]. Alignments for non-coding loci (16S and 12SV16S) were optimized manually; ambiguous sites were excluded from further analysis. Those for the coding loci (CO1, RAG-1 and CXCR-4) were checked by comparison with the translated amino acid sequences. The best-fit models of sequence evolution for each gene partition were determined through a greedy search using the corrected Akaike Information Criterion (AICc) in PartitionFinder 2 [45].

We first prepared a mitochondrial 16S rRNA dataset that included all new and previously available *Jingophrys* sequences, with a total of 77 ingroup samples and *Megophrys montana* as the outgroup taxon (supplementary material, table S1). Tree topology was inferred based on a 511-bp alignment using both the maximum likelihood (ML) and Bayesian inference (BI) approaches. An ML phylogeny was constructed in the IQ-TREE software version 1.6.12 [46] using the best fit GTR+I+G model and obtaining branch support with 10 000 ultrafast bootstrap (UFB) replicates [47]. Nodes with UFB support values ≥ 95% were considered strongly supported [48]. The BI analysis was performed in MrBayes version 3.1.2 [49] using the same model of evolution, with four parallel runs of four Metropolis-Coupled Markov Chain Monte Carlo chains executed for 20 million generations. Default priors were used with tree sampling every 1000 generations. Convergence of the runs was confirmed by average standard deviation of the split frequencies < 0.01 and potential scale reduction factors approaching 1.0. Stationarity of the log-likelihood scores and sufficient effective sample sizes (ESS > 200) for all parameters were also assessed in the software. Bayesian posterior probabilities (BPP) were summarized after discarding the first 25% trees as burn-in [50]; nodes with BPP ≥ 95% were considered strongly supported [51]. Additionally, an ultrametric tree was inferred using the Bayesian evolutionary analysis by sampling trees (BEAST) approach [52] in BEAST version 2.4.4 [53] for use in the general mixed Yule-coalescent (GMYC) species delimitation method discussed below. The BEAST analysis was performed using an uncorrelated relaxed clock model and a Yule tree prior for 100 million generations with tree sampling every 1000 generations. Convergence of the runs, ESS values > 200 for all parameters and burn-in were assessed in Tracer version 1.7 [54]. A maximum clade credibility (MCC) tree was summarized after discarding the initial 10% trees using TreeAnnotator [52].

Molecular species delimitation analyses for the genus *Jingophrys* were conducted based on the mitochondrial 16S rRNA dataset that has complete data for all 77 included taxa. We tested putative species hypotheses using five delimitation methods, including both distance- and tree-based approaches, using default settings: (i) assemble species by automatic partitioning (ASAP) method [55] implemented in iTaxoTools [56], (ii) automatic barcode gap discovery (ABGD) method [57] performed on the SpartExplorer web platform (https://spartexplorer.mnhn.fr) [58], (iii) Poisson tree processes (PTP) method [59] using the IQ-TREE generated ML tree, (iv) Bayesian Poisson tree processes (bPTP) method [59] using the MrBayes-generated Bayesian tree, with both PTP and bPTP implemented on the webserver (http://species.h-its.org), and (v) general mixed Yule-coalescent (GMYC) method [60] implemented in the software R [61] using the ‘splits’ package [62] and applying the single-rate model to the BEAST-generated ultrametric gene tree. Additionally, intra- and interspecific uncorrected pairwise distances were independently computed using PAUP* 4.0b10 [63].

To further assess genetic structure and potential haplotype sharing in the nuclear markers, median-joining networks were independently constructed using available *Jingophrys* samples for the CXCR-4 (582 bp, 20 samples) and RAG-1 (863 bp, 46 samples) gene alignments after excluding sites with gaps and missing data. Haplotypes were estimated for each set of gene sequences using the PHASE algorithm [64] as implemented through ConvPhase in the software Hapsolutely [65]. The phased data were then used to construct haplotype networks using the Fitchi algorithm [66] within Hapsolutely.

Based on the preliminary species hypotheses from our mitochondrial 16S analyses, a concatenated multi-locus data matrix comprising alignments for six gene fragments, four mitochondrial (525 bp of 16S, 748 bp of 12SV16S and 626 bp of COI) and two nuclear (677 bp of CXCR-4 and 914 bp of RAG-1), was used to evaluate phylogenetic support and relationships in the framework of a wider phylogeny of the family Megophryidae with 231 taxa (supplementary material, table S1). Tree topology was inferred in both ML and BI frameworks based on the final 3490-bp character matrix partitioned by genes and using the following best-fit models resulting from PartitionFinder 2: GTR+I+G for 16S, 12SV16S, COI and RAG-1; TIM+I+G for CXCR-4. The mixed-model partitioned ML and BI analyses were executed in IQ-TREE version 1.6.12 [46] and MrBayes version 3.1.2 [49], respectively, setting all parameters (except the models) identical to those described above for the 16S analyses.

Additionally, a time-calibrated phylogeny based on the multi-locus dataset was constructed using BEAST version 2.4.4 [53]. We estimated divergence times among lineages in the subfamily Megophryinae based on two literature-based secondary calibration points taken from results based on the most optimum RelTime analyses of mt+nuDNA dataset [5, fig. 1 and tab. S6]: (i) the crown age of the most recent common ancestor (MRCA) of Megophryinae (peak age, 26.4 million years ago (Ma)), and (ii) the MRCA of the split between clades containing genera (*Boulenophrys* + *Ophryophryne*) and ((*Xenophrys* + *Grillitschia*) + *Jingophrys*) (corresponding to the diversification of clades ‘7–(5–6)’, peak age 20.3 Ma). Each gene partition was independently assigned an HKY+G substitution model to avoid over-parameterization and an uncorrelated relaxed molecular clock model with a log-normal distribution. A birth–death model was used for the tree prior, as it takes into account both lineage-birth and extinction-rate parameters that may be expected for phylogenetic diversification estimations at a subfamily level in this radiation of an ancient group of frogs. The MCMC chains were executed for 100 million generations with tree sampling frequency set at every 1000 iterations. Convergence and sufficient ESS values > 200 for all parameters were ensured using Tracer version 1.7 [54]. An MCC tree was summarized after discarding the initial 10% trees as burn-in using TreeAnnotator [52].

**Figure 1.**
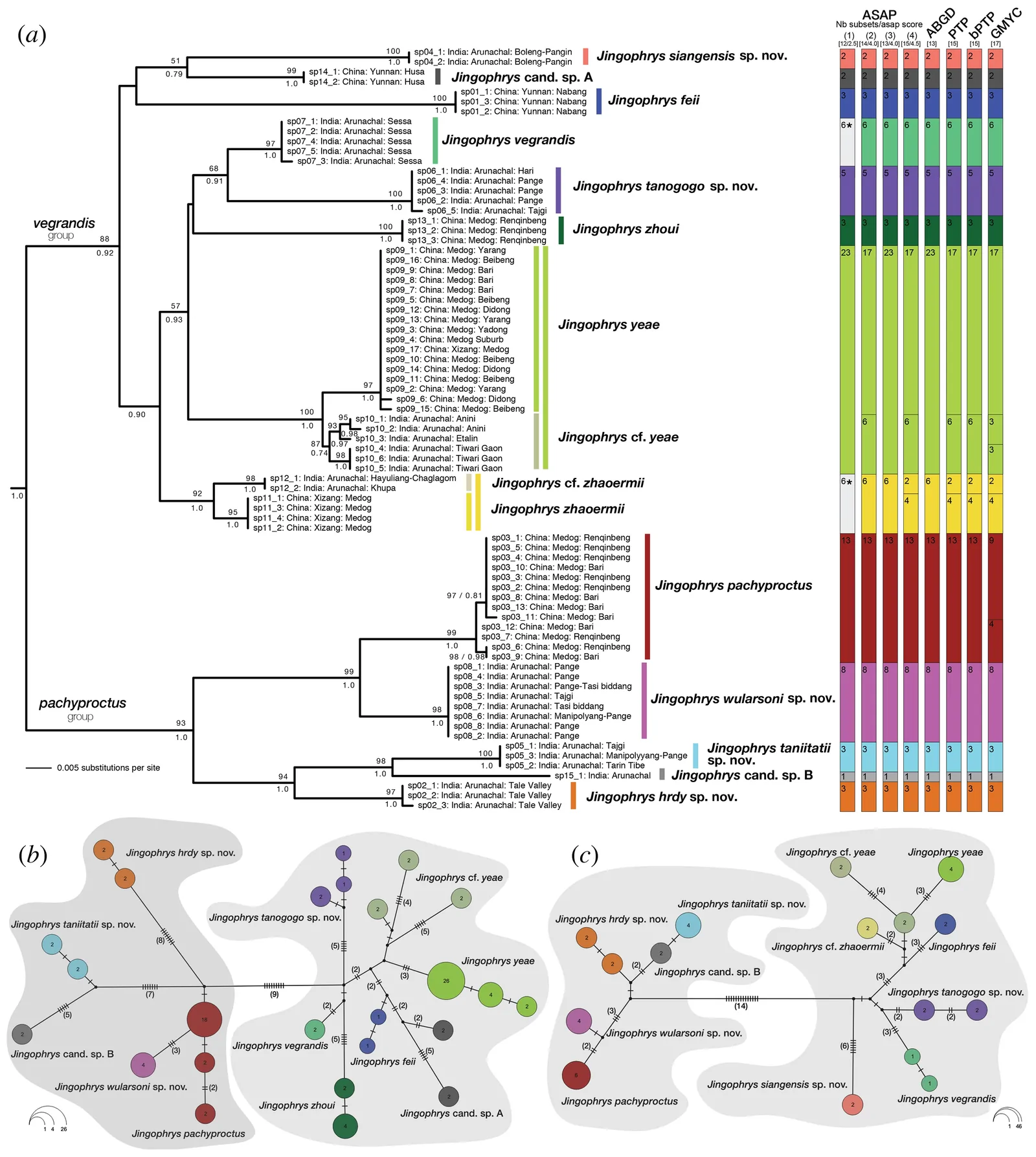
Molecular species delimitation and relationships. (*a*) Maximum likelihood (ML) phylogram based on the mitochondrial 16S rRNA gene shows delimited species partitions and relationships among all 77 samples of *Jingophrys*. Values above and below branches indicate Ultrafast bootstrap support (UFB > 50%) and Bayesian posterior probabilities (BPP > 0.50), respectively. Voucher details for taxon labels at the terminal nodes are presented in table S1 in the supplementary material. Colours of thin vertical bars and corresponding taxon names to the right of tip labels indicate the confirmed species hypothesis based on integrative approaches. Larger coloured vertical rectangles depict the results of species delimitation analyses (number of recovered putative species partitions in parentheses): four lowest-scoring ASAP hypotheses (12–15 species), ABGD hypothesis (13 species), ML tree-based PTP hypothesis (15 species), Bayesian tree-based bPTP hypothesis (15 species) and ML-based GMYC hypothesis (17 species). Light-grey segments with asterisks denote two phylogenetically unsupported partitions. (*b*, *c*) Haplotype networks for two nuclear markers show genealogical relationships among *Jingophrys* populations: (*b*) RAG-1 network based on an 863-bp fragment for 46 populations and (*c*) CXCR-4 network based on a 582-bp fragment for 20 populations. Sequences were phased prior to analysis; hence, each sample is represented by two sequences. Circles represent haplotypes; sizes are proportional to allele frequencies and colours indicate species and other divergent populations. Small black circles represent median vectors. Number of mutational steps is indicated on branches.

We estimated diversification rates (DRs) across Megophryinae and tested for rate variation among lineages. The DR statistic [67] was calculated as an estimator of lineage-specific speciation rates over time. Only a single exemplar was retained for each species-level lineage, and outgroups were excluded from the analyses. Rate distribution was visualized on the MCC tree using the ‘phytools’ R package [68]. Similarly, lineage-through-time (LTT) plots were generated on the MCC tree to visualize lineage accumulation over time for Megophryinae as well as on subsampled trees for three genera (*Jingophrys*, *Xenophrys* and *Boulenophrys*), using the ‘phytools’ [68] and ‘ape’ [69] packages in R [61]. Multiple lineage-through-time (MLTT) plots were additionally generated for 1000 trees sampled from the posterior distribution to account for phylogenetic uncertainty. The observed lineage accumulation rates were quantified and compared for deviation against the expected pure-birth null model based on the Pybus and Harvey's gamma (*γ*) statistics [70].

The biogeographic history of Megophryinae was inferred using the BioGeoBEARS version 0.2.1 [71] package in R [61]. Ancestral range reconstructions were made using the time-calibrated MCC tree after retaining a single exemplar for each species-level lineage and excluding outgroups. Seven simplified geographical areas were identified based on the present-day distributions of megophryine frogs across Asia: (A) Himalayas, (B) Indo-Burma, (C) Southwest China, (D) Southeast China, (E) Malay Peninsula, (F) West Sunda Islands and (G) East Sunda Islands. Since no species is currently known to occupy more than three of these areas, the maximum number of areas allowed in the analyses was set at three. Six different models of historical biogeography were tested in an ML framework: dispersal–extinction–cladogenesis (DEC), dispersal-vicariance analysis LIKE (DIVALIKE), BayArea LIKE (BAYAREALIKE), and a variation of each model with an additional founder-event speciation (jump dispersal, J) parameter included (DEC+J, DIVALIKE+J and BAYAREALIKE+J) [72–75]. The best-supported model was determined using AICc scores and relative model probabilities based on AICc weights.

### 2.3. External morphology

We studied the external morphology of all newly sampled *Jingophrys* specimens and compared them with original species descriptions and type specimens (when available). Identities were confirmed based on comparison of DNA sequences with those from published types and, in some cases, with new topotypic material from our sampling. Detailed comparisons were made among populations suspected of representing new species and all currently recognized congeners. All the *Jingophrys* members were further assigned to two distinct species groups newly recognized in this study (see §3) and compared to identify group-level diagnostic morphological characters.

Sex and maturity of specimens were determined by the presence of external secondary sexual characters (e.g. vocal sacs in males) or direct examination of gonads through a small ventral incision. Only adult specimens were used in morphometric studies. The following 26 measurements were taken to the nearest 0.1 mm by using digital slide-calipers or a stereomicroscope equipped with an ocular micrometer: snout-vent length (SVL, from snout tip to cloacal opening), head length (HL, from rear of mandible to tip of snout), head width (HW, at the angle of the jaws), snout length (SL, from tip of snout to anterior orbital border), eye length (EL, horizontal distance between bony orbital borders), greatest tympanum diameter (TYD), distance from tympanum to back of eye (TYE), distance from tympanum to distal end of supratympanic fold (TYF), distance from front of eye to nostril (EN), distance from nostril to tip of snout (NS), inter upper eyelid width (IUE, shortest distance between upper eyelids), maximum upper eyelid width (UEW), internarial distance (IN), snout projection (SP, from anterior end of lower lip to tip of snout), forearm length (FAL, from flexed elbow to base of outer palmar tubercle), hand length (HAL, from base of outer palmar tubercle to tip of third finger), lengths of fingers I–IV (FIL–FIVL), thigh length (TL, from vent to knee), shank length (SHL, from knee to heel), foot length (FOL, from base of inner metatarsal tubercle to tip of fourth toe), total foot length (TFOL, from heel to tip of fourth toe), maximum width of toe IV (TIVW) and maximum width of toe IV fringe (TIVF). Measurements and photographs were taken from the right side except when otherwise stated, or a character was damaged, in which case they were taken from the left side. Linear measurements are expressed in millimetres, unless noted otherwise. Morphological terminology and definitions largely follow earlier studies [4,76]. Snout-shape terminology follows Heyer *et al.* [77]. Terminology for digit tip morphology follows Biju *et al.* [78]. Illustrations of dermal microstructures are based on the examination of the skin surface under a stereomicroscope.

Multivariate analyses were performed to assess the degree of morphological differentiation and variation within and among species of the two species groups. Morphometric data for 22 characters from adult male specimens were size-corrected (ratio of each head character to HL; ratio of HL and remaining measurements to SVL) and log-transformed to reduce the impact of allometry. Principal component analyses (PCAs) were executed in the software R [61], and factor loadings of the first two principal components (PCs) were visualized to assess spatial distribution on group-wise scatterplots using the ‘ggplot2’ [79] and ‘ggfortify’ [80] packages.

### 2.4. Micro-computed tomography

Micro-computed tomography (µCT) scans were prepared from adult specimens of two *Jingophrys* species reported from India as part of a separate unpublished study. This led to visualization of calcified stomach contents that are reported in the present study. Specimens were scanned using a SkyScan 1273 scanner (Bruker MicroCT, Belgium) with voltage and current set between 70 and 80 kV and 114 and 187 µA, respectively, exposure times between 75 and 470 ms and voxel resolutions between 9.8 and 13 µm. Raw X-ray data were reconstructed using NRecon version 1.6.6.0 (Bruker MicroCT). Image stacks were processed in VG StudioMax version 2025.1 (Volume Graphics, Germany) to visualize and isolate the stomach contents using various rendering and segmentation tools. No additional processing of data was carried out for the purpose of this study.

### 2.5. Vocalization

Male advertisement calls were recorded from all eight *Jingophrys* species reported from India in the present study. Recordings were made on a hand-held solid-state digital audio recorder (Marantz PMD620, Tascam DR-40X or Zoom H6) connected to a unidirectional microphone (Sennheiser ME 66 or MKH 416) equipped with a foam windscreen. Individual calls were sampled at a 44.1 kHz rate with 16 bit resolution and monitored in real time using headphones. The recorder's gain settings were adjusted prior to each recording to avoid clipping the amplitude envelopes and then maintained to ensure a constant signal-to-noise ratio throughout the recording. The microphone was positioned 30–80 cm from each calling individual. A minimum of 20 calls were recorded per individual, and recordings with at least that many continuous notes were used for further analyses. A total of 50 calls were analysed per species.

Acoustic analyses were performed in Raven Pro version 1.6.5 [81], following Charif *et al.* [82]. Since all species in the present study produced a single type of call with pulsatile temporal structure, we measured the same five temporal properties (call duration, call rise time, call fall time, number of pulses per call and pulse rate) for each call. Oscillograms depicting the amplitude-versus-time waveform were generated to graphically represent call segments across relevant time frames (10, 1 or 0.3 s). Additionally, a spectral property (overall dominant frequency) was measured using the spectrogram function after averaging the spectrum over the entire call (1024-point fast Fourier transform, Hann window, 50% overlap, 43.1 Hz resolution). Spectrograms were prepared corresponding to the 1- or 10-s time-frame oscillograms. Representative cuts of the call recordings analysed are deposited in the Zenodo repository. All measurements were taken following the definitions, terminologies and methods of Bee *et al.* [83] and Garg *et al.* [84]. Species-specific vocal repertoires and interspecific call comparisons among species and species groups, including limited previously published call data [29,31], are presented to describe the broad call characteristics of members of this poorly studied group of frogs. At the same time, reported values of acoustic properties do not account for possible sources of variation, such as differences in ambient temperature and social context, that may cause variation both among and within individuals of a species [85].

Multivariate analyses were further performed on the acoustic data to assess the degree of differentiation among members of the two recognized species groups. PCAs were performed using the raw descriptive statistical data for all six measured temporal and spectral variables. Factor loadings of the first two PCs were visualized to assess spatial distribution on group-wise scatterplots using the ‘ggplot2’ [79] and ‘ggfortify’ [80] packages in R [61].

### 2.6. Larval morphology

External morphology of newly sampled tadpoles of three *Jingophrys* species reported from India in the present work was examined using a Nikon SMZ 1500 stereomicroscope. Tadpoles were staged following Gosner [86], and specimens ranging from Gosner stages 26 to 35 were used for detailed morphological descriptions and morphometric studies. The following 18 characters were measured to the nearest 0.1 mm using digital slide-calipers: total length (TL), body length (BL), body height (BH), maximum body width (BW), snout to upper tail fin (SU), snout to spiracle (SS), tail length (TAL), maximum tail height (MTH), upper fin height (UFH), lower fin height (LFH), tail muscle height (TMH), tail muscle width (TMW), snout to nares (SN), nares to eye (NE), maximum eye diameter (ED), interorbital distance (IOD), internarial distance (IND) and oral disc width (ODW). Larval measurements and descriptions use the definitions, terminologies and methods of Gopika *et al.* [87] following Altig and McDiarmid [88] and Altig [89]. Mouthparts, including oral disc morphology and margins of papillae, were visualized using blue ink and illustrated with the aid of photographs taken using a digital Q-imaging camera fitted to a stereomicroscope.

### 2.7. Nomenclatural acts

The electronic version of this article in Portable Document Format represents a published work according to the International Commission on Zoological Nomenclature (ICZN); hence, the new names contained in the electronic version are effectively published under that Code from the electronic edition alone. This published work and the nomenclatural acts it contains have been registered in ZooBank, the online registration system for the ICZN. The ZooBank LSIDs (Life Science Identifiers) can be resolved, and the associated information viewed, using any standard web browser by appending the LSID to the prefix http://zoobank.org/. The LSID for this publication is: urn:lsid:zoobank.org:pub:9485DB13-FC2C-4ECE-90AB-387435ED010B. The online version of this work is archived and available from major indexing services and digital repositories such as PubMed, PubMed Central, Web of Science and Scopus, Directory of Open Access Journals, Astrophysics Data System as well as many other indexes.

### 2.8. Abbreviations

Institutional acronyms and other frequently used abbreviations are as follows: Systematics Lab, University of Delhi (SDBDU), India; Zoological Survey of India, Kolkata (ZSI A, formerly ZSIC), India; Zoological Survey of India–North Eastern Regional Centre (ZSI–NERC), India; Chengdu Institute of Biology (CIB), People's Republic of China; Natural History Museum (NHM), formerly British Museum (Natural History) (BMNH), London, UK; Museum of Comparative Zoology (MCZ), Harvard University, USA; holotype (HT); paratype (PT); topotype (TT); referred specimen (RS); Wildlife Sanctuary (WLS); Northeastern Region of India (NER India).

## 3. Results

### 3.1. Molecular species delimitation

Molecular species delimitation analyses combined with phylogenetic inferences based on the 16S genetic marker recover a large number of distinct mitochondrial lineages that far exceed the number of nominal *Jingophrys* species and potentially available scientific names from the known range in the Himalayan and Indo-Burma regions of India and the People's Republic of China (figure 1*a*; supplementary material, tables S1 and S2;). Previously identified as the *Megophrys* (*Xenophrys*) *vegrandis* group with only two described species [4,5,25,30], *Jingophrys* as currently recognized [15] contains six nominal species, four of which were described only recently [19,29,31]. This total represents less than half of the potential diversity suggested by our tests of several species-delimitation hypotheses.

The ASAP approach recovers between 12 and 25 species partitions with scores between 2.5 and 10.5; only those partitions with the four lowest scores were considered further (figure 1*a*). The lowest-scored partitioning scheme (ASAP 2.5, with 12 species subsets) merges two clades containing previously recognized species (*J. vegrandis* and (*J. zhaoermii* + *J*. cf. *zhaoermii*)) that are not recovered as a single monophyletic lineage in any other analysis based on either 16S or the multigene datasets, or in published literature [19]. The next two partitioning schemes, each with a score of 4.0, suggest 14 and 13 species, respectively, that are also phylogenetically recovered as well-supported clusters. The fourth hypothesis (ASAP 4.5, 15 species) is congruent with findings of two additional delimitation tests, PTP and bPTP (see below). The latter three results (ASAP 4.5, PTP and bPTP) were, therefore, considered the most parsimonious preliminary species hypothesis and adopted as the best partitioning schemes for further evaluation. Another distance-based approach, ABGD, suggests 13 species partitions that best align with our final taxonomic decisions following evaluation of multiple lines of evidence, including phylogeny, morphology and behaviour (figure 1*a*).

Three additional delimitation analyses employing tree-based approaches recover the same number of or slightly more species than the four lowest-scoring ASAP partitions. Both the ML tree-based single-rate PTP analysis and the Bayesian tree-based bPTP analysis yield identical 15 species partitions. Overall, this was the best-supported species hypothesis phylogenetically, based on the 16S as well as multigene datasets, although we ultimately adopted a more conservative approach in recognizing only 13 species. The coalescence-based GMYC approach, with 17 species, delimits the highest number of partitions. It not only differentiates Indian from Chinese populations of both *J. yeae* and *J. zhaoermii*, hence recognizing as candidate species populations we treat as *J*. cf. *yeae* and *J.* cf. *zhaoermii*, but it also partitions Medog populations of *J. pachyproctus* into two distinct, albeit shallowly divergent clusters (figure 1*a*).

Based on the molecular species delimitation results and corresponding phylogenetic support in the ML and BI trees (figure 1*a*), we assign names to 15 reliable preliminary species partitions, viz., candidate species. These are hereafter referred to by their final names, as determined through the following integrative analyses that incorporate multiple lines of additional evidence. The 15 taxa include six previously described *Jingophrys* species that we validated based on sequence data derived from the type series or morphologically verified topotype and other populations—*J. feii* (Yang, Wang, and Wang, 2018), *J. pachyproctus* (Huang, 1981), *J. vegrandis* (Mahony, Teeling and Biju, 2013), *J. yeae* (Shi, Zhang, Xie, Jiang, Liu, Ding, Luan and Wang, 2020), *J. zhaoermii* Huang, Liu, Chen and Yuan, 2026 and *J. zhouii* (Shi, Zhang, Xie, Jiang, Liu, Ding, Luan and Wang, 2020); five new species formally described in the present study—*Jingophrys hrdy* sp. nov., *Jingophrys siangensis* sp. nov., *Jingophrys taniitatii* sp. nov., *Jingophrys tanogogo* sp. nov. and *Jingophrys wularsoni* sp. nov.; two distinct but currently unnamed species—*Jingophrys* candidate sp. A and *Jingophrys* candidate sp. B; and two additional genetically divergent populations of recognized species—*Jingophrys* cf. *yeae* and *Jingophrys* cf. *zhaoermii* (figure 1*a*).

Although 15 preliminary species partitions are best supported in our various molecular species delimitation analyses, our subsequent taxonomic decisions incorporate additional lines of evidence that include phylogeny, external morphology and acoustic characters. When combined, these data reliably support recognition of 13 species-level lineages, 11 of which are assigned formal taxonomic names and two are retained as candidate species pending additional taxonomic study. The remaining two lineages, *Jingophrys* cf. *yeae* and *Jingophrys* cf. *zhaoermii*, are treated as divergent populations of previously named species based on shallow genetic divergence, inconsistent phylogenetic affinities and at best subtle differences in morphology and acoustic characters that may represent intraspecific variation. In the absence of additional evidence that suggests otherwise, we refrain from treating them as distinct species at this time. Additional explanation of our taxonomic decisions and comparisons between these lineages and their allied species are provided in the respective species accounts.

### 3.2. Genetic differentiation and haplotype networks

The inferred candidate species of *Jingophrys* exhibit a high degree of genetic differentiation in the mitochondrial 16S rRNA gene (figure 1*a*; supplementary material, table S3). For the 15-species hypothesis, the average uncorrected pairwise genetic distance ranges from 1.6 to 11.9% between candidate species and from 0 to 0.8% within candidate species. For the 13-species hypothesis that is favoured by our integrative assessments, inter- and intraspecific divergence falls in the range of 3.2–11.9% and 0–2%, respectively. The latter values also align with species-level divergence observed across frogs more broadly, including megophryids (e.g. [12,31,34,90–92]). Not surprisingly, interspecific divergence is high between members of the *J. pachyproctus* and *J. vegrandis* species groups; values range from 6.9 to 11.9%. Within species groups, members of the *J. pachyproctus* and *J. vegrandis* groups are similarly divergent, with interspecific ranges of 3.4–8.7% and 3.2–8.9%, respectively. Intraspecific divergence among populations, however, is higher in the latter group, reaching 2% in *J. yeae* sensu lato (versus 0.4% in *J. yeae* sensu stricto, 1% in *J.* cf. *yeae* and 1.6–2.0% between *J. yeae* sensu stricto and *J.* cf. *yeae*) and 1.6% in *J. zhaoermii* sensu lato (versus 0% in both *J. zhaoermii* sensu stricto and *J.* cf. *zhaoermii*, and 1.6% between *J. zhaoermii* sensu stricto and *J.* cf. *zhaoermii*). Remaining species exhibit low intraspecific divergence: < 0.4% in the *J. vegrandis* group and < 0.6% in the *J. pachyproctus* group. Species pairs with the lowest divergence include *J. vegrandis* and *J. zhaoermii* sensu stricto (3.2–3.4%), which otherwise are confirmed to be distinct from each other and treated as valid species based on published evidence. The 13-species partition also shows no overlap between the lowest interspecific distance and the highest intraspecific distance. For group-wise pairwise comparisons and discussions of species within species groups, see table S3 in the supplementary material and the following systematic accounts.

The above mitochondrial 16S rRNA data also corroborate results of independent analyses of the two nuclear markers. Median-joining haplotype networks for both RAG-1 and CXCR-4 recover all 15 inferred species partitions as distinct clusters with no shared nuclear haplotypes (figure 1*b*). Putative candidate species are clearly separated into group-level clusters that correspond to the two species groups recognized here, with a minimum of nine and 14 mutation steps for RAG-1 and CXCR-4, respectively. In the RAG-1 network, each candidate species is separated from the closest haplotype of another candidate species by at least three mutation steps. Similarly, all but one formally named taxa, including candidate species, are separated by at least three mutation steps in the CXCR-4 network. The lone exception is *Jingophrys* cand. sp. B, which is separated by a single mutation step from its closest relative, *Jingophrys taniitatii* sp. nov.; these taxa are separated by at least six mutation steps in the RAG-1 network. The larger number of hypothetical nodes among some taxa may indicate sampling gaps or point to yet undiscovered diversity in underexplored regions.

### 3.3. Phylogenetic relationships

Phylogenetic inferences based on a multigene mitochondrial + nuclear (mt + nu) dataset recover all the inferred candidate species of *Jingophrys* as distinct and well-supported lineages in both the ML and BI analyses (figure 2*a*). Individual species-level clades are recovered with strong support (BPP 1.0, UFB 99–100), with two exceptions: there is weak support for the *J.* cf. *yeae* clade in one analysis (BPP 0.96, unsupported in ML), and *J.* cf. *zhaoermii* is a distinct subclade in BI (BPP 1.0) but nests within *J.* cf. *yeae* with strong support in ML (UFB 100). The single available sample of *J*. cand. sp. B is well supported as the sister taxon of *Jingophrys taniitatii* sp. nov. (BPP 1.0, UFB 100). All inferred lineages are consistently assigned to their respective monophyletic sister species groups (BPP 1.0, UFB 100), each of which receives strong support (BPP 1.0, UFB 100): the *J. pachyproctus* species group, with five members—*Jingophrys hrdy* sp. nov., *J. pachyproctus*, *Jingophrys taniitatii* sp. nov., *Jingophrys wularsoni* sp. nov. and *J*. cand. sp. B; and the *J. vegrandis* species group, with ten members—*J. feii*, *Jingophrys siangensis* sp. nov., *Jingophrys tanogogo* sp. nov., *J. vegrandis*, *J. yeae*, *J.* cf. *yeae*, *J. zhaoermii*, *J.* cf. *zhaoermii*, *J. zhouii* and *J*. cand. sp. A.

**Figure 2.**
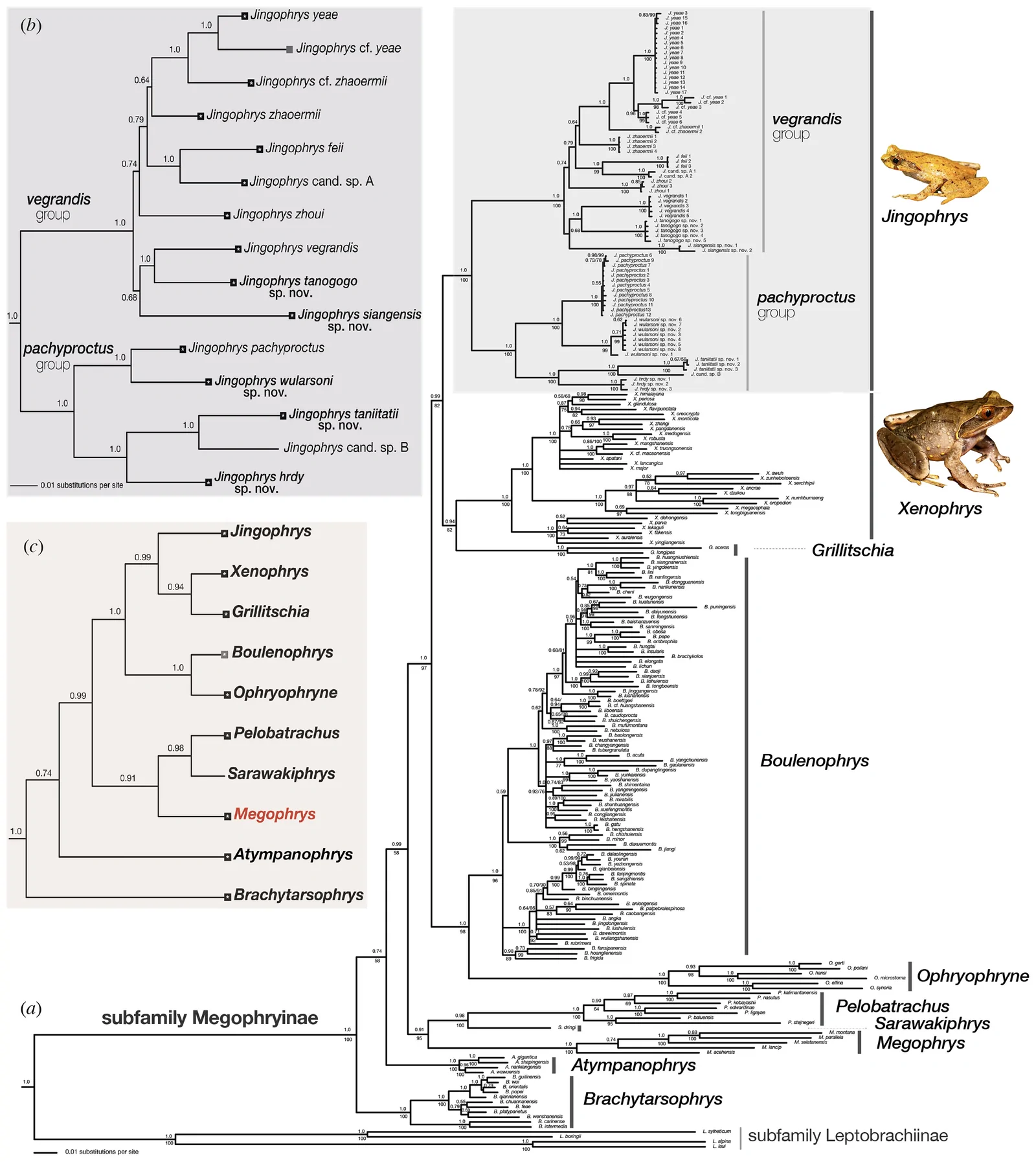
Phylogeny of the family Megophryidae based on the concatenated multi-locus dataset of mitochondrial and nuclear DNA. (*a*) Maximum likelihood (ML) phylogram depicting genus- and species-level relationships. Phylogenetic position of *Jingophrys*, as well as relationships among species recovered in two major groups—*J. pachyproctus* species group and *J. vegrandis* species group—are based on multiple samples. All other recognized species in the family are represented by a single sample each. Voucher details are presented in table S1 in the supplementary material. An outgroup pelobatid taxon included in the analyses is not shown. Values above and below branches indicate Bayesian posterior probabilities (BPP > 0.50) and Ultrafast bootstrap support (UFB > 50%), respectively. All currently recognized genera are indicated by dark grey vertical bars; frog images indicate the two genera currently known to occur in India. The shaded rectangle highlights *Jingophrys*, with the two newly recognized species groups indicated by light grey bars; (*b*) phylogram depicting relationships among the 15 distinct lineages recovered in the genus *Jingophrys*, including 11 recognized species, two divergent populations of named species and two unnamed candidate species; (*c*) cladogram depicting relationships recovered among genera of the subfamily Megophryinae. The type genus is indicated in red.

Interspecific relationships within species groups receive varying degrees of support. In the *J. pachyproctus* group, all relationships are congruent in both the ML and BI analyses of the mt + nu dataset and are identical to similar analyses of the 16S dataset alone (figures 1*a* and 2*a*). These include the sister relationships of *J. pachyproctus* and *Jingophrys wularsoni* sp. nov. (mt + nu: BPP 1.0, UFB 100; 16S: BPP 1.0, UFB 99), *Jingophrys taniitatii* sp. nov. and *J*. cand. sp. B (mt + nu: BPP 1.0, UFB 100; 16S: BPP 1.0, UFB 99) and *Jingophrys hrdy* sp. nov. and the clade containing *Jingophrys taniitatii* sp. nov. + *J*. cand. sp. B (mt + nu: BPP 1.0, UFB 100; 16S: BPP 1.0, UFB 94). In the *J. vegrandis* group, however, relationships are considerably more variable; those that are well supported in one analysis are not always consistent with relationships recovered in the other. For example, a sister relationship of *J. feii* and *J*. cand. sp. A is well supported in the mt + nu analyses (BPP 1.0, UFB 99), but *J*. cand. sp. A is recovered as sister to *Jingophrys siangensis* sp. nov. in the 16S analyses, albeit with weak support (BPP 0.79, UFB 51), and the latter two taxa together are the sister group to *J. feii*. Similarly, mt + nu analyses yield weak support (BPP 0.68, not supported in ML) for a sister relationship between *Jingophrys siangensis* sp. nov. and the clade containing *Jingophrys tanogogo* sp. nov. and *J. vegrandis* (BPP 1.0, UFB, not supported in ML); the latter sister group relationship also receives only moderate support in the 16S analyses (BPP 0.91, UFB 68). Overall, relationships within the *J. vegrandis* group receive stronger support in the BI analyses (figure 2*b*). However, placements of *J. zhaoermii* and *J. zhouii* remain largely uncertain in all analyses. A basal position of the two species relative to multi-species clusters is inferred with weak to moderate support (BPP 0.64–0.79, UFB 54), yet there is strong support for *J. zhaoermii* as basal to other members of the *J. vegrandis* group (mt + nu: UFB 100). At the same time, *J.* cf. *zhaoermii* does not nest with *J. zhaoermii* in the mt + nu analyses, which instead place it basal to the clade containing *J. yeae* + *J.* cf. *yeae* (BPP 1.0, not supported in ML), although it nests with *J. zhaoermii* in the 16S analyses (BPP 1.0, UFB 92). Similarly, nesting of *J.* cf. *yeae* with *J. yeae* is weakly supported in the mt + nu ML analyses (UFB 67), but with the *J.* cf. *zhaoermii* subclade included. *Jingophrys yeae* + *J.* cf. *yeae* are consistently recovered as a monophyletic group with strong support in other analyses (mt + nu: BPP 1.0; 16S: BPP 1.0, UFB 100).

Phylogenetic relationships at the subfamily level are largely congruent between ML and BI analyses (figure 2*a*) and are in broad agreement with established genus-level relationships in Megophryinae (e.g. [5,15]). *Jingophrys* is well supported as a monophyletic genus (BPP 1.0, UFB 100), allowing us to confidently assign our new samples to it (figure 2*b*), which, as discussed below, additionally possess diagnostic morphological characters for the genus [15]. Its sister group relationship with the clade containing *Xenophrys* + *Grillitschia* receives strong to moderate support (BPP 0.99, UFB 82), as does the sister group relationship between the latter two genera (BPP 0.94, UFB 82). The (*Jingophrys* + (*Xenophrys* + *Grillitschia*)) clade is also well supported as the sister group to *Boulenophrys* + *Ophryophryne* (BPP 1.0, UFB 97), as is the sister group relationship between the latter two genera (BPP 1.0, UFB 96). Remaining megophryine genera are recovered as basal lineages to the five aforementioned genera, but with variable support. The split between the clade containing (*Jingophrys* + (*Xenophrys* + *Grillitschia*)) + (*Boulenophrys* + *Ophryophryne*) and the clade uniting (*Pelobatrachus* + *Sarawakiphrys*) + *Megophrys* receives strong BI support but weak ML support (BPP 0.99, UFB 58). The sister group relationship of *Pelobatrachus* and *Sarawakiphrys* is well supported (BPP 0.98, UFB 100), as is their sister relationship with *Megophrys*, the type genus of the subfamily (BPP 0.91, UFB 95; figure 2*c*). The position of *Atympanophrys* as basal to all the above taxa is weakly supported (BPP 0.74, UFB 58), whereas the position of *Brachytarsophrys* as most basal within the subfamily is well supported (BPP 1.0, UFB 100). Previous studies yield similar sequential relationships of *Atympanophrys* and *Brachytarsophrys*, but also an *Atympanophrys* + *Brachytarsophrys* sister relationship (e.g. [5,93]); the latter is recovered with significant support as an alternate topology in our time-calibrated Bayesian analyses (BPP 0.94; figure 3). Monophyly of the subfamily Megophryinae receives strong support based on complete sampling of species-level representatives and additional samples of the sister group, subfamily Leptobrachiinae (BPP 1.0, UFB 100). Overall support for and agreement regarding relationships at higher taxonomic levels in the ML and BI analyses provide additional confidence in our phylogenetic inferences (figure 2*c*).

**Figure 3.**
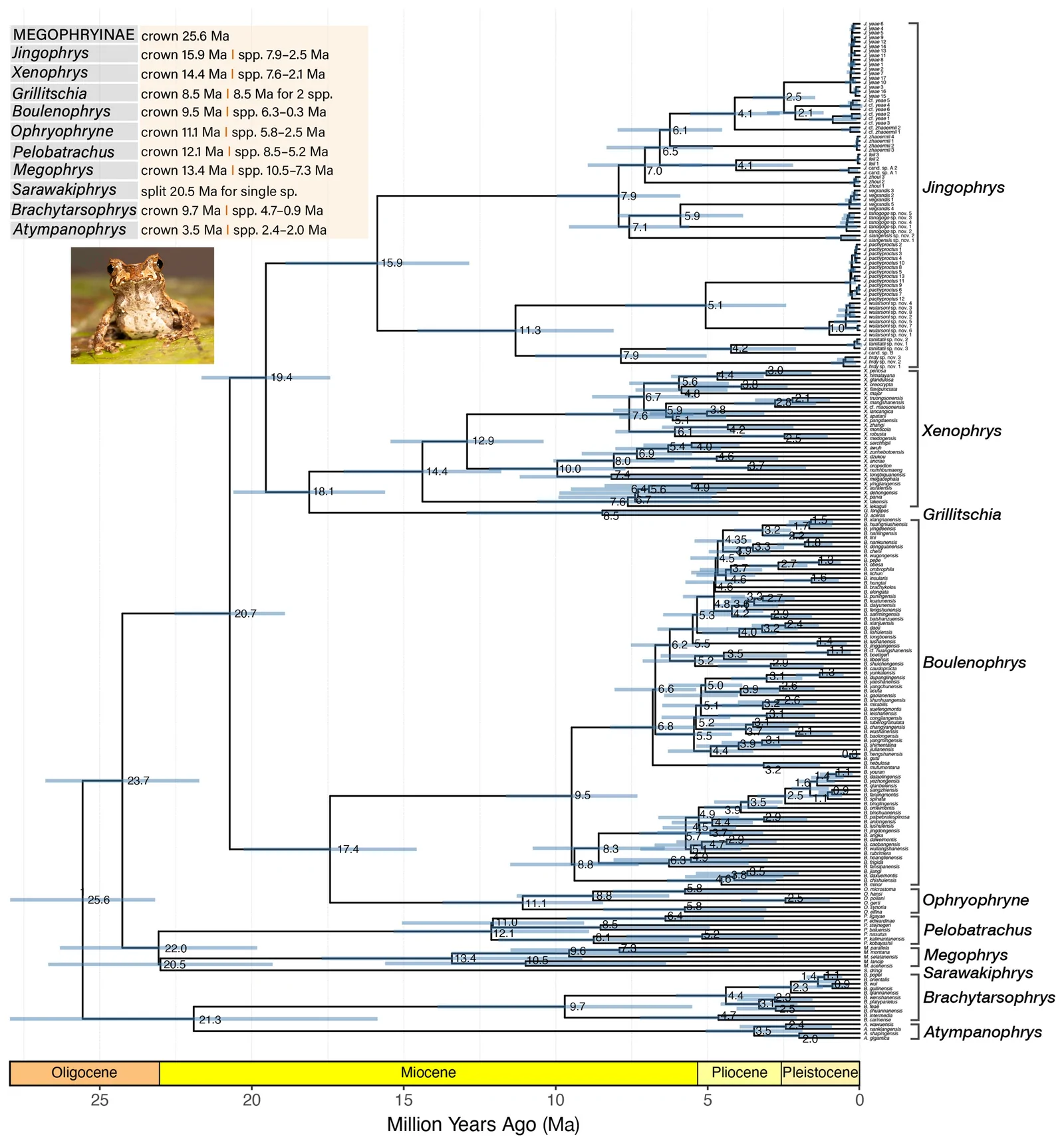
Time-calibrated maximum credibility cladogram (MCC) of the subfamily Megophryinae based on the concatenated multi-locus dataset of mitochondrial and nuclear DNA. Initial diversification began in the Late Oligocene. Major genus-level clades were established after the Oligocene–Miocene transition, coincident with periods of rapid restructuring of the continental Sundaland plate driven by collision of Indian and Eurasian plates and accelerated Himalayan–Tibetan orogeny. Major species group-level clades were established during the Mid-to-Late Miocene, followed by rapid within-clade diversification of *Jingophrys* and other closely related groups, including *Boulenophrys* and *Xenophrys*. The latter diversifications accelerated only in the Late Miocene, coincident with formation and intensification of the South Asian monsoon system. Numbers at major nodes indicate node age; horizontal blue bars represent 95% HPD intervals of the inferred estimates. Members of the subfamily Leptobrachiinae and the outgroup pelobatid taxon included in the analyses are not shown. Crown ages of the major taxonomic groups and the respective time range of species-level splits are summarized at the top left. Frog in the inset image is a *Jingophrys* member.

### 3.4. Divergence timing

Our time-calibrated phylogenetic analysis based on secondary calibration points at two well-supported deep nodes estimates the crown group age of the family Megophryidae at 46.9 Ma (95% highest posterior density (HPD): 35.1–61.3 Ma), in the Early Eocene, and that of the subfamily Megophryinae at 25.6 Ma (95% HPD: 23.2–27.9 Ma), in the Late Oligocene (figure 3). These estimates are comparable to those of Mahony *et al.* [5] and considered reliable for understanding the relative divergence ages of descendant lineages. Megophryinae is understood to have originated in Southeast Asia, although whether it originated in northern regions of the continental mainland or in southern insular regions has remained unclear [5] (see §3.6). Regardless, the initial major split between the exclusively or largely insular groups (*Megophrys*, *Pelobatrachus* and *Sarawakiphrys*) and those restricted to continental Southeast Asia is estimated at 23.7 Ma (95% HPD: 21.2–26.2 Ma; Late Oligocene; figure 3). This broadly coincides with a period of rapid restructuring and subsequent fragmentation of the continental Sundaland plate, which was driven by tectonic events associated with collision of the Indian plate with Eurasia that began around 55 Ma and was followed by the formation of major mountain ranges, plateaus and river systems that shape the present-day physiography of South and Southeast Asia [20]. The next major genus-level clades in the subfamily are estimated to have originated after the Oligocene–Miocene transition (22.0–17.4 Ma). The exclusively mainland groups—*Boulenophrys*, *Grillitschia*, *Jingophrys*, *Ophryophryne* and *Xenophrys*—originated during the Early Miocene (19.4–17.4 Ma), with species-group-level divisions during the Mid-to-Late Miocene (15.9–9.5 Ma). This was followed by within-clade diversification of these groups beginning around Mid-Miocene but accelerating only around Late Miocene (starting after around 10 Ma; figure 3) (see §3.5). Rapid diversification is observed particularly in clades occupying regions of continental Asia that today are regarded as hotspots of megophryine distribution: Southeast and Southwest China, Indo-Burma and Himalayas. Of the two most speciose genera, *Boulenophrys* (crown age 9.5 Ma; 95% HPD: 7.4–11.7 Ma) experienced a pronounced burst of speciation around 7–5 Ma in the Late Miocene and Early Pliocene, whereas *Xenophrys* (crown age 14.4 Ma; 95% HPD: 11.8–17.0 Ma) had a more gradual species accumulation around 10–4 Ma through the Mid-to-Late Miocene and Early Pliocene (see §3.5). *Jingophrys*, which now stands alongside *Brachytarsophrys* as the next most speciose genus, also experienced rapid speciation around the same time, through Mid-to-Late Miocene and Early Pliocene (11.3–4.1 Ma), even though the major group-level clades and subsequent initial splits were established earlier during the Mid-Miocene. The crown age of the MRCA of *Jingophrys* is estimated at 15.9 Ma (95% HPD: 12.7–18.8 Ma) in the Mid-Miocene (figure 3). Thus, ancestors of this geographically restricted group were already present in the Indo-Burma region or Himalayas during the period of accelerated Himalayan–Tibetan uplift and reconfiguration of major river systems [21,94]. Subsequent formation and intensification of the modern South Asian monsoon system beginning around 13 Ma [22,95] likely was a major driver of the inferred *in situ* diversification of *Jingophrys* over little more than 7 Myr, from 11.3 to around 4.1 Ma. The same driver may also have promoted speciation in Himalayan lineages of *Xenophrys* (figure 3).

Within *Jingophrys*, the estimated crown ages of the *J. pachyproctus* and *J. vegrandis* species groups are 11.3 and 7.9 Ma, respectively. The oldest species-level splits are between *Jingophrys hrdy* sp. nov. and *Jingophrys taniitatii* sp. nov. + *J*. cand. sp. B (7.9 Ma), between *Jingophrys siangensis* sp. nov. and *Jingophrys tanogogo* sp. nov. + *J. vegrandis* (7.1 Ma) and between *J. zhouii* and the rest of the *J. vegrandis* group (7.0 Ma). Remaining sister groups in the *J. pachyproctus* species group were established between 5.1 Ma (*J. pachyproctus* and *Jingophrys wularsoni* sp. nov) and 4.2 Ma (*Jingophrys taniitatii* sp. nov. and *J*. cand. sp. B), and in the *J. vegrandis* species group between 5.9 Ma (*Jingophrys tanogogo* sp. nov. and *J. vegrandis*) and 4.1 Ma (*J. feii* and *J*. cand. sp. A). Splits in the *J. yeae* and *J. zhaoermii* species complexes started 6.1 Ma (*J. zhaoermii* sensu stricto) and continued until the establishment of *J. yeae* sensu lato, the youngest species-level clade, 2.5 Ma age. Ages of all new and candidate species in the present study range between 7.9 and 5.1 Ma (five new species) and 4.2–4.1 Ma (two unnamed candidate species). These estimates are comparable to those of established species-level clades in *Xenophrys* (7.6–2.1 Ma). However, recognized species-level lineages are much younger in *Boulenophrys* (6.3–1.1 Ma), and ages of some unverified species may be even younger (0.9–0.3 Ma). Other, less-speciose genera within Megophryinae show variable divergence ages of established species-level clades—younger in *Atympanophrys* (2.4–2.0 Ma), *Brachytarsophrys* (4.7–0.9 Ma) and *Ophryophryne* (5.8–2.5 Ma), but older in *Megophrys* (10.5–7.3 Ma) and *Pelobatrachus* (8.5–5.2 Ma) (figure 3).

### 3.5. Diversification patterns

Diversification rates and lineage accumulation over time across Megophryinae present an interesting dynamic that supports the timing of divergence discussed above (figure 3). Distribution of the DR statistic as a tip-level estimator of speciation rate shows rate heterogeneity across branches of the phylogeny (figure 4*a*). Corroborating the results presented above, higher rates are observed in clades distributed in continental Asia. In particular, the highest rates are seen in *Boulenophrys* and *Brachytarsophrys*, followed by moderately high rates in *Xenophrys* and *Jingophrys*. Other genera, especially those restricted largely to insular Southeast Asia (e.g. *Megophrys* and *Pelobatrachus*), show relatively slower rates over time. Both the lineage-through-time (LTT) and multiple-lineage-through-time (MLTT) plots further confirm these patterns, both within Megophryinae overall and within the three subsampled genera: *Boulenophrys*, *Xenophrys* and *Jingophrys* (figure 4*b*). The subfamily experienced an early burst of rapid speciation during the Late Oligocene and Early Miocene, coincident with periods of active Himalayan–Tibetan uplift. The orogeny was itself a result of India–Eurasia collision, which rapidly restructured landmasses and changed physiography in regions constituting present-day South and Southeast Asia [20]. Slightly slower rates of lineage accumulation through the Mid-Miocene were followed by another profound burst of rapid speciation starting around 10 Ma, in the Late Miocene. This latter burst coincided with formation and intensification of the Asian monsoon system, which likely created new ecological niches for megophryine frogs. The highly significant negative deviation of the Pybus and Harvey's gamma (*γ*) value from an expected constant-rate pure-birth null model for Megophryinae (*γ* = –4.76, *p* < 0.0001), as well as those for *Boulenophrys* (*γ* = –6.86, *p* < 0.0001), *Xenophrys* (*γ* = –4.53, *p* < 0.0001) and *Jingophrys* (*γ* = –2.34, *p* = 0.0191), confirm these findings. Each of these genera clearly shows an upward and rapid lineage accumulation curve beginning immediately after the 10 Ma timepoint in the Late Miocene and continuing until around the Miocene–Pliocene boundary (about 5 Ma). All three curves eventually plateaued, although *Boulenophrys* experienced further steady lineage accumulation through the Pliocene (figure 4*b*).

**Figure 4.**
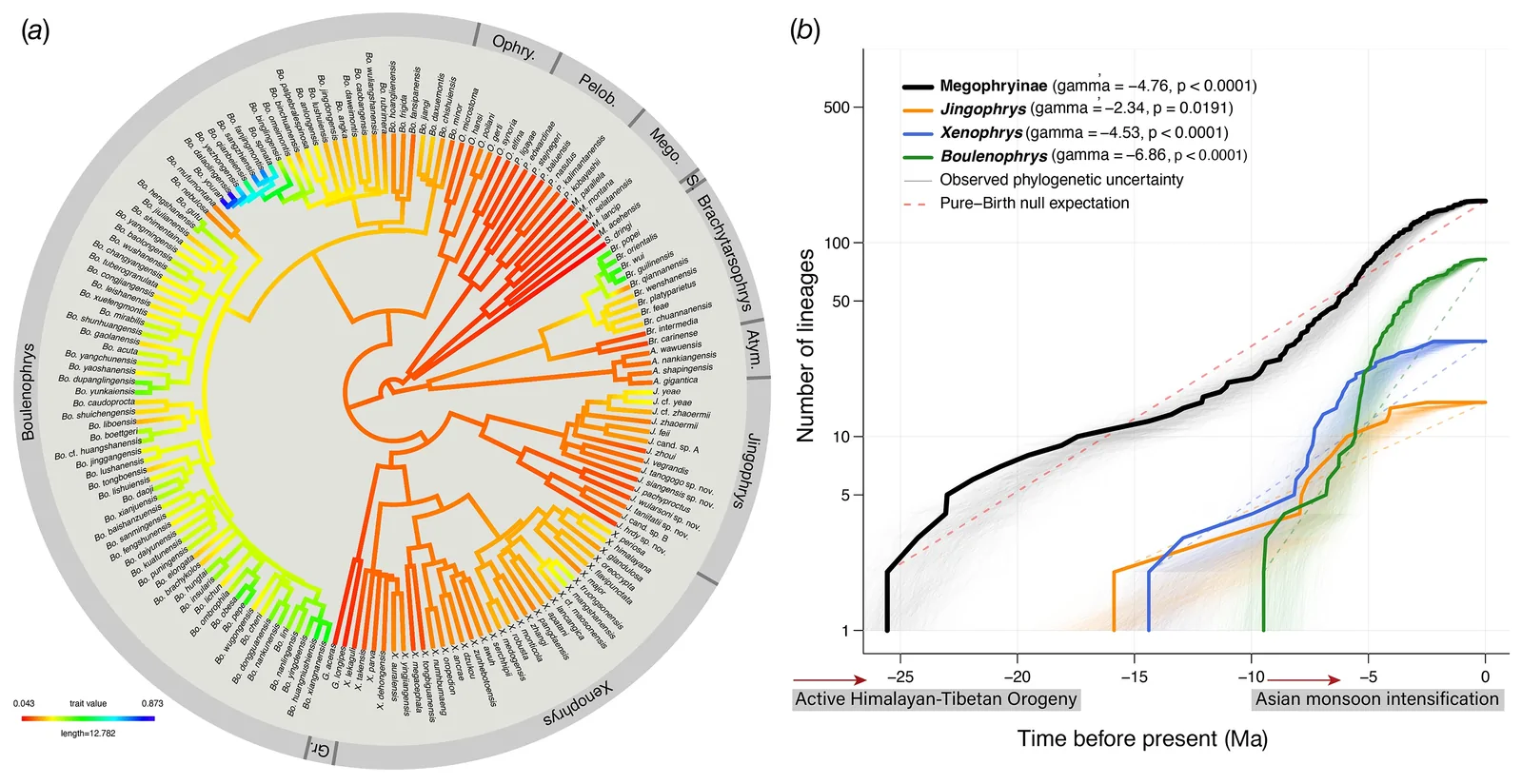
Diversification rates (DRs) across the subfamily Megophryinae over time, based on the concatenated multi-locus dataset of mitochondrial and nuclear DNA. (*a*) DRs traced along branches of the time-calibrated MCC highlight rate heterogeneity among taxonomic groups. Cooler colours represent faster rates; warmer colours represent slower rates; (*b*) LTT plots based on MCC for the entire subfamily Megophryinae (black) and three genera separately: *Jingophrys* (orange), *Xenophrys* (blue) and *Boulenophrys* (green). The corresponding coloured shading denotes MLTT plots for 1000 sampled posterior trees. Expectations for the pure-birth null model are indicated by dashed lines. The Pybus and Harvey's gamma (*γ*) statistics and associated *p*-values for each tree highlight lineage accumulation and diversification dynamics through time. A significant early burst diversification observed in Megophryinae coincides with active periods of Himalayan–Tibetan orogeny. This is followed by another pronounced late burst of rapid diversification, particularly evident in *Boulenophrys*, *Xenophrys* and *Jingophrys* (in order), starting around 10 Ma and coinciding with intensification of the Asian monsoon system.

### 3.6. Biogeography of Megophryinae

Megophryinae-wide ancestral range reconstructions offer additional insights into the historical biogeography of the subfamily, which today is distributed across Asia. Of the six models tested, DEC+J emerged as the best-supported model of inference based on it having the lowest Log-Likelihood (–213.1) and AICc (432.3) scores and the highest AICc weight (0.95). The nested DEC model was second best, with close values for LnL and AICc. However, a likelihood ratio test comparison between DEC and DEC+J yields a statistically significant improvement (*p* = 0.005) in model fit by adding the jump dispersal (j) parameter, which matches the AICc weight of DEC+J capturing 95% of the total support among all six models (supplementary material, table S4).

Two parameters dominate the best model: long-distance jump dispersals (*j* = 0.0043) that explain founder-event speciation or cladogenesis, and dispersal events over shorter distances (*d* = 0.0047) that result in gradual range expansions; only very low levels of local extinction are inferred. These results suggest that diversification and geographic range expansion of Megophryinae (figure 5) were driven primarily by long-distance colonization events at ancestral nodes that likely crossed significant geographical barriers and established populations in new areas. An example is the MRCA of *Jingophrys*, which crossed from Indo-Burma to the Himalayas around Mid-Miocene (15.9 Ma), gradually accumulated lineages through Mid-Miocene, and then underwent rapid *in situ* diversification within the Himalayas after the 10 Ma timepoint, and specifically between 7.9 and 2.5 Ma for extant species-level lineages (figure 5). These findings reinforce conclusions derived from the timing and pattern of diversification discussed above (figures 3 and 4). A subsequent single dispersal event from the Himalayas back to Indo-Burma by the MRCA of the clade containing *J. feii* + *J.* cand. sp. A is inferred around the Miocene–Pliocene boundary.

**Figure 5.**
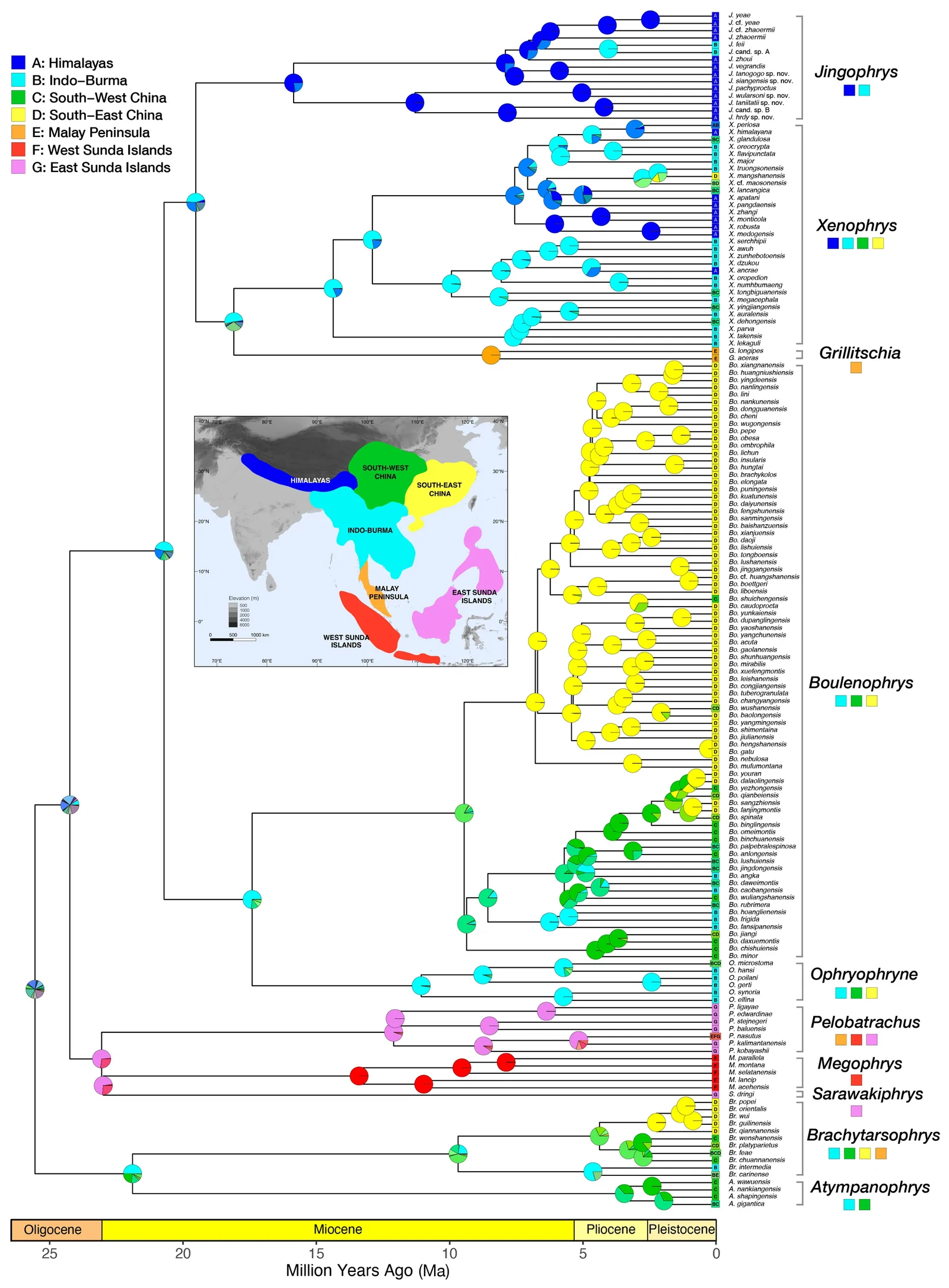
Historical biogeography of the subfamily Megophryinae based on ancestral range reconstructions in BioGeoBEARS. The best-supported DEC+J model (AICc = 432.3, AICc weight = 0.95) infers cladogenesis through long-distance jump dispersals (*j* = 0.0043) and gradual range expansion through shorter dispersals (*d* = 0.0047) as primary drivers of megophryine evolution and biogeography. These events coincided with periods of rapid continental restructuring across Asia driven by collision of Indian and Eurasian plates, which resulted in Himalayan–Tibetan orogeny. In particular, the MRCA of *Jingophrys* colonized the Himalayas in the Mid-Miocene through a founder speciation event followed by extensive *in situ* diversification over time. *Jingophrys*, as well as closely related groups *Xenophrys* and *Boulenophrys*, subsequently expanded their ranges in the Himalayas and/or adjacent Indo-Burma and Southwest China. Their more pronounced speciation coincided with intensification of the Asian monsoon, which started around 10 Ma in the Late Miocene. Colours denote broad geographic areas depicted in the inset map and defined based on present-day distributions, as indicated by the terminal nodes; pie charts represent inferred relative probabilities of an ancestral area or multi-area ranges at the internal nodes. Areas currently occupied by each genus are indicated on the right.

The MRCA of Megophryinae is inferred to have a broad ancestral range across Asia without a clear area of geographic origin, either in continental or insular regions. However, the MRCA of basal clades (*Atympanophrys* + *Brachytarsophrys*) is inferred as being restricted to the mainland (likely Indo-Burma) in the Early Miocene, from where it later (starting around the Miocene–Pliocene boundary) expanded and dispersed into adjacent areas, largely northward into Southwest China (both genera) and Southeast China (*Brachytarsophrys* only), and also southward into the Malay Peninsula (a single *Brachytarsophrys* species; figure 5). The MRCA of primarily insular lineages (*Megophrys*, *Pelobatrachus* and *Sarawakiphrys*) appears to have been established through a single dispersal event early in megophryine diversification, around the Oligocene–Miocene boundary. Around the same time, the single known member of *Sarawakiphrys* originated in the East Sunda Islands. *Megophrys* originated in the West Sunda Islands, where its distribution expanded between Mid-to-Late Miocene. *Pelobatrachus* also originated and largely expanded within the East Sunda Islands through Mid-to-Late Miocene; a single species found more widely in both East and West Sunda Islands as well as the Malay Peninsula suggests another jump dispersal event back to the mainland around the Miocene–Pliocene boundary (figure 5). Among continental lineages, the MRCA of *Boulenophrys* and *Ophryophryne* is inferred with high probability to have originated in Indo-Burma around Early Miocene. *Ophryophryne* subsequently expanded its distribution mostly within Indo-Burma during the Late Miocene, with a single species dispersing into Southwest and Southeast China around the Miocene–Pliocene boundary. The MRCA of *Boulenophrys*, the most speciose genus, is inferred with high probability to have been present in Southwest China around 10 Ma. It diverged into two major lineages that underwent contrasting patterns of range expansion and dispersal: one with ancestral ranges and gradual expansion in Indo-Burma and Southwest China through the Late Miocene, as well as a few later dispersals into Southeast China around the Pliocene–Pleistocene boundary; the second with a restricted ancestral distribution in Southeast China and only a few later dispersals into Southwest China during the Pliocene (figure 5). The MRCA of *Xenophrys* and *Grillitschia* is inferred to have originated in Indo-Burma during Early Miocene, with a greater probability that the MRCA of *Xenophrys* ancestrally occupied Indo-Burma around Mid-Miocene. *Grillitschia* likely became established in the Malay Peninsula through a single dispersal event around 10 Ma. *Xenophrys* further diversified and expanded geographically along two slightly different trajectories—one with its ancestral distribution likely restricted to Indo-Burma and gradual expansion within that region through the Late Miocene, and a few dispersals into adjacent Southwest China and Himalayas starting around the Pliocene–Pleistocene boundary; and the second with a higher probability of the MRCA having crossed into the Himalayas during the Late Miocene, followed by a dispersal back into Indo-Burma as well as dispersals into Southwest and Southeast China, potentially through Late Miocene to Pliocene. Patterns inferred for *Xenophrys* provide an additional instance of *in situ* diversification in the Eastern Himalayas, albeit one that is limited in comparison to the more intensive Himalayan diversification of *Jingophrys* (figure 5).

### 3.7. Distribution patterns within *Jingophrys*

The present-day geographical distribution of *Jingophrys* reveals patterns of species richness and elucidates potential drivers and modes of spatial diversification. Of the total diversity recognized here, 11 named species and at least two unnamed distinct lineages, only two taxa, *J. feii* and *J*. cand. sp. A, extend outside the Eastern Himalayas into the Indo-Burma region (figure 6). All remaining members are restricted exclusively to mountain ranges in the Eastern Himalayas. While distributions of the two species groups overlap, the *J. vegrandis* group is much more widely distributed; the *J. pachyproctus* group is found only in two narrow regions (figure 6*a*). Furthermore, of the 11 named species, eight are concentrated in just two regions: (i) Tale Valley and Ziro Valley, Subansiri River basin, Lower Subansiri District, Arunachal Pradesh State, India: *J. vegrandis* group— *Jingophrys tanogogo* sp. nov.; *J. pachyproctus* group—*Jingophrys hrdy* sp. nov., *Jingophrys taniitatii* sp. nov. and *Jingophrys wularsoni* sp. nov. (figure 6*b*). (ii) Medog County, Yarlung Tsangpo River basin, Tibet Autonomous Region (Xizang), People's Republic of China: *J. vegrandis* group—*J. yeae*, *J. zhaoermii* and *J. zhouii*; *J. pachyproctus* group—*J. pachyproctus* (figure 6*c*). Only two of these eight species, *J.* cf. *yeae* and *J.* cf. *zhaoermii*, are additionally found outside of these regions, and these outlier populations are genetically divergent from *J. yeae* sensu stricto and *J. zhaoermii* sensu stricto, respectively. Members occurring in the remaining areas of the *Jingophrys* range, all in Arunachal Pradesh State, India, are the sole representatives of the genus in those localities: *J. vegrandis* in Kameng River basin (Sessa), West Kameng District; *Jingophrys siangensis* sp. nov. in Siang River basin (Kebang, Pangin and Yembung), East Siang District; *J.* cf. *yeae* in Dibang River basin (Anini, Etalin and Tiwari Gaon), Lower Dibang District; and *J.* cf. *zhaoermii* in Lohit River basin (Hayuliang-Chaglagom and Kupha), Anjaw District. Overall, the geographic distribution of *Jingophrys*, as currently understood, appears to have been shaped by the five major rivers that flow from north to south and subdivide the Himalayan region from west to east: Kameng River, Subansiri River, Yarlung Tsangpo River (People's Republic of China) that becomes Siang River (India), Dibang River and Lohit River. Additional testing of regional-scale dispersal-vicariance scenarios that might yield the same river system-associated distribution patterns can shed more light on this biogeographical hypothesis. Future field surveys might also reveal additional diversity of *Jingophrys* within regions surveyed in the present study, but especially in intervening and understudied areas.

**Figure 6.**
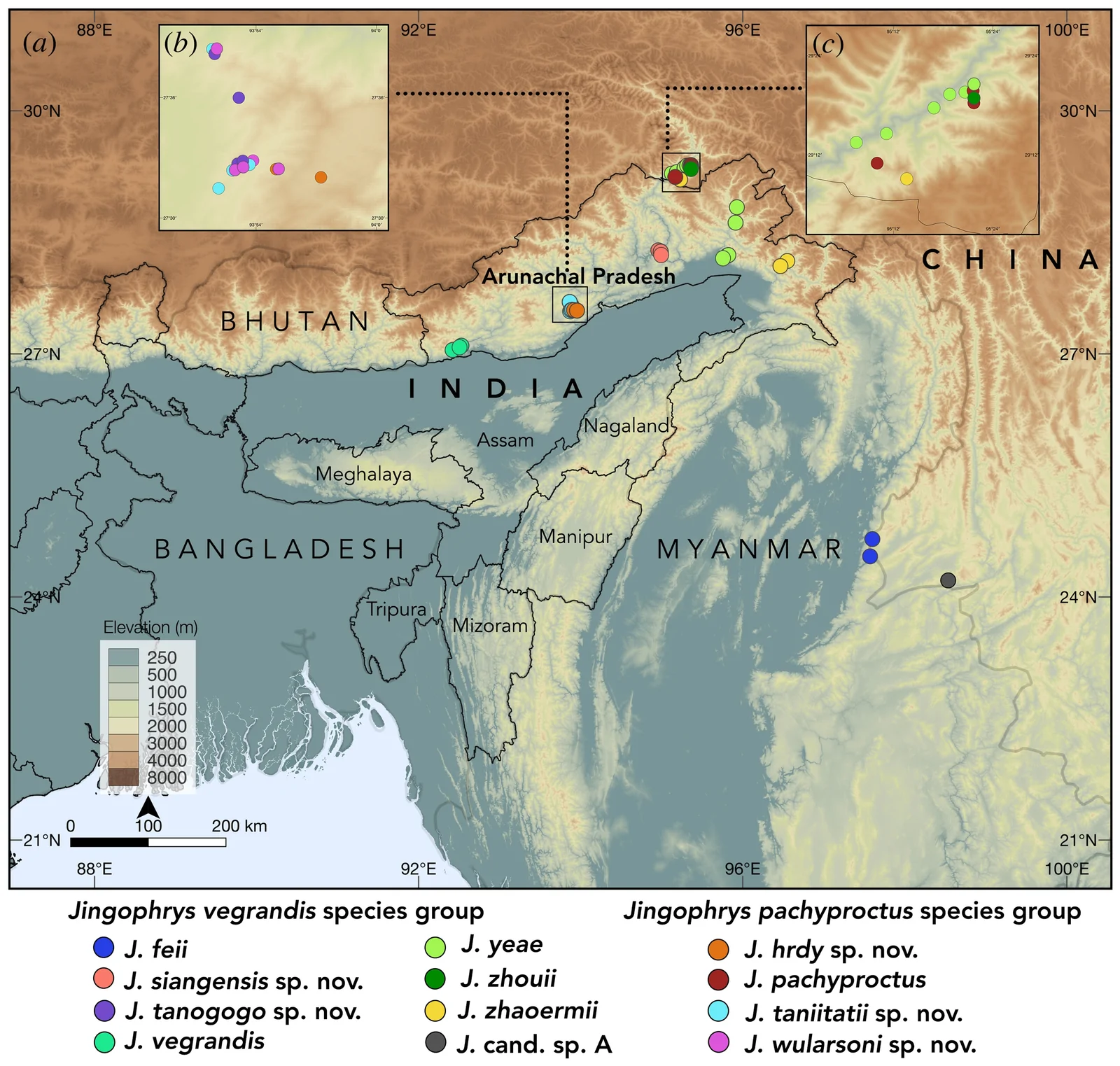
(*a*) Geographical distribution of the genus *Jingophrys*, including the type localities and new distribution records from India reported in the present study; (*b*, *c*) Insets present enlarged views of regions with high species richness: (*b*) Lower Subansiri District, Arunachal Pradesh State, India, and (*c*) Medog County, People's Republic of China. Coordinates and source information are presented in table S2 in the supplementary material.

The broad elevational range of *Jingophrys*, from river valleys as low as 300 m to mountain peaks at 2600 m, likely provided opportunities for *in situ* diversification within otherwise circumscribed geographic regions. At the species group level, the *J. vegrandis* group occupies the broadest elevational range, 300–2590 m, with *Jingophrys siangensis* sp. nov. found at the lowest elevations (304–314 m) and *J. zhaoermii* at the highest (2590 m). By contrast, the *J. pachyproctus* group occupies a much narrower range, 1530–2344 m, with *J. pachyproctus* and *Jingophrys wularsoni* sp. nov. occupying lower elevations (1530–2003 m) and *Jingophrys hrdy* sp. nov. and *Jingophrys taniitatii* sp. nov. found slightly higher (1740–2344 m). The widest elevational range documented in the *J. pachyproctus* group is only approximately 381 m, for *Jingophrys wularsoni* sp. nov. By contrast, *J. yeae* in the *J. vegrandis* group has an approximately 1100 m-wide range, although it is approximately 1810 m for *J. zhaoermii* + *J.* cf. *zhaoermii* populations combined (supplementary material, table S2). Within the two regions with high species richness, some closely occurring species occupy different elevational ranges, which suggests a possible role of elevation in promoting speciation. Examples include, in the Medog region, *J. zhaoermii* (2590 m) separated from the closely related *J. zhouii* (2003 m) and from *J. pachyproctus* (1530–1780 m) of a different species group; and in the Lower Subansiri region, separation of *Jingophrys hrdy* sp. nov. (2121–2344 m) from the closely related *Jingophrys taniitatii* sp. nov. (1740–1945 m) and from *Jingophrys tanogogo* sp. nov. (1729–1945 m) of a different species group. Microhabitat characteristics and behaviour likely also play a role in niche partitioning among sympatric species of the same or different species group. In India, for example, *Jingophrys tanogogo* sp. nov., *Jingophrys taniitatii* sp. nov. and *Jingophrys wularsoni* sp. nov. of the same or different species groups occur together at three sites (Pange, Tajgi and Tarin Tibe); and *Jingophrys hrdy* sp. nov. and *Jingophrys wularsoni* sp. nov. of the same species group occur together at one site (Tasi Biddang). In the People's Republic of China, sympatric species pairs include *J. pachyproctus* and *J. yeae* (Bari village) and *J. pachyproctus* and *J. zhaoermii* (Renqingbeng Temple), members of each pair belonging to different species groups [31] (supplementary material, table S2). Additional studies of the ecology and microhabitat preferences of these species are needed to obtain a better understanding of these interesting patterns.

### 3.8. Morphometric differentiation

Morphological analyses of new *Jingophrys* samples reveal several reliable characters for taxonomic identification of candidate lineages. Moreover, multivariate statistical analyses that quantitatively assess differentiation among members of the two species groups provide additional support for the recognition of at least five new species. The PCA of external morphometric data for five *J. vegrandis* group members from India—*Jingophrys siangensis* sp. nov., *Jingophrys tanogogo* sp. nov., *J. vegrandis*, *J.* cf. *yeae* and *J.* cf. *zhaoermii*—yields five PCs with eigenvalues > 1 that collectively explain 77.1% of the total variation in the dataset (supplementary material, tables S5 and S6). The first PC accounts for 26.9% of the variation, with head length (HL), length of finger III and four hind-limb measurements (TL, SHL, FL and TFOL) contributing most heavily. PC2 accounts for 18.2% of the variance, which is explained mostly by UEW and length of finger III. PC1 and PC2 together account for 45.1% of total variance, and the corresponding scatterplot shows considerable separation of the five group members (figure 7*a*). Of the remaining three PCs, PC3 accounts for a significant 16.4% of total variation, with TYE, two forelimb measurements (FAL and HAL) and lengths of fingers II and IV contributing most heavily. Finally, PC4 and PC5 account for 9.4 and 6.2% of total variation, respectively, with heavy loadings largely for head characters (supplementary material, table S6). The PCA of three members of the *J. pachyproctus* group from India—*Jingophrys hrdy* sp. nov., *Jingophrys taniitatii* sp. nov. and *Jingophrys wularsoni* sp. nov.—yields four PCs with eigenvalues > 1 that collectively explain 86.1% of the total variation observed in this dataset (supplementary material, tables S5 and S6). The first PC explains 39.8% of total variation, with TYD as the lone significantly contributing variable. PC2 explains 24.1% of the variance, with body size, HW, EL, EN and NS, as well as TL and SHL contributing most heavily. PC1 and PC2 together account for 63.9% of total variance, and the corresponding morphospace shows wide separation of all three group members (figure 7*b*). The two remaining significant PCs (3 and 4), which together account for 22.2% of the variation, are loaded heavily by characters relating to the head, forelimb, and finger lengths (supplementary material, table S6). The above results, when combined with several qualitative characters, particularly those relating to body colouration and markings, skin texture and granulation, snout shape, foot webbing, dermal extensions and prominence of foot tubercles (figure 8), enable morphological characterization and differentiation of *Jingophrys* species, as presented in the following taxonomic accounts.

**Figure 7.**
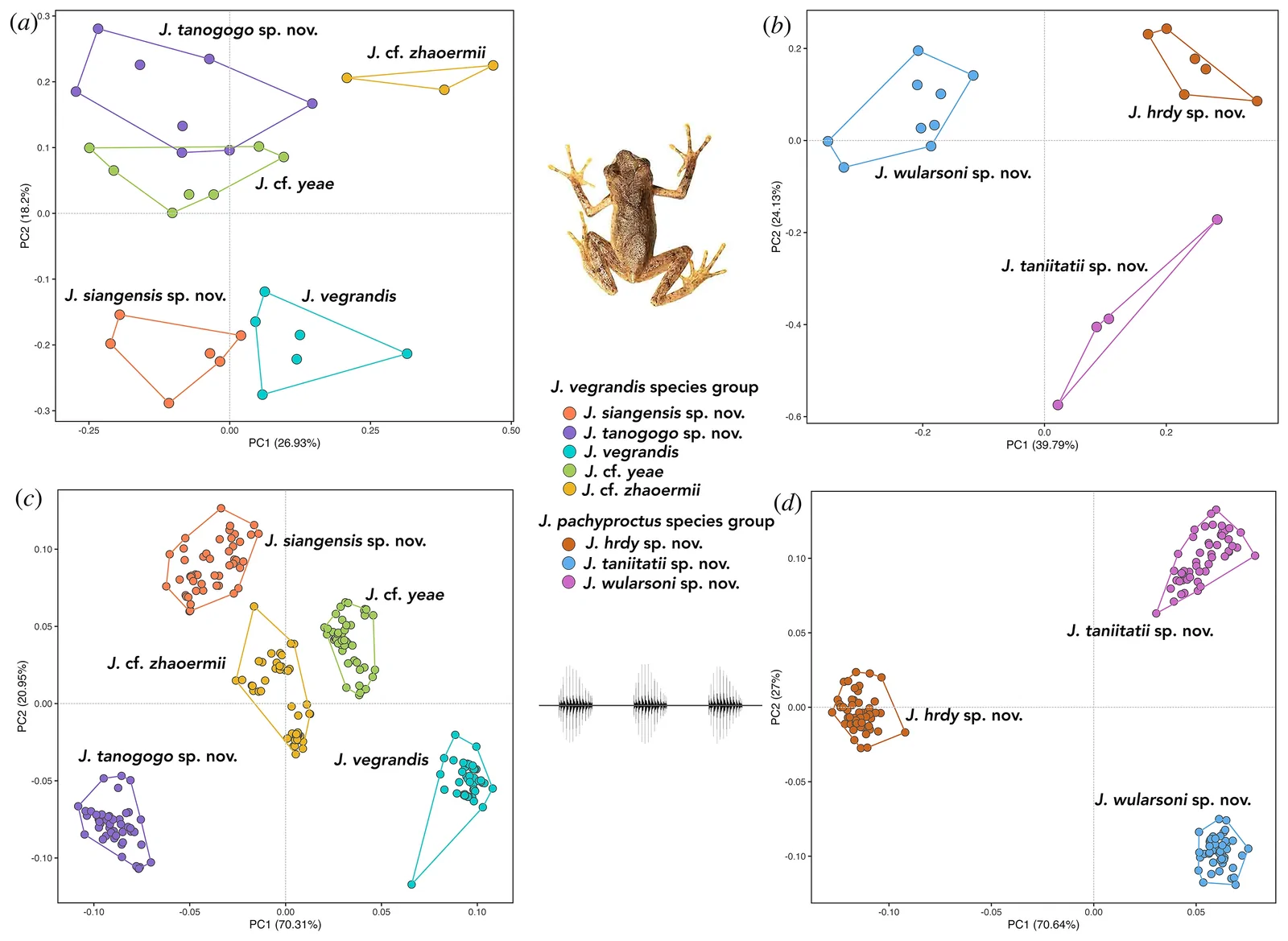
Integrative evidence from external morphology and behaviour for species recognition in *Jingophrys*. (*a*, *b*) PCA of morphometric data from adult male specimens: (*a*) scatterplot of first two PCs explaining 45.1% of the total variance among five species of the *J. vegrandis* species group, and (*b*) scatterplot of first two PCs explaining 63.9% of the total variance among three species of the *J. pachyproctus* species group; (*c*, *d*) PCA of acoustic properties of male calls: (*c*) scatterplot of first two PCs explaining 91.3% of the total variance among five species of the *J. vegrandis* species group, and (*d*) scatterplot of first two PCs explaining 97.6% of the total variance among three species of the *J. pachyproctus* species group.

**Figure 8.**
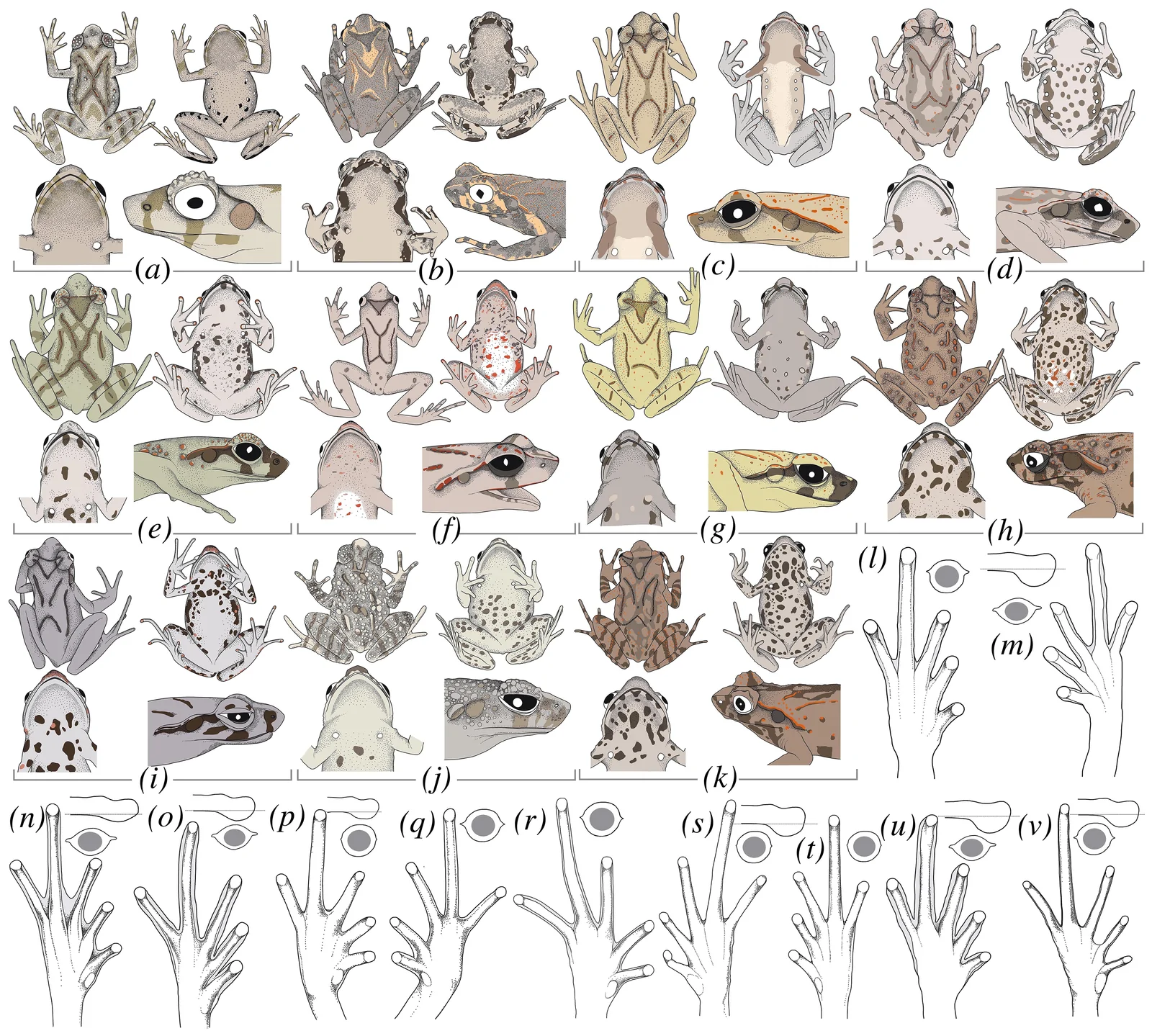
Schematic comparison of external morphology of all 11 recognized *Jingophrys* species. *Jingophrys vegrandis* group: (*a*, *l*)* J. feii*, (*b*, *m*)* Jingophrys siangensis* sp. nov., (*c*, *n*)* Jingophrys tanogogo* sp. nov., (*d*, *o*)* J. vegrandis*, (*e*, *p*)* J. yeae,* (*f*, *q*)* J. zhoui,* (*g*, *r*)* J. zhaoermii*; *J. pachyproctus* group: (*h*, *s*)* Jingophrys hrdy* sp. nov., (*i*, *t*)* J. pachyproctus,* (*j*, *u*)* Jingophrys taniitatii* sp. nov., (*k*, *v*)* Jingophrys wularsoni* sp. nov. In *a–k*, four features are arranged clockwise in each panel: skin granulations (body in dorsal view), colour and markings (body in ventral view), lateral markings and tubercles (head in lateral view) and snout shape (head in ventral view). In *l–v*, aspects of foot morphology are arranged clockwise in each panel: webbing and dermal extensions on toes (ventral view), projection of tubercle on toe tip IV (lateral view) and skin extension along toe IV (cross section). Illustrations: S. D. Biju.

### 3.9. Acoustic differentiation

Multivariate analyses of acoustic properties of male calls (supplementary material, table S7) yield significant additional support for species recognition, especially compared with the more highly conserved external morphology. PCA of five species of the *Jingophrys vegrandis* group from India results in just two PCs with eigenvalues > 1, which together explain 91.3% of the total variation in the dataset (supplementary material, table S8). PC1, which accounts for 70.3% of the variation, is loaded heavily by three temporal properties—call duration, rise time and fall time. PC2 accounts for 21% of the variation, with one temporal (pulse rate) and one spectral (dominant frequency) property as major contributing variables. The scatterplot of PC1 and PC2 shows clear separation among all five species (figure 7*c*). For the *J. pachyproctus* group, the PCA results in three PCs with eigenvalues > 1 that collectively explain nearly all variation in this dataset (99.4%); PC1 and PC2 together explain 97.6% of the variation (supplementary material, table S8). PC1 accounts for 70.6% of the variation, which is explained mostly by three temporal properties—call duration, fall time and number of pulses. PC2 accounts for the remaining 27% of variation, which is explained largely by one temporal (pulse rate) and one spectral (dominant frequency) property. The scatterplot of PC1 and PC2 shows clear separation among all three species (figure 7*d*).

Additional univariate comparisons reinforce the above multivariate statistical results showing that acoustic properties of male vocalizations effectively differentiate among species of *Jingophrys*. The calls of different species can be reliably told apart based on single-variable comparisons of several properties, such as frequency, duration, rise time, pulse rate and pulses per call. For example, mean pulse rate ranges from 71.03 pulses per second in *J. vegrandis* to 193.66 pulses per second in *Jingophrys siangensis* sp. nov., while call duration ranges from 31.1 ms in *Jingophrys tanogogo* sp. nov. to 746 ms in *J*. cf. *pachyproctus*. Detailed univariate comparisons between individual species pairs are provided in the ‘Call comparison’ section of each systematic account below, and summary statistics for each acoustic property in each species are presented in table S7 in the supplementary material.

### 3.10. Integrative evidence for species and species-group recognition

Integration of molecular phylogenetic, morphometric and acoustic data provides unambiguous support for recognition of two distinct species groups and five new species of *Jingophrys*. Hence, we recognize a total of 11 *Jingophrys* species, six named previously and five described here. In addition, we identify two distinct candidate species—*J*. cand. sp. A and *J*. cand. sp. B—that are well supported phylogenetically but whose formal description requires additional detailed taxonomic investigation. Below, we present comprehensive systematic accounts for the eight Indian species, which include five new to science and two new country records that represent a significant increase from the lone previously confirmed species (*J. vegrandis*) and one proposed record (as *J. pachyproctus*).

### 3.11. Systematic accounts and descriptions of new species

Family: MEGOPHRYIDAE Bonaparte, 1850

Subfamily: MEGOPHRYINAE Bonaparte, 1850

Genus: *Jingophrys* Lyu and Wang, 2023

Common name: Diminutive horned frogs

**Taxonomic note.** *Jingophrys* was first identified as a distinct megophryine lineage (the ‘*Xenophrys vegrandis* species group’) [5] with a single species from northeast India, *Megophrys vegrandis* Mahony, Teeling and Biju, 2013. It was subsequently revised as ‘*Megophrys* (*Xenophrys*) *vegrandis* species group’ with an additional member, *Megophrys pachyproctus* Huang, 1981 [12]. Before these studies, Chen *et al.* [6] had also reported the distinct phylogenetic position of this group (as subclade ‘B3’) within *Xenophrys* along with another unnamed population (as ‘*X*. sp. 20’; *Jingophrys* cand. sp. A, in the present study). More recently, this group was recognized as a distinct genus [15] with transfer of another three species, *Megophrys feii* Yang, Wang and Wang, 2018, *Megophrys yeae* Shi, Zhang, Xie, Jiang, Liu, Ding, Luan and Wang, 2020 and *Megophrys zhouii* Shi, Zhang, Xie, Jiang, Liu, Ding, Luan and Wang, 2020, followed by an additional recent description (*Jingophrys zhaoermii* Huang, Liu, Chen and Yuan, 2026). Before the present study, the genus comprised a total of six species [19].

**Etymology.** The etymology of the name *Jingophrys* was reported in the original description as a combination of two words—‘*jing*’ (Pinyin), a Chinese character reportedly meaning ‘lilliputian’ in English and referring to the small body size of the frogs in this genus, combined with the suffix ‘*phrys*’, a common generic root for Asian horned toads [15]. The specific scientific nomen ‘*phrys*’ is derived from Greek ‘*Ophrys*’, meaning ‘eyebrow’ in English and likely referring to the prominent raised horn-like dermal structures above the eyes in Asian horned frogs of the subfamily Megophryinae.

**Suggested common name.** English: Diminutive horned toads [15] or Diminutive horned frogs (proposed here for the use of a more inclusive word ‘frogs’ in order to avoid confusion based on the word ‘toads’, which is more commonly used for referring to members of the family Bufonidae).

**Diagnosis.** Small adult size (SVL < 41 mm in males, SVL < 48 mm in females); body slender or stout; tympanum distinct; supratympanic fold narrow and curved over tympanum; maxillary teeth present; vomerine teeth present or absent; subarticular tubercles absent on fingers; dermal ridges present at mid-dorsum; upper eyelid without appendage, except for a single tiny horn-like tubercle at centre of upper eyelid outer margin; dorsolateral dermal ridges present (continuous or discontinuous) or absent; belly with tubercles on each side; and endemic to the Eastern Himalayas and adjacent Indo-Burma.

**Distribution.** The genus is currently known only from the Eastern Himalayas and Indo-Burma regions encompassing the countries of India (Arunachal Pradesh State) and the People's Republic of China (Xizang, Tibet Autonomous Region; and Yunnan Province) (figure 6; supplementary material, table S2).

**Type species.** *Jingophrys feii* (Yang, Wang and Wang, 2018), by original designation [15].

**Species included.** Total 11 currently recognized species. Six species restricted to India: *Jingophrys vegrandis* (Mahony, Teeling and Biju, 2013), *Jingophrys hrdy* sp. nov., *Jingophrys siangensis* sp. nov., *Jingophrys taniitatii* sp. nov., *Jingophrys tanogogo* sp. nov. and *Jingophrys wularsoni* sp. nov.; three species restricted to People's Republic of China: *Jingophrys feii* (Yang, Wang and Wang, 2018), *Jingophrys pachyproctus* (Huang, 1981), *Jingophrys zhouii* (Shi, Zhang, Xie, Jiang, Liu, Ding, Luan and Wang, 2020); two species originally described from People's Republic of China and here also reported from India: *Jingophrys yeae* (Shi, Zhang, Xie, Jiang, Liu, Ding, Luan and Wang, 2020) and *Jingophrys zhaoermii* Huang, Liu, Chen and Yuan, 2026.

**Species groups.** Two recognized here: *Jingophrys pachyproctus* species group (four recognized species, as listed above in ‘Molecular species delimitation’ results); and *Jingophrys vegrandis* species group (restricted here for a subclade of the genus *Jingophrys* with seven recognized species, as listed above in ‘Molecular species delimitation’ results). **Note.** The name ‘*vegrandis* species group’ was formerly used to refer to the ‘*Xenophrys vegrandis*’ species group, which was proposed earlier to contain a single species [5]. Subsequently, its name was treated as *Megophrys* (*Xenophrys*) *vegrandis* species group [12] based on a revised subgeneric classification scheme, along with assignment of a second species. More recently, it was expanded to include all taxa currently included in the new genus *Jingophrys* proposed as part of a genus-level revision [15].

#### 3.11.1. *Jingophrys vegrandis* species group

Until this study, *Jingophrys* was known from India from a single confirmed species, *J. vegrandis*. Here, we report the discovery and formal descriptions of two new species and two new country records for India for this species group. This increases the currently recognized number of species in this group from India to five: *Jingophrys siangensis* sp. nov., *Jingophrys tanogogo* sp. nov., *J. vegrandis*, *J*. *yeae* (reported as *J*. cf. *yeae* for comparative purpose), and *J*. *zhaoermii* (reported as *J*. cf. *zhaoermii* for comparative purpose).

***Jingophrys siangensis* sp. nov.**

(http://zoobank.org/urn:lsid:zoobank.org:act:BE44F009-65B6-4263-AE42-431D4D9393D2) figures 1–10; tables S1–S9 and figures S1 and S2 in the supplementary material

**Figure 9.**
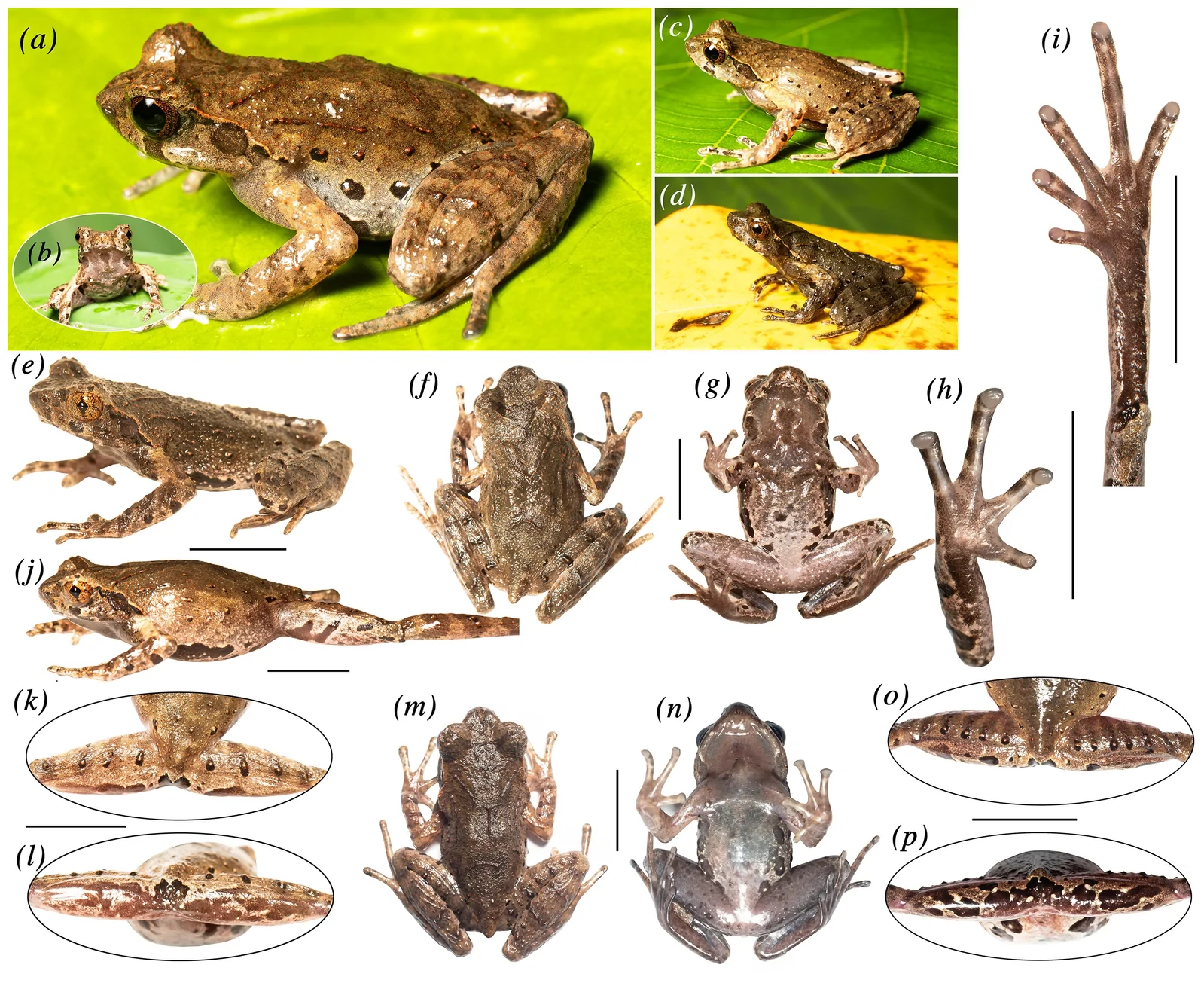
Type series of *Jingophrys siangensis* sp. nov., in life. (*a, m, n*) Holotype (ZSI A 16611, male): (*a*) dorsolateral view, (*m*) dorsal view and (*n*) ventral view; (*c*, *d*) paratypes in dorsolateral view: (*c*) ZSI A 16612 and (*d*) ZSI A 16615; (*b*, *e–l*) paratype (ZSI A 16612, male): (*b*) frontal view, (*e*) dorsolateral view, (*f*) dorsal view, (*g*) ventral view, (*h*) ventral view of hand, (*i*) ventral view of foot, (*j*) lateral view of flank, groin and thigh, (*k*) dorsal view of thighs and (*l*) posterior view of thighs; (*o*, *p*) ZSI A 16613 (male): (*o*) dorsal view of thighs and (*p*) posterior view of thighs. Scale bars = 10 mm. Photographs: S. D. Biju and T. Tajo.

**Figure 10.**
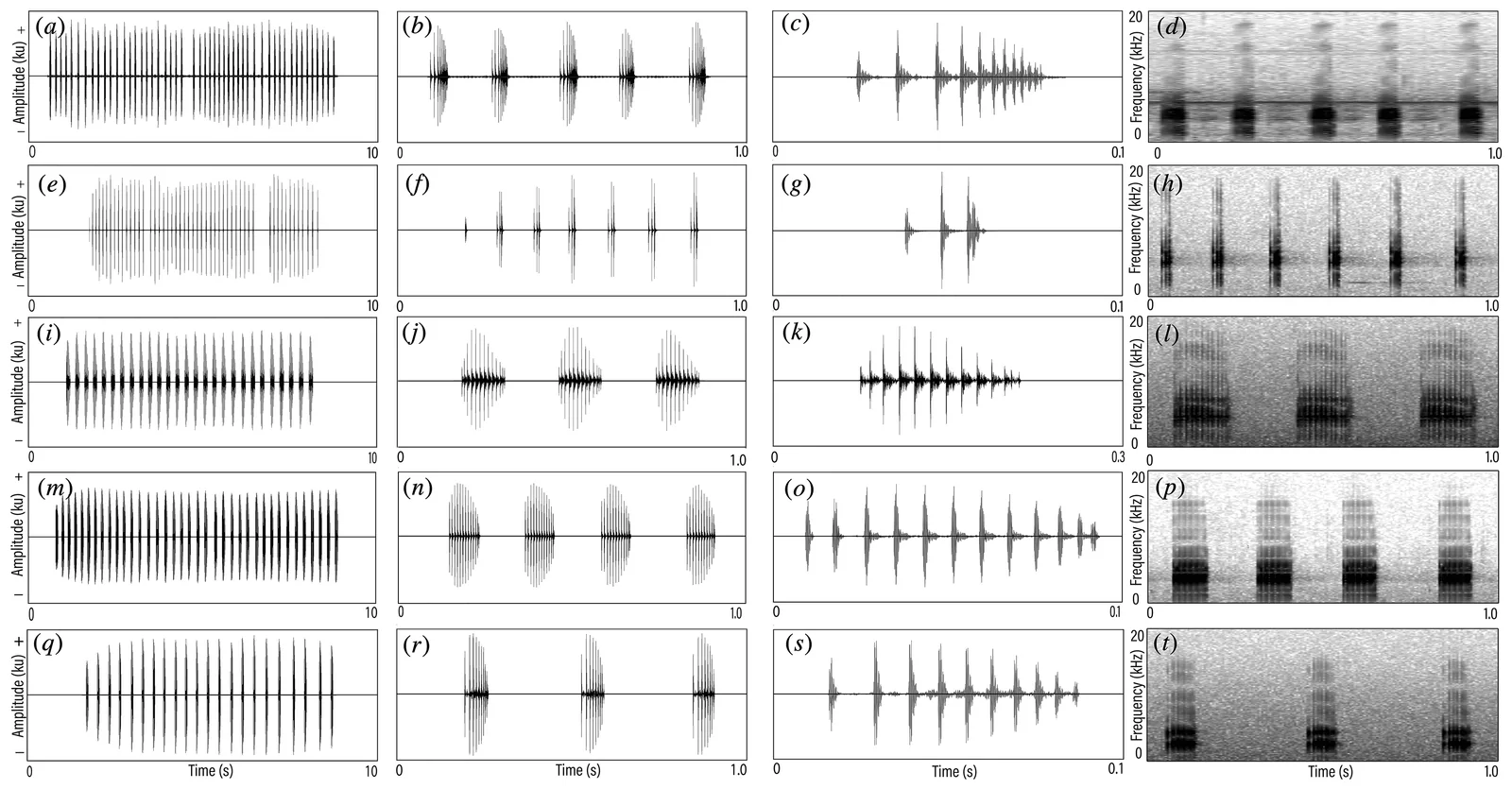
Acoustic properties of typical male calls of five species in the *Jingophrys vegrandis* species group. (*a–d*) *Jingophrys siangensis* sp. nov.: (*a*) oscillogram for a 10-s call segment, (*b*) oscillogram for a 1.0-s call segment, (*c*) oscillogram for a 0.1-s call segment and (*d*) spectrogram for a 1.0-s call segment; (*e–h*) *Jingophrys tanogogo* sp. nov.: (*e*) oscillogram for a 10-s call segment, (*f*) oscillogram for a 1.0-s call segment, (*g*) oscillogram for a 0.1-s call segment and (*h*) spectrogram for a 1.0-s call segment; (*i–l*) *J. vegrandis*: (*i*) oscillogram for a 10-s call segment, (*j*) oscillogram for a 1.0-s call segment, (*k*) oscillogram for a 0.3-s call segment and (*l*) spectrogram for a 1.0-s call segment; (*m–p*) *J.* cf. *yeae*: (*m*) oscillogram for a 10-s call segment, (*n*) oscillogram for a 1.0-s call segment, (*o*) oscillogram for a 0.1-s call segment and (*p*) spectrogram for a 1.0-s call segment; (*q–t*) *J.* cf. *zhaoermii*: (*q*) oscillogram for a 10-s call segment, (*r*) oscillogram for a 1.0-s call segment, (*s*) oscillogram for a 0.1-s call segment and (*t*) spectrogram for a 1.0-s call segment.

**Holotype.** An adult male, ZSI A 16611 (SDB 2024.6634), from Kebang (28.2773° N, 94.9576° E, 305 m asl), Siang District, Arunachal Pradesh State, India, collected by SDB, TT, SG and RUK on 23 August 2024.

**Paratypes.** Two adult males, ZSI A 16612–16613 (SDB 2024.6637–6638), collected with the holotype; an adult male, ZSI A 16614 (SDB 2024.6729), from Pangin (28.2628° N, 94.9870° E, 304 m asl), Siang District, Arunachal Pradesh State, India, collected by SDB, TT, SG and RUK on 23 August 2024; and an adult male, ZSI A 16615 (SDB 2024.6652), from Yembung (28.2588° N, 94.9865° E; 314 m asl), Siang District, Arunachal Pradesh State, India, collected by SDB, TT, SG and RUK on 23 August 2024.

**Referred specimen.** An adult male, SDBDU 2024.6636, collected with the holotype.

**Etymology.** The species is named for the Siang region of Arunachal Pradesh State, India, which includes both the East Siang District and the Siang River, where the type series was collected.

**Suggested common name.** Siang horned frog.

**Diagnosis.** *Jingophrys siangensis* sp. nov. can be distinguished from other known congeners by the following combination of characters: small adult size (SVL 28–32 mm); supratympanic fold narrow anteriorly but slightly wide posteriorly; foot webbing rudimentary; lateral fringes on toes well developed; and anterior and posterior surfaces of thighs mottled and blotched with blackish-brown colouration.

**Phylogenetic relationships.** *Jingophrys siangensis* sp. nov. is a member of the *J. vegrandis* species group. Based on our multilocus analyses, it is likely most closely related to a clade containing *Jingophrys tanogogo* sp. nov. and *J. vegrandis*, although this relationship received low support (figures 2 and 3). Within its species group, *Jingophrys siangensis* sp. nov. shows the following DNA sequence divergence in the mitochondrial 16S rRNA gene: 8.1% from *J. feii*; 7.3–7.5% from *Jingophrys tanogogo* sp. nov.; 5.7–6.0% from *J. vegrandis*; 6.9–7.5% from *J. yeae* sensu lato; 6.7% from *J. zhaoermii* sensu lato; 8.1–8.3% from *J. zhoui*; and 5.3% from the undescribed candidate species *J*. cand. sp. A. (supplementary material, table S3).

**Holotype description (measurements in mm).** Adult male (SVL 30.2), slender body; head moderately large, as long as wide (HL 10.6, HW 10.6, MN 8.7; MFE 7.0; MBE 3.9); snout nearly bluntly pointed in dorsal view and subovoid ventral view, strongly acutely protruding in lateral view, snout tip projecting far beyond the lower lip (SP 2.4 mm); loreal region acute, concave; canthus rostralis sharp (angled); dorsal snout slightly concave; eye larger than tympanum but smaller than snout length (EL 3.6, TYD 2.0, SL 4.1); tympanum-to-eye distance (TYE 1.7) shorter than tympanum diameter (TYD 2.0); tympanum moderately distinct, oval, dorsally compressed, upper border concealed by supratympanic ridge; pupil oval in preservative, oriented vertically; nostril oval, oriented laterally, at slightly lower distance from the eye as to the snout tip (EN 1.6, NS 1.8); internarial distance subequal to upper eyelid width, and only slightly lower than distance between upper eyelids (IN 3.2, UEW 3.3, IUE 3.4); distance between posterior margins of eyes (IBE 8.6) about 1.7 times the distance between anterior margins of eyes (IFE 5.1); pineal ocellus not visible externally; vomerine ridges and teeth absent; maxillary teeth present; tongue large, rounded, with no medial lingual process. Forelimbs long, thin; forearm shorter than hand (FAL 7.6, HAL 9.0); fingers moderately long, thin, weakly developed lateral dermal fringes, relative finger lengths I < II < IV < III (FIL 2.8, FIIL 4.1, FIIIL 6.3, FIVL 4.3); interdigital webbing, and subarticular and supernumerary tubercles absent; thenar and outer metacarpal tubercles weakly developed; fingertips rounded, not raised ventrally, not expanded relative to digit widths, oval pads, terminal grooves absent. Hindlimbs long, thin; thigh shorter than shank and longer than foot (TL 14.5, SHL 15.9, FOL 13.4); distance from heel to tip of toe IV (TFOL 21.6); toes short and flattened with broad lateral dermal fringes that extend to tips of all digits, relative toe lengths I < II < V < III < IV; tips not dilated, with distinct pads raised ventrally; terminal grooves absent; toes with rudimentary webbing; outer metatarsal tubercle, subarticular and supernumerary tubercles absent; inner metatarsal tubercle present but barely distinguishable (figure 9).

*Skin texture of holotype*. Dorsal surfaces of head, body and limbs shagreened with weakly developed, scattered, small, smooth granules; flanks with small scattered tubercles; tympanum smooth, borders slightly raised; supratympanic fold well developed, extends from posterior corner of eye to above forearm insertions, narrow with slight widening posteriorly; weakly developed tubercle present on outer edge of upper eyelid with a tiny horn-like tubercle in the middle (especially in life); weakly developed inverted triangular ridge between eyes extends halfway over upper eyelids; short, thin, weakly developed, discontinuous dorsolateral dermal ridge extends from behind forearm to near groin on either side; weakly developed parietoscapular-sacral ridges form a vertically oriented ‘> <’ configuration, composed of rows of small tubercles, the two arms of the first inverted ‘V’-shaped ridge begin above the tympanum and extend posteriorly where they meet medially beyond the level of axilla, and the second inverted ‘V’-shaped ridge present at mid-dorsum; dorsal surfaces of forearms, thighs and shanks with several rows of weakly developed small tubercles arranged transversely. Ventral surfaces of body and limbs smooth; pectoral glands small, raised, rounded and positioned at level of axilla; belly with tubercles on each side arranged from below forearm insertion to near groin; femoral glands small, weakly developed, rounded (figure 8*b*,*m*).

*Colour of holotype*. *In life*. Dorsum, limbs and lateral surfaces of head light brown, lighter on limbs; flanks grey; the inverted triangular marking between eyes brown with light central blotch; granular ridges and granulations orangish red, especially on supratympanic folds; broad horizontal greyish-brown band extends from anterior margin of loreal region and over the entire tympanum with a break at the eye; obscure grey ‘X’-shaped marking on dorsum; dorsal surfaces of fore- and hindlimbs brown with faint dark brown transverse stripes on forearm; dorsal surfaces of thighs with a row of black spots; anterior and posterior surfaces of thighs mottled and blotched with blackish brown; iris reddish brown with golden tinge. Ventral surfaces of throat greyish brown, chest lighter than throat and belly greyish brown with faint irregular mottling; broad dark greyish-brown discontinuous band on either side of belly extends from forearm insertion to groin; fore- and hindlimbs greyish brown (figure 9). *In preservative*. Dorsum, limbs, lateral surfaces of head and flanks light brown; fore- and hindlimbs light straw-coloured (slightly lighter than dorsum); inverted triangular marking between eyes dark greyish brown; obscure light grey ‘X’-shaped marking on dorsum. Ventral surfaces of throat and chest light greyish brown; belly dark grey (supplementary material, figure S1).

**Morphological variation.** Morphometric variation among five types and one referred specimen (six adult males) is provided in table S5 in the supplementary material. Paratypes and referred specimen resemble the holotype in most morphological characters, with some exceptions. *Colour in life*: ZSI A 16615 (SDB 2024.6652)—dorsum dark brown; ZSI A 16612 (SDB 2024.6637)—posterior surfaces of thighs light brown, and throat and chest dark brown with irregular blotches. *Skin texture*: ZSI A 16613 (SDB 2024.6638)—dorsum relatively more granular, especially on ridges, and dorsal surfaces of thighs with row of black spots.

**Morphological comparison.** *Jingophrys siangensis* sp. nov. differs from all the currently recognized members of the subfamily Megophryinae by a combination of external morphological characters that includes a slender body, small adult male size (SVL 28.6–31.3 mm, *n =* 6), absence of vomerine teeth, rudimentary foot webbing and lateral fringes on toes.

*Jingophrys siangensis* sp. nov. cannot be confused with two megophryid species whose phylogenetic placement within their currently assigned genus (or subgenus) remains unknown. It can be distinguished by the following characters: from *Xenophrys damrei* by a much smaller adult male size, SVL 28.6–31.3 mm, *n =* 6 (versus larger, adult male SVL 47.7–57.1 mm, *n =* 7 [96,97]); and from *Ophryophryne pachyproctus* by the presence of maxillary teeth (versus absent) and a single horn-like tubercle on the outer margin of each upper eyelid (versus absent) [98].

Within the genus, *Jingophrys siangensis* sp. nov. differs from *J. feii* (based on [29]) by its larger adult male size, SVL 28.6–31.3 mm, *n =* 6 (versus smaller, SVL 24.3–25.1 mm, *n =* 4); snout nearly bluntly pointed in dorsal view (versus rounded); dorsal skin with small smooth granules (versus distinctly granular and with dense small tubercles); lateral sides of belly and flanks shagreened with small scattered tubercles (versus prominently tuberculate); and broad dark greyish-brown discontinuous bands on either side of belly from forearm insertion to groin (versus absent). *Jingophrys siangensis* sp. nov. differs from *Jingophrys hrdy* sp. nov. and *Jingophrys taniitatii* sp. nov. by its absence of prominent granulation and warts on dorsum (versus present); and specifically differs from *Jingophrys hrdy* sp. nov. by its toes rounded and with ventrally raised tips (versus fingers and toes ventrally flattened and without ventrally raised tips). *Jingophrys siangensis* sp. nov. differs from *J. pachyproctus* by its smaller adult male size, SVL 28.6–31.3 mm, *n =* 6 (versus larger, 33.0–37.0 mm, *n =* 5); vomerine teeth absent (versus present); and lateral fringes on toes (versus absent). *Jingophrys siangensis* sp. nov. differs from *Jingophrys tanogogo* sp. nov. by its upper eyelid width nearly equal or slightly shorter than distance between upper eyelids, UEW/IUE 0.84–1.05 (versus upper eyelid width much shorter than distance between upper eyelids, UEW/IUE 0.65–0.75); rudimentary webbing between toes (versus basal webbing); toes rounded and with ventrally raised tips (versus fingers and toes ventrally flattened and without raised tips). *Jingophrys siangensis* sp. nov. differs from *J. vegrandis* by its tympanum diameter being nearly equal or smaller than the distance between tympanum and eye, TYD/TYE 0.79–1.18 (versus wider, TYD/TYE 1.27–1.64); and finger and toe tips pale grey (versus orangish). *Jingophrys siangensis* sp. nov. differs from *Jingophrys wularsoni* sp. nov. by its smaller adult male size, SVL 28.6–31.3 mm, *n =* 6 (versus larger, SVL 36.4–40.8 mm, *n =* 9); internarial distance slightly narrower or nearly equal to horizontal diameter of eye, IN/EL 0.86–1.03 (versus wider than eye diameter, IN/EL 1.05–1.26); fingers and toes rounded and with ventrally raised tips (versus fingers and toes ventrally flattened and without ventrally raised tips); ventral surfaces of belly greyish white without prominent markings (versus greyish brown with blackish-brown blotches); lateral surfaces of belly and flanks shagreened with small scattered tubercles (versus prominently tuberculate, surrounded with blackish-brown spots); and lateral fringes on toes (versus absent). *Jingophrys siangensis* sp. nov. differs from *J. yeae* by its snout nearly bluntly pointed in dorsal view (versus rounded); upper margin of tympanum slightly concealed by supratympanic fold (versus upper one-third of tympanum concealed by supratympanic fold); tympanum nearly equal or smaller than the distance between tympanum and eye, TYD/TYE 0.79–1.18 (versus wider, TYD/TYE 1.21–1.46); upper eyelid width nearly equal or slightly shorter than distance between upper eyelids, UEW/IUE 0.84–1.05 (versus upper eyelid width much shorter than distance between upper eyelids, UEW/IUE 0.68–0.76); and lateral surfaces of belly and flanks shagreened with small scattered tubercles (versus prominently tuberculate, surrounded with blackish-brown spots). *Jingophrys siangensis* sp. nov. differs from *J. zhoui* (based on [31]) by its larger adult size, male SVL 28.6–31.3 mm, *n =* 6 (versus smaller, male SVL 23.0 mm, *n =* 1; female SVL 23.5–23.9 mm, *n =* 2); snout nearly bluntly pointed in dorsal view (versus near rounded); upper margin of tympanum slightly concealed by supratympanic fold (versus upper one-third of tympanum concealed by supratympanic fold); and dorsum light brown or greyish brown (versus dorsum and limbs orange-brown). *Jingophrys siangensis* sp. nov. differs from *J. zhaoermii* by its smaller adult male size, SVL 28.6–31.3 mm, *n =* 6 (versus larger, SVL 34.5–35.4 mm); snout bluntly pointed in dorsal view (versus rounded); vomerine ridge absent (versus weakly present); upper eyelid width nearly equal or slightly shorter than distance between upper eyelids, UEW/IUE 0.84–1.05 (versus upper eyelid width much shorter than distance between upper eyelids, UEW/IUE 0.64–0.70); and weak discontinuous parietoscapular-sacral ridges form a vertically oriented ‘><’ configuration on dorsum (versus strong and discontinuous). For additional comparisons, see the ‘Morphological comparisons’ sections for the respective species.

**Taxonomic note and additional comparison.** Another nomen, *Megalophrys kempii* Annandale, 1912, was described from ‘Upper Rotung’, a locality that is close to where we collected the type series of the new species, *Jingophrys siangensis* sp. nov. This taxon is currently known with certainty only from the original description [99] and the single type specimen (ZSIC 17013, HT), which is dehydrated (supplementary material, figure S2). It has previously been confused and variously assigned, including to Megophryidae [9,100–102], specifically with a history of being confused with *Xenophrys boettgeri* (see review by Mahony *et al.* [4], including references therein), as well as to rhacophorid genera [4,10,103–105], both of which have been doubted albeit with little evidence (e.g. [3,4,84]). Recently, this nomen was proposed to be a ceratobatrachid, *Liurana kempii*, based on multiple morphological traits differentiating it specifically from *Raorchestes* and *Philautus*, as well as from several megophryids [106]. We concur with this treatment based on examination of the holotype specimen and original description [99], as well as our additional new material of a *Liurana* (SDBDU 2022.6272) from Yembung, in the vicinity of the type locality of *Megalophrys kempii* Annandale, 1912. To avoid further confusion, we compare our new species with this nomen as reported in the original literature as well as its holotype (supplementary material, figures S1 and S2). We confirm that *Jingophrys siangensis* sp. nov. cannot be confused with *Megalophrys kempii* Annandale, 1912 (= *Liurana kempii* (Annandale, 1912)) based on key morphological differences such as its tympanum diameter nearly half of eye length, TYD/EL 0.44–0.56 (versus ‘nearly as large as eye’); upper border of tympanum concealed by supratympanic ridge (versus ‘fully exposed’); wide tympanum-to-eye distance, nearly as wide as tympanum diameter, TYE/TYD 0.85–1.27 (versus ‘close to which [eye] it is situated;’ very short tympanum-to-eye distance observed in the type specimen) (supplementary material, figure S2); nostril nearly as close to snout as eye, NS/EN 0.86–1.13 (versus ‘much nearer tip of snout than eye’); larger adult size, male SVL 28.6–31.3 mm (versus ‘15 mm’, suspected ‘sub-adult’ by Annandale [99], not confirmed owing to fragile condition of the type, but this length falls in the adult size range of *Liurana*, male SVL 11–28 mm); presence of dorsolateral dermal folds and supratympanic fold (versus ‘No dorso-lateral or supratympanic folds’); and dorsal skin shagreened with scattered granules, tubercles and dermal ridges, belly with tubercles on each side (versus ‘skin of back, limbs, throat and chest smooth, of belly obscurely granular’).

**Secondary sexual characters.** Males: nuptial pad or spines not visible; and a single subgular vocal sac while calling.

**Vocalization**. Calls of male *Jingophrys siangensis* sp. nov. were recorded at 20.00 hours on 23 August 2024 at Pangin, East Siang District, Arunachal Pradesh. Ambient temperatures were 29.2°C dry bulb and 26.2°C wet bulb. Calling individuals were found on leaves of shrubs adjacent to small roadside streams, with peak calling activity from 19.00 to 22.00 hours. Based on analyses of 50 calls from a single individual, the typical vocal repertoire of this species is as follows: males produce a single type of call with a pulsatile temporal structure; calls are not delivered in groups; the mean call duration is 59.7 ms; the amplitude envelope has a mean rise time of 21.5 ms and a mean fall time of 38.1 ms; calls deliver 10–15 pulses each at a mean rate of 193.66 pulses/s; and the call spectrum shows two broad peaks with a mean dominant frequency of 4.43 kHz (supplementary material, table S7; figure 10*a*–*d*).

**Call comparison.** Calls of *Jingophrys siangensis* sp. nov. can be differentiated from those of other congeners primarily by their frequency, pulses per call, pulse rate, duration and rise time: (i) *Call frequency*: *Jingophrys siangensis* sp. nov. calls differ from those of *Jingophrys hrdy* sp. nov., *Jingophrys taniitatii* sp. nov. and *Jingophrys wularsoni* sp. nov. by their higher dominant frequency, 4.43 kHz (versus lower, 3.23, 3.59 and 2.71 kHz, respectively), and from *Jingophrys tanogogo* sp. nov. and *J. vegrandis* calls by their lower dominant frequency, 4.43 kHz (versus higher, 4.88 and 5.46 kHz, respectively). The dominant frequency of *Jingophrys siangensis* sp. nov. calls, 4.31–4.57 kHz, falls within the range of those of *J.* cf. *yeae* and *J.* cf. *zhaoermii* (4.35–4.82 kHz and 4.31–4.78 kHz, respectively), with the mean dominant frequency, 4.43 kHz, slighter lower (4.61 and 4.57 kHz, respectively), but its calls can be differentiated from those of the other two species by other acoustic properties. (ii) *Pulses per call*: *Jingophrys siangensis* sp. nov. calls differ from those of *Jingophrys hrdy* sp. nov. by their fewer pulses per call, 10–15 (versus more, 22–27 pulses per call), and from *Jingophrys tanogogo* sp. nov. and *Jingophrys wularsoni* sp. nov. by their more pulses per call, 10–15 (versus lower, 5–6 and 7–9 pulses per call, respectively). *Jingophrys siangensis* sp. nov. calls have a similar number of pulses per call (10–15) as those of *Jingophrys taniitatii* sp. nov. (9–11 pulses per call), *J. vegrandis* (11–14 pulses per call), *J.* cf. *yeae* (11–13 pulses per call) and *J.* cf. *zhaoermii* (8–10 pulses per call), but they can be differentiated from those of all these other species by other acoustic properties. (iii) *Pulse rate*: *Jingophrys siangensis* sp. nov. calls have the fastest pulse rate among all studied congeners, 193.66 pulses/s (versus slower, 184.13 pulses/s in *Jingophrys hrdy* sp. nov., 127.48 pulses/s in *Jingophrys taniitatii* sp. nov., 140.98 pulses/s in *Jingophrys tanogogo* sp. nov., 99.46 pulses/s in *J. vegrandis*, 71.03 pulses/s in *Jingophrys wularsoni* sp. nov., 140.80 pulses/s in *J.* cf. *yeae* and 102.55 pulses/s in *J.* cf. *zhaoermii*). (iv) *Call duration*: *Jingophrys siangensis* sp. nov. calls differ from those of *Jingophrys taniitatii* sp. nov., *Jingophrys tanogogo* sp. nov. and *Jingophrys wularsoni* sp. nov. by their longer duration, 59.7 ms (versus shorter, 54.5, 31.1 and 51.7 ms, respectively), and from *Jingophrys hrdy* sp. nov., *J. vegrandis*, *J.* cf. *yeae* and *J.* cf. *zhaoermii* by their shorter duration (versus longer, 180.2, 166.6, 109.4 and 78.9 ms). (v) *Call rise time*: *Jingophrys siangensis* sp. nov. calls differ from those of *Jingophrys hrdy* sp. nov., *Jingophrys taniitatii* sp. nov., *J. vegrandis*, *J.* cf. *yeae* and *J.* cf. *zhaoermii* by their shorter call rise time (versus longer, 57.4, 23.3, 59.7, 39.2 and 31.6 ms, respectively), and from those of *Jingophrys tanogogo* sp. nov. and *Jingophrys wularsoni* sp. nov. by their longer call rise time (versus shorter, 14.7 and 15.6 ms, respectively) (figure 10; supplementary material, table S7).

Additionally, we compared vocalizations of *Jingophrys siangensis* sp. nov. with those published for three members in the People's Republic of China [29,31]. Calls of *Jingophrys siangensis* sp. nov. have a lower dominant frequency, 4.31–4.57 kHz, compared to those of *J. feii* and *J. yeae* (versus higher, 4.74–4.91 kHz and 4.4–5.2 kHz, respectively), and a higher dominant frequency, 4.31–4.57 kHz, in comparison to those of *J.* cf*. pachyproctus* (versus lower, 3.2–3.3 kHz). *Jingophrys siangensis* sp. nov. calls also differ from those of *J. feii*, *J.* cf*. pachyproctus* and *J. yeae* by their shorter duration, 59.7 ms (versus longer, 132.6, 746 and 139 ms, respectively), as well as by their fewer pulses per call (10–15) compared with *J. feii* and *J.* cf*. pachyproctus* (versus higher, 14–20 and 22–62 pulses per call, respectively), and relatively more pulses per call (10–15) in comparison to *J. yeae* (versus lower, 7–12 pulses per call) (supplementary material, table S7).

**Distribution.** *Jingophrys siangensis* sp. nov. is currently known only from three locations where the type series was collected: Kebang, Pangin and Yembung, East Siang District, Arunachal Pradesh State, India, at elevations between 304 and 315 m asl (figure 6; supplementary material, table S2).

**Habitat and natural history**. The habitat of *Jingophrys siangensis* sp. nov. is primary forest made accessible only owing to recent expansion of roads. The forest type is tropical wet evergreen with scattered canopy cover interspersed with hill streams and shallow wetlands. The specific sampling site at Kebang was a roadside hill stream almost completely covered with thick overhanging bushes. The area was less disturbed, apart from moderate vehicular noise during both day and night, and the animals called continuously regardless, perhaps owing to thick vegetation that prevented external light from penetrating the calling sites inside bushes. The site at Pangin was located on the outskirts of the town, and frog choruses were used to locate individuals on vegetation along a small hill stream running near and under a roadside culvert. All specimens were collected after dusk between late June and mid-August. Calling individuals perched on thick shrubs, bamboo thickets or leaves of small grasses immediately adjacent to shallow flowing water bodies. Calling activity was observed at all sampling sites during our surveys, with peak activity in August when animals called continuously. During the survey at Pangin in August 2024, up to 10–15 calling individuals were observed within short distances (10–20 cm) from each other along a small stretch of stream.

***Jingophrys tanogogo* sp. nov.**

(http://zoobank.org/urn:lsid:zoobank.org:act:484EC6A4-4AF6-47CF-9BFC-1217BB2B5ABF) figures 1–8 and 10–13; tables S1–S9 and figure S3 in the supplementary material

**Figure 11.**
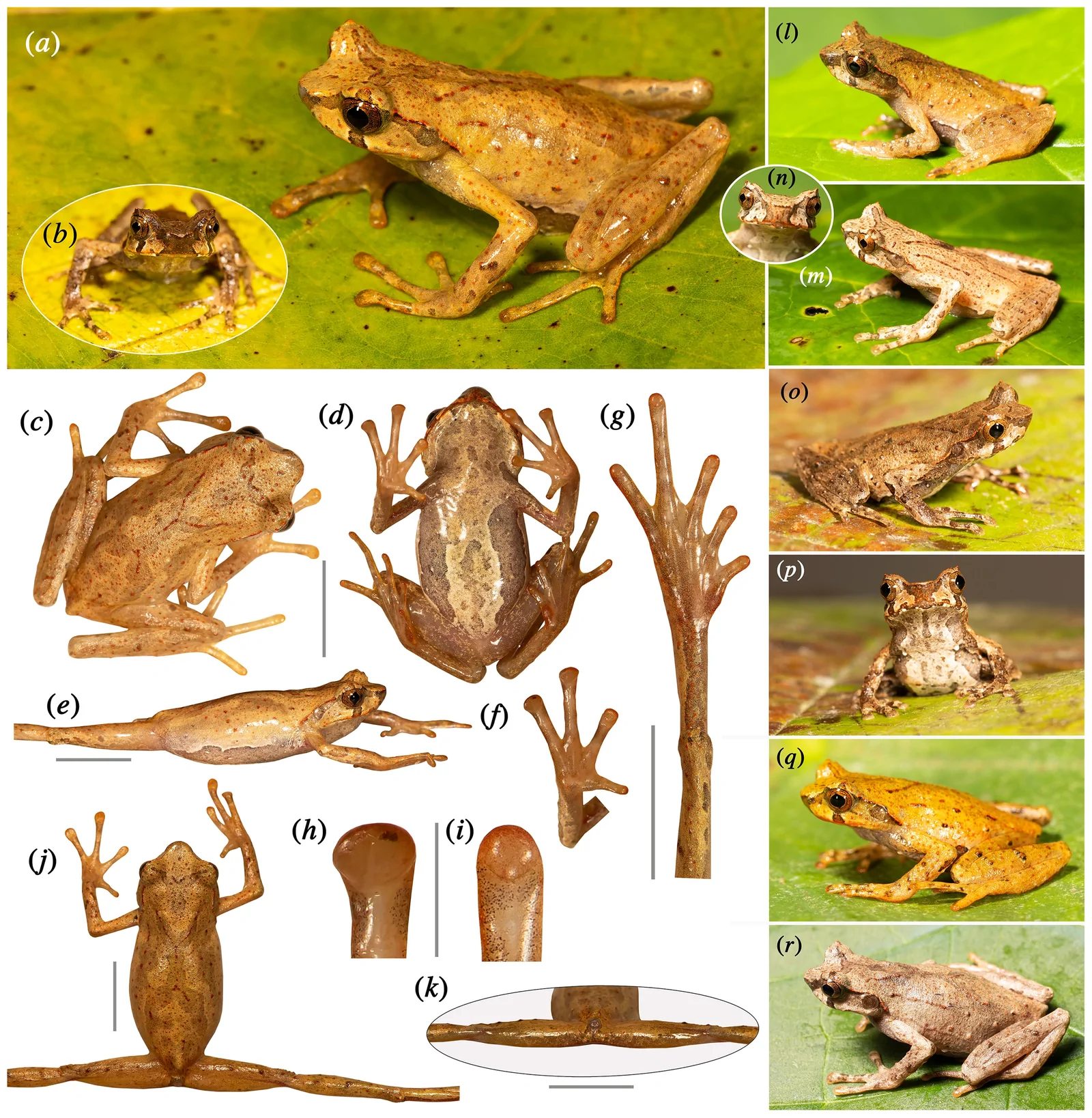
Types and referred specimens of *Jingophrys tanogogo* sp. nov., in life. (a*–k*) Holotype (ZSI A 16616, adult male): (*a*) dorsolateral view, (*b*) frontal view, (*c*) dorsal view, (*d*) ventral view, (e) lateral view of flank, groin and thigh, (*f*) ventral view of hand, (*g*) ventral view of foot, (*h*) enlarged view of third fingertip, (*i*) enlarged view of fourth toe tip, (*j*) dorsal view of thighs, shank and dorsum and (*k*) posterior view of thighs; (*l*) paratype (ZSI A 16622, male) in dorsolateral view; (*m*, *n*) referred specimen (SDBDU 2023.6530, female): (*m*) dorsolateral view and (*n*) frontal view; (*o*, *p*) referred specimen (SDBDU 2023.4483, male): (*o*) dorsolateral view and (*p*) frontal view; (*q*, *r*) dorsolateral views of two paratypes: (*q*) ZSI A 16617, a male and (*r*) ZSI A 16623, a female. Scale bars = 10 mm. Photographs: S. D. Biju and T. Tajo.

**Figure 12.**
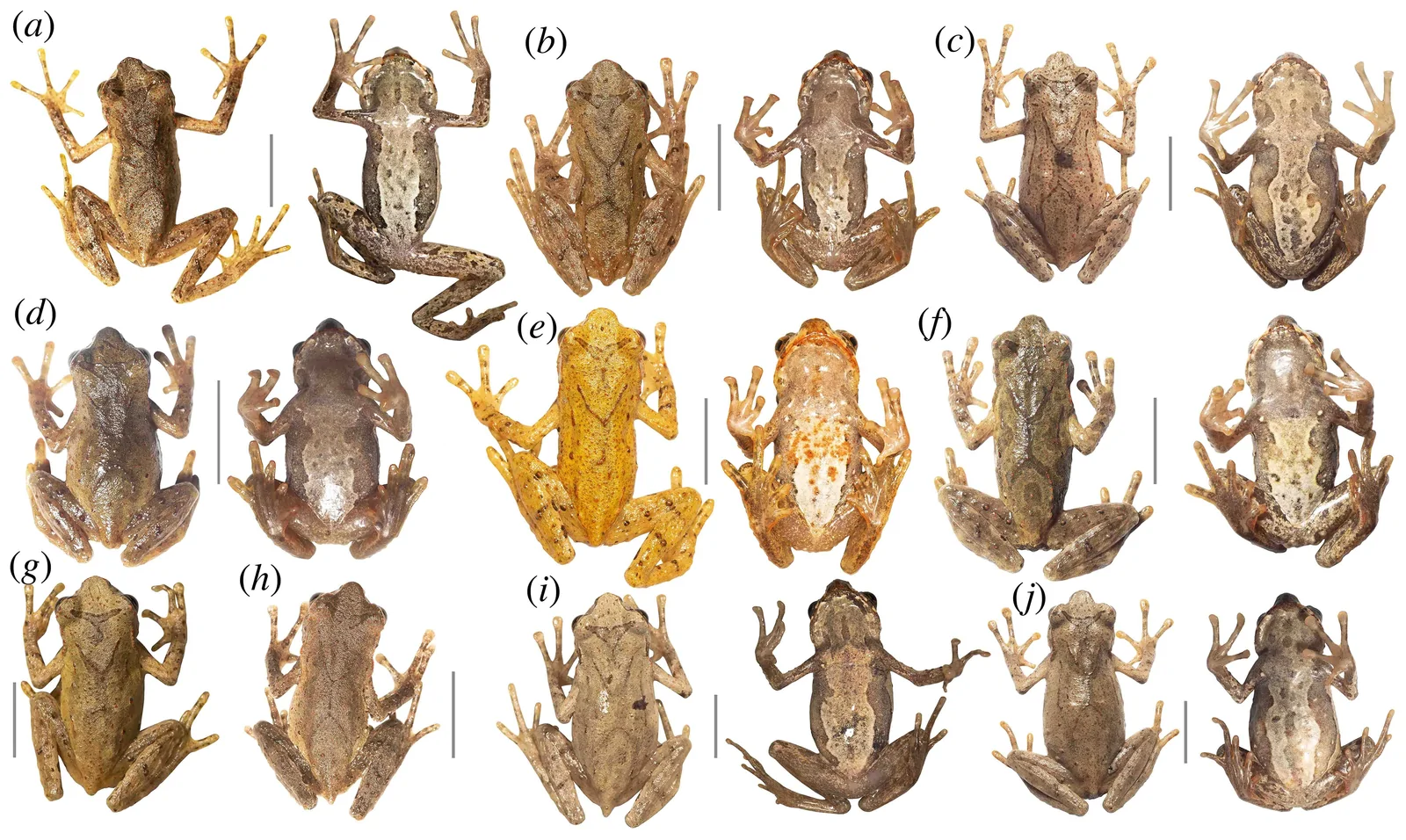
Morphological variation in skin colouration and markings among individuals of *Jingophrys tanogogo* sp. nov., in life. (*a–f*) Dorsal (left) and ventral (right) views: (*a*) paratype (ZSI A 16619, male), (*b*) paratype (ZSI A 16620, male), (*c*) referred specimen (SDBDU 2023.6530, female), (*d*) paratype (ZSI A 16622, male), (*e*) paratype (ZSI A 16617, male) and (*f*) referred specimen (SDBDU 2022.6026); (*g*, *h*) dorsal views of (*g*) referred specimen (SDBDU 2021.5109, female) and (*h*) adult male (not collected); (*i*, *j*) dorsal (left) and ventral (right) views of two female paratypes: (*i*) ZSI A 16621 and (*j*) ZSI A 16623. Scale bars = 10 mm. Photographs: S. D. Biju and T. Tajo.

**Figure 13.**
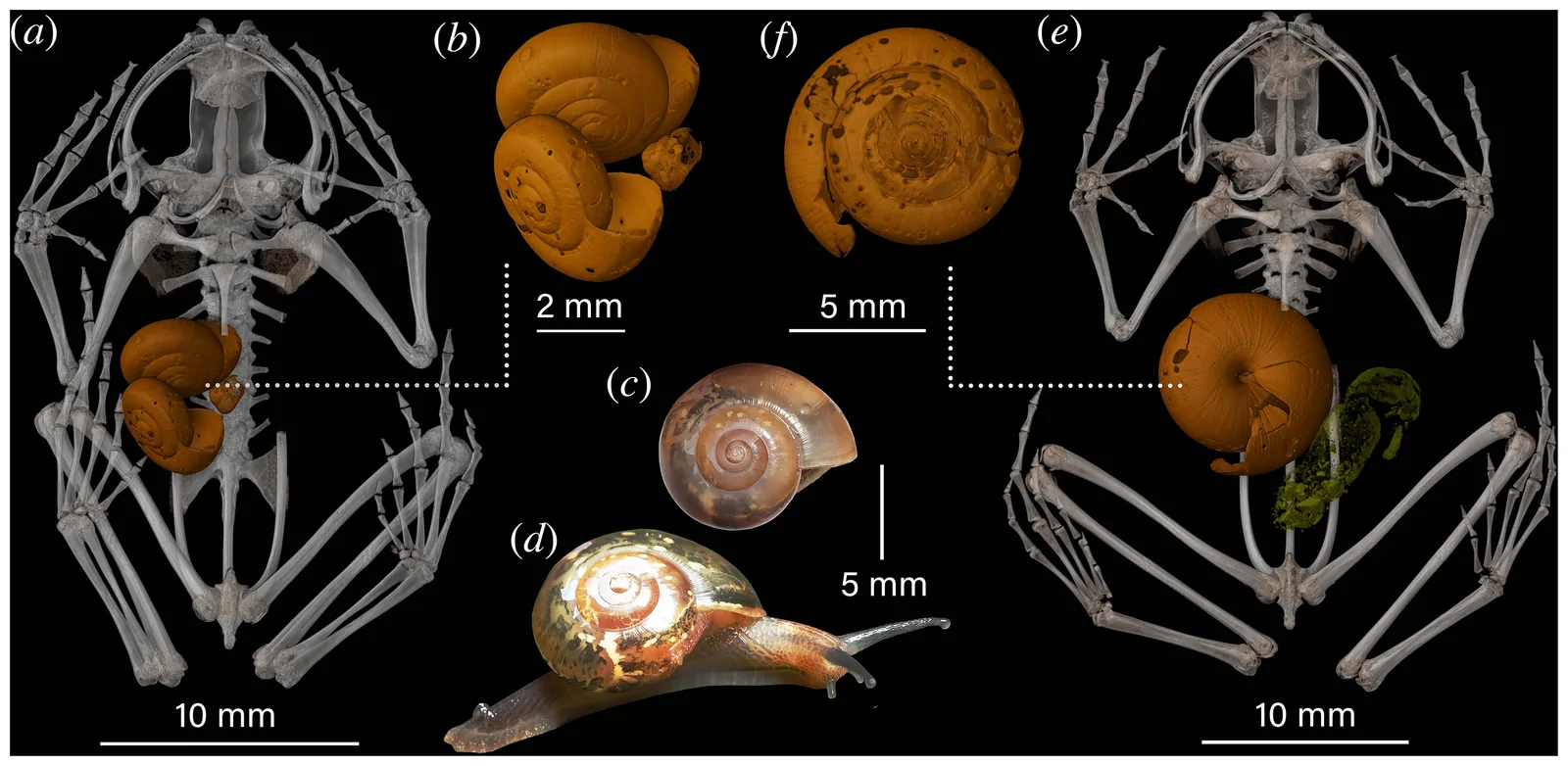
Snail-eating species of *Jingophrys* as revealed by stomach contents visualized through 3D microCT scans. (*a*, *b*) *Jingophrys tanogogo* sp. nov. (SDBDU 2021.5109, female): (*a*) entire skeleton in ventral view highlighting the presence of three snail shells (orange) inside the body, and (*b*) enlarged view of the snail shells, suspected to be *Macrochlamys* cf. *indica*; (*c*, *d*) freshly collected snail (*Macrochlamys* cf. *indica*, SDBDU 2024.6758) from the type locality of *Jingophrys tanogogo* sp. nov.: (*c*) dorsal view after preservation and (*d*) dorsolateral view in life; (*e*, *f*) *J. vegrandis* (SDB 2022.6290, male): (*e*) entire skeleton in ventral view highlighting the presence of a snail shell (orange) inside the body along with partially digested food (green) and (*f*) dorsal view of the snail shell, suspected to be *Macrochlamys* cf. *indica*.

**Holotype.** An adult male, ZSI A 16616 (SDB 2021.5009) from Hari Village (27.5999° N, 93.8857° E, 1729 m asl), Ziro Valley, Lower Subansiri District, Arunachal Pradesh State, India, collected by SDB, SG, RUK and TT on 9 October 2021.

**Paratypes.** An adult male, ZSI A 16617 (SDB 2022.6210), collected from the type locality by SDB, SG, RUK and TT on 5 August 2022; three adult males, ZSI A 16618–16619 (SDB 2021.5055–5056) and ZSI A 16620 (SDB 2022.6241), and an adult female, ZSI A 16621 (SDB 2022.5423), from Pange (27.5443° N, 93.8946° E, 1925 m asl), Tale Valley WLS, Lower Subansiri District, Arunachal Pradesh State, India, collected by SDB, SG, RUK and TT on 18 October 2021, 8 August 2022 and 2 June 2022, respectively; an adult male, ZSI A 16622 (SDB 2023.6529), and an adult female, ZSI A 16623 (SDB 2022.6022), from Tarin Tibe Khole Pass (27.5396° N, 93.8005° E, 1945 m asl), Tale Valley WLS, Lower Subansiri District, Arunachal Pradesh State, India, collected by SDB, SG, RUK and TT on 5 August 2023 and 16 June 2022, respectively.

**Referred specimens.** An adult male, SDBDU 2022.6226, and an adult female, SDBDU 2021.5109, from Pange (27.5443° N, 93.8946° E, 1925 m asl), Tale Valley WLS, Lower Subansiri District, Arunachal Pradesh State, India, collected by SDB, SG, RUK and TT on 2 June 2022, and 20 October 2021, respectively; an adult male, SDBDU 2023.4483, from Tajgi village (27.6402° N, 93.8638° E, 1740 m asl), Ziro Valley, Lower Subansiri District, Arunachal Pradesh State, India, collected by SDB, SG, RUK and TT on 24 June 2023; and an adult female, SDBDU 2023.6530, from Tarin Tibe Khole Pass (27.5396° N, 93.8005° E, 1945 m asl), Tale Valley WLS, Lower Subansiri District, Arunachal Pradesh State, India, collected by SDB, SG, RUK and TT on 5 August 2023.

**Etymology.** The specific name is taken from the word ‘tanogogo’ for ‘snails’ in the Apatani tribal language that is spoken in the region where the type series was collected. This new species feeds on terrestrial horntail snails (*Macrochlamys* cf. *indica*), as revealed through 3D micro-CT imaging (figure 13). The specific epithet *tanogogo* is treated as a noun in apposition to the genus name.

**Suggested common name.** Snail-eating horned frog.

**Diagnosis.** *Jingophrys tanogogo* sp. nov. cannot be confused with any other known congener based on a combination of characters that include its slender body; dorsal skin without prominent granulations; weakly developed lateral dermal fringes on fingers; well-developed lateral dermal fringes on all toes; basal webbing between toes; and ventral surfaces of chest and belly straw-coloured with a light brown ‘)(’ mark that extends over the forearms on either side.

**Phylogenetic relationships.** *Jingophrys tanogogo* sp. nov. is a member of the *J. vegrandis* species group and closely related to *J. vegrandis* (figures 1 and 2). Within its species group, *Jingophrys tanogogo* sp. nov. shows the following DNA sequence divergence in the mitochondrial 16S rRNA gene: 8.7–8.9% from *J. feii*; 7.3–7.5% from *Jingophrys siangensis* sp. nov.; 4.1–4.6% from *J. vegrandis*; 5.3–6.5% from *J. yeae* sensu lato; 4.5–4.7% from *J. zhaoermii* sensu lato; 5.9–6.1% from *J. zhoui*; and 6.9–7.1% from the undescribed candidate species *J*. cand. sp. A. (supplementary material, table S3).

**Holotype description (measurements in mm).** Adult male (SVL 30.3), slender body; head moderately large, as long as wide (HL 10.4, HW 10.4, MN 8.6; MFE 6.6; MBE 4.1); snout nearly bluntly pointed in dorsal and ventral views, strongly acutely protruding in lateral view, snout tip projecting far beyond lower lip (SP 2.2); loreal region acute, concave; canthus rostralis sharp (angled); dorsal snout slightly concave; eye larger than tympanum diameter but smaller than snout length (EL 2.9, TYD 1.8, SL 3.9); tympanum-to-eye distance (TYE 1.9) nearly equal to tympanum diameter (TYD 1.8); tympanum moderately distinct, oval, dorsally compressed, upper border not concealed by supratympanic ridge; pupil oval in preservative, oriented vertically; nostril oval, oriented laterally, closer to snout than to eye (EN 2.0, NS 1.6); internarial distance greater than upper eyelid width, and shorter than narrowest distance between upper eyelids (IN 3.1, UEW 2.5, IUE 3.6); distance between posterior margins of eyes (IBE 9.3) nearly twice the distance between anterior margins of eyes (IFE 4.8); pineal ocellus not visible externally; vomerine ridges and teeth absent; maxillary teeth present; tongue large, rounded, with no medial lingual process. Forelimbs long, thin; forearm shorter than hand (FAL 7.8, HAL 10.1); fingers moderately long, thin, weakly developed lateral dermal fringes, relative finger lengths I < II < IV < III (FIL 2.7, FIIL 4.8, FIIIL 6.5, FIVL 5.0); interdigital webbing, and subarticular and supernumerary tubercles absent; thenar and outer metacarpal tubercles weakly developed; fingertips rounded, not raised ventrally, not expanded relative to digit widths, oval pads, terminal grooves absent. Hindlimbs long, thin; thigh shorter than shank, and longer than foot (TL 14.6, SHL 15.7, FOL 12.6); distance from heel to tip of toe IV (TFOL 21.7); toes short and flattened with broad lateral fringes that extend to tips of all digits, relative toe lengths I < II < V < III < IV; tips not dilated, with distinct pads not raised ventrally; terminal grooves absent; toes with basal webbing; outer metatarsal tubercle, subarticular and supernumerary tubercles absent; inner metatarsal tubercle present but barely distinguishable (figure 11).

*Skin texture of holotype*. Dorsal surfaces of head, body, flanks and limbs shagreened with densely scattered small and smooth granules, relatively less prominent on limbs and lateral surfaces of head; tympanum smooth, borders slightly raised; supratympanic fold well developed, extends from posterior corner of eye to above forearm insertion, narrow without widening posteriorly; prominent tubercles present on outer edge of upper eyelid with a tiny horn-like tubercle in the middle; inverted triangular ridge between eyes extends halfway over upper eyelids; short, thin and discontinuous dorsolateral dermal ridge extends from behind forearm to near groin on either side; weakly developed parietoscapular-sacral ridges form a vertically oriented ‘> <’ configuration, composed of rows of small tubercles, the two arms of the first inverted ‘V’-shaped ridge begin above the tympanum and extend posteriorly where they meet medially beyond the level of axilla, and the second inverted ‘V’-shaped ridge present at mid-dorsum; dorsal surfaces of forearms, thighs and shanks with several rows of small tubercles arranged transversely. Ventral surfaces of body and limbs smooth; pectoral glands small, raised, rounded and positioned at level of axilla; belly with tubercles on each side arranged from below forearm insertion to near groin; femoral glands small, rounded, weakly developed, slightly raised, closer to outer edge of knee than to cloaca; prominent tubercles surrounding the cloacal region (figure 8*c*,*n*).

*Colour of holotype*. *In life*. Dorsum, limbs, lateral surfaces of head, and flanks light yellowish brown, lighter on lateral surfaces of belly; inverted triangular marking between eyes light brown with light central blotch; roughly ‘X’-shaped light brown blotch present on dorsum; granular ridges and granulations orangish red, including margins of supratympanic folds; broad horizontal greyish-brown band extends from anterior margin of loreal region and over entire tympanum with a break at the eye; another vertical band extends from margins of upper eyelid to margins of upper lips with a break at the eye, forming roughly a ‘+’ mark on lateral surfaces of head with eye as the centre point; anterior snout mottled with dark brown; iris reddish brown with golden tinge. Ventral surfaces of throat, chest and belly straw-coloured with irregular faint mottling; broad dark grey band on either side of belly extends from forearm insertion to groin making the straw-coloured central belly narrower; occasionally a light brown ‘)(’ mark on lower half of throat that extends over the forearms on either side; fore- and hindlimbs greyish brown, thighs with light yellowish-brown marbling; dark greyish-brown colouration surrounding cloacal opening; tips of fingers and toes and toe margins reddish orange (figure 11). *In preservative*. Dorsum, limbs, lateral surfaces of head and flanks straw-coloured; inverted triangular marking between eyes light greyish brown with light grey central blotch; light grey ‘X’-shaped marking on dorsum; upper eyelids dark grey; light brown ‘+’ mark on lateral surface of head with eye as the centre point; anterior snout light brown. Ventral surfaces of throat and chest light straw-coloured; belly greyish white; broad grey band on either side of belly extends from forearm insertion to groin; fore- and hindlimbs light straw-coloured; light grey colouration surrounds cloacal opening (supplementary material, figure S3).

**Morphological variation.** Morphometric variation among eight types and four referred specimens (eight adult males and four adult females) is provided in table S5 in the supplementary material. Paratypes and referred specimens resemble the holotype in most morphological characters, with some exceptions. *Colour in life*: ZSI A 16617 (SDB 2022.6210)—dorsum prominently yellowish brown, ventral surfaces with a few scattered reddish-orange blotches at mid-belly, and lower and upper margins of lips with more prominent reddish-orange colouration; SDBDU 2023.4483—dorsum light brown; ZSI A 16623 (SDB 2022.6022) and ZSI A 16619 (SDB 2021.5056)—dorsum dark grey. *Skin texture*: Females [ZSI A 16623 (SDB 2022.6022) and ZSI A 16621 (SDB 2022.5423)] possess fewer dermal asperities on dorsal skin compared to males; ZSI A 16620 (SDB 2022.6241), ZSI A 16623 (SDB 2022.6022), SDBDU 2022.6226 and SDBDU 2023.4483—the vertically oriented ‘>-<’ configuration is faintly raised; ZSI A 16617 (SDB 2022.6210) and SDBDU 2021.5109 possess only the ‘V’-shaped parietoscapular ridges; ZSI A 16619 (SDB 2021.5056), ZSI A 16620 (SDB 2022.6241), ZSI A 16617 (SDB 2022.6210) and SDBDU 2021.5109 have prominent longitudinal granular tubercles on limbs (more prominent on the hindlimbs) that correspond with blackish-brown crossbands, and fingers and toes with more prominent crossbands; ZSI A 16619 (SDB 2021.5056), ZSI A 16620 (SDB 2022.6241), ZSI A 16617 (SDB 2022.6210) and SDBDU 2021.5109—prominent crossbands on limbs (more prominent on hindlimbs) and fingers and toes. *Dermal extensions:* ZSI A 16620 (SDB 2022.6241)—extensive broad lateral fringes on all toes; ZSI A 16620 (SDB 2022.6241), ZSI A 16621 (SDB 2022.5423) and SDBDU 2023.4483—protuberance present on vent and extends beyond cloaca (figures 11 and 12).

**Morphological comparison.** *Jingophrys tanogogo* sp. nov. differs from all currently recognized members of the subfamily Megophryinae by a combination of external morphological characters that include a slender body, smaller adult male size (SVL 24.9–30.3 mm, *n =* 8), absence of vomerine teeth, basal webbing between toes, well-developed lateral dermal fringes on all toes and ventral surfaces immaculate with two broad dark grey bands extending on either side of the belly from forearm insertion to groin.

*Jingophrys tanogogo* sp. nov. cannot be confused with two megophryid species whose phylogenetic placement within their currently assigned genus (or subgenus) remains unknown. It can be distinguished by the following characters: from *Xenophrys damrei* by its much smaller adult male size, SVL 24.9–30.3 mm, *n =* 8 (versus larger, adult male SVL 47.7–57.1 mm, *n =* 7 [96,97]); and from *Ophryophryne pachyproctus* by the presence of maxillary teeth (versus absent) and a single horn-like tubercle on outer margin of each upper eyelid (versus absent) [98].

Within the genus, *Jingophrys tanogogo* sp. nov. differs from all currently recognized species (including six previously known and five newly described) by basal foot webbing (versus absent or rudimentary); weakly developed lateral dermal fringes on fingers (versus absent); and ventral skin immaculate with two continuous broad dark grey bands extending on either side of the belly from forearm insertion to groin (versus partial or complete maculation, and presence or absence of broad discontinuous bands on lateral sides of the belly). It also differs from *Jingophrys hrdy* sp. nov. and *Jingophrys taniitatii* sp. nov. by its absence of prominent granulation and warts on dorsum (versus present); and toes ventrally flattened and without ventrally raised tips (versus rounded and with ventrally raised tips). Further, it differs from *J. feii* (based on [29]) by its snout nearly bluntly pointed in dorsal view (versus rounded); upper eyelid width narrower than the distance between upper eyelids, UEW/IUE 0.65–0.75 (versus nearly equal, UEW/IUE 0.94–1.03); toes ventrally flattened and without ventrally raised tips (versus rounded and with ventrally raised tips); dorsal skin with small smooth granules (versus distinctly granular and with dense small tubercles); lateral sides of the belly and flanks shagreened with small scattered tubercles (versus prominently tuberculate); and broad dark grey bands on either side of belly from forearm insertion to groin (versus absent). *Jingophrys tanogogo* sp. nov. differs from *J. pachyproctus* by its smaller adult male size, SVL 24.9–30.3 mm, *n =* 8 (versus larger, SVL 33.0–37.0 mm, *n =* 5); vomerine teeth absent (versus present); and well-developed lateral fringes on toes (versus absent). *Jingophrys tanogogo* sp. nov. differs from *Jingophrys siangensis* sp. nov. by its upper eyelid width much shorter than the distance between eyelids, UEW/IUE 0.65–0.75 (versus slightly shorter or nearly equal, UEW/IUE 0.84–1.05); basal webbing between toes (versus rudimentary webbing); and toes ventrally flattened and without raised tips (versus toes rounded and with ventrally raised tips). *Jingophrys tanogogo* sp. nov. differs from *J. vegrandis* by shank being nearly equal or longer than thigh in length, SHL/TL 1–1.08 (versus shank relatively longer than thigh, SHL/TL 1.05–1.12); toes ventrally flattened and without ventrally raised tips (versus rounded and with ventrally raised tips); belly greyish white without prominent markings (versus greyish brown with light to dark brown blotches); ventral surfaces of thighs and entire hindlimbs immaculate (versus ventral surfaces of anterior and posterior parts of thighs, and dorsal surface of shank and foot mottled and blotched with blackish brown); and lateral side of belly and flanks shagreened with small scattered tubercles (versus prominently tuberculate, surrounded with blackish-brown spots). *Jingophrys tanogogo* sp. nov. differs from *Jingophrys wularsoni* sp. nov. by its smaller adult male size, SVL 24.9–30.3 mm, *n =* 8 (versus larger, SVL 36.4–40.8 mm, *n =* 9); internarial distance nearly equal or slightly shorter than horizontal diameter of eye, IN/EL 0.83–1.07 (versus wider than eye diameter, IN/EL 1.05–1.26); toes ventrally flattened and without ventrally raised tips (versus rounded and with ventrally raised tips); belly greyish white without prominent markings (versus greyish brown with blackish-brown blotches); ventral surfaces of thighs and entire hindlimbs immaculate (versus ventral surfaces of anterior and posterior parts of thigh mottled and blotched with blackish brown); lateral side of belly and flanks shagreened with small scattered tubercles (versus prominently tuberculate, surrounded with blackish-brown spots); and well-developed lateral fringes on toes (versus absent). *Jingophrys tanogogo* sp. nov. differs from *J. yeae* by its snout nearly bluntly pointed in dorsal view (versus rounded); shank nearly equal or longer than thigh in length, SHL/TL 1–1.08 (versus shank relatively longer than thigh, SHL/TL 1.02–1.13); toes ventrally flattened and without ventrally raised tips (versus rounded and with ventrally raised tips); lateral sides of belly and flanks shagreened with small scattered tubercles (versus prominently tuberculate, surrounded with blackish-brown spots); and well-developed lateral fringes on toes (versus weakly developed). *Jingophrys tanogogo* sp. nov. differs from *J. zhaoermii* by its snout nearly bluntly pointed in dorsal view (versus rounded); head nearly as long as wide, HL/HW 0.99–1.01 (versus head longer than wide, HL/HW 1.14–1.19); and shank nearly equal or longer than thigh in length, SHL/TL 1–1.08 (versus shank relatively longer than thigh, SHL/TL 1.07–1.12). *Jingophrys tanogogo* sp. nov. differs from *J. zhoui* (based on [31]) by its larger adult size, male SVL 24.9–30.3 mm, *n =* 8, female SVL 31.8–35.5 mm, *n =* 4 (versus smaller, male SVL 23.0 mm, *n =* 1, female SVL 23.5–23.9 mm, *n =* 2); snout nearly bluntly pointed in dorsal view (versus near rounded); dorsum light yellowish brown or greyish brown (versus dorsum and limbs orange-brown); and well-developed lateral fringes on toes (versus absent or weakly developed). For additional comparisons, see the ‘Morphological comparisons’ sections for the respective species.

**Secondary sexual characters.** Males: nuptial pad or spines not visible; and a single subgular vocal sac while calling. Females: both specimens contained moderately large (diameter 2.9 ± 0.3 mm, *n =* 20), creamy white (pigmentless) ova.

**Vocalization** **.** Calls of male *Jingophrys tanogogo* sp. nov. were recorded at 20.00 hours on 6 August 2023 at Pange in Tale Valley WLS, Lower Subansiri District, Arunachal Pradesh. Ambient temperatures were 19.5°C dry bulb and 18.2°C wet bulb. Frogs called from leaves of shrubs adjacent to small streams, with peak calling from 17.30 to 23.00 hours as well as erratic calls from one or two individuals during the day. Based on analyses of 50 calls from a single individual, the typical vocal repertoire of this species is as follows: males produce a single type of call that has a pulsatile temporal structure; calls are not delivered in groups; the mean call duration is 31.1 ms; the amplitude envelope has a mean rise time of 14.7 ms and a mean fall time of 16.5 ms; calls deliver 5–6 pulses at a mean rate of 140.98 pulses/s; and the call spectrum shows two broad peaks with a mean dominant frequency of 4.88 kHz (figure 10*e*–*h*; supplementary material, table S7).

**Call comparison.** Calls of *Jingophrys tanogogo* sp. nov. can be differentiated from those of other congeners primarily by their frequency, pulse rate, duration and rise time, as follows: (i) *call frequency*: *Jingophrys tanogogo* sp. nov. calls differ from those of *Jingophrys hrdy* sp. nov., *Jingophrys siangensis* sp. nov., *Jingophrys taniitatii* sp. nov., *Jingophrys wularsoni* sp. nov., *J.* cf. *yeae* and *J.* cf. *zhaoermii*, by their higher dominant frequency, 4.88 kHz (versus lower, 3.23, 4.43, 3.59, 2.7, 4.61 and 4.57 kHz, respectively), and from those of *J. vegrandis* by their lower dominant frequency, 4.88 kHz (versus higher, 5.46 kHz). The dominant frequencies of *Jingophrys tanogogo* sp. nov. and *J.* cf. *yeae* fall within the same range. However, *Jingophrys tanogogo* sp. nov. differs by its faster pulse rate, 140.98 pulses/s (versus slower, 102.55 pulses/s), fewer pulses per call, 5–6 pulses per call (versus more, 11–13 pulses per call), and shorter call duration, 31.1 ms (versus longer, 109.4 ms). (ii) *Pulse rate*: *Jingophrys tanogogo* sp. nov. calls differ from those of *Jingophrys hrdy* sp. nov., *J. vegrandis*, *Jingophrys wularsoni* sp. nov., *J.* cf. *yeae* and *J.* cf. *zhaoermii*, by their faster pulse rate, 140.98 pulses/s (versus slower, 127.48, 71.03, 140.8, 102.55 and 99.46 pulses/s, respectively). The pulse rate of *Jingophrys tanogogo* sp. nov. (140.98 pulses/s) almost overlaps with that of *Jingophrys wularsoni* sp. nov. (140.8 pulses/s). However, *Jingophrys tanogogo* sp. nov. calls differ by their higher dominant frequency, 4.88 kHz (versus lower, 2.7 kHz). *Jingophrys tanogogo* sp. nov. calls differ from those of *Jingophrys siangensis* sp. nov. and *Jingophrys taniitatii* sp. nov. by their slower pulse rate, 140.98 pulses/s (versus faster, 193.66 and 184.13 pulses/s, respectively). (iii) *Call duration*: *Jingophrys tanogogo* sp. nov. calls have the shortest duration (31.1 ms) among all studied congeners (versus longer, 180.2 ms in *Jingophrys hrdy* sp. nov., 59.7 ms in *Jingophrys siangensis* sp. nov., 54.5 ms in *Jingophrys taniitatii* sp. nov., 51.7 ms in *Jingophrys wularsoni* sp. nov., 166.6 ms in *J. vegrandis*, 109.4 ms in *J.* cf. *yeae* and 78.9 ms in *J.* cf. *zhaoermii*). (iv) *Call rise time*: *Jingophrys tanogogo* sp. nov. calls also have the shortest rise time (14.7 ms) among all studied congeners (versus longer, 57.4 ms in *Jingophrys hrdy* sp. nov., 23.3 ms in *Jingophrys taniitatii* sp. nov., 15.6 ms in *Jingophrys wularsoni* sp. nov., 59.7 ms in *J. vegrandis*, 39.2 ms in *J.* cf. *yeae* and 31.6 ms in *J.* cf. *zhaoermii*) (figure 10; supplementary material, table S7).

Additionally, we compared vocalizations of *Jingophrys tanogogo* sp. nov. from our study with published reports for three members in the People's Republic of China [29,31]. Calls of *Jingophrys tanogogo* sp. nov. have a similar dominant frequency (4.78–5.08 kHz) as those of *J. feii* (4.74–4.91 kHz) and *J. yeae* (4.4–5.2 kHz), but a higher dominant frequency (4.78–5.08 kHz) than the calls of *J.* cf. *pachyproctus* (versus lower, 3.2–3.3 kHz). *Jingophrys tanogogo* sp. nov. calls also differ from those of *J. feii*, *J.* cf. *pachyproctus* and *J. yeae* by their shorter caller call duration, 31.1 ms (versus longer, 132.6, 746 and 139 ms, respectively) and fewer pulses per call, 5–6 pulses per call (versus more, 14–20, 22–62 and 7–12 pulses per call, respectively) (supplementary material, table S7).

**Distribution.** *Jingophrys tanogogo* sp. nov. is currently known only from four sites within the Tale Valley WLS (Pange and Tarin Tibe Khole Pass) and Ziro Valley (Hari Village and Tajgi Village) regions in the Lower Subansiri District, Arunachal Pradesh State, India, at elevations from 1729 to 1945 m asl (figure 6; supplementary material, table S2).

**Habitat and natural history** **.** The habitat of *Jingophrys tanogogo* sp. nov. is primary forest made accessible only owing to recent expansion of roads. The forest type is tropical wet evergreen with scattered canopy cover interspersed with hill streams and shallow wetlands. The type locality at Hari Village is a primary forest patch with a canopy-covered hill stream. Individuals were collected from vegetation on the edges of a fast-flowing stretch with rapids near a waterfall. The sampling location at Tajgi Village is an excavated mountain site remaining from construction of a National Highway (NH 13). The specific collection site is a small and steep hill stream close to a large waterfall. Areas adjacent to the highway are disturbed with human activities like garbage dumping and bonfires, but upstream forest stretches remain pristine. At this location, no calls were heard, and few adults were collected through opportunistic searches. At Pange in Tale Valley WLS, up to 8–10 calling individuals were often observed within short distances (10–20 cm) from each other along stretches of a single mountain stream during July 2023. All specimens were collected after dusk between late June and mid-August. Individuals were perched on thick shrubs, bamboo thickets or leaves of small grasses immediately adjacent to small streams. During peak calling, the animals called continuously and were not disturbed by the flashlights. During our surveys, *Jingophrys tanogogo* sp. nov. was found sympatrically with *Jingophrys wularsoni* sp. nov. at Pange; with both *Jingophrys hrdy* sp. nov. and *Jingophrys wularsoni* sp. nov. at Tarin Tibe Khole Pass, and with *Jingophrys taniitatii* sp. nov. at Tajgi.

We found evidence for snail eating by *Jingophrys tanogogo* sp. nov. through 3D micro-CT imaging. Three specimens (two medium-sized and one small) of the Horntail or Indian land snails were revealed inside the gut of a single specimen (SDBDU 2021.5109, female). Fresh collections of snails from the specific collection site confirm their species identity as *Macrochlamys* cf. *indica* of the family Ariophantidae (figure 13).

***Jingophrys vegrandis* (Mahony, Teeling and Biju, 2013)**

Figures 1–8, 10 and 13–15; tables S1–S9 in the supplementary material

**Figure 14.**
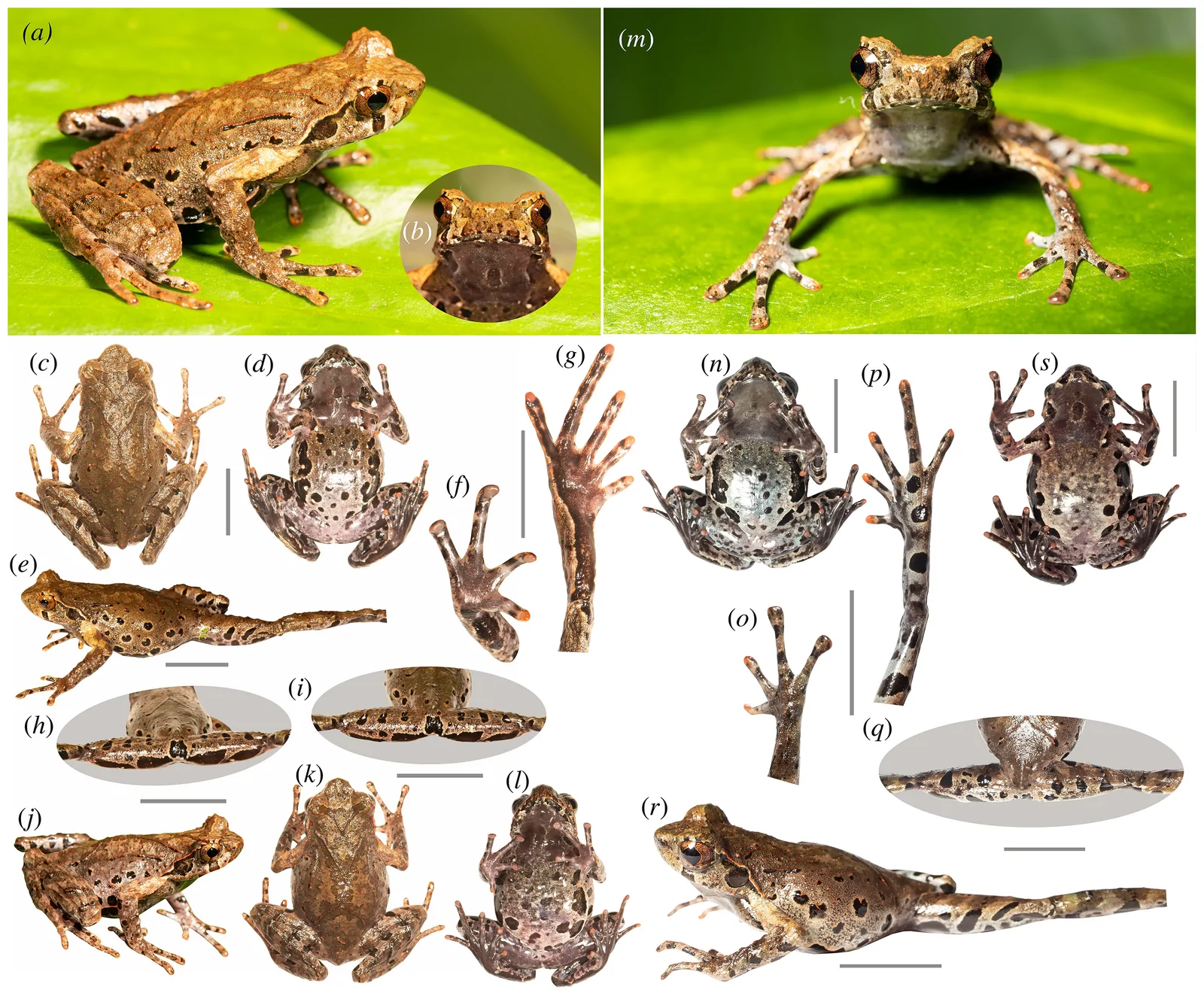
Adult male topotypes of *Jingophrys vegrandis*, in life. (*a–i*) SDBDU 2022.6289: (*a*) dorsolateral view, (*b*) frontal view, (*c*) dorsal view, (*d*) ventral view, (*e*) lateral view of flank, groin and thigh, (*f*) ventral view of hand, (*g*) ventral view of foot, (*h*) posterior view of thighs and (*i*) dorsal view of thighs; (*j–l*) SDBDU 2022.6290: (*j*) dorsolateral view, (*k*) dorsal view and (*l*) ventral view; (*m–r*) SDBDU 2023.6521: (*m*) frontal view, (*n*) ventral view, (*o*) dorsal view of hand, (*p*) dorsal view of foot, (*q*) dorsal view of thighs and (*r*) lateral view of flank, groin and thigh; (*s*) SDBDU 2023.6522, ventral view. Scale bars = 10 mm. Photographs: S. D. Biju and T. Tajo.

**Figure 15.**
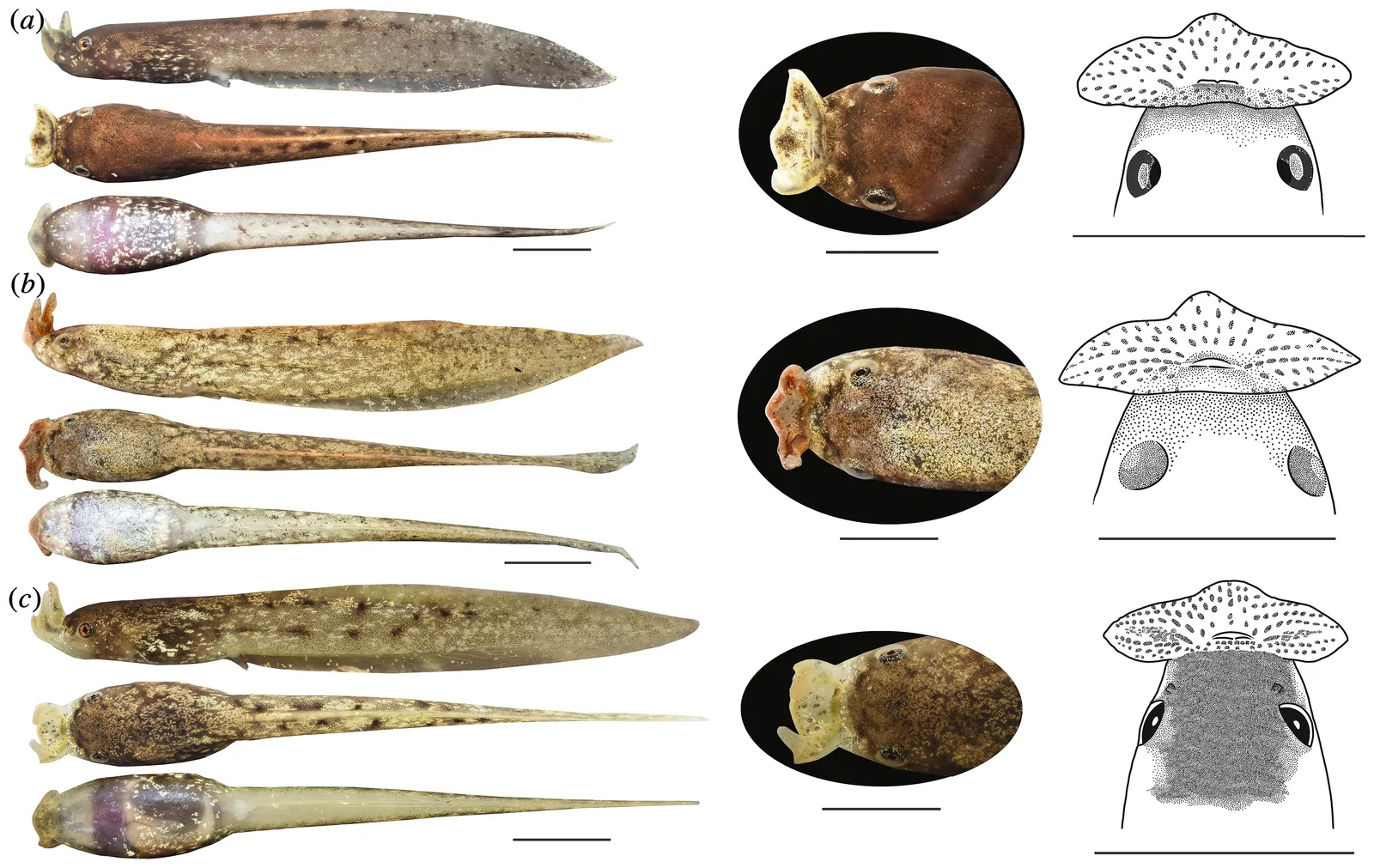
Tadpoles of three *Jingophrys* species from India, in life. *(a) Jingophrys vegrandis* (SDBDU 2024.6625, Gosner stage 26), *(b) J. yeae* (SDBDU 2023.6512, Gosner stage 28) and *(c) Jingophrys hrdy* sp. nov. (SDBDU 2023.6535, Gosner stage 35). Images of each species are arranged as follows: left panel, from top to bottom—whole specimens in lateral, dorsal and ventral views; right panel, from left to right—enlarged view of head in dorsal view, and schematic illustration of oral disc. Scale bars = 5 mm. Photographs and illustrations: S. D. Biju and T. Tajo.

**Original name and description.** *Megophrys vegrandis* Mahony, Teeling and Biju, 2013. In: Mahony, S., E. C. Teeling and S. D. Biju. 2013. Three new species of horned frogs, *Megophrys* (Amphibia: Megophryidae), from Northeast India, with a resolution to the identity of *Megophrys boettgeri* populations reported from the region. Zootaxa, 3722: 143–169, p. 158. **Holotype.** By original designation, ZSI A 11605, an adult male collected by Systematics Lab members on 8 August 2009. **Paratypes.** ZSI A 11604 and BNHS 5597, two adult males collected with the holotype. **Type locality.** Sessa Village (27°06.067′ N 92°31.642′ E, 1110 m asl), from a tributary of the Sessa Nadi (river), Bhalukpong Forest Division, West Kameng District, Arunachal Pradesh State, India. **Current status of specific name.** Valid name, as *Jingophrys vegrandis* (Mahony, Teeling and Biju, 2013).

**Taxonomic treatment.** The species was originally described as *Megophrys vegrandis* [4] and subsequently transferred to *Xenophrys vegrandis* [5]. It was referred under a subgeneric classification scheme as *Megophrys* (*Xenophrys*) *vegrandis* [12] and is currently treated as *Jingophrys vegrandis* under a generic-level classification scheme [15].

**Referred specimens.** An adult male, ZSI A 11605, holotype; an adult male, SDBDU 2009.1274, collected with the holotype; and four adult males, SDBDU 2022.6289, SDBDU 2022.6290 and SDBDU 2023.6521–6522, from Sessa Village (27.0992° N, 92.5242° E, 1210 m asl), Bhalukpong Forest Division, West Kameng District, Arunachal Pradesh State, India, collected by SDB, SG, RUK and TT on 15 August 2022 and 2 July 2023, respectively.

**Etymology.** The specific name is derived from the Latin word ‘*vegrandis*’, meaning ‘diminutive’ or ‘tiny’ in English, referring to the small adult male size of this species [4].

**Suggested common name.** Sessa horned frog.

**Diagnosis.** This species can be distinguished from known congeners by the following combination of characters: small adult size (SVL 26.8–29.9 mm); supratympanic fold narrow anteriorly and does not widen posteriorly; foot webbing rudimentary; lateral dermal fringes on toes moderately to well developed; anterior and posterior surfaces of thighs mottled and blotched with dark brown; lateral surfaces of forearm, shank, tibia and foot with dark brown blotches; and a row of broad dark brown blotches present on either side of the lateral surfaces of abdomen.

**Phylogenetic relationships.** *Jingophrys vegrandis* is a member of the *J. vegrandis* species group and closely related to *Jingophrys tanogogo* sp. nov. (figures 1 and 2). Within its species group, *J. vegrandis* shows the following DNA sequence divergence in the mitochondrial 16S rRNA gene: 7.1–7.3% from *J. feii*; 5.7–6.0% from *Jingophrys siangensis* sp. nov.; 4.1–4.6% from *Jingophrys tanogogo* sp. nov.; 4.0–6.9% from *J. yeae* sensu lato; 3.2–3.8% from *J. zhaoermii* sensu lato; 4.5–4.7% from *J. zhoui*; and 5.7–6.0% from the undescribed candidate species *J*. cand. sp. A. (supplementary material, table S3).

**Morphological description.** A detailed description was published by Mahony *et al.* [4]. Additional morphological insights from new specimens reported in the present study include morphometric measurements (supplementary material, table S5) and intraspecific variation (figures 8 and 14), as well as additional diagnostic characters used to compare closely related congeners (see species accounts).

**Morphological variation.** *Skin texture*. Dorsum and snout shagreened to sparsely granular (SDBDU 2009.1274 and SDBDU 2023.6521) or granular (SDBDU 2022.6289), especially posteriorly; upper eyelids sparsely to prominently granular; fore- and hindlimbs smooth with prominent tubercles that correspond with transverse crossbands; flanks with prominent and large tubercles; tympanum smooth with slightly raised borders and surrounding region posterior to eye sparsely to prominently granular; well-developed supratympanic fold extends from posterior corner of eye to above forearm insertion, narrow anteriorly and slightly wider posteriorly at the level of arms; fingers and toes smooth; margins of inverted triangular marking between eyes with faint or prominent ridges; dorsolateral dermal ridge extends from behind supratympanic fold to near groin on either side; parietoscapular-sacral ridges form a vertically oriented ‘> <’ shaped (SDBDU 2022.6289) or ‘>-<’ shaped (SDBDU 2009.1274) configuration, composed of rows of small tubercles that are either continuous (SDBDU 2009.1274 and SDBDU 2022.6289) or discontinuous (SDBDU 2023.6521), the two arms of the first inverted ‘V’-shaped ridge begin above tympanum and extend posteriorly where they meet medially beyond the level of axilla, and the second inverted ‘V’-shaped ridge present at mid-dorsum. Ventral surfaces of body and limbs smooth; pectoral glands small, raised, rounded and positioned at the level of axilla; belly with tubercles on each side arranged from below forearm insertion to near groin; femoral glands small, rounded, weakly developed, slightly raised and closer to outer edge of knee than to cloaca (figure 8*d*,*o*).

**Secondary sexual characters.** Males: nuptial pads or spines not clearly visible other than a swelling on finger I; and a single subgular vocal sac while calling.

**Vocalization** **.** Calls of male *Jingophrys vegrandis* were recorded between 21.25 and 21.45 hours on 2 August 2023 at Sessa, West Kameng District, Arunachal Pradesh. Frogs called from leaves of shrubs adjacent to small hill streams. Ambient temperatures were 23°C dry bulb and 22°C wet bulb. Based on analyses of 50 calls from a single individual, the typical vocal repertoire of this species is as follows: males produce a single type of call that has a pulsatile temporal structure; calls are not delivered in groups; the mean call duration is 166.6 ms; the amplitude envelope has a mean rise time of 59.7 ms and a mean fall time of 106.9 ms; calls deliver 11–14 pulses at a mean rate of 71.03 pulses/s; and the call spectrum shows two broad peaks with a mean dominant frequency of 5.46 kHz (figure 10*i*–*l*; supplementary material, table S7).

**Call comparison.** Calls of *Jingophrys vegrandis* can be differentiated from those of other congeners primarily by their frequency, pulse rate, duration and rise time, as follows: (i) *Call frequency*: *J. vegrandis* calls have the highest dominant frequency (5.46 kHz) among all studied congeners (versus lower, 3.23 kHz in *Jingophrys hrdy* sp. nov., 4.43 kHz in *Jingophrys siangensis* sp. nov., 3.59 kHz in *Jingophrys taniitatii* sp. nov., 4.88 kHz in *Jingophrys tanogogo* sp. nov., 2.71 kHz in *Jingophrys wularsoni* sp. nov., 4.61 kHz in *J.* cf. *yeae* and 4.57 kHz in *J.* cf. *zhaoermii*). (ii) *Pulse rate*: *J. vegrandis* calls have the slowest pulse rate (71.03 pulses/s) among all studied congeners (versus faster, 127.48 pulses/s in *Jingophrys hrdy* sp. nov., 193.66 pulses/s in *Jingophrys siangensis* sp. nov., 184.13 pulses/s in *Jingophrys taniitatii* sp. nov., 140.98 pulses/s in *Jingophrys tanogogo* sp. nov., 140.8 pulses/s in *Jingophrys wularsoni* sp. nov., 102.55 pulses/s in *J.* cf. *yeae* and 99.46 pulses/s in *J.* cf. *zhaoermii*). (iii) *Call duration*: the duration of *J. vegrandis* calls (166.6 ms) is shorter than those of *Jingophrys hrdy* sp. nov. (versus longer, 180.2 ms), or longer (166.6 ms) than those of all other congeners (versus shorter, 59.7 ms in *Jingophrys siangensis* sp. nov., 54.49 ms in *Jingophrys taniitatii* sp. nov., 31.12 ms in *Jingophrys tanogogo* sp. nov., 51.66 ms in *Jingophrys wularsoni* sp. nov., 109.45 ms in *J.* cf. *yeae* and 78.86 ms in *J.* cf. *zhaoermii*). (iv) *Call rise time*: *J. vegrandis* calls also have the longest rise time (59.7 ms) among all studied congeners (versus shorter, 57.4 ms in *Jingophrys hrdy* sp. nov., 21.5 ms in *Jingophrys siangensis* sp. nov., 23.3 ms in *Jingophrys taniitatii* sp. nov., 14.7 ms in *Jingophrys tanogogo* sp. nov., 15.5 ms in *Jingophrys wularsoni* sp. nov., 39.2 ms in *J.* cf. *yeae* and 31.6 ms in *J.* cf. *zhaoermii*) (figure 10; supplementary material, table S7).

Additionally, we compared vocalizations of *J. vegrandis* from our study with those published for three members in the People's Republic of China [29,31]. Calls of *J. vegrandis* are longer in duration (166.6 ms) compared to those of *J. feii* and *J. yeae* (versus shorter, 132.6 and 139 ms, respectively), and shorter (166.6 ms) compared to *J.* cf. *pachyproctus* (versus longer, 746 ms); have fewer pulses per call, 11–14 pulses per call (versus higher, 14–20 pulses per call in *J. feii* and 22–62 pulses per call in *J.* cf. *pachyproctus*), and more pulses per call, 11–14 pulses per call (versus lower, 12 pulses per call in *J. yeae*); as well as a higher dominant frequency, 5.21–5.51 kHz (versus lower, 4.74–4.91 kHz in *J. feii*, 3.2–3.3 kHz in *J.* cf. *pachyproctus* and 4.7 kHz in *J. yeae*) (supplementary material, table S7).

**Tadpoles.** *External morphology*. The following description is based on a Gosner stage 26 tadpole, SDBDU 2024.6625: body cylindrical with equal height (BH 4.0) and width (BW 4.0); body length (BL 8.1) 30% of total length (TL 32.4); in dorsal view body broader (BW 4.0) than tail (TMW 2.6, TMW 64% of BW), horizontally elliptical, and with maximum body width at abdominal region; snout truncate in lateral view, the profile interrupted by an anterodorsally positioned oral disc; narial opening oval, anterolaterally positioned, with serrated rim and no projection; nares equidistant to both snout (SN 0.8) and eyes (NE 0.8); eyes medium-sized (ED 0.8) and positioned dorsolaterally; internarial distance (IND 2.2) 72% of interorbital distance (IOD 3.1); spiracle opens sinistrally at the level of hindlimbs, immediately caudal to the gills laterally; snout-to-spiracle distance (SS 4.3) 53% of body length (BL 8.1); spiracular tube short and narrow, directed laterally, with tip of inner wall of spiracular aperture free from body wall; vent tube opens medially at ventral end of body terminus, its dorsal wall medially attached to ventral fin but ends at a short distance before the tip; tail long (TAL 24.3), 75% of total length (TL 32.4), heavily muscularized (TMH 3.1, 70% of MTH 4.5 and TMW 2.6, 64% of BW 4.0); upper fin originates after body-tail junction (SU 12.7), lower in height at its proximal end; lower fin originates at ventral terminus of body; both fins of relatively low height, upper fin higher than lower fin (UFH 1.3, 28% of MTH 4.5, LFH 1.0, 22% of MTH 4.5); each fin reaches maximum height at mid-region of tail and tapers gradually into tail tip; tail muscle shows maximum height from body-tail junction to mid-region and then gradually tapers and ends before tail tip; tail tip bluntly pointed (figure 15*a*). Oral disc umbelliform, anterodorsally positioned, horizontally elliptical, wider (ODW 5.0) than body width (BW 4.0) (BW 80% of ODW), and lacking emarginations laterally; oral disc folds inward when not fully extended and in preservative; edges of the lip wavy and without marginal papillae; submarginal papillae on oral disc arranged in four to five rows in ventral view, oriented vaguely parallel to the transverse axis of oral disc as well as in a radiating pattern extending from inner region of oral cavity; about four rows of submarginal papillae arranged in parallel on dorsal side of funnel; submarginal papillae medium-sized, oval-shaped in area close to jaw sheath, gradually reduce in size and become more circular going towards outer margin of oral disc; submarginal papillae on dorsal side smaller than those on ventral side; tooth rows absent; jaw sheaths feebly keratinized and serrated; upper jaw sheath almost in straight line shape with a median notch; lower jaw sheath almost in straight line shape (figure 15*a*).

*Colour in life*. Overall colour of tadpole body and tail yellow with a shade of dark brown; dark brown melanocytes distributed mainly on lateral and dorsal body surfaces and tail muscle; tail fin mostly translucent, dusted with dark melanophores; dorsal margin of tail with orange shade; golden iridocytes completely cover body and tail; ventral body surfaces silvery; silver iridocytes on lateral body surfaces and anterior tail muscle; gills visible as red structures on venter; abdominal area opaque; gills and gut coil not visible; eyes black with golden iridocytes scattered on outer margin of sclera and forming orange ring around pupil; umbelliform oral disc with strong orange tinge and scattered dark brown pigmentation on lateral sides and submarginal papillae (figure 15*a*).

**Distribution.** *Jingophrys vegrandis* is currently known only from its type locality and vicinity in Sessa Village, West Kameng District, Arunachal Pradesh State, India, at elevations ranging from 1110 to 1250 m asl (figure 6; supplementary material, table S2).

**Habitat and natural history.** The habitat of *Jingophrys vegrandis* is primarily subtropical moist forest. The specific collection sites are located around a hill stream (2–3 m wide) that eventually drains into the large Sessa River. The stream and surrounding forest are not undisturbed, as local residents frequent the area to collect water with the help of small pipes and for occasional logging. However, the water body faces limited disturbance compared to other streams close to human settlements in the area. Our field surveys and collections were conducted during August in 2022 and 2023. Breeding activity likely peaks between mid-July and mid-August. Heavy rainfall encountered during the survey period also contributed to high water levels and flow in the stream. All calling individuals were located in thick and high shrub vegetation along the edges of the fast-flowing stream at heights approximately 1.5–2 m above-ground. We did not observe more than two calling individuals at any given specific site. During the surveys, at least three sympatric species (a large-sized *Xenophrys* sp., *Nanorana* sp. and *Amolops* sp.) were encountered along the same stretch of stream. This species feeds on terrestrial horntail snails (*Macrochlamys* cf. *indica*), as revealed through 3D micro-CT imaging (figure 13).

***Jingophrys yeae* (Shi, Zhang, Xie, Jiang, Liu, Ding, Luan and Wang, 2020)**

Figures 1–8, 10, 15 and 16; tables S1–S9 and figure S4 in the supplementary material

**Figure 16.**
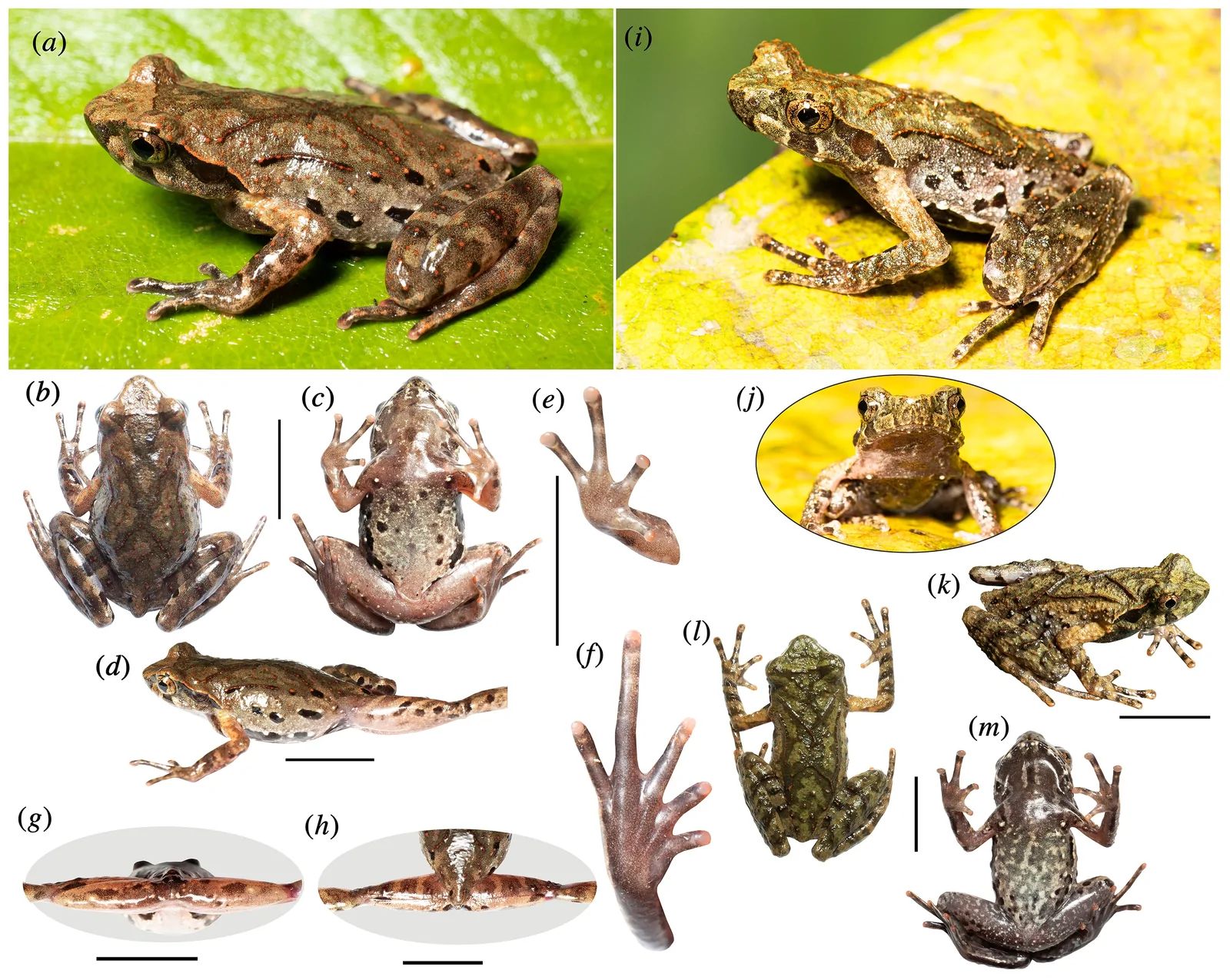
Adult males of *Jingophrys yeae* from India, in life. (*a–h*) SDBDU 2022.6121: (*a*) dorsolateral view, (*b*) dorsal view, (*c*) ventral view, (*d*) lateral view of flank, groin and thigh, (*e*) ventral view of hand, (*f*) ventral view of foot, (*g*) posterior view of thighs and (*h*) dorsal view of thighs; (*i*, *j*) SDBDU 2023.6379a: (*i*) dorsolateral view and (*j*) frontal view; (*k–m*) SDBDU 2025.6771: (*k*) dorsolateral view, (*l*) dorsal view and (*m*) ventral view. Scale bars = 10 mm. Photographs: S. D. Biju and T. Tajo.

**Original name and description.** *Megophrys yeae* Shi, Zhang, Xie, Jiang, Liu, Ding, Luan and Wang, 2020. In: Multiple data revealed two new species of the Asian horned toad *Megophrys* Kuhl & Van Hasselt, 1822 (Anura, Megophryidae) from the eastern corner of the Himalayas. ZooKeys, 977: 101–161, p. 140. **Holotype.** By original designation, CIB201706MT02, an adult male collected by SC Shi and L Ding, at 1.40 hours on 15 June 2017. **Paratypes.** 13 specimens (11 males and two females) from Medog County, Tibet Autonomous Region, People's Republic of China [31]. **Type locality.** Beibeng Village, Medog County, Tibet Autonomous Region, People's Republic of China (29.24292° N, 95.18561° E, 870 m). **Current status of specific name.** Valid name, as *Jingophrys yeae* (Shi, Zhang, Xie, Jiang, Liu, Ding, Luan and Wang, 2020).

**Taxonomic treatment of Indian populations.** *Jingophrys yeae* was originally described from Chinese populations (Medog) bordering India. Our new collections from the adjoining Indian regions in the state of Arunachal Pradesh and separated from the type locality by approximately 53–80 km in a southeasterly direction are closely related to this taxon but form a shallowly divergent sister cluster (figures 1, 2 and 6). Based on additional evidence from morphology and male calls, we treat Indian and Chinese populations as conspecific, *J. yeae* sensu lato, resulting in a new country record of this species from India. At the same time, Indian populations (referred here as *J*. cf. *yeae* for comparative purposes) differ from *J. yeae* sensu stricto as follows: (i) *Molecular phylogeny*: intraspecific genetic distances of 1.6–2.0% in mitochondrial 16S rRNA (supplementary material, table S3), and a divergence age of approximately 2.49 Ma based on multilocus data (figure 3). (ii) *Morphology*: morphometric measurements such as the ratios of upper eyelid width to distance between upper eyelids, UEW/IUE 0.68–0.76 (versus UEW/IUE 0.77–0.91), and of tympanum to tympanum-to-eye distance, TYD/TYE 1.21–1.46 (versus TYD/TYE 0.79–1.21 in *J. yeae* sensu stricto) and qualitative characters such as dorsal granulations (present study: figures 8 and 16) [31]. Call properties of the Indian population, however, are not significantly different and fall within broad ranges of those reported for the Chinese population (figure 10; present study: supplementary material, table S7) [31]. Below, we provide detailed descriptions of adult and tadpole morphology, male calls and comparisons with closely related species from India, as well as a revised species distribution range with additional natural history observations. Morphometric measurements are included in table S5 in the supplementary material.

**Referred specimens.** Two adult males, SDBDU 2023.6379a–6379b, from Tiwari Gaon Village (28.2115° N, 95.8067° E, 954 m asl and 28.2193° N, 95.8225° E, 1455 m asl), Lower Dibang Valley District, Arunachal Pradesh State, India, collected by SDB, RUK and TT on 28 February 2023; and five adult males, SDBDU 2022.6120–6122, and SDBDU 2022.6125a–6125b, from Anini Village (28.8097° N, 95.9273° E, 1390 m asl), Dibang Valley District, Arunachal Pradesh State, India, collected by SDB, RUK and TT on 19 July 2022.

**Etymology.** The specific epithet honours Professor Ye Chang-Yuan, a Chinese amphibian researcher, for her contributions to Chinese herpetology [31].

**Suggested common name.** Ye's horned frog.

**Diagnosis.** This species can be distinguished from known congeners by the following combination of characters: first finger weakly developed and much smaller; large, raised tubercles on flanks and posterior part of dorsum, surrounded with blackish brown; supratympanic fold narrow anteriorly but does not widen posteriorly; toes flat with weakly developed lateral fringes; and prominent brownish-black blotches on belly tubercles.

**Phylogenetic relationships.** *Jingophrys yeae* sensu lato is a member of the *J. vegrandis* species group. It appears to be more closely related to *J. zhaoermii* based on various analyses but does not evince a clear sister-group relationship (figures 1 and 2). Within its species group, *J. yeae* sensu lato shows the following DNA sequence divergence in the mitochondrial 16S rRNA gene: 7.7–8.3% from *J. feii*; 6.9–7.5% from *Jingophrys siangensis* sp. nov.; 5.3–6.5% from *Jingophrys tanogogo* sp. nov.; 4.0–6.9% from *J. vegrandis*; 4.6–5.2% from *J. zhaoermii* sensu lato; 6.0–6.5% from *J. zhoui*; and 6.2–6.8% from the undescribed candidate species *J*. cand. sp. A. (supplementary material, table S3). For separate comparisons of *J. yeae* sensu stricto and *J.* cf. *yeae* populations with other members, see table S3 in the supplementary material.

**Morphological description.** The following detailed description is based on adult specimens from India (*n =* 6, males). Small adult size (SVL 24.9–28.0 mm), head nearly as wide as long; snout rounded in dorsal view, acutely protruding in lateral view, dorsal region concave, snout tip projects far beyond the lower lip; loreal region nearly vertical, concave; canthus rostralis rounded; eye diameter larger than maximum tympanum diameter but smaller than snout length; tympanum-to-eye distance shorter than maximum tympanum diameter; tympanum distinct, rounded, upper border concealed by supratympanic ridge; pupil oval in preservative, oriented vertically; nostril oval, oriented laterally; distance between upper eyelids nearly equal to snout length; internarial distance larger than upper eyelid width but shorter than narrowest point between upper eyelids; pineal ocellus not visible externally; vomerine ridges weakly developed without teeth; maxillary teeth present; tongue large, rounded, with no medial lingual process. Forelimbs long; forearm shorter than hand; fingers moderately long, thin, lateral dermal fringes absent, relative finger lengths I < II < IV < III (or I < IV < II < III); interdigital webbing and subarticular and supernumerary tubercles absent; thenar and outer metacarpal tubercles weakly developed; fingertips rounded, raised ventrally, not expanded laterally relative to digit widths, terminal grooves absent on fingertips. Hindlimbs long, thin; thigh shorter than shank and longer than foot; toes without lateral dermal fringes, relative toe lengths I < II < V < III < IV; toe tips rounded, not dilated, with distinct pads raised ventrally; terminal grooves absent; base of toes with rudimentary webbing; outer metatarsal tubercle absent; subarticular and supernumerary tubercles absent; inner metatarsal tubercle present but barely distinguishable (figure 16).

*Skin texture*. Dorsum and head shagreened to sparsely granular; dorsal surfaces of snout sparsely granular; upper eyelids sparsely to prominently granular; fore- and hindlimbs smooth with prominent tubercle corresponding with transverse crossbands; flanks with prominent and large tubercles; posterior part of thighs sparsely to prominently granular, especially close to cloacal opening; tympanum smooth with slightly raised borders and surrounding region posterior to the eye sparsely to prominently granular; supratympanic fold well developed, extends from posterior corner of eye to above forearm insertion, narrow anteriorly and slightly wider posteriorly at the level of arms, ending with a glandular tubercle; fingers and toes smooth; margins of inverted triangular marking between eyes with or without faint ridges; dorsolateral dermal ridge extends from behind supratympanic fold to near groin on either side; parietoscapular-sacral ridges form a vertically oriented ‘> <’ or ‘>-<’ configuration, composed of rows of small tubercles, the two arms of the first inverted ‘V’-shaped ridge begin above the tympanum and extend posteriorly where they meet medially beyond the level of axilla, and the second inverted ‘V’-shaped ridge present at mid-dorsum. Ventral surfaces of body and limbs smooth; pectoral glands small, raised, rounded and positioned at the level of axilla; belly with tubercles on both sides, arranged from below forearm insertion to near groin; femoral glands small, rounded, weakly developed, slightly raised, closer to outer edge of knee than to cloaca; prominent tubercles on cloacal region. Ventral surface of body smooth; a few granular projections on upper margins of lower jaw; snout tip projection beyond lower lip sparsely granular; pectoral glands small and raised, rounded, positioned at level with axilla; both sides of belly with tubercles arranged from below forearm insertion to near groin; femoral glands small, rounded, weakly developed, slightly raised and closer to outer edge of knee than to cloaca; prominent tubercles surrounding cloacal opening (figure 8*e*,*p*).

*Colour in life*. Dorsum and fore- and hindlimbs light greyish brown (SDBDU 2023.6379) or greenish brown (SDBDU 2022.6122); roughly ‘X’-shaped light brown mark present on dorsum; inverted triangular markings between eyes brown; borders of ridges and tubercles blackish brown, with a tinge of light orange-red; inner margin of supratympanic folds dark brown; anterior snout dark brown; a horizontal light brown band extends from anterior margin of loreal region and over entire tympanum with a break at the eye; lateral surfaces of head greyish brown; wide vertical dark brown bands along upper lip and lateral surfaces of head; flanks lighter than dorsum, tubercles on flanks dark brown; finger and toe tips light orange-red; iris reddish brown with golden tinge; dorsal surfaces of fore- and hindlimbs (including fingers and toes) with brownish-black crossbands that extend on ventral surfaces. Ventral surfaces of throat and chest light reddish brown (SDBDU 2023.6379) or greyish brown (SDBDU 2022.6122); lateral surfaces of belly (visible ventrally) with broad blackish-brown stripe (continuous in SDBDU 2022.6125; discontinuous in SDBDU 2022.6120, SDBDU 2022.6121, SDBDU 2022.6122, SDBDU 2023.6379 and SDBDU 2022.6125a; or scattered blackish-brown spots in SDBDU 2022.6125); anterior and posterior parts of thighs with larger blackish-brown blotches (prominent in SDBDU 2022.6125, SDBDU 2022.6120, SDBDU 2022.6121 and SDBDU 2022.6122; faintly present in SDBDU 2023.6379 and SDBDU 2022.6125a); two pale grey streaks extend from lower margin of jaw joint halfway to ventral surface of upper arm, with two vertical lines on centre of throat; belly light grey with minute black spots (SDBDU 2023.6379 and SDBDU 2022.6125); prominent blackish-brown spots on belly (SDBDU 2022.6120); blackish-brown colouration around cloacal opening (figure 16). *In preservative*. Dorsum, limbs, lateral surfaces of head and flanks light or dark greyish brown; inverted triangular marking between eyes light brown; ‘X’-shaped marking on dorsum faint; all blackish-brown blotches turned to greyish brown. Ventral surfaces of throat and chest light-brown or greyish brown; belly light grey or light greyish brown; blotches light-brown; hand and foot light greyish brown (supplementary material, figure S4).

**Secondary sexual characters.** Males: nuptial pads or spines not clearly visible other than a swelling on finger I; and a single subgular vocal sac while calling.

**Vocalization** **.** Calls of male *Jingophrys* cf. *yeae* were recorded between 19.00 and 19.15 hours on 19 and 25 July 2023 at Anini, Dibang Valley District, Arunachal Pradesh. Calling was observed from 18.30 to 23.30 hours. The frogs called from leaves of small plants near small rocky streams. Ambient temperatures at the two study sites were 22.8°C and 20.5°C dry bulb, and 20.2°C and 17.8°C wet bulb, respectively. Based on analyses of 50 calls from two individuals, the typical vocal repertoire of this species is as follows: males produce a single type of call that has a pulsatile temporal structure; calls are not delivered in groups; the mean call duration is 109.5 ms; the amplitude envelope has a mean rise time of 39.2 ms and a mean fall time of 70.2 ms; calls deliver 11–13 pulses at a mean rate of 102.55 pulses/s; and the call spectrum shows two broad peaks with a mean dominant frequency of 4.61 kHz (figure 10*m*–*p*; supplementary material, table S7).

**Call comparison.** Calls of *Jingophrys* cf. *yeae* can be differentiated from those of other congeners primarily by their frequency, pulse rate, duration and rise time, as follows: (i) *Call frequency*: *J.* cf. *yeae* calls differ from those of *Jingophrys hrdy* sp. nov., *Jingophrys siangensis* sp. nov., *Jingophrys taniitatii* sp. nov., *Jingophrys wularsoni* sp. nov. and *J.* cf. *zhaoermii* by their higher dominant frequency, 4.61 kHz (versus lower, 3.23, 4.43, 3.59, 2.7 and 4.57 kHz, respectively); and from *Jingophrys tanogogo* sp. nov. and *J. vegrandis* by their lower dominant frequency, 4.61 (versus higher, 4.88 and 5.46 kHz, respectively). Although the dominant frequency of *J.* cf. *yeae* calls falls within the range of those of *Jingophrys siangensis* sp. nov., *J.* cf. *yeae* calls can be distinguished by their faster pulse rate. (ii) *Pulse rate*: *J.* cf. *yeae* calls differ from those of *J. vegrandis* and *J.* cf. *zhaoermii* by their faster pulse rate, 102.55 pulses/s (versus slower, 71.03 and 99.46 pulses/s, respectively); and from *Jingophrys hrdy* sp. nov., *Jingophrys siangensis* sp. nov., *Jingophrys taniitatii* sp. nov., *Jingophrys tanogogo* sp. nov. and *Jingophrys wularsoni* sp. nov. by their slower pulse rate, 102.55 pulses/s (versus faster, 127.48, 196.66, 184.13, 140.98, 140.8 pulses/s, respectively). (iii) *Call duration*: *J.* cf. *yeae* calls differ from those of *Jingophrys siangensis* sp. nov., *Jingophrys taniitatii* sp. nov., *Jingophrys tanogogo* sp. nov., *Jingophrys wularsoni* sp. nov. and *J.* cf. *zhaoermii* by their longer duration, 109.5 ms (versus shorter, 59.7, 54.5, 31.1, 51.7 and 78.9 ms, respectively); and from *Jingophrys hrdy* sp. nov. and *J. vegrandis* by their shorter call duration, 109.5 ms (versus longer, 180.2 and 166.6 ms, respectively). (iv) *Call rise time*: *J.* cf. *yeae* calls differ from those of *Jingophrys siangensis* sp. nov., *Jingophrys taniitatii* sp. nov., *Jingophrys tanogogo* sp. nov., *Jingophrys wularsoni* sp. nov. and *J.* cf. *zhaoermii* by their longer rise time, 39.2 ms (versus shorter, 21.5, 23.3, 14.7, 15.6 and 31.6 ms, respectively) (figure 10; supplementary material, table S7).

Additionally, we compared vocalizations of *J.* cf. *yeae* from our study with those published for three members in the People's Republic of China [29,31]. Calls of *J.* cf. *yeae* have a slightly lower dominant frequency (4.35–4.82 kHz) compared to those of *J. feii* (versus higher, 4.74–4.91 kHz), and a higher dominant frequency (4.35–4.82 kHz) compared to those of *J.* cf. *pachyproctus* (versus lower, 3.2–3.3 kHz); *J.* cf. *yeae* calls are shorter in duration, 109.4 ms (versus longer, 132.6 ms in *J. feii* and 746 ms in *J.* cf. *pachyproctus*, respectively); and *J.* cf. *yeae* calls have fewer pulses, 11–13 pulses per call (versus more, 14–20 pulses per call in *J. feii* and 22–62 pulses per call in *J.* cf. *pachyproctus*, respectively) (supplementary material, table S7). Call properties of *J.* cf. *yeae* from the present study are comparable to those of *J. yeae* sensu stricto from published work [31], with dominant frequencies within the same range (4.35–4.82 kHz versus 4.4–5.2 kHz), although the mean frequency of *J.* cf. *yeae* is slightly lower (4.35 kHz versus 4.7 kHz). Similarly, call durations overlap within the same broad range (99–212 ms versus 99.4–124.1 ms), although mean call duration is slightly lower in *J.* cf. *yeae* (109.4 ms versus 139 ms) ( supplementary material, table S7).

**Tadpoles.** *External morphology*: the following description is based on a Gosner stage 28 tadpole, SDBDU 2023.6512: body cylindrical with height (BH 3.8) 90% of the width (BW 4.1); body length (BL 8.5) 30% of total length (TL 30.3); in dorsal view, body broader (BW 4.1) than tail (TMW 2.5, TMW 61% of BW), horizontally elliptical, and with maximum body width at abdominal region; snout truncate in lateral view, the profile interrupted by an anterodorsally positioned oral disc; narial opening oval, anterolaterally positioned, with serrated rim and no projection; nares half as close to snout (SN 0.8) than to eyes (NE 1.6); eyes medium-sized (ED 0.7) and positioned dorsolaterally; internarial distance (IND 1.9) 62% of interorbital distance (IOD 3.0); spiracle opens sinistrally at level of hindlimbs, immediately caudal to the gills laterally; snout-to-spiracle distance (SS 4.9) 57% of BL (BL 8.5); spiracular tube short and narrow, directed laterally, with tip of inner wall of spiracular aperture free from body wall; vent tube opens medially at ventral end of body terminus, its dorsal wall medially attached to ventral fin but ends a short distance before tip; tail long (TAL 21.8), 72% of total length (TL 30.3), heavily muscularized (TMH 2.9, 65% of MTH, 4.4; and TMW 2.5, 61% of BW 4.1); upper fin originates after body-tail junction (SU 8.7), lower in height at proximal end; lower fin originates at ventral terminus of body; both fins of low relative height, upper fin slightly higher than lower fin (UFH 1.0, 22% of MTH 4.4, LFH 0.9, 21% of MTH 4.4); each fin reaches maximum height at mid-region of tail and tapers gradually to tail tip; tail muscle shows maximum height from body-tail junction to mid-region, then gradually tapers and ends before tail tip; tail tip bluntly pointed (figure 15*b*). Oral disc umbelliform, anterodorsally positioned, horizontally elliptical, wider (ODW 4.4) than body width (BW 4.1) (BW 96% of ODW) and lacking emarginations laterally; oral disc folds inward when not fully extended and in preservative; edges of lip wavy and without marginal papillae; submarginal papillae on oral disc arranged in four to five rows in ventral view, oriented vaguely parallel to transverse axis of oral disc as well as in radiating pattern extending from inner region of oral cavity; about four rows of submarginal papillae arranged in parallel on dorsal side of funnel; submarginal papillae pustule-like, larger and oval-shaped in area close to jaw sheath, gradually reduce in size and become more circular towards outer margin of oral disc; single long ridge-like papillae located closer to oral cavity on lateral sides of oral disc; submarginal papillae on dorsal side smaller than those on ventral side; tooth rows absent; jaw sheaths feebly keratinized and serrated; upper jaw sheath almost in straight line shape with a median notch; lower jaw sheath almost in straight line shape (figure 15*b*).

*Colour in life*. Overall colour of tadpole body and tail beige with a shade of dark brown; dark brown melanocytes distributed mainly on lateral and dorsal body surfaces and tail muscle; tail fin mostly translucent, dusted with dark melanophores; golden iridocytes distributed throughout dorsal and lateral surfaces of body and sparsely on ventral body surfaces, tail muscle and tail fins; gills visible as red structures on ventral body surface; abdominal area transparent; gut coil partially visible; eyes black with few golden iridocytes scattered on outer margin of sclera form orange ring around pupil; umbelliform oral disc with dark brown pigmentation on lateral sides and on submarginal papillae (figure 15*b*).

**Distribution.** *Jingophrys yeae* was previously known only from Medog County, Tibet Autonomous Region, People's Republic of China, between elevations of 670 and 1780 m asl [31,107]. Our study extends the reported distribution of the species into India. Our new collections are from multiple locations (Anini, Etalin and Tiwari Gaon) in the Dibang and Lower Dibang Valley districts of Arunachal Pradesh state, India, at elevations ranging from 714 to 1455 m asl (figure 6; supplementary material, table S2).

**Habitat and natural history.** In India, the calling males of *Jingophrys yeae* were found on small shrubs adjacent to minor streams with no large water bodies nearby. All sampling sites were located close to roads and lacked much vegetation except for some shrubs where individuals were found perched mostly on leaves or stems, and sometimes on trees. This species is likely to be present further upstream along steep and densely covered high canopy forests. The forest vegetation type varies with elevation: it is tropical evergreen at Etalin, but subtropical moist forest at Anini. Collections were made after dusk around 19.00–20.00 hours during July 2022. No heavy rainfall was encountered during surveys. All sites being close to roads, they experienced occasional disturbance from passing vehicles. Nevertheless, the calls were loud and clearly audible despite background vehicular noise. At a single location, 4–6 calling individuals were observed. Individuals usually sit on a single leaf while calling. Tadpoles were collected at Anini from a small water puddle below vegetation used as calling sites.

***Jingophrys zhaoermii* Huang, Liu, Chen and Yuan, 2026**

Figures 1–8, 10 and 17; tables S1–S9 and figure S5 in the supplementary material

**Figure 17.**
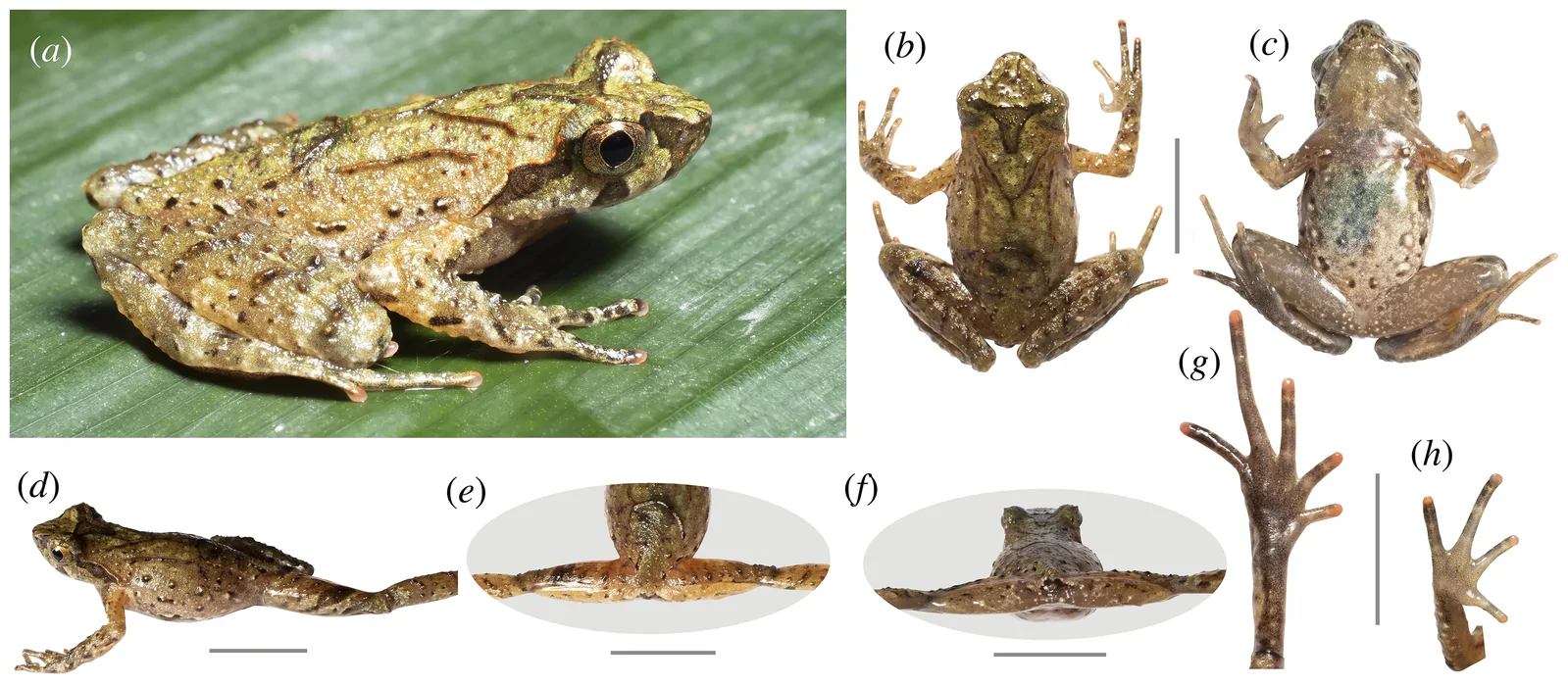
*Jingophrys zhaoermii* from India (SDBDU 2023.6376), in life. (*a*) Dorsolateral view, (*b*) dorsal view, (*c*) ventral view, (*d*) lateral view of flank, groin and thigh, (*e*) dorsal view of thighs, (*f*) posterior view of thighs, (*g*) ventral view of foot and (*h*) ventral view of hand. Scale bars = 10 mm. Photographs: T. Tajo.

**Original name and description.** *Jingophrys zhaoermii* Huang, Liu, Chen and Yuan, 2026. In: Yuan X, Chen J, Ai R, Lu W, Liu X, Zhou W, Huang J and Yuan Z. A new Asian horned toad of the genus *Jingophrys* (Amphibia, Megophryidae) from Xizang, People's Republic of China. Zootaxa, 5763 (4): 551–567, p. 557. **Holotype.** By original designation, SWU 0015896, an adult male collected by Renda Ai and Wenzhu Lu on 11 May 2025. **Paratypes.** Three specimens (one adult male and two adult females) collected with the holotype. **Type locality.** Gelin Village, Medog County, Xizang Tibetan Autonomous Region, People's Republic of China (29.151808° N, 95.226333° E, 2589.52 m asl). **Current status of specific name.** Valid name, as *Jingophrys zhaoermii* Huang, Liu, Chen and Yuan, 2026.

**Taxonomic treatment of Indian populations.** *Jingophrys zhaoermii* was originally described based on populations from the Medog region of the People's Republic of China bordering India. Our new collections from two localities in the Anjaw District (Hayuliang-Chaglagom and Kupha) of the adjoining Indian state of Arunachal Pradesh, and separated from the type locality by approximately 170 km in a southeasterly direction, form a shallowly divergent sister-cluster in the mitochondrial 16S analyses (figures 1 and 6), but there is insufficient support for the sister-group relationship in the multilocus dataset (figures 2 and 3). However, based on additional evidence from morphology, we currently assign the Indian populations to *J. zhaoermii* sensu lato, which thus represents a new country record of this species from India. At the same time, the Indian populations (referred here as *J.* cf. *zhaoermii* for comparative purposes) differ from *J. zhaoermii* sensu stricto as follows: (i) *Molecular phylogeny*: Intraspecific genetic distances of 1.6% in mitochondrial 16S rRNA (supplementary material, table S3) and divergence more than 4.0 Ma from any other currently known *Jingophrys* populations, and specifically from *J.* cf. *zhaoermii* (figure 3); and (ii) *Morphology*: morphometric measurements such as small adult body size, male SVL 22.5–26.5 mm (versus male SVL 34.5–35.4 mm; female SVL 39.6–43.1 mm) and head longer than wide, HL/HW 1.14–1.19 (versus equal or slightly wider, HL/HW 0.98–1, and qualitative characters such as dorsal skin shagreened with scattered granular projections (versus relatively smooth and lack granules), well developed and continuous dorsolateral dermal ridges (versus absent or weak), and flanks, including lateral surfaces of belly, with prominent tubercles (versus sparse large tubercles on flanks) (present study: figures 8 and 17) [19]. Below we provide detailed morphological descriptions and comparisons of our new populations with closely related species from India, and the first report and description of male calls for this species, as well as a revised species geographical distribution and additional natural history observations. Morphometric measurements are included in table S5 in the supplementary material.

**Referred specimens.** Three adult males, SDBDU 2023.6365, SDBDU 2023.6376 and SDBDU 2023.6377, from Khupa (28.0826° N, 96.4651° E, 785 m asl), Anjaw District, Arunachal Pradesh State, India, collected by SDB, RUK and TT on 26 February 2023.

**Etymology.** The specific epithet honours the renowned Chinese herpetologist, Professor Er-Mi Zhao, for his significant contributions to comprehensive scientific expeditions on the Tibetan Plateau [19].

**Suggested common name.** Zhao's horned toad [19]; Zhao's horned frog (present study).

**Diagnosis.** This species can be distinguished from known congeners by the following combination of characters: small adult male size, SVL 22.5–26.5 mm, *n =* 3; weakly developed dermal fringes on toes; rudimentary webbing between toes; well-developed dorsolateral dermal ridges and parietoscapular-sacral ridges; belly with well-developed tubercles on either side that extend from below forearm insertion to near groin; and dorsal colouration in life greyish green.

**Phylogenetic relationships.** *Jingophrys zhaoermii* sensu lato is a member of the *J. vegrandis* species group. It is closely related to *J. yeae* based on various analyses but does not evince a clear sister-group relationship (figures 1–3). Within its species group, *J. zhaoermii* sensu lato shows the following DNA sequence divergence in the mitochondrial 16S rRNA gene: 6.3–6.9% from *J. feii*; 6.7% from *Jingophrys siangensis* sp. nov.; 4.5–4.7% from *Jingophrys tanogogo* sp. nov.; 3.2–3.8% from *J. vegrandis*; 4.6–5.2% from *J. yeae* sensu lato; 4.8–5.1% from *J. zhoui*; and 5.1% from the undescribed candidate species *J*. cand. sp. A. (supplementary material, table S3). For separate comparisons of *J. zhaoermii* sensu stricto and *J.* cf. *zhaoermii* populations with other members, see table S3 in the supplementary material.

**Morphological description.** The following detailed description is based on adult specimens reported here from India (*n =* 3, males) (figure 17; supplementary material, table S5). Small adult size (SVL 22.5–26.5 mm), slender body; head moderately large, longer than wide; snout rounded in dorsal view, acutely protruding in lateral view, snout tip projects far beyond lower lip; loreal region vertical, concave; canthus rostralis sharp (angled); dorsal snout concave; eye larger than maximum tympanum diameter but smaller than snout length; tympanum-to-eye distance slightly shorter than maximum tympanum diameter; tympanum moderately distinct, oval, dorsally compressed, upper border concealed by supratympanic ridge; pupil oval in preservative, oriented vertically; nostril oval, oriented laterally, closer to eye than to snout; internarial distance larger than upper eyelid width, and smaller than narrowest point between upper eyelids; distance between posterior margins of eyes nearly 1.5 times the distance between anterior margins of eyes; pineal ocellus not visible externally; vomerine ridges and teeth absent; maxillary teeth present; tongue large, rounded, with no medial lingual process. Forelimbs long, thin; forearm shorter than hand; fingers moderately long, thin, lateral dermal fringes absent, relative finger lengths I < IV < II < III; interdigital webbing, and subarticular and supernumerary tubercles absent; thenar and outer metacarpal tubercles weak; fingertips rounded and raised ventrally, not expanded relative to digit widths, with small oval pads, terminal grooves absent on fingertips. Hindlimbs long, thin; thigh shorter than shank and longer than foot; toes short and flattened, with moderate lateral dermal fringes, relative toe lengths I < II < V < III < IV; tips not dilated, but with distinct raised pad on ventral side; terminal grooves absent; base of toes without webbing; outer metatarsal tubercle absent; subarticular and supernumerary tubercles absent; inner metatarsal tubercle present but barely distinguishable (figure 17).

*Skin texture*. Dorsal surfaces of head, upper eyelids, dorsum and limbs shagreened with scattered granular projections; more prominently granular on posterior end of dorsum (SDBDU 2023.6376) or more prominently granular throughout dorsum (SDBDU 2023.6377); flanks, including lateral surfaces of belly, with prominent tubercles; canthal region smooth; tympanum smooth with spinules, borders slightly raised, surrounding area posterior to eye granular; supratympanic fold well developed, narrow with slight widening posteriorly, extends from posterior corner of eye to region above forearm insertion; prominent tubercle present on outer edge of upper eyelid with a tiny horn-like tubercle in the middle; an inverted triangular ridge in between eyes extends halfway onto upper eyelids; short, thin and continuous dorsolateral dermal ridge extends on either side from just above level of forearms to below mid-dorsum but above level of groin; parietoscapular-sacral ridges form a ‘>-<’ configuration, the two arms of the first inverted ‘V’-shaped ridge begin behind eye and extend posteriorly where they meet medially beyond the level of axilla, and the second inverted nearly ‘V’ or ‘U’-shaped ridge present at mid-dorsum; dorsal surface of thighs with a row of round tubercles placed horizontally; shank with several rows of transversely placed small tubercles corresponding with the crossbands. Ventral surfaces of throat, chest, belly and limbs smooth; pectoral glands small, raised, rounded and positioned at level of axilla; belly with tubercles on both sides, arranged from below forearm insertion to near groin; femoral glands small, rounded, weakly developed, slightly raised, closer to outer edge of knee than to cloaca; prominent tubercles in cloacal region (figure 8*g*,*r*).

*Colour in life*. Dorsum, limbs, lateral surfaces of head and belly greyish green (SDBDU 2023.6376) or dark greyish brown (SDBDU 2023.6377), lighter on lateral surface of belly; granular ridges and granulations orangish red and surrounded with greyish brown; inverted triangular marking between eyes blackish brown with light central blotch; supratympanic folds orangish red; broad horizontal greyish-brown band extends from posterior corner of eye to posterior end of supratympanic fold on either side; broad vertical bands on margins of upper lip just below eye; anterior part of snout dark brown; tubercles on flanks, including lateral surfaces of belly, surrounded with blackish brown; limbs including fingers and toes with greyish-brown crossbands, more prominent and contrasting in SDBDU 2023.6377; thigh and shank cross bands correspond with prominent warts; iris reddish brown with golden tinge. Ventral margins of lower jaw with faint alternating light brown and light grey bands; throat and chest light brown with vertical dark greyish-brown streak and obscure greyish-brown spots; belly grey with brownish tinge and obscure light brown spots; centre part of belly with bluish-black patch; white spots on pectoral glands and belly with tubercles; fore- and hindlimbs light grey with irregular yellowish-brown spots; posterior part of thighs light greyish brown with light blackish-brown spots; lateral surfaces of thigh and shank with light blackish-brown crossbands that extend towards ventral surfaces; blackish-brown colouration around the cloacal opening; tips of fingers and toes and margins of toes light orangish red (figure 17). *In preservative*. Dorsum uniformly ash grey with minute blackish-brown spots; limbs slightly lighter than dorsum; inverted triangular marking between eyes light brown; granular ridges and granulations surrounded with greyish brown; limbs grey with blackish-brown crossbands. Ventral surfaces of throat light grey with minute blackish-brown spots; belly light grey with faint black spots (supplementary material, figure S5).

**Secondary sexual characters.** Males: nuptial pads or spines not visible; and a single subgular vocal sac while calling.

**Vocalization** **.** Calls of male *Jingophrys* cf. *zhaoermii* were recorded between 18.30 and 19.00 hours on 26 February 2023 at Hayuliang–Chaglagam road, Anjaw District, Arunachal Pradesh. Ambient temperatures were 17.5°C dry bulb and 14.5°C wet bulb. Based on analyses of 50 calls from two individuals, the typical vocal repertoire of this species is as follows: males produce a single type of call that has a pulsatile temporal structure; calls are delivered in groups; the mean call duration is 78.9 ms; the amplitude envelope has a mean rise time of 31.6 ms and a mean fall time of 47.2 ms; calls deliver 8–10 pulses at a mean rate of 99.46 pulses/s; and the call spectrum shows two broad peaks with a mean dominant frequency of 4.57 kHz (figure 10*q*–*t*; supplementary material, table S7).

**Call comparison.** Calls of *Jingophrys* cf. *zhaoermii* can be differentiated from those of other congeners primarily by their frequency, pulse rate, duration and rise time, as follows: (i) *Call frequency*: *J.* cf. *zhaoermii* calls differ from those of *Jingophrys hrdy* sp. nov., *Jingophrys siangensis* sp. nov., *Jingophrys taniitatii* sp. nov. and *Jingophrys wularsoni* sp. nov. by their higher dominant frequency, 4.57 kHz (versus lower, 3.23, 4.43, 3.59 and 2.7 kHz, respectively), and from *Jingophrys tanogogo* sp. nov., *J. vegrandis*, and *J.* cf. *yeae* by their lower dominant frequency, 4.57 kHz (versus higher, 4.88, 5.46 and 4.61 kHz, respectively). Although the dominant frequency of *J.* cf. *zhaoermii* falls within the range of *J.* cf. *yeae*, the calls of *J.* cf. *zhaoermii* are shorter. (ii) *Pulse rate*: *J.* cf. *zhaoermii* calls differ from those of *J. vegrandis* by their faster pulse rate, 99.46 pulses/s (versus slower, 71.03 pulses/s), and from those of *Jingophrys siangensis* sp. nov., *Jingophrys hrdy* sp. nov., *Jingophrys taniitatii* sp. nov., *Jingophrys tanogogo* sp. nov., *Jingophrys wularsoni* sp. nov. and *J.* cf. *yeae* by their slower pulse rate, 99.46 pulses/s (versus faster, 193.66, 127.48, 184.13, 140.98, 140.8 and 102.55 pulses/s, respectively). Although the pulse rate of *J.* cf. *zhaoermii* calls falls within the range of those of *J.* cf. *yeae*, the calls of *J.* cf. *zhaoermii* are shorter. (iii) *Call duration*: *J.* cf. *zhaoermii* calls differ from those of *Jingophrys siangensis* sp. nov., *Jingophrys taniitatii* sp. nov., *Jingophrys tanogogo* sp. nov. and *Jingophrys wularsoni* sp. nov. by their longer duration, 78.9 ms (versus shorter, 59.7, 54.5, 31.12 and 51.7 ms, respectively), and from those of *Jingophrys hrdy* sp. nov., *J. vegrandis* and *J.* cf. *yeae* by their shorter duration, 78.9 ms (versus longer, 180.2, 166.6 and 109.45 ms, respectively). (iv) *Call rise time*: *J.* cf. *zhaoermii* calls differ from those of *Jingophrys siangensis* sp. nov., *Jingophrys taniitatii* sp. nov., *Jingophrys tanogogo* sp. nov. and *Jingophrys wularsoni* sp. nov. by their longer rise time, 31.6 ms (versus shorter, 21.5, 15.6, 14.7 and 23.3 ms, respectively); and from those of *Jingophrys hrdy* sp. nov., *J. vegrandis* and *J.* cf. *yeae* by their shorter rise time, 31.6 ms (versus longer, 57.4, 59.7 and 39.2 ms, respectively) (figure 10; supplementary material, table S7).

Additionally, we compared vocalizations of *J.* cf. *yeae* from our study with those published for three members in the People's Republic of China [29,31]. Calls of *J.* cf. *zhaoermii* have a slightly lower dominant frequency (4.31–4.78 kHz) compared to those of *J. feii* and *J. yeae* sensu stricto (versus higher, 4.74–4.91 kHz and 4.4–5.2 kHz, respectively), but a higher dominant frequency (4.31–4.78 kHz) compared to those of *J.* cf. *pachyproctus* (versus lower, 3.2–3.3 kHz). Calls of *J.* cf. *zhaoermii* also differ from those of *J. feii*, *J.* cf. *pachyproctus*, and *J. yeae* by their shorter duration, 78.9 ms (versus longer, 132.6, 746 and 139 ms, respectively), and from those of *J. feii* and *J.* cf. *pachyproctus* by their fewer pulses per call, 8–10 pulses per call (versus more, 14–20 pulses per call and 22–62 pulses per call, respectively) (supplementary material, table S7).

**Distribution.** *Jingophrys zhaoermii* was previously known only from its type locality, Gelin Village in Medog County, Tibet Autonomous Region, People's Republic of China, at an elevation of 2589.52 m asl [19]. Our study extends the reported distribution of the species into India. Our new collections are from two locations (Hayuliang-Chaglagom and Kupha) in the Anjaw District of Arunachal Pradesh State, India, at elevations between 780 and 785 m asl (figure 6; supplementary material, table S2).

**Habitat and natural history.** In India, individuals of *Jingophrys* cf. *zhaoermii* were located in tropical dry evergreen forests with less canopy cover owing to undulating terrain and a broad fast-flowing stream (approx. 6–9 m wide). Water from the stream flows over the road to join a tributary of the Lohit River. The edges of the stream were covered in thick shrubs. All individuals were collected after dusk and found perched on leaves of shrubs adjacent to the stream. Calling individuals were recorded and collected around 20.00 hours during February 2023, which is the earliest time of year when we observed any *Jingophrys* species active in the present study. We found 3 to 4 calling individuals at this time, but did not encounter adult individuals in a later survey during the peak monsoon season in July 2023.

#### 3.11.2. *Jingophrys pachyproctus* species group members

A member of this species group was previously believed to occur in India as ‘*Megophrys pachyproctus* Huang, 1981’ (= *Jingophrys pachyproctus* (Huang, 1981)), based on a single specimen [32]. This report requires further confirmation owing to the absence of detailed comparisons with current members of the later-recognized genus *Jingophrys*. Based on our extensive new collections and comprehensive investigations using multiple lines of evidence, we confirm that *J. pachyproctus* (Huang, 1981) is currently not known to occur in India. We further conclude that the previous report of this taxon from Tale Valley WLS is assignable to another new species described in the present study (see ‘Note’ under the account for *Jingophrys wularsoni* sp. nov.). Here, we report the discovery and formal descriptions of three new species of this species group from India. These additions restrict the currently recognized number of species in this group from India to the following three: *Jingophrys hrdy* sp. nov., *Jingophrys taniitatii* sp. nov. and *Jingophrys wularsoni* sp. nov. Additionally, we report an unnamed distant lineage *Jingophrys* cand. sp. B. from Northeast India, whose taxonomic status is pending future studies.

***Jingophrys hrdy* sp. nov.**

(http://zoobank.org/urn:lsid:zoobank.org:act:32DE21BE-A0B3-48B9-8FD8-E939264CFD2C)

figures 1–8, 15, 18 and 19; tables S1–S9 and figure S6 in the supplementary material

**Figure 18.**
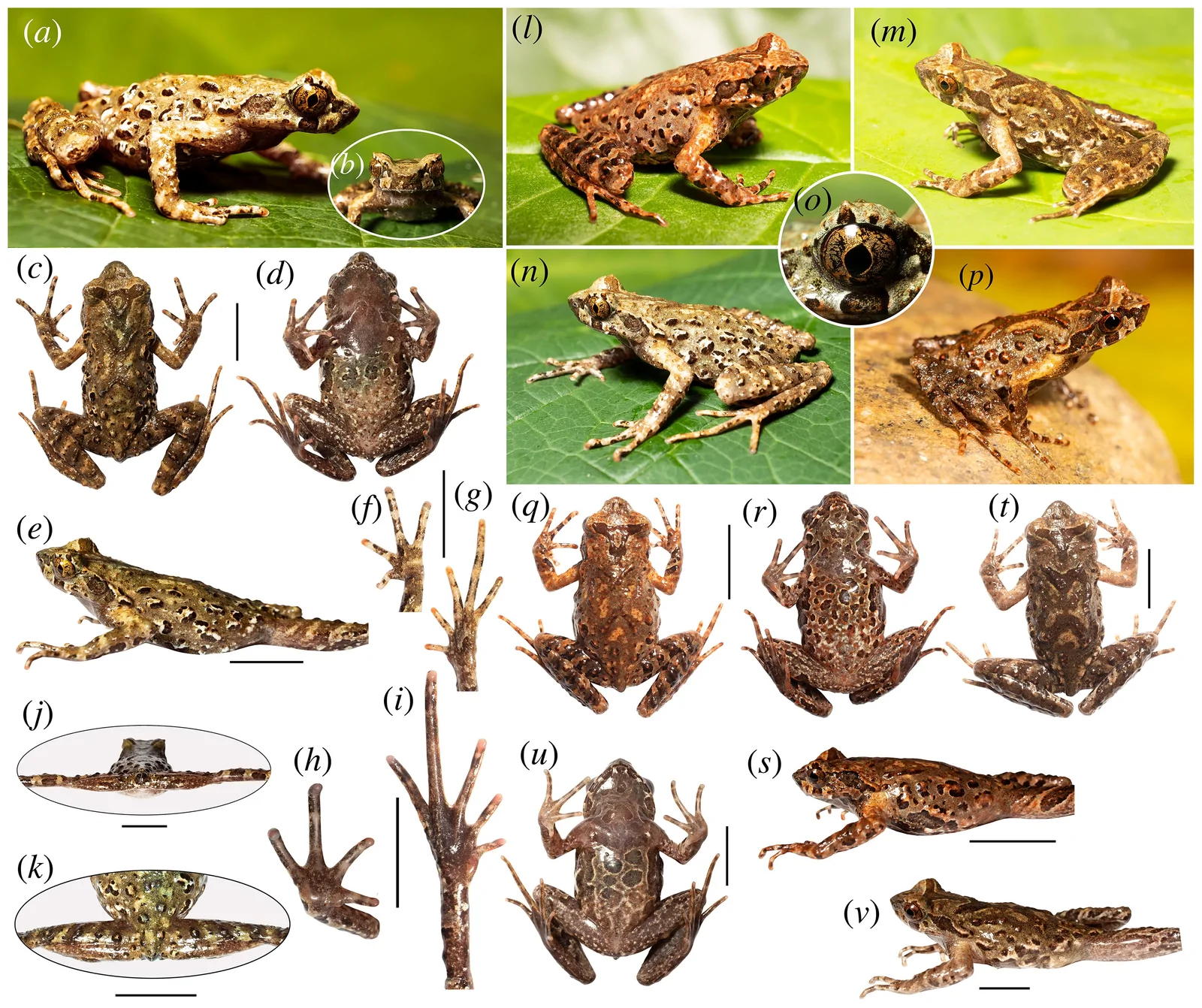
Types and referred specimens of *Jingophrys hrdy* sp. nov., in life. (*a–k*) Holotype (ZSI A 16624, adult male): (*a*) lateral view, (*b*) frontal view, (*c*) dorsal view, (*d*) ventral view, (*e*) lateral view of flank, groin and thigh, (*f*) dorsal view of hand, (*g*) dorsal view of foot, (*h*) ventral view hand, (*i*) ventral view of foot, (*j*) posterior view of thighs and (*k*) dorsal view of thighs; (*l, m, n, p*) three types and one referred specimen, dorsolateral view: ZSI A 16625 (male), ZSI A 16626 (male), ZSI A 16624 (male) and SDBDU 2021.5104 (subadult male), respectively; (*o*) enlarged view of upper eyelid showing a single small tubercle (ZSI A 16624, male); (*q–s*) ZSI A 16625: dorsal view, ventral view and lateral view of flank, groin and thigh, respectively; (*t–v*) ZSI A 16626: dorsal view, ventral view, and lateral view of flank, groin and thighs, respectively. Scale bars = 10 mm. Photographs: S. D. Biju and T. Tajo.

**Figure 19.**
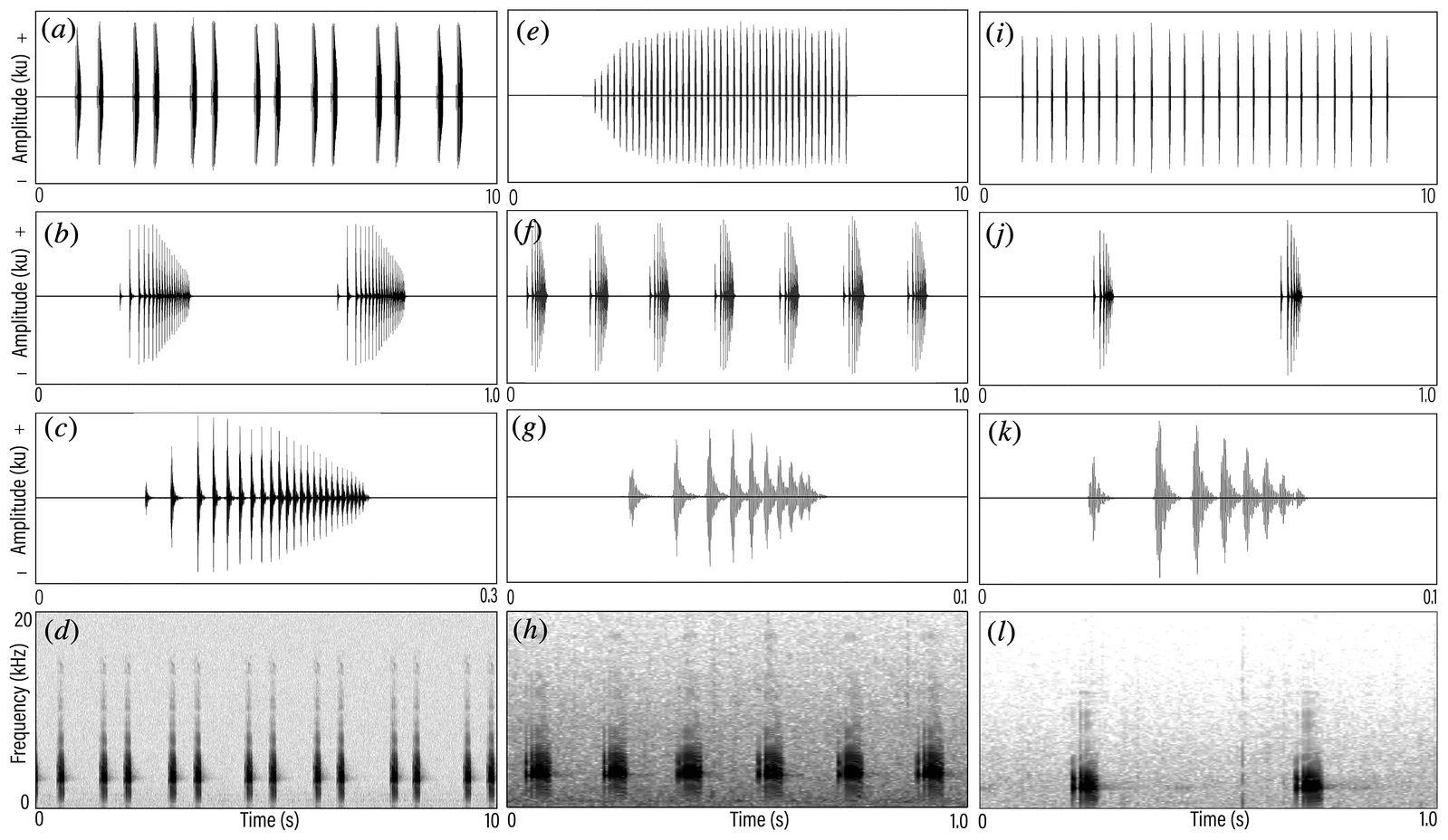
Acoustic properties of typical male calls of three species in the *Jingophrys pachyproctus* species group. (*a–d*) *Jingophrys hrdy* sp. nov.: (*a*) oscillogram for a 10-s call segment, (*b*) oscillogram for a 1.0-s call segment, (*c*) oscillogram for a 0.3-s call segment and (*d*) spectrogram for a 10-s call segment; (*e–h*) *Jingophrys taniitatii* sp. nov.: (*e*) oscillogram for a 10-s call segment, (*f*) oscillogram for a 1.0-s call segment, (*g*) oscillogram for a 0.1-s call segment and (*h*) spectrogram for a 1.0-s call segment; (*i–l*) *Jingophrys wularsoni* sp. nov.: (*i*) oscillogram for a 10-s call segment, (*j*) oscillogram for a 1.0-s call segment, (*k*) oscillogram for a 0.1-s call segment and (*l*) spectrogram for a 1.0-s call segment.

**Figure 20.**
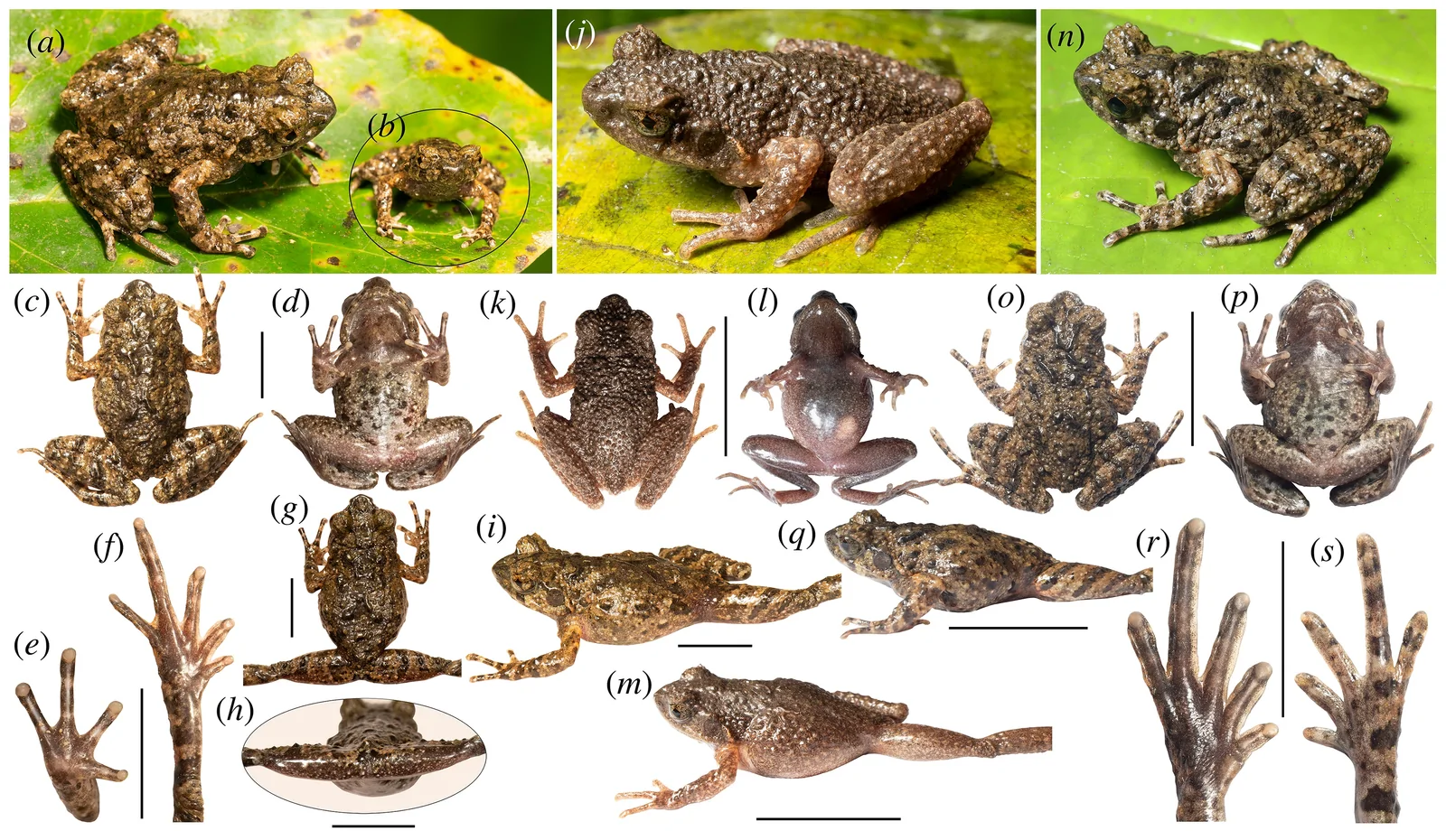
Type series of *Jingophrys taniitatii* sp. nov., in life. (*a–i*) Holotype (ZSI A 16628, adult male): (*a*) dorsolateral view, (*b*) frontal view, (*c*) dorsal view, (*d*) ventral view, (*e*) ventral view of hand, (*f*) ventral view of foot, (*g*) dorsal view of dorsum and thighs, (*h*) posterior view of thighs and (*i*) lateral view of flank, groin and thigh; (*j–m*) paratype (ZSI A 16630, adult male): (*j*) dorsolateral view, (*k*) dorsal view, (*l*) ventral view and (*m*) lateral view of flank, groin and thigh; (*n–s*) paratype (ZSI A 16629, adult male): (*n*) dorsolateral view, (*o*) dorsal view, (*p*) ventral view, (*q*) lateral view of flank, groin and thigh, (*r*) ventral view of foot and (*s*) dorsal view of foot. Scale bars = 10 mm. Photographs: S. D. Biju and T. Tajo.

**Holotype.** An adult male, ZSI A 16624 (SDB 2023.6471), from Tale Top (27.5338° N, 93.9539° E, 2344 m asl), Tale Valley Wildlife Sanctuary, Lower Subansiri District, Arunachal Pradesh State, India, collected by TT, RUK and SDB on 4 June 2023.

**Paratypes** **.** Three adult males, ZSI A 16625 (SDB 2023.6487), ZSI A 16626 (SDB 2023.6488) and ZSI A 16627 (SDB 2023.6490), collected with the holotype.

**Referred specimens.** An adult male, SDBDU 2023.6489, collected with the holotype; and an adult male, SDBDU 2023.6538, from Tasi Biddang (27.5406° N, 93.9167° E, 2121 m asl), Tale Valley WLS, Lower Subansiri District, Arunachal Pradesh State, India, collected by SDB, SG, TT and RUK on 8 September 2023.

**Etymology.** The species is named in honour of Dr Sarah Blaffer Hrdy (anthropologist and primatologist) and Dr Daniel Bruce Hrdy (medical professor) in appreciation of their long-time support for and commitment to the study of conservation biology. The specific epithet *hrdy* is treated as an invariable noun in apposition to the generic name.

**Suggested common name.** Hrdy's horned frog.

**Diagnosis.** *Jingophrys hrdy* sp. nov. can be distinguished from other known congeners based on a combination of characters that include prominently raised tubercles on the posterior dorsum and flanks; a distinct granular tubercle just behind the upper eyelid on either side of the dorsal surface of the head; supratympanic fold narrow anteriorly, curving posteriorly at the level of the axilla into an elongated tubercle; toes without lateral dermal fringes, tips rounded; and prominent blackish-brown blotches on belly.

**Phylogenetic relationships.** *Jingophrys hrdy* sp. nov. is a member of the *J. pachyproctus* species group. It has a sister-group relationship with the clade containing *Jingophrys taniitatii* sp. nov. and the unnamed *J*. cand. sp. B. (figures 1–3). Within its species group, *Jingophrys hrdy* sp. nov. shows the following DNA sequence divergence in the mitochondrial 16S rRNA gene: 7.5–7.9% from *J. pachyproctus*; 4.9–5.1% from *Jingophrys taniitatii* sp. nov.; 6.7–6.9% from *Jingophrys wularsoni* sp. nov.; and 5.5–5.7% from the undescribed candidate species *J*. cand. sp. B (supplementary material, table S3).

**Holotype description (measurements in mm).** Adult male (SVL 35.3), stout body; head moderately large, head length subequal to width (HL 12.4, HW 12.5, MN 10.1; MFE 8.3; MBE 4.7); snout nearly rounded in dorsal view, acutely protruding in lateral view, snout tip projecting far beyond the lower lip (SP 2.6 mm); loreal region nearly vertical, concave; canthus rostralis rounded; dorsal snout slightly concave; eye diameter larger than maximum tympanum diameter but smaller than snout length (EL 3.5, TYD 2.5, SL 5.0); tympanum-to-eye distance (TYE 1.5) shorter than maximum tympanum diameter (TYD 2.5); tympanum distinct, oval, dorsally compressed, upper border concealed by supratympanic ridge; pupil oval in preservative, oriented vertically; nostril oval, oriented laterally; nostril slightly further from eye than from snout (EN 2.3, NS 2.1); internarial distance greater than upper eyelid width and the narrowest point between upper eyelids (IN 3.9, UEW 3.2, IUE 3.6); distance between posterior margins of eyes (IBE 10.1) 1.7 times the distance between anterior margins of eyes (IFE 5.9); pineal ocellus not visible externally; vomerine ridges and teeth absent; maxillary teeth present; tongue large, rounded, with no medial lingual process. Forelimbs long; forearm shorter than hand (FAL 8.0, HAL 10.8); fingers moderately long, thin, dermal fringes absent, relative finger lengths I < II < IV < III (FIL 3.1, FIIL 4.9, FIIIL 7.6, FIVL 5.5); interdigital webbing, and subarticular and supernumerary tubercles absent; thenar and outer metacarpal tubercles weakly developed; fingertips rounded, raised ventrally, not expanded relative to digit widths, terminal grooves absent on fingertips. Hindlimbs long, thin; thight length subequal to both shank and foot lengths (TL 17.5, SHL 17.6, FOL 17.7); distance from heel to tip of toe IV (TFOL 27.6); toes without lateral fringes, relative toe lengths I < II < V < III < IV; tips not dilated, rounded, with distinct pads, raised ventrally, terminal grooves absent; base of toes with rudimentary webbing; outer metatarsal tubercle absent; subarticular and supernumerary tubercles absent; inner metatarsal tubercle present but barely distinguishable (figure 18).

*Skin texture of holotype*. Dorsal surface of head shagreened; dorsum sparsely granular, posterior part of dorsum and flanks with large raised tubercles; posterior parts of thighs with prominent large tubercles; upper eyelids shagreened with a tiny horn-like tubercle in the middle of the outer edge; a distinct granular tubercle just behind upper eyelids on either side of dorsal surface of head; tympanum smooth, borders slightly raised, surrounding area posterior to eye sparsely granular; well-developed supratympanic fold extends from posterior corner of eye to above forearm insertion, narrow anteriorly and widens posteriorly at the level of arm forming an elongated glandular tubercle; oval-shaped ridge at the centre of the triangular marking between eyes; parietoscapular-sacral ridges form a vertically oriented ‘> <’ configuration, the two arms of the first ‘V’-shaped ridge begin above the tympanum and extend posteriorly where they meet medially beyond the level of axilla, and the second inverted ‘V’-shaped ridge present at mid-dorsum; dorsolateral dermal ridges absent; dorsal surfaces of fore- and hindlimbs shagreened, shanks with prominent elongated tubercles corresponding with the transverse crossbands; dorsal surface of forearms granular; fingers and toes smooth. Ventral surfaces of body and limbs smooth; dermal asperities on lower jaw absent; snout tip projection beyond lower lip sparsely granular; pectoral glands small and raised, rounded, positioned at level with axilla; both sides of belly with tubercles arranged from below forearm insertion to near groin; femoral glands weakly developed, slightly raised, closer to outer edge of knee than to cloaca; tubercles on cloacal region (figure 8*h*,*s*).

*Colour of holotype*. *In life*. Dorsum and fore- and hindlimbs greyish brown with light greenish-yellow tinge; roughly ‘X’-shaped light brown blotch present on dorsum; inverted triangular marking between eyes brown, outlined with light grey; borders of dermal ridges and tubercles blackish brown with light reddish tinge; inner margin of supratympanic folds dark brown; anterior part of the snout dark brown; light brown band extends from anterior margin of loreal region and over entire tympanum with a break at the eye; lateral surfaces of head greyish brown; wide vertical dark brown bands along upper lips and lateral surfaces of head; thin dark brown bands on margins of upper eyelids; flanks lighter than dorsum, dark brown tubercles on margins of flanks; finger and toe tips light orangish red; iris reddish brown with golden tinge; dorsal surfaces of fore- and hindlimbs, including fingers and toes, with blackish-brown transverse crossbands that extend towards ventral surfaces. Ventral surfaces of throat and chest light reddish brown; gular region near chest with two large brown spots; discontinuous broad dark grey bands on either side of belly extend from forearm insertion to groin; belly light greyish brown with prominent blackish-brown blotches and greyish-white margins and spots; fore- and hindlimbs brown with prominent greyish-white marbling; anterior and posterior thighs light brown with prominent blackish-brown blotches; blackish-brown colouration around cloacal opening (figure 18). *In preservative*. Dorsum, limbs, lateral surfaces of head and flanks light greyish brown, with green tinge from head to mid-dorsum; inverted triangular marking between eyes light brown with grey central blotch; obscure light grey ‘X’-shaped marking on dorsum; all blackish-brown blotches turned to lighter greyish brown. Ventral surfaces of throat and chest light brown; belly light greyish brown; blotches light brown; hand and foot light greyish brown (supplementary material, figure S6).

**Morphological variation.** Morphometric variation among four types and two referred specimens (six adult males) is provided in table S5 in the supplementary material. Paratypes and referred specimens resemble the holotype in most morphological characters, with some exceptions. *Colour in life*: ZSI A 16625 (SDB 2023.6487)—dorsum light reddish brown with small red spots on granulations, ventral surfaces light reddish brown with dark brown blotches, and margins of upper and lower lips orangish red; ZSI A 16626 (SDB 2023.6488)—dorsum light brown with dark brown inverted triangular marking between eyes, and ventral surface of throat with prominent blackish-brown blotches with margins. *Skin texture*: ZSI A 16626 (SDB 2023.6488)—dorsum coarsely granular; ZSI A 16625 (SDB 2023.6487) and SDBDU 2023.6489—dorsolateral dermal ridge present, broken into shorter discontinuous blotches; SDBDU 2021.5104—dorsum reddish brown; borders of ridges and tubercles blackish brown with reddish tinge, and margins of supratympanic fold light reddish orange.

**Morphological comparison.** *Jingophrys hrdy* sp. nov. cannot be confused with two megophryid species whose phylogenetic placement within their currently assigned genus (or subgenus) remains unknown. It can be distinguished by the following characters: from *Xenophrys damrei* by its smaller adult male size, SVL 30.0–36.2 mm, *n =* 6 (versus larger, adult male SVL 47.7–57.1 mm, *n =* 7 [96,97]); and from *Ophryophryne pachyproctus* by relatively larger adult male size, SVL 30.0–36.2 mm, *n =* 6 (versus smaller, adult male SVL 28.0–30.0 mm), and presence of maxillary teeth (versus absent) [98].

Within the genus, *Jingophrys hrdy* sp. nov. differs from all currently recognized species (including six previously known and five newly described) by its anterior dorsum shagreened to sparsely granular and large raised tubercles on posterior half (versus dorsum shagreened to densely or sparsely granular with scattered tubercles, except in *Jingophrys taniitatii* sp. nov., which has densely granular tubercles and smooth warts); lateral surfaces of dorsum (including flanks) with large, raised tubercles (versus granular or less prominent tubercles, except in *Jingophrys taniitatii* sp. nov.); and supratympanic fold narrow anteriorly and curving posteriorly at the level of arm into an elongated tubercle (versus elongated tubercle absent). Specifically, *Jingophrys hrdy* sp. nov. differs from *Jingophrys taniitatii* sp. nov. by its larger adult male size, SVL 30.0–36.2 mm, *n =* 6 (versus smaller, male SVL 27.4–31.1 mm, *n =* 4); and the absence of lateral dermal fringes on toes (versus present, wide). *Jingophrys hrdy* sp. nov. differs from *J. feii* (based on [29]) by its larger adult male size, SVL 30.0–36.2 mm, *n =* 6 (versus smaller, SVL 24.3–25.1 mm, *n =* 4); foot length nearly equal to thigh length, FOL/TL 0.96–1.02 (versus foot shorter than thigh, FOL/TL 0.82–0.98); lateral dermal fringes on toes absent (versus present, moderate to wide); and ventral surfaces of body greyish brown with prominent blackish-brown blotches with greyish-white margins (versus white with distinct, irregular, small, scattered dark brown spots and flecks on the belly). *Jingophrys hrdy* sp. nov. differs from *J. pachyproctus* by its tympanum-to-eye distance shorter than tympanum diameter, TYE/TYD 0.60–0.73 (versus tympanum-to-eye distance nearly equal to tympanum diameter [30]); absence of vomerine teeth (versus present [male HT, NWIPB 770650 [31]); anterior dorsum shagreened, posterior part sparsely granular with large, raised tubercles (versus dorsum sparsely granular [male HT, NWIPB 770650 [31]); lateral surfaces of dorsum (including flanks) with large, raised tubercles (versus granular, with scattered tubercles [male HT, NWIPB 770650 [31]); and dorsolateral dermal ridges absent or present as short interrupted ridges on either side of anterior dorsum (versus continuous and uninterrupted dorsolateral dermal ridges from behind supratympanic fold to near groin on either side [male HT, NWIPB 770650 [31]). *Jingophrys hrdy* sp. nov. differs from *Jingophrys tanogogo* sp. nov. by its stout body (versus slender); relatively larger adult male size, SVL 30.0–36.2 mm, *n =* 6 (versus smaller, adult male SVL 24.9–30.3 mm, *n =* 8); snout nearly rounded in dorsal view (versus nearly bluntly pointed); tympanum-to-eye distance shorter than tympanum diameter, TYE/TYD 0.60–0.73 (versus larger, TYE/TYD 0.87–1.31); foot length nearly equal to thigh length and nearly equal or slightly shorter than shank length, FOL/TL 0.96–1.02 and FOL/ShL 0.92–1.01 (versus foot shorter than thigh and shank, FOL/TL 0.83–0.90 and FOL/ShL 0.80–0.88); lateral dermal fringes on toes absent (versus present, wide); dorsolateral dermal ridge absent or present as short interrupted ridges on either side of anterior dorsum (versus continuous and discontinuous dorsolateral dermal ridges from behind supratympanic fold to near groin on either side); finger and toe tips raised ventrally (versus not raised); and ventral surfaces of body greyish brown with prominent blackish-brown blotches with greyish-white margins (versus belly off-white or creamy white with broad dark grey bands on either side from forearm insertion to groin). *Jingophrys hrdy* sp. nov. differs from *J. vegrandis* by its relatively larger adult male size, SVL 30.0–36.2 mm, *n =* 6 (versus smaller, adult male SVL 26.8–29.9 mm, *n =* 6); FOL nearly equal to thigh length and nearly equal or slightly shorter than shank length, FOL/TL 0.96–1.02 and FOL/ShL 0.92–1.01 (versus foot shorter than thigh and shank, FOL/TL 0.83–0.92 and FOL/ShL 0.76–0.85); lateral dermal fringes on toes absent (versus present, moderate to wide); and dorsolateral dermal ridges absent or present as short interrupted ridges on either side of anterior dorsum (versus continuous or discontinuous dorsolateral dermal ridges from behind supratympanic fold to near groin on either side). *Jingophrys hrdy* sp. nov. differs from *Jingophrys wularsoni* sp. nov. by its relatively smaller adult male size, SVL 30.0–36.2 mm, *n =* 6 (versus larger, 36.4–40.8 mm, *n =* 9); tympanum-to-eye distance shorter than tympanum diameter, TYE/TYD 0.60–0.73 (versus wider, TYE/TYD 1.26–1.56); and foot length nearly equal to thigh length and nearly equal or slightly shorter than shank length, FOL/TL 0.96–1.02 and FOL/ShL 0.92–1.01 (versus foot shorter than thigh and shank, FOL/TL 0.87–0.96 and FOL/ShL 0.86–0.94). *Jingophrys hrdy* sp. nov. differs from *J. yeae* by its relatively larger adult male size, SVL 30.0–36.2 mm, *n =* 6 (versus smaller, 24.9–28.0 mm, *n =* 7 [23.8–29.1 mm, *n =* 12, in Chinese population [31]); internarial distance nearly equal or shorter than distance between upper eyelids, IN/IUE 0.89–1.08 (versus much shorter, IN/IUE 0.76–0.86); foot length nearly equal or slightly shorter than shank length, FOL/ShL 0.92–1.01 (versus foot shorter than shank, FOL/ShL 0.82–0.92); and lateral dermal fringes on toes absent (versus present, weakly developed). *Jingophrys hrdy* sp. nov. differs from *J. zhaoermii* by its larger adult male size, SVL 30.0–36.2 mm, *n =* 6 (versus smaller, adult male SVL 22.5–26.5 mm, *n =* 3); head nearly as long as wide, HL/HW 0.99–1.04 (versus head longer than wide, HL/HW 1.14–1.19); dorsolateral dermal ridges absent or present as short interrupted ridges on either side of anterior dorsum (versus continuous dorsolateral dermal ridges from behind supratympanic fold to near groin on either side); and ventral surfaces of body greyish brown with prominent blackish-brown blotches that have greyish-white margins (versus belly with grey mottling with purplish-grey hue, scattered with small white blotches). *Jingophrys hrdy* sp. nov. differs from *J. zhoui* by its larger adult male size, SVL 30.0–36.2 mm, *n =* 6 (versus smaller, 23.0 mm, *n =* 1 [31]); head nearly as long as wide, HL/HW 0.99–1.04 (versus head longer than wide [31]); dorsolateral dermal ridges absent or present as short interrupted ridges on either side of anterior dorsum (versus continuous dorsolateral dermal ridges from behind supratympanic fold to near groin on either side [31]); and ventral surfaces of body greyish brown with prominent blackish-brown blotches having greyish-white margins (versus abdomen orange-brown, stained with several faint orange dots; lower abdomen white and scattered with several clean-orange dots [31]). For additional comparisons, see the ‘Morphological comparisons’ sections for the respective species.

**Secondary sexual characters.** Males: nuptial pads or spines not visible other than a swelling on finger I; and a single subgular vocal sac while calling.

**Vocalization** **.** Calls of male *Jingophrys hrdy* sp. nov. were recorded at 19.00 hours on 4 June 2023 at Tale Top (Tale Valley WLS), Lower Subansiri District, Arunachal Pradesh. Ambient temperatures were 17.8°C dry bulb and 14.8°C wet bulb. The species is cathemeral, and individuals were active and observed calling at irregular intervals throughout the day and night, between 10.00 and 20.00 hours. Based on analyses of 50 calls from a single individual, the typical vocal repertoire of this species is as follows: males produce a single type of call that has a pulsatile temporal structure; calls are not delivered in groups; the mean call duration is 180.2 ms; the amplitude envelope has a mean rise time of 57.4 ms and a mean fall time of 122.5 ms; calls deliver 22–23 pulses at a mean rate of 127.48 pulses/s; and the call spectrum shows three broad peaks with a mean dominant frequency of 3.23 kHz (figure 19*a*–*d*; supplementary material, table S7).

**Call comparison.** Calls of *Jingophrys hrdy* sp. nov. can be differentiated from those of other congeners primarily by their frequency, pulses per call, pulse rate, duration and rise time, as follows: (i) *Call frequency*: *Jingophrys hrdy* sp. nov. calls differ from those of *Jingophrys wularsoni* sp. nov. by their higher frequency, 3.23 kHz (versus lower, 2.7 kHz); and from those of *Jingophrys siangensis* sp. nov., *Jingophrys tanogogo* sp. nov., *Jingophrys taniitatii* sp. nov., *J. vegrandis*, *J.* cf. *yeae* and *J.* cf. *zhaoermii* by their lower frequency, 3.23 kHz (versus higher, 4.43, 4.88, 3.59, 5.46, 4.61 and 4.57 kHz, respectively). The dominant frequency of *Jingophrys hrdy* sp. nov. (3.14–3.36 kHz) falls within the range of *Jingophrys taniitatii* sp. nov. (3.45–3.75 kHz), but *Jingophrys hrdy* sp. nov. calls can be differentiated by other acoustic properties such as more pulses per call, slower pulse rate and longer rise time. (ii) *Pulses per call*: Although number of pulses per call can be a dynamic character, *Jingophrys hrdy* sp. nov. calls had considerably more pulses per call (22–27) among all studied congeners (versus lower, 10–15 pulses in *Jingophrys siangensis* sp. nov., 5–6 pulses in *Jingophrys tanogogo* sp. nov., 9–11 pulses in *Jingophrys taniitatii* sp. nov., 11–14 pulses in *J. vegrandis*, 7–9 pulses in *Jingophrys wularsoni* sp. nov., 11–13 pulses in *J.* cf. *yeae* and 8–10 pulses in *J.* cf. *zhaoermii*). (iii) *Pulse rate*: *Jingophrys hrdy* sp. nov. calls differ from those of *J. vegrandis*, *J.* cf. *yeae* and *J.* cf. *zhaoermii* by their faster pulse rate, 127.48 pulses/s (versus slower, 71.03, 102.55 and 99.46 pulses/s, respectively); and from those of *Jingophrys siangensis* sp. nov., *Jingophrys tanogogo* sp. nov., *Jingophrys taniitatii* sp. nov. and *Jingophrys wularsoni* sp. nov. by their slower pulse rate, 127.48 pulses/s (versus faster, 193.66, 140.98, 184.13 and 140.8 pulses/s, respectively). (iv) *Call duration*: *Jingophrys hrdy* sp. nov. calls have the longest duration (180.2 ms) among all studied congeners (versus shorter, 59.7 ms in *Jingophrys siangensis* sp. nov., 31.1 ms in *Jingophrys tanogogo* sp. nov., 54.5 ms in *Jingophrys taniitatii* sp. nov., 166.6 ms in *J. vegrandis*, 51.7 ms in *Jingophrys wularsoni* sp. nov., 109.4 ms in *J.* cf. *yeae* and 78.9 ms in *J.* cf. *zhaoermii*). (v) *Call rise time*: *Jingophrys hrdy* sp. nov. calls differ from those of *Jingophrys siangensis* sp. nov., *Jingophrys tanogogo* sp. nov., *Jingophrys taniitatii* sp. nov., *Jingophrys wularsoni* sp. nov., *J.* cf. *yeae* and *J.* cf. *zhaoermiii* by their longer rise time, 57.4 ms (versus shorter, 21.5, 14.7, 23.3, 15.6, 39.2 and 31.6 ms, respectively); and from those of *J. vegrandis* by their shorter rise time, 57.4 ms (versus relatively longer, 59.7 ms) (figure 19; supplementary material, table S7).

Additionally, we compared vocalizations of *Jingophrys hrdy* sp. nov. with those published for three members in the People's Republic of China [29,31]. Calls of *Jingophrys hrdy* sp. nov. differ from those of *J. feii* and *J. yeae* by their lower dominant frequency, 3.23 kHz (versus higher, 4.74–4.91 kHz and 4.4–5.2 kHz, respectively); longer call duration, 180.2 ms (versus shorter, 132.6 ms and 139 ms, respectively); and more pulses per call, 22–27 (versus relatively fewer, 14–29 and 7–12 pulses per call, respectively). *Jingophrys hrdy* sp. nov. calls have a similar dominant frequency (3.23 kHz) as those of *J.* cf. *pachyproctus* (3.2 kHz) but differ by their shorter duration, 180.2 ms (versus longer, 746 ms), and fewer pulses per call, 22–27 (versus more, 22–62 pulses per call) (supplementary material, table S7).

**Tadpole.** *External morphology*: the following description is based on a single Gosner stage 35 tadpole, SDBDU 2023.6535: body cylindrical with height (BH 5.0) 90% of body width (BW 5.6); body length (BL 11.0) 30% of total length (TL 35.5); in dorsal view body broader (BW 5.6) than tail (TMW 3.5, TMW 62% of BW), horizontally elliptical, and with maximum body width at abdominal region; snout truncate in lateral view, the profile interrupted by an anterodorsally positioned oral disc; narial opening oval, anterolaterally positioned, with serrated rim and no projection; nares closer to snout (SN 1.0) than eyes (NE 1.9) (SN 58% of NE); eyes medium-sized (ED 1.3) and positioned dorsolaterally; internarial distance (IND 2.9) 68% of interorbital distance (IOD 4.2); spiracle opens sinistrally at level of hindlimbs, immediately caudal to the gills laterally; snout-to-spiracle distance (SS 6.3) 57% of body length (BL 11.0); spiracular aperture smaller than spiracular tube, directed laterally, with tip of inner wall of spiracular aperture free from body wall; vent tube opens medially at ventral end of body terminus, its dorsal wall medially attached to ventral fin; tail long (TAL 24.6) 69% of total length (TL 35.5), heavily muscularized (TMH 3.8, 79% of MTH 4.8, and TMW 3.5, 62% of BW 5.6); upper fin originates after body-tail junction (SU 14.5), lower in height at proximal end; lower fin originates after vent tube; both fins of relative low height, upper fin higher than lower fin (UFH 1.9, 40% of MTH 4.8, LFH 1.5, 32% of MTH 4.8); each fin reaches maximum height at mid-region of tail and gradually tapers into tail tip; tail muscle shows maximum height from body-tail junction to mid-region and then gradually tapers and ends before tail tip; tail tip pointed (figure 15*c*). Oral disc umbelliform, anterodorsally positioned, horizontally elliptical, wider (ODW 6.5) than body width (BW 5.6) (BW 86% of ODW), and lacking emarginations laterally; oral disc folds inward when not fully extended and in preservative; the edges of lip wavy and without marginal papillae; submarginal papillae on oral disc arranged in four to five rows in ventral view, oriented vaguely parallel to transverse axis of oral disc, as well as in radiating pattern extending from inner region of oral cavity; about four rows of submarginal papillae arranged vaguely in parallel on dorsal side of funnel; submarginal papillae pustule-like, larger-sized and oval-shaped in area close to jaw sheath, gradually reduce in size and become more circular towards the outer margin of oral disc; a single long ridge-like papilla located closer to oral cavity laterally on both sides of oral disc; submarginal papillae on dorsal side smaller than those on ventral side; tooth rows absent; jaw sheaths feebly keratinized; upper jaw sheath almost in straight line shape with a median notch; lower jaw sheath almost in straight line shape (figure 15*c*).

*Colour in life*. Overall colour of tadpole body and tail light brown with a shade of dark brown, dark brown shading mainly present on lateral and dorsal body surfaces, and outer margin of tail muscle, while the remaining tail muscle and most part of tail fin dusted with dark melanocytes; tail fin mostly translucent; golden iridocytes widely distributed throughout body and randomly dusted throughout tail; gills visible as red structures on ventral body surface; abdominal area transparent, gut coil partially visible; eyes black with few golden iridocytes scattered on outer margin of sclera form orange ring around pupil; umbelliform oral disc with dark brown pigmentation laterally and on submarginal papillae (figure 15*c*).

**Distribution.** *Jingophrys hrdy* sp. nov. is currently known only from two sites, Tale Top and Tasi Biidang in the Tale Valley WLS, Lower Subansiri District, Arunachal Pradesh State, India, at elevations ranging from 2121 to 2344 m asl (figure 6; supplementary material, table S2).

**Habitat and natural history** **.** The habitat of *Jingophrys hrdy* sp. nov. is confined to higher elevation forests sparsely covered with montane wet temperate vegetation and therefore has much less canopy cover than forests at lower elevations. Tale Top is a high-elevation valley surrounded by mountains on all sides and the specific site has a stream approximately 7–8 m wide. The type locality is a bamboo patch with scattered trees and a small water channel that meets the larger stream. Most individuals were collected during June and July 2023. This is the only *Jingophrys* species reported from Tale Top. All calling individuals were found perched on bamboo leaves and stems. Calling height ranged from 1 to 1.5 m above-ground. This is the only species of *Jingophrys* from Tale Valley WLS that calls actively both during the day and night with equal intensity, but calling reduces and eventually stops after 22.00 hours. During the non-breeding season (October 2022), a single individual at Tasi Biidang was found sitting on a shrub leaf approximately 2 m from a stream. It was sympatric with *Jingophrys wularsoni* sp. nov. at this site*.* No calling individuals were observed during subsequent surveys in early August 2023 at both Tasi Biidang and Tale Top, but tadpoles were collected from Tale Top.

***Jingophrys taniitatii* sp. nov.**

(http://zoobank.org/urn:lsid:zoobank.org:act:F639055D-DA15-4542-8219-1E87CB68CCD9)

figures 1–8, 19 and 20; tables S1–S9 and figure S7 in the supplementary material

**Holotype.** An adult male, ZSI A 16628 (SDB 2023.4488), from Tarin Tibe Khole Pass (27.5396° N, 93.8805° E, 1945 m asl), Tale Valley Wildlife Sanctuary, Lower Subansiri District, Arunachal Pradesh State, India, collected by SDB, SG, RUK and TT on 17 July 2023.

**Paratypes.** An adult male, ZSI A 16629 (SDB 2022.6021), collected from the same locality as the holotype by RUK, TT, SG and SDB on 16 June 2022; an adult male, ZSI A 16630 (SDB 2023.6417), from Tajgi Village (27.6402° N, 93.8638° E, 1740 m asl), Ziro Valley, Lower Subansiri District, Arunachal Pradesh State, India, collected by TT, RUK, SG and SDB on 14 May 2023.

**Referred specimens.** An adult male, SDBDU 2024.6616, from Reru Moreh, Ziro Valley, Lower Subansiri District, Arunachal Pradesh State, India, collected by TT, RUK and SDB on 30 June 2024.

**Etymology.** The species name is derived from a combination of two words: ‘tanii’, in honour of the Tanii tribe of Arunachal Pradesh State, India, who live in the Ziro Valley region where the type series was collected, and ‘tatii’, the Apatani language word for frogs and toads. The authors recognize the tremendous field support and guidance received from members of the tribal community during their studies. The specific epithet *taniitatii* is treated as an invariable noun in apposition to the generic name.

**Suggested common name.** Tanii horned frog.

**Diagnosis.** *Jingophrys taniitatii* sp. nov. is the most distinctive species of the genus and cannot be confused with other known congeners based on a combination of characters that include its largely uniform greyish-brown dorsum without contrasting colouration and markings; dorsal skin with prominent granular tubercles and smooth warts of various sizes; and the absence of both dorsolateral dermal ridges and parietoscapular-sacral ridges.

**Phylogenetic relationships.** *Jingophrys taniitatii* sp. nov. is a member of the *J. pachyproctus* species group and has a sister-group relationship with the unnamed *J*. cand. sp. B.; the two are closely related to *Jingophrys hrdy* sp. nov. (figures 1–3). Within its species group, *Jingophrys taniitatii* sp. nov. shows the following DNA sequence divergence in the mitochondrial 16S rRNA gene: 4.9–5.1% from *Jingophrys hrdy* sp. nov.; 8.3–8.7% from *J. pachyproctus*; 8.3% from *Jingophrys wularsoni* sp. nov.; and 4.4% from the undescribed candidate species *J*. cand. sp. B. (supplementary material, table S3).

**Holotype description (measurements in mm).** Adult male (SVL 29.0), stout body; head moderately large, wider than long (HL 11.3, HW 11.9, MN 9.2; MFE 8.0; MBE 4.3); snout nearly bluntly pointed in dorsal and ventral views, acutely protruding in lateral view, snout tip projecting far beyond lower lip (SP 2.5); loreal region acute, concave; canthus rostralis sharp (angled); dorsal snout concave; eye diameter larger than maximum tympanum diameter and equal to snout length (EL 3.8, TYD 1.8, SL 3.8); tympanum-to-eye distance (TYE 1.7) subequal to maximum tympanum diameter (TYD 1.8); tympanum distinct, rounded, upper border not concealed by supratympanic ridge; pupil oval in preservative, oriented vertically; nostril oval, oriented laterally, closer to eye than to snout (EN 1.5, NS 2.0); internarial distance greater than upper eyelid width and narrowest point between upper eyelids (IN 3.7, UEW 3.2, IUE 3.4); distance between posterior margins of eyes (IBE 10.1) 1.7 times the distance between anterior margins of eyes (IFE 6.1); pineal ocellus absent; vomerine ridges and teeth absent; maxillary teeth present; tongue moderately large, rounded, with no medial lingual process. Forelimbs long, thin; forearm shorter than hand (FAL 7.1, HAL 9.1); fingers moderately long, thin, dermal fringes absent, relative finger lengths I < II < IV < III (FIL 2.8, FIIL 3.7, FIIIL 5.9, FIVL 3.9); interdigital webbing, subarticular and supernumerary tubercles absent; thenar and outer metacarpal tubercles weakly developed; fingertips rounded and raised ventrally, not expanded relative to digit widths, with small oval pads, terminal grooves absent on fingertips. Hindlimbs long, thin; thigh shorter than shank, and longer than foot (TL 15.4, SHL 16.0, FOL13.6); distance from heel to tip of toe IV (TFOL 21.6); toes short and flattened with extensive broad lateral fringes that extend up to the tips of all digits, relative toe lengths I < II < V < III < IV; tips not dilated, but with distinct raised pads on ventral side; terminal grooves absent on toe tips; base of toes with rudimentary webbing; outer metatarsal tubercle absent; subarticular and supernumerary tubercles absent; inner metatarsal tubercle present but barely distinguishable (figure 20).

*Skin texture of holotype*. Dorsal surfaces of head, body, upper eyelids, flanks, posterior parts of lateral head surfaces, flanks and limbs (except fingers and toes) with smooth tubercles and warts of various sizes; posterior parts of dorsum more densely tuberculated; canthal regions with spinules; tympanum smooth with scattered spinules, borders slightly raised, area between tympanum and eye granular; supratympanic fold well developed, extends from posterior corner of eye to above forearm insertion, narrow without widening posteriorly; prominent tubercle on outer edge of each upper eyelid, without a horn-like tubercle in the middle; dorsolateral dermal ridges and parietoscapular-sacral ridges absent. Ventral surfaces of body and limbs smooth, weakly granular projections towards belly margins; small dermal asperities on margins of upper and lower jaws; pectoral glands small and raised, rounded, positioned at level of axilla; belly with tubercles on each side arranged from below forearm insertion to near groin; femoral glands not visible; anterior and posterior thigh granular; prominent tubercles on cloacal region (figure 8*j*,*u*).

*Colour of holotype*. *In life*. Dorsum, limbs, lateral surfaces of head, and belly greyish brown, lighter on lateral surface of belly; warts and surrounding regions blackish brown; faint band on loreal and tympanic region on either side; entire tympanum blackish brown; margins of upper lips, and finger and toe tips without any contrasting colouration; anterior snout mottled with blackish brown; wide oblique dark brown bar below eyes; iris reddish brown with golden tinge; limbs including fingers and toes with greyish-brown crossbands, prominent warts on thigh and shank corresponding with the crossbands. Ventral surface of lower jaw margins with faint alternating light brown and light grey bands; throat and chest light brown with obscure blackish-brown blotches; anterior belly lighter than throat; posterior belly light grey with prominent blackish-brown blotches intermingled with dark grey spots; anterior and posterior thigh light brown with greyish spots; blackish-brown colouration around cloacal opening; tips of fingers and toes, and margins of toes, without contrasting colouration (figure 20). *In preservative*. Dorsum uniformly dark ash grey; limbs light grey with blackish-brown crossbands. Ventral surface of throat light grey with minute blackish-brown spots; belly light grey with black spots; lateral surfaces of thigh and shank greyish white with crossbands (supplementary material, figure S7).

**Morphological variation.** Morphometric variation among three types and one referred specimen (four adult males) is provided in table S5 in the supplementary material. Paratypes and referred specimen resemble the holotype in most morphological characters, with some exceptions. *Colour in life*: ZSI A 16629 (SDB 2022.6021)—similar to the holotype; ZSI A 16630 (SDB 2023.6417)—dorsum uniformly light brown, crossbands on limbs faint, ventral surfaces of throat and chest blackish brown, and belly and limbs light reddish brown with faint dark grey reticulation forming a mosaic pattern. *Skin texture*: ZSI A 16629 (SDB 2022.6021)—similar to the holotype; ZSI A 16630 (SDB 2023.6417)—more prominently granular tubercles with smooth warts (figure 20).

**Morphological comparison.** *Jingophrys taniitatii* sp. nov. differs from all members of the subfamily Megophryinae by the combination of its small adult male size, SVL 27.4–31.1 mm, *n =* 4; dorsal skin with prominently smooth tubercles and warts of various sizes; absence of dorsolateral dermal ridges and parietoscapular-sacral ridges; and well-developed lateral fringes on all toes.

*Jingophrys taniitatii* sp. nov. cannot be confused with two megophryid species whose phylogenetic placement within their currently assigned genus (or subgenus) remains unknown. It can be distinguished by the following characters: from *Xenophrys damrei* by its much smaller adult male size, SVL 27.4–31.1 mm, *n =* 4 (versus larger, adult male SVL 47.7–57.1 mm, *n =* 7 [96,97]); and from *Ophryophryne pachyproctus* by the presence of maxillary teeth (versus absent), and the absence of a horn-like tubercle on the outer margin of upper eyelid (versus single horn-like tubercle) [98].

Within the genus, *Jingophrys taniitatii* sp. nov. differs from all currently recognized species (including six previously known and five newly described) by its dorsal skin with uniformly prominent smooth tubercles and granular warts (versus absence of granular warts) and the absence of dorsolateral dermal ridges and parietoscapular-sacral ridges (versus present). In addition, *Jingophrys taniitatii* sp. nov. also differs from *J. feii* by its larger adult male size, SVL 27.4–31.1 mm, *n =* 4 (versus smaller, 24.3–25.1 mm, *n =* 4); head wider than long, HW/HL 1.05–1.15, HW 11.5 ± 0.4 mm versus HL 10.7 ± 0.5 mm, *n =* 4 (versus approximately as wide as long [29]). *Jingophrys taniitatii* sp. nov. also differs from *Jingophrys hrdy* sp. nov. by its smaller adult male size, SVL 27.4–31.1 mm, *n =* 4 (versus larger, 30.0–36.2 mm, *n =* 5); head wider than long, HW/HL 1.05–1.15 (versus nearly as wide and long, HW/HL 0.97–1.01); snout length nearly equal or slightly longer than horizontal diameter of eye, SL/EL 1–1.14 (versus snout much longer than horizontal diameter of eye, SL/EL 1.19–1.43); thigh shorter than shank, TL/ShL 0.88–0.96 (versus thigh nearly equal to shank, TL/ShL 0.96–0.99); foot shorter than thigh and shank lengths, FOL/TL 0.88–0.93 and FOL/ShL 0.78–0.88 (versus foot nearly as long as thigh and shank, FOL/TL 0.96–1.02 and FOL/ShL 0.92–1.01); snout nearly bluntly pointed in dorsal view (versus rounded); tympanum rounded (versus oval, dorsally compressed); canthus rostralis sharp, angled (versus rounded); and presence of well-developed lateral fringes on all toes (versus weak or absent). Specifically, *Jingophrys taniitatii* sp. nov. also differs from *J. pachyproctus* by its smaller adult male size, SVL 27.4–31.1 mm, *n =* 4 (versus larger, 33.0–37.0 mm, *n =* 5); vomerine teeth absent (versus present); and well-developed lateral fringes on all toes (versus absent). *Jingophrys taniitatii* sp. nov. also differs from *Jingophrys tanogogo* sp. nov. by its stout body (versus slender); upper eyelid width nearly equal to narrowest point between upper eyelids, IUE/UEW 0.91–1.06 (versus distance between upper eyelids wider than upper eyelid width, IUE/UEW 1.33–1.55); tympanum rounded (versus oval, dorsally compressed); snout acutely protruding in lateral view (versus strongly acutely protruding); finger and toe tips raised ventrally (versus not raised); greyish band on lateral sides of head absent (versus present); and the absence of contrasting colouration on tips of fingers and toes, and margins of upper and lower lips (versus reddish-orange colouration on tips of fingers and toes and on margins of upper and lower lip). *Jingophrys taniitatii* sp. nov. also differs from *J. vegrandis* by its head wider than long, HW/HL 1.05–1.15 (versus head nearly as wide as long, HW/HL 0.97–1.01); ventral surface of thighs and posterior parts of hindlimb immaculate (versus mottled with dark brown); and the absence of contrasting colouration on tips of fingers and toes and on margins of upper and lower lips (versus reddish-orange colouration on tips of fingers and toes and on margins of upper and lower lips). *Jingophrys taniitatii* sp. nov. also differs from *Jingophrys wularsoni* sp. nov. by its smaller adult male size, SVL 27.4–31.1 mm, *n =* 4 (versus larger, 36.4–40.8 mm, *n =* 9); snout length nearly equal or slightly longer than horizontal diameter of eye, SL/EL 1–1.14 (versus snout much longer than horizontal diameter of eye, SL/EL 1.32–1.57); distance between eye and tympanum shorter or subequal to tympanum diameter, TYE/TYD 0.81–0.94 (versus wider, TYE/TYD 1.26–1.56); upper eyelid width nearly equal to narrowest point between upper eyelids, IUE/UEW 0.91–1.06 (versus distance between upper eyelids wider than upper eyelid width, IUE/UEW 1.27–1.47); thigh shorter than shank, TL/ShL 0.88–0.96 (versus thigh nearly equal to shank, TL/ShL 0.98–1.05); absence of tiny horn-like tubercle on outer edge of upper eyelids (versus horn-like tubercle at centre of upper eyelid margins); and well-developed lateral fringes on all toes (versus absent). *Jingophrys taniitatii* sp. nov. also differs from *J. yeae* by its head wider than long, HW/HL 1.05–1.15 (versus nearly as wide as long, HW/HL 0.96–1.05); snout length nearly equal or slightly longer than horizontal diameter of eye, SL/EL 1–1.14 (versus snout longer than horizontal diameter of eye, SL/EL 1.21–1.42); upper eyelid width nearly equal to narrowest point between upper eyelids, UEW/IUE 0.94–1.09 (versus upper eyelid width narrower than distance between upper eyelids, UEW/IUE 0.68–0.76); and snout nearly bluntly pointed in dorsal view (versus rounded). *Jingophrys taniitatii* sp. nov. also differs from *J. zhaoermii* by its larger adult male size, SVL 27.4–31.1 mm, *n =* 4 (versus smaller, 22.5–26.5 mm, *n =* 3); snout nearly bluntly pointed in dorsal view (versus rounded); head wider than long, HW/HL 1.05–1.15 (versus head longer than wide, HW/HL 0.84–0.88); snout length nearly equal or slightly longer than horizontal diameter of eye, SL/EL 1–1.14 (versus snout longer than horizontal diameter of eye, SL/EL 1.25–1.29); upper eyelid width nearly equal to narrowest point between upper eyelids, UEW/IUE 0.94–1.09 (versus upper eyelid width narrower than distance between upper eyelids, UEW/IUE 0.64–0.70); and well-developed lateral fringes on all toes (versus weakly developed or absent). *Jingophrys taniitatii* sp. nov. also differs from *J. zhoui* [31] by its larger adult male size, SVL 27.4–31.1 mm, *n =* 4 (versus smaller, 23.0 mm, *n =* 1); dorsal colour in life light or dark blackish brown without contrasting colouration (versus dorsum and limbs orange-brown, granules on body orange red); and well-developed lateral fringes on all toes (versus weakly developed). For additional comparisons, see the ‘Morphological comparisons’ sections for the respective species.

**Secondary sexual characters.** Males: nuptial pad or spines not visible; and a single subgular vocal sac while calling.

**Vocalization** **.** Calls of a male *Jingophrys taniitatii* sp. nov. were recorded at 21.40 hours on 16 June 2022 at Tarin Tibe Khole Pass (Tale Valley WLS), Lower Subansiri District, Arunachal Pradesh. A second individual was recorded at 20.00 hours on 14 May 2023 at Tajgi Village, Ziro Valley, Lower Subansiri District, Arunachal Pradesh. Ambient temperatures at the two sites were 19.5°C and 17°C dry bulb, and 17.8°C and 15°C wet bulb, respectively. Based on analyses of 50 calls from the two individuals, the typical vocal repertoire of this species is as follows: males produce a single type of call that has a pulsatile temporal structure; calls are not delivered in groups; the mean call duration is 54.5 ms; the amplitude envelope has a mean rise time of 23.3 ms and a mean fall time of 31.2 ms; calls deliver 9–11 pulses at a mean rate of 184.13 pulses/s; and the call spectrum shows three broad peaks with a mean dominant frequency of 3.59 kHz (figure 19*e*–*h*; supplementary material, table S7).

**Call comparison.** Calls of *Jingophrys taniitatii* sp. nov. can be differentiated from those of congeners primarily by their frequency, pulses per call, pulse rate, duration and rise time, and especially from the calls of seven species from India by their dominant frequency and pulse rate, as follows: (i) *Call frequency*: *Jingophrys taniitatii* sp. nov. calls differ from those of *Jingophrys hrdy* sp. nov. and *Jingophrys wularsoni* sp. nov. by their higher frequency, 3.59 kHz (versus lower, 3.23 and 2.70 kHz, respectively); and from those of *Jingophrys siangensis* sp. nov., *Jingophrys tanogogo* sp. nov., *J. vegrandis*, *J.* cf. *yeae* and *J.* cf. *zhaoermii* by their lower frequency, 3.59 kHz (versus higher, 4.43, 4.88, 5.46, 4.61 and 4.57 kHz, respectively). (ii) *Pulse rate*: *Jingophrys taniitatii* sp. nov. calls differ from those of *Jingophrys siangensis* sp. nov. by their slower pulse rate, 184.13 pulses/s (versus faster, 193.66 pulses/s); and from rest of the studied congeners by their faster pulse rate, 184.13 pulses/s (versus slower, 127.48 pulses/s in *Jingophrys hrdy* sp. nov., 140.98 pulses/s in *Jingophrys tanogogo* sp. nov., 71.03 pulses/s in *J. vegrandis*, 140.8 pulses/s in *Jingophrys wularsoni* sp. nov., 102.55 pulses/s in *J.* cf. *yeae* and 99.46 pulses/s in *J.* cf. *zhaoermii*). (iii) *Call duration*: *Jingophrys taniitatii* sp. nov. calls differ from those of *Jingophrys tanogogo* sp. nov. and *Jingophrys wularsoni* sp. nov. by their longer duration, 54.5 ms (versus shorter, 31.1 and 51.7 ms, respectively); and from those of *Jingophrys hrdy* sp. nov., *Jingophrys siangensis* sp. nov., *J. vegrandis*, *J.* cf. *yeae* and *J.* cf. *zhaoermii* by their shorter duration, 54.9 ms (versus longer, 180.2, 59.7, 166.6, 109.4 and 78.9 ms, respectively). (iv) *Call rise time*: *Jingophrys taniitatii* sp. nov. calls differ from those of *Jingophrys siangensis* sp. nov., *Jingophrys tanogogo* sp. nov. and *Jingophrys wularsoni* sp. nov. by their longer rise time, 23.3 ms (versus shorter, 21.5, 15.6 and 14.7 ms, respectively); and from those of *Jingophrys hrdy* sp. nov., *J. vegrandis*, *J.* cf. *yeae* and *J.* cf. *zhaoermii* by their shorter rise time, 23.3 ms (versus longer, 57.4, 59.7, 39.2 and 31.6 ms, respectively). Specifically in comparison to the closely related species *Jingophrys hrdy* sp. nov., the calls of *Jingophrys taniitatii* sp. nov. differ by their faster pulse rate, 184.13 pulses/s (versus slower, 127.48 pulses/s); shorter call duration, 54.5 ms (versus longer, 180.2 ms); slightly higher frequency, 3.59 kHz (versus lower, 3.23 kHz); and fewer pulses per call, 9–11 (versus more, 22–27 pulses per call) (figure 19; supplementary material, table S7).

Additionally, we compared vocalizations of *Jingophrys taniitatii* sp. nov. from our study with those published for three members in the People's Republic of China [29,31]. Calls of *Jingophrys taniitatii* sp. nov. differ from those of *J. feii* and *J. yeae* by their lower dominant frequency, 3.45–3.75 kHz (versus higher, 4.74–4.91 kHz and 4.4–5.2 kHz, respectively); from those of *J.* cf. *pachyproctus* by their higher dominant frequency, 3.45–3.75 kHz (versus lower, 3.2–3.3 kHz); from the calls of all three species and by their shorter call duration, 54.5 ms (versus longer, 132.6 ms in *J. feii*, 746 ms in *J.* cf. *pachyproctus* and 139 ms in *J. yeae*, respectively); and from the calls of *J. feii* and *J.* cf. *pachyproctus* by their fewer pulses per call, 9–11 (versus more, 14–20 pulses per call and 22–62 pulses per calls, respectively) (supplementary material, table S7).

**Distribution.** *Jingophrys taniitatii* sp. nov. is currently known only from four sites in the Tale Valley WLS (Pange, Manipolyang-Pange road, Tarin Tibe Khole Pass) and Ziro Valley (Tajgi Village) in the Lower Subansiri District, Arunachal Pradesh State, India, at elevations ranging from 1740 to 1945 m asl (figure 6; supplementary material, table S2).

**Habitat and natural history** **.** The habitat of *Jingophrys taniitatii* sp. nov. is primary forest with lush undergrowth, mostly on the slopes where trees are less abundant and in areas with prominent undergrowth. The holotype was found perched on a branch of a fallen tree next to a small hill stream. Owing to the distinctive warty dorsal skin of this species, it camouflages well with tree trunks and can go unnoticed unless heard calling. At the type locality, Tarin Tibe Khole Pass, two individuals collected during June in 2022 and 2023 were found calling while perched on a fallen tree trunk and a branch. The breeding season, therefore, likely falls during this month. At Tajgi Village, an individual was found calling from the edge of a small cascading stream. We found *Jingophrys taniitatii* sp. nov. to be very secretive or rare. We encountered only 1 or 2 individuals at any sampling site, even during the suspected peak calling month. *Jingophrys taniitatii* sp. nov. was found in sympatry with both *Jingophrys tanogogo* sp. nov. and *Jingophrys wularsoni* sp. nov. at three of the four sites (Pange, Tarin Tibe Khole Pass and Tajgi) (supplementary material, table S2).

***Jingophrys wularsoni* sp. nov.**

(http://zoobank.org/urn:lsid:zoobank.org:act:BCAC7B25-3491-4BBD-8CD5-47F973CF43C9)

figures 1–8, 19, 21 and 22; tables S1–S9 and figure S8 in the supplementary material

**Figure 21.**
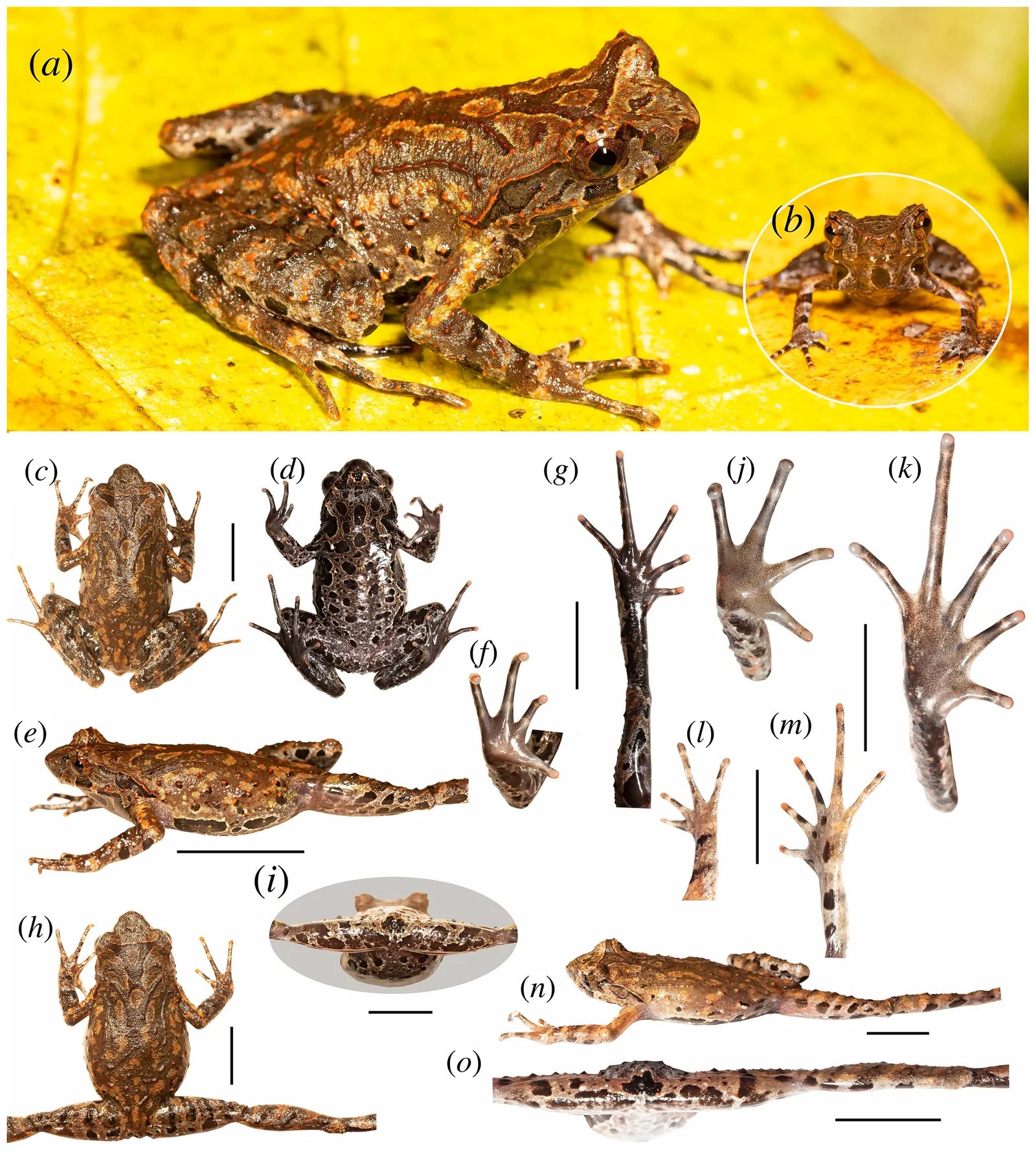
Type series of *Jingophrys wularsoni* sp. nov., in life. (*a–i*) Holotype (ZSI A 16631, adult male): (*a*) dorsolateral view, (*b*) frontal view, (*c*) dorsal view, (*d*) ventral view, (*e*) lateral view of flank, groin and thigh, (*f*) ventral view of hand, (*g*) ventral view of foot, (*h*) dorsal view of dorsum and thighs and (*i*) posterior view of thighs; (*j–o*) paratype (ZSI A 16634, adult male): (*j*) ventral view of hand, (*k*) ventral view of foot, (*l*) dorsal view of hand, (*m*) dorsal view of foot, (*n*) lateral view of flank, groin and thigh and (*o*) posterior view of thighs and shank. Scale bars = 10 mm. Photographs: S. D. Biju.

**Figure 22.**
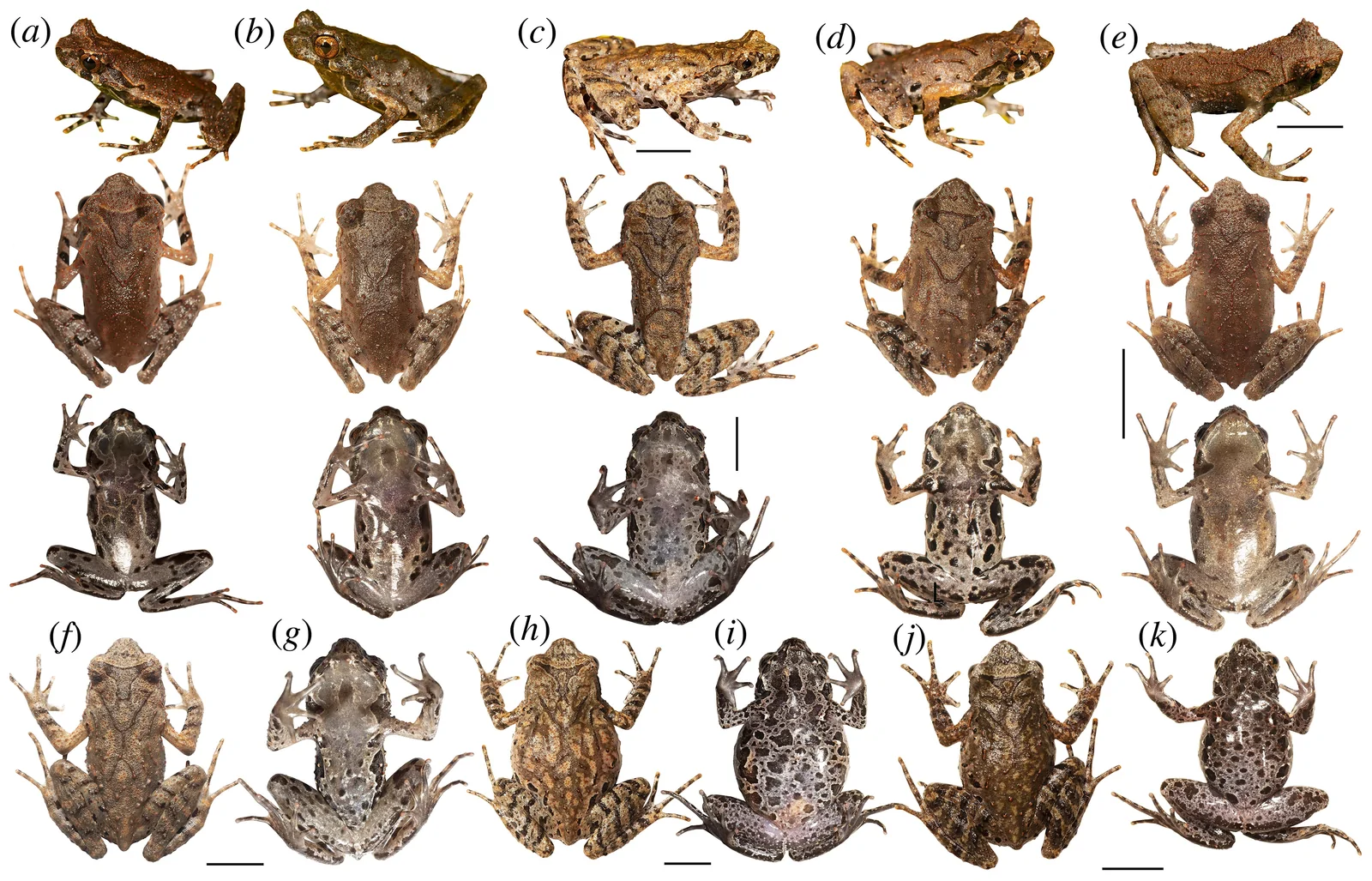
Additional paratypes and referred specimens of *Jingophrys wularsoni* sp. nov., in life. (*a–e*) Dorsolateral, dorsal and ventral views arranged from top to bottom, respectively; (*a*) adult male, not preserved, (*b*) adult male, not preserved, (*c*) adult male, ZSI A 16636, (*d*) adult male, not preserved and (*e*) subadult male, SDBDU 2023.6469; (*f*, *g*) dorsal and ventral views of an adult male, ZSI A 16634; (*h*, *i*) dorsal and ventral views of an adult female, ZSI A 16635; (*j*, *k*) dorsal and ventral views of an adult male, ZSI A 16632. Scale bars = 10 mm. Photographs: S. D. Biju.

**Holotype.** An adult male, ZSI A 16631 (SDB 2021.5053), from Pange (27.5443° N, 93.8946° E, 1925 m asl), Tale Valley Wildlife Sanctuary, Lower Subansiri District, Arunachal Pradesh State, India, collected by SDB, TT, SG and RUK on 18 October 2021.

**Paratypes.** Two adult males, ZSI A 16632–16633 (SDB 2021.5111 and SDB 2021.5057), collected with the holotype; an adult male, ZSI A 16634 (SDB 2023.6416), collected from the type locality by SDB, SG, RUK and TT on 10 May 2023; an adult female, ZSI A 16635 (SDB 2021.5110), collected from the type locality by SDB, TT, SG and RUK on 20 October 2021; an adult male, ZSI A 16636 (SDB 2022.5275), from Tajgi Village (27.6402°N, 93.8638° E, 1740 m asl), Ziro Valley, Lower Subansiri District, Arunachal Pradesh State, India, collected by SBD, SG, RUK and TT on 19 April 2022; an adult male, ZSI A 16637 (SDB 2022.6023), from Manipolyang-Pange (27.5246°N, 93.8689° E, 1780 m asl), Tale Valley Wildlife Sanctuary, Lower Subansiri District, Arunachal Pradesh State, India, collected by SBD, SG, RUK and TT on 16 June 2022.

**Referred specimens.** An adult male, SDBDU 2021.5112, collected with the holotype; an adult male, SDBDU 2022.5244, collected from the type locality by SDB, SG, RUK and TT on 11 April 2022; and an adult male, SDBDU 2022.5276, from Tajgi Village (27.6402°N, 93.8638° E, 1740 m asl), Ziro Valley, Lower Subansiri District, Arunachal Pradesh State, India, collected by SBD, SG, RUK and TT on 19 April 2022.

**Etymology.** The species name honours Barbara Wu and Eric Larson in appreciation of their long-time, generous support for herpetological field research through The Ken Miyata Fund to the Museum of Comparative Zoology, Harvard University. The specific epithet *wularsoni* is treated as an invariable noun in apposition to the generic name.

**Suggested common name.** Wu-Larson's horned frog.

**Diagnosis.** *Jingophrys wularsoni* sp. nov. can be distinguished from other known congeners based on a combination of characters that include large adult male size (SVL 36.4–40.8 mm), toes without lateral fringes and tips rounded, dark brown colouration and tubercles around the flank, prominent blackish-brown blotches on the belly and alternating dark blackish-brown and greyish-brown vertical stripes on the margins of both upper and lower jaws.

**Phylogenetic relationships.** *Jingophrys wularsoni* sp. nov. is a member of the *J. pachyproctus* species group and has a sister-group relationship with *J. pachyproctus* (figures 1–3). Within its species group, *Jingophrys wularsoni* sp. nov. shows the following DNA sequence divergence in the mitochondrial 16S rRNA gene: 6.7–6.9% from *Jingophrys hrdy* sp. nov.; 3.4–3.7% from *J. pachyproctus*; 8.3% from *Jingophrys taniitatii* sp. nov.; and 7.7% from the undescribed candidate species *J*. cand. sp. B. (supplementary material, table S3).

**Holotype description (measurements in mm).** Adult male (SVL 36.5), stout body; head moderately large, as long as wide (HL 13.3, HW 13.3, MN 10.7; MFE 8.8; MBE 5.1); snout nearly rounded in dorsal view, acutely protruding in lateral view, snout tip projecting far beyond lower lip (SP 2.5); loreal region nearly vertical, concave; canthus rostralis rounded; dorsal snout slightly concave; eye diameter larger than maximum tympanum diameter but smaller than snout length (EL 3.8, TYD 1.7, SL 5.0); tympanum-to-eye distance (TYE 2.5) longer than maximum tympanum diameter (TYD 1.7); tympanum moderately distinct, rounded, upper border concealed by supratympanic ridge; pupil oval in preservative, oriented vertically; nostril oval, oriented laterally, closer to eye than to snout (EN 2.2, NS 2.4); internarial distance larger than upper eyelid width and smaller than narrowest point between upper eyelids (IN 4.0, UEW 3.3, IUE 4.3); distance between posterior margins of eyes (IBE 11.6) 1.8 times the distance between anterior margins of eyes (IFE 6.4); pineal ocellus not visible externally; vomerine ridges present, weakly developed and without teeth; maxillary teeth present; tongue large, rounded, with no medial lingual process. Forelimbs long; forearm shorter than hand length (FAL 8.3, HAL 11.1); fingers moderately long, thin, lateral dermal fringes absent, relative finger lengths I < II < IV < III (FIL 3.9, FIIL 4.9, FIIIL 8.1, FIVL 6.1); interdigital webbing, and subarticular and supernumerary tubercles absent; thenar and outer metacarpal tubercles weakly developed; fingertips rounded, raised ventrally, not expanded relative to digit widths, terminal grooves absent on fingertips. Hindlimbs long, thin; thigh shorter than shank and longer than foot (TL 17.9, SHL 18.1, FOL 17.1); distance from heel to tip of toe IV (TFOL 26.3); toes rounded without lateral fringes, relative toe lengths I < II < V < III < IV; tips not dilated, with distinct pads raised ventrally, terminal grooves absent on toe tips; base of toes without webbing; outer metatarsal tubercle, and subarticular and supernumerary tubercles absent; inner metatarsal tubercle present but barely distinguishable (figure 21).

*Skin texture of holotype*. Dorsal surfaces of head and anterior dorsum shagreened to sparsely granular; posterior dorsum shagreened and slightly more granular with scattered tubercles; flanks and lateral surfaces of abdomen with prominent large scattered tubercles; limbs shagreened with small granules and scattered tubercles; tympanum smooth, borders raised, surrounding area posterior to eye granular; supratympanic fold well developed, extends from posterior corner of eye to above forearm insertion, narrow anteriorly and widens posteriorly into an enlarged glandular tubercle; prominent tubercle present on upper eyelid with a tiny horn-like tubercle in the middle of outer edge; inverted triangular ridge between eyes extends halfway over upper eyelids; short, thin and discontinuous dorsolateral dermal ridges extend on either side from behind supratympanic fold to nearly halfway on groin; distinct parietoscapular-sacral ridges form a vertically oriented ‘> <’ configuration, the two arms of the first inverted ‘V’-shaped ridge begin above tympanum and extend posteriorly where they meet medially beyond the level of axilla, and the second inverted ‘V’-shaped ridge present at mid-dorsum; dorsal surfaces of forearms, thighs and shanks with several rows of small tubercles arranged transversely. Ventral surfaces of body and limbs smooth to shagreened; dermal asperities on lower jaw weakly developed; snout tip projection of lower lip sparsely granular; pectoral glands small and raised, rounded, positioned at level of axilla; belly with tubercles on each side arranged from below forearm insertion to near groin; femoral glands small, rounded, weakly developed, slightly raised, closer to outer edge of knee than to cloaca; prominent tubercles on cloacal region (figure 8*k*,*v*).

*Colour of holotype*. *In life*. Dorsum and hindlimbs light reddish brown with light greyish-brown blotches on dorsum; inverted triangular marking between eyes dark brown; borders of dorsolateral ridges dark brown; parietoscapular-sacral ridges dark brown and roughly form a ‘X’-shape on dorsum; upper margins of supratympanic folds orangish red; tubercles on ridges and dorsal tubercles orangish red; tympanum and surrounding inner margin of supratympanic folds dark brown; anterior snout dark brown; lateral surfaces of head greyish brown; dark brown bands on upper lips on lateral surfaces of head; short dark bands on margins of upper eyelids; flanks lighter than dorsum, tubercles on flanks orangish red surrounded with dark brown spots; iris reddish brown with golden tinge; dorsal surfaces of fore- and hindlimbs, including fingers and toes, with prominent blackish-brown crossbands that extend towards ventral surfaces. Ventral surfaces of throat, chest, belly and thighs light greyish brown with large dark blackish-brown blotches with light grey margins; a longitudinal blotch extends from the mid-gular region to anterior chest; broad dark grey band extends from forearm insertion to groin on either side of belly; fore- and hindlimbs greyish brown with blackish-brown blotches; anterior and posterior parts of thighs light orangish grey with blackish-brown blotches; blackish-brown colouration around the cloacal opening; tips of fingers and toes and margins of toes light reddish orange (figure 21). *In preservative*. Dorsum, limbs, lateral surfaces of head and flanks light greyish brown; inverted triangular marking between eyes light brown with light grey central blotch; obscure light brown ‘X’-shaped mark on dorsum; upper eyelids dark greyish brown; all blackish-brown blotches turned to lighter greyish brown. Ventral surfaces of throat and chest greyish brown with brownish blotches; belly light greyish brown with blackish-brown blotches; hand and foot light greyish brown (supplementary material, figure S8).

**Morphological variation.** Morphometric variation among seven types and three referred specimens (nine males and one female) is provided in table S5 in the supplementary material. The paratypes and referred specimens resemble the holotype in most morphological characters, with some exceptions. *Colour in life*: ZSI A 16634 (SDB 2023 6416)—dorsum light greyish brown with more prominent granulation on dorsal skin and ventral surfaces light greyish brown with fewer dark blotches on throat. ZSI A 16635 (SDB 2021.5110) and ZSI A 16632 (SDB 2021.5111)—dorsum dark reddish brown with irregular yellowish-brown blotches, minute orangish-red spots inside the blotches and ventral surface including fore- and hindlimbs with prominently blackish-brown marbling and large blotches. SDBDU 2021.5041—dorsum dark reddish brown and ventral surfaces light greyish brown with black blotches. SDBDU 2023.6469 (subadult male)—dorsum reddish-brown with deep red granulations, prominent granulation on anterior half of dorsum, upper eyelids prominently granular, finger and toe tips deep orangish red and fewer blotches on ventral surfaces. *Skin texture:* ZSI A 16635 (SDB 2021.5110, female) and ZSI A 16632 (SDB 2021.5111, male)—more dermal asperities on dorsal skin compared to other males. ZSI A 16635 (SDB 2021.5110), SDBDU 2022.5276 and ZSI A 16636 (SDB 2022.5275)—more prominent ‘> <’ shaped parietoscapular-sacral ridges (figure 22).

**Morphological comparison.** *Jingophrys wularsoni* sp. nov. cannot be confused with two megophryid species whose phylogenetic placement within their currently assigned genus (or subgenus) remains unknown. It can be distinguished by the following characters: from *Xenophrys damrei* by its smaller adult male size, SVL 36.2–40.8 mm, *n =* 9 (versus larger, adult male SVL 47.7–57.1 mm, *n =* 7 [96,97]); and from *Ophryophryne pachyproctus* by the presence of maxillary teeth (versus absent) and the absence of horn-like tubercle on outer margin of upper eyelids (versus single horn-like tubercle) [98].

Within the genus, *Jingophrys wularsoni* sp. nov. differs from all currently recognized species (including six previously known and five newly described) by the presence of vomerine teeth (versus absent, except in *J. pachyproctus*). However, it differs from *J. pachyproctus* by the absence of nuptial pads with dense nuptial spines on the first finger (versus present [30]). *Jingophrys wularsoni* sp. nov. also differs from *Jingophrys hrdy* sp. nov., *Jingophrys taniitatii* sp. nov., *Jingophrys tanogogo* sp. nov. and *J.* cf. *zhaoermii* by its larger adult male size, SVL 36.4–40.8 mm, *n =* 9 (versus smaller than 36 mm in all others, SVL 30.0–36.2 mm, *n =* 6; SVL 27.4–31.1 mm, *n =* 4; SVL 24.9–30.3 mm, *n =* 8; and SVL 22.5–26.5 mm, *n =* 3, respectively). *Jingophrys wularsoni* sp. nov. differs from *J. feii* (based on [29]) by its larger adult male size, SVL 36.4–40.8 mm, *n =* 9 (versus SVL 24.3–25.1 mm, *n =* 4); upper eyelid width narrower than distance between upper eyelids, UEW/IUE 0.68–0.79 (versus upper eyelid width nearly equal to distance between eyelids, UEW/IUE 0.94–1.03); tympanum-to-eye distance longer than tympanum diameter, TYE/TYD 1.26–1.56 (versus tympanum-to-eye distance shorter than tympanum diameter, TYE/TYD 0.63–0.67); dorsal skin with scattered smooth granules (versus distinctly granular and with dense small tubercles); absence of lateral fringes on toes (versus well-developed lateral fringes); and belly light to dark brown with prominent contrasting blackish-brown blotches (versus belly white with distinct, irregular small dark brown spots and flecks). *Jingophrys wularsoni* sp. nov. differs from *J. pachyproctus* by its head nearly as wide as long, HW/HL 0.99–1.01 (versus head wider than long [31]); tympanum-to-eye distance longer than tympanum diameter, TYE/TYD 1.26–1.56 (versus same distance as diameter of tympanum [30]); thigh nearly equal to shank, TL/ShL 0.98–1.05 (versus thigh longer than shank [30]); lateral surfaces of dorsum, including flanks, with large, raised tubercles (versus granular with scattered tubercles [male HT, NWIPB 770650 [31]); shank longer than foot, FOL/ShL 0.86–0.94 (versus equal [30]); and the absence of nuptial pad with dense nuptial spines on the first finger (versus present [30]). *Jingophrys wularsoni* sp. nov. differs from *J. vegrandis* by its tympanum-to-eye distance longer than tympanum diameter, TYE/TYD 1.26–1.56 (versus tympanum-to-eye distance shorter than tympanum diameter, TYE/TYD 0.61–0.79); thigh nearly equal to shank, TL/ShL 0.98–1.05 (versus thigh shorter than shank, TL/ShL 0.90–0.95); and the absence of lateral dermal fringes on toes (versus present). *Jingophrys wularsoni* sp. nov. differs from *J. yeae* by its larger adult male size, SVL 36.4–40.8 mm, *n =* 9 (versus smaller, SVL 24.9–28.0 mm, *n =* 7); thigh nearly equal to shank, TL/ShL 0.98–1.05 (versus thigh shorter than shank, TL/ShL 0.89–0.98); and ventral surfaces of throat, belly and thighs in life light to dark greyish brown with blackish-brown blotches (versus throat light grey without prominent markings, belly with scattered light brown blotches and thighs immaculate). *Jingophrys wularsoni* sp. nov. differs from *J. zhoui* (based on [31]) by its larger adult size, male SVL 36.4–40.8 mm, *n =* 9, female SVL 47.6 mm (versus male SVL 22.0 mm, *n =* 1, female SVL 23.5–23.9 mm, *n =* 2); head nearly as wide as long, HW/HL 0.99–1.01 (versus head longer than wide); thigh nearly equal to shank, TL/ShL 0.98–1.05 (versus thigh shorter than shank); dorsum in life light to dark brown (versus dorsum and limbs orange-brown); and ventral surfaces of throat, belly and thighs in life light to dark greyish brown with blackish-brown blotches (versus throat light brown, abdomen orange brown and stained with several faint orange dots, and lower abdomen white with orange dots).

We also compared *Jingophrys wularsoni* sp. nov. with an additional population of *J. pachyproctus* from Bari and Renqingbeng Temple, ca. 20 km from Gelin, reported as *J.* cf. *pachyproctus* [31]. Differences between these two populations with respect to ‘inner metatarsal tubercle partially fused with toe I’ and ‘large arc-shaped protuberance’ beyond the cloaca were noted as being weak [15]. Multiple studies have observed that the degree of protuberance beyond the cloaca can vary among individuals [15,29,108,109], even within the same population (present study, figure 22). Similarly, the state of the inner metatarsal tubercle also varies among individuals [15]. Based on their study of both populations, Lyu *et al.* [15] assign the ‘*Megophrys* cf. *pachyproctus*’ population of Shi *et al.* [31] to *J. pachyproctus*. Nevertheless, since our new species *Jingophrys wularsoni* sp. nov. is closely related to *J. pachyproctus*, we additionally provide a separate comparison with *J.* cf. *pachyproctus* of Shi *et al.* [31] to avoid further confusion. *Jingophrys wularsoni* sp. nov. differs from *J.* cf. *pachyproctus* sensu Shi *et al.* [31] by its larger adult male size, SVL 36.4–40.8 mm, *n =* 9 (versus smaller, SVL 33.6–36.6 mm, *n =* 5); head nearly as wide as long, HW/HL 0.99–1.01 (versus head wider than long, HW/HL 1.04–1.18); snout longer than eye length, SL/EL 1.32–1.57 (versus snout relatively shorter than eye length, SL/EL 0.85–1.33); tympanum-to-eye distance longer than tympanum diameter, TYE/TYD 1.26–1.56 (versus tympanum-to-eye distance relatively shorter than tympanum diameter, TYE/TYD 0.75–1.26); and ventral skin in life light to dark greyish brown with blackish-brown blotches (versus light greyish brown with brownish blotches). For additional comparisons, see the ‘Morphological comparisons’ sections for the respective species.

**Secondary sexual characters.** Males: nuptial pads or spines not clearly visible other than a swelling on finger I; and a single subgular vocal sac while calling. Females: studied specimens contain moderately large (diameter 3.0 ± 0.4 mm, *n =* 20), creamy white (pigmentless) ova.

**Vocalization.** Calls of male *Jingophrys wularsoni* sp. nov. were recorded at 22.00 hours on 16 June 2023 at Pange (Tale Valley WLS), Lower Subansiri District, Arunachal Pradesh. Ambient temperatures were 17.2°C dry bulb and 16.8°C wet bulb. Based on analyses of 50 calls from a single individual, the typical vocal repertoire of this species is as follows: males produce a single type of call that has a pulsatile temporal structure; calls are not delivered in groups; the mean call duration is 51.7 ms; the amplitude envelope has a mean rise time of 15.6 ms and a mean fall time of 36.1 ms; calls deliver 7–9 pulses at a mean rate of 140.8 pulses/s; and the call spectrum shows a single broad peak with a mean dominant frequency of 2.71 kHz (figure 19*i*–*l*; supplementary material, table S7).

**Call comparison.** Calls of *Jingophrys wularsoni* sp. nov. can be differentiated from those of congeners primarily by their frequency, pulse rate, duration and rise time, as follows: (i) *Call frequency*: *Jingophrys wularsoni* sp. nov. calls have the lowest dominant frequency (2.7 kHz) among studied congeners (versus higher, 4.34 kHz in *Jingophrys siangensis* sp. nov., 3.23 kHz in *Jingophrys hrdy* sp. nov., 3.59 kHz in *Jingophrys taniitatii* sp. nov., 4.88 kHz in *Jingophrys tanogogo* sp. nov., 5.46 kHz in *J. vegrandis*, 4.61 kHz in *J.* cf. *yeae* and 4.57 kHz in *J.* cf. *zhaoermii*). (ii) *Pulse rate*: *Jingophrys wularsoni* sp. nov. calls differ from those of *Jingophrys hrdy* sp. nov., *J. vegrandis*, *J.* cf. *yeae*, *J.* cf. *zhaoermii* by their higher pulse rate, 140.8 pulses/s (versus lower, 127.48, 71.03, 102.5 and 99.46 pulses/s, respectively); and from those of *Jingophrys siangensis* sp. nov., *Jingophrys taniitatii* sp. nov. and *Jingophrys tanogogo* sp. nov. by their lower pulse rate, 140.8 pulses/s (versus higher, 193.66, 184.13 and 140.98 pulses/s, respectively). (iii) *Call duration*: *Jingophrys wularsoni* sp. nov. calls differ from those of *Jingophrys hrdy* sp. nov., *Jingophrys siangensis* sp. nov., *Jingophrys taniitatii* sp. nov., *J. vegrandis*, *J.* cf. *yeae*, *J.* cf. *zhaoermii* by their shorter duration, 51.7 ms (versus longer, 180.2, 59.7, 54.5, 166.6, 109.4 and 78.6 ms, respectively). (iv) *Call rise time*: *Jingophrys wularsoni* sp. nov. calls differ from those of *Jingophrys hrdy* sp. nov., *Jingophrys siangensis* sp. nov., *Jingophrys taniitatii* sp. nov., *J. vegrandis*, *J.* cf. *yeae* and *J.* cf. *zhaoermii* by their shorter call rise time, 15.59 ms (versus longer, 57.4, 21.5, 23.3, 59.7, 39.2 and 31.6 ms, respectively). *Jingophrys wularsoni* sp. nov. calls also differ from those of *Jingophrys tanogogo* sp. nov. by their longer duration, 51.7 ms (versus shorter, 31.12 ms), and by their slightly more pulses per call, 7–9 (versus fewer, 5–6 pulses per call), although pulse rates of the two species are similar (figure 19; supplementary material, table S7).

Additionally, we compared vocalizations of *Jingophrys wularsoni* sp. nov. with those published for three members in the People's Republic of China [29,31]. Calls of *Jingophrys wularsoni* sp. nov. differ from those of *J. feii*, *J.* cf. *pachyproctus* and *J. yeae* by their lower dominant frequency, 2.63–2.8 kHz (versus higher, 4.74–4.91, 3.2–3.3 and 4.4–5.2 kHz, respectively), and shorter duration, 51.7 ms (versus longer, 132.6, 746 and 139 ms, respectively); and also from *J. feii* and *J.* cf. *pachyproctus* calls by their fewer pulses per call, 7–9 (versus more, 14–20 and 22–62 pulses per call, respectively; supplementary material, table S7).

**Distribution.** *Jingophrys wularsoni* sp. nov. is currently known only from its type locality and vicinity (Pange, Tarin Tibe Khole Pass and Tasi Biidang), which lie within a radius of about 3 km in the Tale Valley WLS, and from Tagji Village in nearby Ziro Valley, all in the Lower Subansiri District, Arunachal Pradesh State, India, at elevations ranging from 1740 to 2121 m asl (figure 6; supplementary material, table S2).

**Habitat and natural history** **.** Habitat at the known localities of *Jingophrys wularsoni* sp. nov. consists of primary tropical evergreen forest with sparsely closed canopy and scattered bamboo patches. All specimens were collected at night from tree trunks covered with moss or leaves of overgrown shrubs adjacent to small seasonal streams. The HT was calling from a tree trunk at a height of 1 m above-ground and approximately 4–6 m from a temporary stream. All calling individuals were collected between May and July, which we regard as the peak breeding season since calling activity ceases after this time of the year. An off-season survey conducted during October 2022 resulted in the collection of multiple individuals, but no calling was heard. *Jingophrys wularsoni* sp. nov. is sympatric with *Jingophrys taniitatii* sp. nov. and *Jingophrys tanogogo* sp. nov. at both Pange and Tarin Tibe Khole Pass (Tale Valley WLS) and Tajgi (Ziro Valley) at elevations between 1740 and 1945 m asl, and with *Jingophrys hrdy* sp. nov. at a slightly higher elevation, 2121 m asl, at Tasi Biddang (Tale Valley WLS) (supplementary material, table S2).

**Note.** Saikia & Sinha [32] reported a megophryid frog, identified as ‘*Megophrys pachyproctus* Huang, 1981’, from ‘Pange, Talle Valley Wildlife Sanctuary’, with a description citing a male specimen, V/A/NERC/1352. Our detailed studies based on multiple new collections from this region confirm that ‘*Megophrys pachyproctus* Huang, 1981’ is not found at Pange (Tale Valley WLS), and based on our collections, we suggest that V/A/NERC/1352 should be assigned to *Jingophrys wularsoni* sp. nov. described here.

## 4. Discussion

Recent findings on *Jingophrys*, including the present work, illustrate how an entire radiation of frogs can go overlooked in the absence of dedicated sampling efforts and comprehensive taxonomic study. First and foremost, they reinforce the importance of primary field data as a critical step for documenting the remarkable diversity, richness and biology of species in understudied regions of tropical Asia. When combined with additional evidence, including morphology, molecular phylogenetics, bioacoustics, geography and natural history, such data not only allow reliable delimitation of species but also enable robust inferences of systematic relationships that yield further evolutionary insights. In the case of *Jingophrys*, their relatively small body size (males, SVL 22–41 mm) and more terrestrial microhabitat, along with arboreal calling sites, may have contributed to them being overlooked during earlier surveys for megophryid frogs, especially in comparison to the closely related and larger (males, SVL 30–100 mm [15]) *Xenophrys* found in stream-associated microhabitats in the same geographical regions. Many other megophryine clades have similarly overlapping ranges across South and Southeast Asia [15]. It will be interesting to investigate whether they also have dissimilar ecological associations.

The present study increases the recognized diversity of *Jingophrys* by more than two-fold, from six to 11 described species (or 13 distinct species-level lineages, including two unnamed candidate species). Species diversity has risen seven-fold for India alone, from an earlier two species (one confirmed and one misidentified) to now eight, including five new species and two new country records (or nine distinct species-level lineages, including one unnamed candidate species). Based on multiple new samples and additional populations, our study captures levels of intra- and interspecific genetic divergence among congeners in the fragment of the mitochondrial 16S rRNA gene that is frequently used for DNA barcoding studies of anurans and can vary among taxonomic groups (e.g. [90–92,110]). Our results are also supported by the absence of shared haplotypes among all putative species in nuclear DNA sequences, as well as phylogenetic inferences based on both mitochondrial and nuclear DNA. Future larger datasets may better resolve population-level patterns of gene flow, especially among genetically divergent populations of *J. yeae* and *J. zhaoermii*, which appear to have experienced more recent gene flow that likely confounds putative species boundaries in these complexes. At the same time, we demonstrate the benefits of an integrative taxonomic approach that includes morphology and behaviour, which are effective tools for delineating species boundaries in the present study.

Patterns of geographic distribution suggest the likely interplay among different modes of speciation in shaping the present diversity of *Jingophrys*: (i) Sympatry: closely related species of the same group occur at the same location. In the *J. pachyproctus* group, for example, *Jingophrys hrdy* sp. nov. is sympatric with *Jingophrys taniitatii* sp. nov. at one site and with *Jingophrys wularsoni* sp. nov. at another. Similarly, in the *J. vegrandis* group, *J. yeae*, *J. zhaoermii* and *J. zhouii* are sympatric. (ii) Allopatry: closely related species of the same group occur in widely separated mountain ranges and river valleys. Two examples are the geographic separation of *J. pachyproctus* and *Jingophrys wularsoni* sp. nov. in the *J. pachyproctus* group, and of *J. vegrandis*, *Jingophrys tanogogo* sp. nov., and potentially related *Jingophrys siangensis* sp. nov. in the *J. vegrandis* group. (iii) Parapatry: closely related species of the same group occur in geographically adjacent and potentially continuous larger regions. Two possible examples are the likely ongoing speciation events involving *J. yeae* sensu stricto and *J. zhaoermii* sensu stricto relative to their respective divergent populations *J.* cf. *yeae* and *J.* cf. *zhaoermii*. (iv) Peripatry: a species occurs on an isolated mountain top at the edge of a larger population of a closely related species. An example is *Jingophrys hrdy* sp. nov. and *Jingophrys taniitatii* sp. nov. of the *J. pachyproctus* group (figure 6; supplementary material, table S2). Elevation also appears to be a likely driver of speciation at a regional scale, as observed from the elevational zonation of geographically close species in at least two species-rich regions—Tale Valley and Ziro Valley in Lower Subansiri District of Arunachal Pradesh, India, and Medog County in Xizang Autonomous Region (Tibet), People's Republic of China. This pattern could be specific to these two regions owing to their unique and perhaps comparable physiography, but it also suggests that other, less intensively surveyed localities may also harbour higher species richness. The numerous instances of sympatric species are especially interesting as they also point to possible ecological niche partitioning among members of the same or different species groups. Future studies focusing on the reproductive biology and natural history of *Jingophrys* could yield valuable knowledge to address these questions.

In groups of morphologically cryptic frogs, behavioural traits may provide useful diagnostic characters. This is especially true for male advertisement calls, which function as premating isolation mechanisms between species [83,84,111,112]. In this study, call data reliably differentiate all eight Indian species based on variation in temporal and spectral properties; they offer an extremely useful, non-invasive tool for diagnosing *Jingophrys* species in the field. However, we emphasize that our study does not account for possible additional sources of inter- and intraspecific call variation, such as ambient temperature and social context, which may warrant future dedicated bioacoustics investigations [85]. At the same time, little is known about the breeding biology of these newly reported populations, which require additional, long-term field-based studies. However, the tadpoles of three species show relatively subtle morphological differences, and when combined with egg clutch or developmental characteristics they may yield useful systematic and evolutionary insights [88,113].

Our unexpected observations of land snails as stomach contents in adults of two closely related species (*Jingophrys tanogogo* sp. nov. and *J. vegrandis*) are the first reported for this genus and family. They offer insights into adult diet as well as pose questions regarding the feeding behaviour and mechanisms that enable ingestion and swallowing of whole snails. Megophryids are regarded as generalist feeders, albeit with a largely insectivorous diet, but the ability to eat large prey items is likely determined by gape size; large-size species such as *Megophrys nasuta* are postulated to have relatively wider mouths [114–116]. A diet of relatively large snails by small *Jingophrys* may indicate that skull shape and size, and especially location of the jaw joints [117–119], play an important role in determining feeding habits in addition to the role played by body size alone. Detailed study of cranial osteology, and particularly gape size, as well as soft tissue anatomy using 3D micro-CT imaging will likely enhance our understanding of these aspects [117,119,120]. Snails, or gastropod molluscs more broadly, have been reported in the diets of European pelobatids (*Pelobates fuscus* and *Pelobates syriacus* [2]), which is the family most closely related to megophryids; African pyxicephalids (*Pyxicephalus adspersus* [121]); African hyperoliids (*Paracassina kounhiensis* and *Paracassina obscura* [117]); South American ceratophryids (*Lepidobatrachus laevis* [122]); and Asian ranids (*Pelophylax nigromaculatus* [123]). We know of no previously published reports of snail-eating in megophryid frogs. It will be interesting to determine if this behaviour is more widespread in the family and opportunistic based on food availability, or if it is a specialized behaviour restricted to *Jingophrys*, as it is in some African hyperoliids [117] and in snail-eating snakes of the genera *Pareas* and *Internatus* [124].

Our phylogenetic analyses allowed robust testing of species hypotheses and inferences of systematic relationships, in addition to offering insights into patterns and timing of evolutionary divergence and biogeography, both within *Jingophrys* and among megophryines. Our species-rich dataset for *Jingophrys* (as many as 13 candidate species) confirms its distinct phylogenetic position and finds strong support for two species-group-level subclades as well as all species-level lineages. At the same time, there is weak support for several interspecific relationships and deeper nodes. Within *Jingophrys*, this is likely owing to incomplete data for several species, especially some of those recently described. For example, all available samples of *J. zhaoermii* have data for only two of six genetic markers used in our analyses. For deeper nodes, greater taxon sampling coupled with additional genetic markers or even genomic-level data may be needed to fully resolve relationships, especially among basal megophryine groups [5].

Our diversification analyses reveal diversification rate heterogeneity among megophryine lineages over time and, together with the timing and pattern of both lineage accumulation and historical biogeography, implicate key geological events. Chief among these are the coincident periods of active Himalayan–Tibetan orogeny through the Late Oligocene and Early Miocene and the strengthening of Asian monsoons beginning in the Late Miocene, which likely shaped the contemporary diversity and distribution patterns of Megophryinae across Asia. Several dispersal events also coincide with the Miocene–Pliocene and Pliocene–Pleistocene boundaries, suggesting that geologic and climatic events associated with these transitions may have had a role in shaping later diversification in the subfamily. In addition, founder-event speciation achieved through jump dispersals across significant geographical barriers, and gradual range expansions, appear to have played an influential role in the evolutionary history of the subfamily. In particular, our results reveal the Eastern Himalayas, which delineate the western limit of megophryine distribution, to be an important region of intensive *in situ* diversification of *Jingophrys* and certain lineages of the closely related *Xenophrys*. The likely interplay between river system-associated vicariance at a wide Himalayan scale and elevation-associated species partitioning at regional scales in shaping present-day diversification patterns is a hypothesis worth testing for *Jingophrys* and other regional faunas. Similar regional-scale hypothesis testing within other major centres of diversification across the megophryine phylogeny may tease out finer-scale evolutionary processes. Genomic-scale frameworks that incorporate increased taxon sampling can also enable more in-depth investigations of the origin, diversification and biogeography of megophryid frogs. Yet, efforts to increase taxon sampling can only be made synergistically with increased search efforts through fieldwork and basic taxonomic studies. Our study is a step in this direction; it fills an important gap by revealing patterns of species richness and endemism of an overlooked radiation in a biogeographically important yet understudied region. It also reinforces the status of the Eastern Himalayas, including the NER India, as a critical amphibian hotspot in and of itself—not simply as a historical gateway for faunal exchange between the Indian subcontinent and the rest of mainland Asia, but as a centre of *in situ* diversification hosting its own species-rich, distinct and microendemic fauna [125–127].

## Supplementary Material



## Data Availability

New DNA sequences have been deposited in the NCBI GenBank under accession numbers PZ484275–PZ484307, PZ485464–PZ485475 and PZ488134–PZ488175. Representative cuts of the call recordings analysed are deposited in the Zenodo repository under (doi:10.5281/zenodo.20480405). Type specimens are deposited at the Zoological Survey of India (ZSI A), Kolkata, India, and other referred voucher specimens are deposited at the Systematics Lab, University of Delhi (SDBDU), India. This work and the new species described in it have been registered in ZooBank under the publication LSID urn:lsid:zoobank.org:pub:9485DB13-FC2C-4ECE-90AB-387435ED010B. Zoobank LSIDs (in parentheses) for the new species are as follows: *Jingophrys hrdy* sp. nov. (http://zoobank.org/urn:lsid:zoobank.org:act:32DE21BE-A0B3-48B9-8FD8-E939264CFD2C); *Jingophrys siangensis* sp. nov. (http://zoobank.org/urn:lsid:zoobank.org:act:BE44F009-65B6-4263-AE42-431D4D9393D2); *Jingophrys taniitatii* sp. nov. (http://zoobank.org/urn:lsid:zoobank.org:act:F639055D-DA15-4542-8219-1E87CB68CCD9); *Jingophrys tanogogo* sp. nov. (http://zoobank.org/urn:lsid:zoobank.org:act:484EC6A4-4AF6-47CF-9BFC-1217BB2B5ABF); *Jingophrys wularsoni* sp. nov. (http://zoobank.org/urn:lsid:zoobank.org:act:BCAC7B25-3491-4BBD-8CD5-47F973CF43C9). All other data are included in the article and its supplementary material [128].
