# Supplementary material for: Two-fold hidden diversity of diminutive horned frogs (Megophryinae: Jingophrys) highlights overlooked in situ diversification in the Himalayas
Source: R Soc Open Sci. 2026 Nov 11;13(11):261030. doi: 10.1098/rsos.261030 (PMC13752532; doi:10.1098/rsos.261030)
Supplement: Supplementary file 1 [file rsos.261030.zip › rsos.261030/rsos261030_si_001.pdf]

Electronic Supplementary Material: Tables S1–S9

Two-fold hidden diversity of diminutive horned frogs (Megophryinae: *Jingophrys*) highlights overlooked *in situ* diversification in the Himalayas

Sonali Garg[1,3], S. D. Biju[1,2,3], Tage Tajo[3], Radhakrishna Upadhyaya K.[3], and James Hanken[1]

[1] Department of Organismic and Evolutionary Biology and Museum of Comparative Zoology and
[2] Radcliffe Institute for Advanced Study, Harvard University, Cambridge, Massachusetts, USA
[3] Systematics Lab, Department of Environmental Studies, University of Delhi, Delhi, India

Author for correspondence: Sonali Garg, e-mail: sonaligarg@fas.harvard.edu

Table S1. Taxa, localities, voucher numbers, and GenBank accession numbers for DNA sequences included in the study. Numbers inside parentheses after species names indicate voucher position on the phylogenetic trees. Abbreviation: WLS, Wildlife Sanctuary.

No.	Taxa	Collection Locality	Voucher Number	GenBank Accession Number					Reference
	**Family Megophryidae**								
	**Subfamily Megophryinae**								
	**Genus *Jingophrys***			16S	12SV16S	COI	RAG-1	CXCR-4	
1	*Jingophrys feii* (1)	China: Nabang, Yingjiang, Dehong, Yunnan	SYS a003874	OQ181005	OQ180951	OQ180891	–	–	Lyu et al. 2023
2	*Jingophrys feii* (2)	China: Nabang, Yingjiang, Dehong, Yunnan	SYS a003875	OQ181006	OQ180952	OQ180892	–	–	Lyu et al. 2023
3	*Jingophrys feii* (3)	China: Nabang, Yingjiang, Dehong, Yunnan	SYS a003876	OQ181007	OQ180953	OQ180893	OQ184111	OQ184115	Lyu et al. 2023
4	*Jingophrys hrdy* **sp. nov.** (1)	India: Tale Valley WLS, Lower Subansiri, Arunachal Pradesh	SDBDU 2021.5104	PZ484275	PZ488160	PZ485464	PZ488134	PZ488147	Present study
5	*Jingophrys hrdy* **sp. nov.** (2)	India: Tale Valley WLS, Lower Subansiri, Arunachal Pradesh	ZSI A 16624	PZ484276	PZ488161	PZ485465	PZ488135	PZ488148	Present study
6	*Jingophrys hrdy* **sp. nov.** (3)	India: Tale Valley WLS, Lower Subansiri, Arunachal Pradesh	SDBDU 2023.6535	PZ484277	–	–	–	–	Present study
7	*Jingophrys pachyproctus* (1)	China: Renqinbeng, Medog, Xizang (Tibet)	CIBMT171059	MN963201	MN963184	MN964328	MN984396	–	Shi et al. 2020
8	*Jingophrys pachyproctus* (2)	China: Renqinbeng, Medog, Xizang (Tibet)	CIBMT171058	MN963202	MN963183	MN964327	MN984395	–	Shi et al. 2020
9	*Jingophrys pachyproctus* (3)	China: Renqinbeng, Medog, Xizang (Tibet)	CIBMT171057	MN963203	MN963182	MN964326	MN984394	–	Shi et al. 2020
10	*Jingophrys pachyproctus* (4)	China: Renqinbeng, Medog, Xizang (Tibet)	CIBMT171056	MN963204	MN963181	MN964325	MN984393	–	Shi et al. 2020
11	*Jingophrys pachyproctus* (5)	China: Renqinbeng, Medog, Xizang (Tibet)	CIBMT171055	MN963205	MN963180	MN964324	MN984392	–	Shi et al. 2020
12	*Jingophrys pachyproctus* (6)	China: Renqinbeng, Medog, Xizang (Tibet)	CIBMT171054	MN963206	MN963179	MN964323	MN984391	–	Shi et al. 2020
13	*Jingophrys pachyproctus* (7)	China: Renqinbeng, Medog, Xizang (Tibet)	CIBMT171052	MN963208	MN963177	MN964321	MN984389	–	Shi et al. 2020
14	*Jingophrys pachyproctus* (8)	China: Bari, Medog, Xizang (Tibet)	CIB201706MT04	MN963214	MN963175	MN964315	MN984383	–	Shi et al. 2020

15	*Jingophrys pachyproctus* (9)	China: Bari, Medog, Xizang (Tibet)	CIB022017061813	MN963220	–	–	–	–	Shi et al. 2020
16	*Jingophrys pachyproctus* (10)	China: Bari, Medog, Xizang (Tibet)	CIB022017061812	MN963221	–	–	–	–	Shi et al. 2020
17	*Jingophrys pachyproctus* (11)	China: Bari, Medog, Xizang (Tibet)	CIB022017061807	MN963226	MN963170	MN964305	MN984375	MN984406	Shi et al. 2020
18	*Jingophrys pachyproctus* (12)	China: Bari, Medog, Xizang (Tibet)	CIB022017061806	MN963227	MN963169	MN964304	MN984374	MN984405	Shi et al. 2020
19	*Jingophrys pachyproctus* (13)	China: Bari, Medog, Xizang (Tibet)	CIB022017061805	MN963228	MN963168	MN964303	MN984373	MN984404	Shi et al. 2020
20	*Jingophrys siangensis* **sp. nov.** (1)	India: Pangin, East Siang, Arunachal Pradesh	SDBDU 2024.6728	PZ484278	PZ488162	–	–	–	Present study
21	*Jingophrys siangensis* **sp. nov.** (2)	India: Yembung, East Siang, Arunachal Pradesh	ZSI A 16615	PZ484279	PZ488163	–	–	PZ488149	Present study
22	*Jingophrys taniitatii* **sp. nov.** (1)	India: Tajgi, Ziro Valley, Lower Subansiri, Arunachal Pradesh	ZSI A 16630	PZ484280	PZ488164	PZ485466	PZ488136	PZ488150	Present study
23	*Jingophrys taniitatii* **sp. nov.** (2)	India: Tarin Tibe Khole Pass, Tale Valley WLS, Lower Subansiri, Arunachal Pradesh	ZSI A 16628	PZ484281	PZ488165	PZ485467	PZ488137	PZ488151	Present study
24	*Jingophrys taniitatii* **sp. nov.** (3)	India: Manipolyang-Pange, Lower Subansiri, Arunachal Pradesh	ZSI A 16629	PZ484282	–	–	–	–	Present study
25	*Jingophrys tanogogo* **sp. nov.** (1)	India: Hari, Ziro Valley, Lower Subansiri, Arunachal Pradesh	ZSI A 16616	PZ484283	PZ488166	PZ485468	PZ488138	PZ488152	Present study
26	*Jingophrys tanogogo* **sp. nov.** (2)	India: Pange, Tale Valley WLS, Lower Subansiri, Arunachal Pradesh	ZSI A 16618	PZ484284	–	–	–	–	Present study
27	*Jingophrys tanogogo* **sp. nov.** (3)	India: Pange, Tale Valley WLS, Lower Subansiri, Arunachal Pradesh	SDBDU 2021.5109	PZ484285	–	–	–	–	Present study
28	*Jingophrys tanogogo* **sp. nov.** (4)	India: Pange, Tale Valley WLS, Lower Subansiri, Arunachal Pradesh	SDBDU 2022.6026	PZ484286	–	–	–	–	Present study
29	*Jingophrys tanogogo* **sp. nov.** (5)	India: Tajgi, Ziro Valley, Lower Subansiri, Arunachal Pradesh	SDBDU 2023.4483	PZ484287	PZ488167	PZ485469	PZ488139	PZ488153	Present study
30	*Jingophrys vegrandis* (1)	India: Sessa, West Kameng, Arunachal Pradesh	ZSI A 11605 / SDBDU 2009.1272	KY022305	KY022305	MH647530	KY022349	KY022327	Mahony et al. 2017, 2018
31	*Jingophrys vegrandis* (2)	India: Sessa, West Kameng, Arunachal Pradesh	SDBDU 2009.1298	MN734413	MN734413	–	–	–	Mahony et al. 2020
32	*Jingophrys vegrandis* (3)	India: Sessa, West Kameng, Arunachal Pradesh	SDBDU 2022.6289	PZ484288	PZ488168	–	PZ488140	–	Present study
33	*Jingophrys vegrandis* (4)	India: Sessa, West Kameng, Arunachal Pradesh	SDBDU 2024.6619	PZ484289	–	–	–	–	Present study
34	*Jingophrys vegrandis* (5)	India: Sessa, West Kameng, Arunachal Pradesh	SDBDU 2024.6626	PZ484290	–	–	–	–	Present study
35	*Jingophrys wularsoni* **sp. nov.** (1)	India: Pange, Tale Valley WLS, Lower Subansiri, Arunachal Pradesh	ZSI A 16631	PZ484291	–	–	–	–	Present study
36	*Jingophrys wularsoni* **sp. nov.** (2)	India: Pange, Tale Valley WLS, Lower Subansiri, Arunachal Pradesh	ZSI A 16633	PZ484292	–	–	–	–	Present study
37	*Jingophrys wularsoni* **sp. nov.** (3)	India: Pange-Tasi biddang, Tale Valley WLS, Lower Subansiri, Arunachal Pradesh	SDBDU 2021.5105	PZ484293	–	–	–	–	Present study

38	*Jingophrys wularsoni* **sp. nov.** (4)	India: Pange, Tale Valley WLS, Lower Subansiri, Arunachal Pradesh	ZSI A 16635	PZ484294	–	–	–	–	Present study
39	*Jingophrys wularsoni* **sp. nov.** (5)	India: Tajgi, Ziro Valley, Lower Subansiri, Arunachal Pradesh	ZSI A 16636	PZ484295	–	–	–	–	Present study
40	*Jingophrys wularsoni* **sp. nov.** (6)	India: Manipolyang-Pange, Lower Subansiri, Arunachal Pradesh	ZSI A 16637	PZ484296	PZ488169	PZ485470	PZ488141	PZ488154	Present study
41	*Jingophrys wularsoni* **sp. nov.** (7)	India: Tasi biddang, Tale Valley WLS, Lower Subansiri, Arunachal Pradesh	SDBDU 2023.6469	PZ484297	PZ488170	PZ485471	PZ488142	PZ488155	Present study
42	*Jingophrys wularsoni* **sp. nov.** (8)	India: Pange, Tale Valley WLS, Lower Subansiri, Arunachal Pradesh	ZSI A 16634	PZ484298	–	–	–	–	Present study
43	*Jingophrys yeae* (1)	China: Yarang, Medog, Xizang (Tibet)	CIBMT171066	MN963196	MN963189	–	MN984401	–	Shi et al. 2020
44	*Jingophrys yeae* (2)	China: Yarang, Medog, Xizang (Tibet)	CIBMT171065	MN963197	MN963188	–	MN984400	–	Shi et al. 2020
45	*Jingophrys yeae* (3)	China: Yadong, Medog, Xizang (Tibet)	CIBMT171064	MN963198	MN963187	–	MN984399	–	Shi et al. 2020
46	*Jingophrys yeae* (4)	China: Suburb of Medog, Xizang (Tibet)	CIB201706MT03	MN963215	MN963174	MN964314	MN984382	–	Shi et al. 2020
47	*Jingophrys yeae* (5)	China: Beibeng, Medog, Xizang (Tibet)	CIB201706MT02	MN963216	MN963173	MN964313	MN984381	–	Shi et al. 2020
48	*Jingophrys yeae* (6)	China: Didong, Medog, Xizang (Tibet)	CIB201706MT01	MN963217	MN963172	MN964312	MN984380	–	Shi et al. 2020
49	*Jingophrys yeae* (7)	China: Bari, Medog, Xizang (Tibet)	CIB022017061811	MN963222	–	MN964309	MN984379	–	Shi et al. 2020
50	*Jingophrys yeae* (8)	China: Bari, Medog, Xizang (Tibet)	CIB022017061809	MN963224	MN963171	MN964307	MN984377	–	Shi et al. 2020
51	*Jingophrys yeae* (9)	China: Bari, Medog, Xizang (Tibet)	CIB022017061804	MN963229	MN963167	MN964302	MN984372	MN984403	Shi et al. 2020
52	*Jingophrys yeae* (10)	China: Beibeng, Medog, Xizang (Tibet)	CIB022017061606	MN963231	MN963166	MN964300	MN984370	–	Shi et al. 2020
53	*Jingophrys yeae* (11)	China: Beibeng, Medog, Xizang (Tibet)	CIB022017061407b	MN963238	MN963165	MN964294	MN984363	MN984402	Shi et al. 2020
54	*Jingophrys yeae* (12)	China: Didong, Medog, Xizang (Tibet)	CIB022017061104	MN963241	MN963164	MN964291	MN984360	–	Shi et al. 2020
55	*Jingophrys yeae* (13)	China: Didong, Medog, Xizang (Tibet)	CIB022017061103	MN963242	MN963163	MN964290	MN984359	–	Shi et al. 2020
56	*Jingophrys yeae* (14)	China: Didong, Medog, Xizang (Tibet)	CIB022017061102	MN963243	MN963162	MN964289	MN984358	–	Shi et al. 2020
57	*Jingophrys yeae* (15)	China: Beibeng, Medog, Xizang (Tibet)	KIZ010978	KX811908	–	KX812153	KX812265	–	Chen et al. 2017
58	*Jingophrys yeae* (16)	China: Beibeng, Medog, Xizang (Tibet)	KIZ011175	KX811909	–	KX812154	KX812266	–	Chen et al. 2017
59	*Jingophrys yeae* (17)	China: Medog, Xizang (Tibet)	SYS a004520	OQ181008	OQ180954	OQ180894	–	–	Lyu et al. 2023
60	*Jingophrys* cf. *yeae* (1)	India: Anini, Dibang Valley, Arunachal Pradesh	SDBDU 2022.6121	PZ484299	PZ488171	PZ485472	PZ488143	PZ488156	Present study
61	*Jingophrys* cf. *yeae* (2)	India: Anini, Dibang Valley, Arunachal Pradesh	SDBDU 2022.6126	PZ484300	–	–	–	–	Present study
62	*Jingophrys* cf. *yeae* (3)	India: Etalin, Dibang Valley, Arunachal Pradesh	SDBDU 2022.6149	PZ484301	–	–	–	–	Present study
63	*Jingophrys* cf. *yeae* (4)	India: Tiwari Gaon, Lower Dibang Valley, Arunachal Pradesh	SDBDU 2022.6190	PZ484302	–	–	–	–	Present study
64	*Jingophrys* cf. *yeae* (5)	India: Tiwari Gaon, Lower Dibang Valley, Arunachal Pradesh	SDBDU 2023.6379	PZ484303	PZ488172	PZ485473	PZ488144	PZ488157	Present study
65	*Jingophrys* cf. *yeae* (6)	India: Tiwari Gaon, Lower Dibang Valley, Arunachal Pradesh	SDBDU 2023.6512	PZ484304	–	–	–	–	Present study
66	*Jingophrys zhaoermii* (1)	China: Medog, Xizang (Tibet)	SWU 0015895	PZ019587	–	PZ022042	–	–	Yuan et al. 2026
67	*Jingophrys zhaoermii* (2)	China: Medog, Xizang (Tibet)	SWU 0015896	PZ019588	–	PZ022043	–	–	Yuan et al. 2026
68	*Jingophrys zhaoermii* (3)	China: Medog, Xizang (Tibet)	SWU 0015897	PZ019589	–	PZ022044	–	–	Yuan et al. 2026

69	*Jingophrys zhaoermii* (4)	China: Medog, Xizang (Tibet)	SWU 0015898	PZ019590	–	PZ022045	–	–	Yuan et al. 2026
70	*Jingophrys* cf. *zhaoermii* (1)	India: Hayuliang-Chaglagom, Anjaw, Arunachal Pradesh	SDBDU 2023.6376	PZ484305	PZ488173	PZ485474	PZ488145	PZ488158	Present study
71	*Jingophrys* cf. *zhaoermii* (2)	India: Kupha, Anjaw, Arunachal Pradesh	SDBDU 2023.6365	PZ484306	PZ488174	–	–	–	Present study
72	*Jingophrys zhoui* (1)	China: Renqinbeng, Medog, Xizang (Tibet)	CIBMT171062	MN963199	MN963186	MN964330	MN984398	–	Shi et al. 2020
73	*Jingophrys zhoui* (2)	China: Renqinbeng, Medog, Xizang (Tibet)	CIBMT171060	MN963200	MN963185	MN964329	MN984397	–	Shi et al. 2020
74	*Jingophrys zhoui* (3)	China: Renqinbeng, Medog, Xizang (Tibet)	CIBMT171053	MN963207	MN963178	MN964322	MN984390	–	Shi et al. 2020
75	*Jingophrys* cand. sp. A (1)	China: Husa, Yunnan	KIZ048517	KX811906	–	KX812151	KX812263	–	Chen et al. 2017
76	*Jingophrys* cand. sp. A (2)	China: Husa, Yunnan	KIZ048518	KX811907	–	KX812152	KX812264	–	Chen et al. 2017
77	*Jingophrys* cand. sp. B	India: Arunachal Pradesh	SDBDU 2022.6292	PZ484307	PZ488175	PZ485475	PZ488146	PZ488159	Present study
	**Genus *Xenophrys***			16S	12SV16S	COI	RAG-1	CXCR-4	
78	*Xenophrys ancrae*	India: Changlang, Arunachal Pradesh	SDBDU 2009.727	KY022318	KY022318	MH647531	KY022350	KY022328	Mahony et al. 2018
79	*Xenophrys apatani*	India: Tale Valley WLS, Arunachal Pradesh	ZSI/V/A/NERC/1370 (“NE3”)	MH423746	–	–	–	–	Saikia et al. 2024
80	*Xenophrys auralensis*	Cambodia: Aural, Kampong Speu	NCSM 79599	KX811807	–	–	–	–	Chen et al. 2017
81	*Xenophrys awuh*	India: Kohima, Nagaland	SDBDU 2007.111	KY022320	KY022320	–	KY022363	KY022341	Mahony et al. 2017
82	*Xenophrys dehongensis*	China: Yingjiang, Yunnan	SYS a003443	MH406746	MH406479	MH406204	MH404980	–	Liu et al. 2018;
83	*Xenophrys dzukou*	India: Kohima, Nagaland	BNHS 6072	KY022324	KY022324	–	KY022362	KY022340	Mahony et al. 2017
84	*Xenophrys flavipunctata*	India: East Khasi Hills, Meghalaya	SDBDU 2009.297	KY022307	KY022307	MH647536	KY022352	KY022330	Mahony et al. 2018
85	*Xenophrys glandulosa*	China: Mt. Gaoligong, Yunnan	SYS a003757	MH406754	MH406487	MH406213	MH404989	–	Liu et al. 2018
86	*Xenophrys himalayana*	India: West Kameng, Arunachal Pradesh	BNHS 6050 / SDBDU 2009.1227	MH647526	MH647526	–	MH647554	MH647538	Mahony et al. 2018
87	*Xenophrys lancangica*	China: Mengla, Yunnan	SYS a002961	MH406728	MH406461	MH406180	MH404954	–	Liu et al. 2018
88	*Xenophrys lekaguli*	Thailand: Mueang Sa Kaeo, Sa Kaeo	FMNH 265955	KY022214	KY022214	–	KY022241	KY022177	Mahony et al. 2017
89	*Xenophrys major*	India: Kohima, Nagaland	SDBDU 2007.229	MH647514	MH647514	–	MH647550	MH647540	Mahony et al. 2018
90	*Xenophrys mangshanensis*	China: Mt. Nankun/ Mt. Nanling, Guangdong	SYS a002025	KJ560372	MH406384	MH406091	MH404859	–	Wang et al. 2014; Liu et al. 2018
91	*Xenophrys* cf. *maosonensis*	Vietnam: Tam Dao, Vinh Phuc	ROM 16679	KX811784	–	KX812081	KX812196	–	Chen et al. 2017
92	*Xenophrys medogensis*	China: Medog, Xizang	SYS a002932	MH406725	MH406458	MH406177	MH404950	–	Liu et al. 2018
93	*Xenophrys megacephala*	India: Ri Bhoi, Meghalaya	ZSI A 11213	KY022315	KY022315	MH647533	KY022357	KY022335	Mahony et al. 2018
94	*Xenophrys monticola*	India: Darjeeling, West Bengal	SDBDU 2011.1047	KY022312	KY022312	–	KY022358	KY022336	Mahony et al. 2017
95	*Xenophrys numhbumaeng*	India: Tamenglong, Manipur	BNHS 6075	KY022316	KY022316	–	KY022361	KY022339	Mahony et al. 2017

96	*Xenophrys oreocrypta*	India: West Garo Hills, Meghalaya	BNHS 6046	KY022306	KY022306	–	KY022351	KY022329	Mahony et al. 2017
97	*Xenophrys oropedion*	India: East Khasi Hills, Meghalaya	SDBDU 2009.299	KY022317	KY022317	MH647534	KY022360	KY022338	Mahony et al. 2018
98	*Xenophrys pangdaensis*	China: Pangda, Yadong, Yadong, Tibet	YBU21248	OR026569	–	OR026034	–	–	Shu et al. 2023
99	*Xenophrys parva*	China: Mengla/Jinghong, Yunnan	SYS a003042	MH406737	MH406470	MH406189	MH404964	–	Liu et al. 2018
100	*Xenophrys periosa*	India: East Siang, Arunachal Pradesh	BNHS 6055 / SDBDU 2009.793	MH647522	MH647522	MH647529	MH647553	MH647537	Mahony et al. 2018
101	*Xenophrys robusta*	India: Darjeeling, West Bengal	SDBDU 2011.1057	KY022314	KY022314	MH647535	KY022365	KY022343	Mahony et al. 2018
102	*Xenophrys serchhipii*	India: North Tripura, Tripura	SDBDU 2009.612	KY022323	KY022323	MH647532	KY022366	KY022344	Mahony et al. 2018
103	*Xenophrys takensis*	Thailand: Khlong Lan, Kampaeng	FMNH 261711	KY022215	KY022215	–	KY022246	KY022183	Mahony et al. 2017
104	*Xenophrys tongbiguanensis*	China: Tongbiguan Provincial Nature Reserve, Yunnan	KIZ 058786	PV820619	–	–	–	–	Wu et al. 2025
105	*Xenophrys truongsonensis*	Vietnam: Nam Tra My, Quang Nam	AMCC 106454 / AMNH 163666	MH647576	MH647576	–	MH647585	MH647581	Mahony et al. 2018
106	*Xenophrys yingjiangensis*	China: Tongbiguan Provincial Nature Reserve, Yunnan	KIZ 053848	PP989317	–	–	–	–	Wu et al. 2025
107	*Xenophrys zhangi*	China: Nyalam, Xizang	KIZ 014278	KX811765	–	KX812084	KX812200	–	Chen et al. 2017
108	*Xenophrys zunhebotoensis*	India: Zunheboto, Nagaland	CES 19909	KY022322	KY022322	–	KY022367	KY022345	Mahony et al. 2017
	**Genus *Megophrys***			16S	12SV16S	COI	RAG-1	CXCR-4	
109	*Megophrys acehensis*	Indonesia: Aceh, Sumatra	MZB Amph 26098	MT710708	–	–	–	–	Munir et al. 2021
110	*Megophrys lancip*	Indonesia: Bengkulu, Sumatra	ENS 7577	KX773567	–	–	KY022228	KY022164	Mahony et al. 2017
111	*Megophrys montana*	Indonesia: Jawa Barat, Java	UTA A-53736, ENS 7381	KY022193	KY022193	–	KY022224	KY022160	Mahony et al. 2017
112	*Megophrys parallela*	Indonesia: West Sumatra, Sumatra	RMAS 022	KY679898	–	–	–	–	Munir et al. 2018
113	*Megophrys selatanensis*	Indonesia: Lampung, Sumatra	MZB Amph 22411	MT710704	–	–	–	–	Munir et al. 2021
	**Genus *Pelobatrachus***			16S	12SV16S	COI	RAG-1	CXCR-4	
114	*Pelobatrachus baluensis*	Malaysia: Mt. Kinabalu, Sabah	IRSNB 15926	DQ642121	DQ642146	–	KY022222	KY022158	Mahony et al. 2017
115	*Pelobatrachus edwardinae*	Malaysia: Bintulu, Sarawak	FMNH 273694	KX811918	–	KX812050	KX812168	–	Chen et al. 2017
116	*Pelobatrachus kalimantanensis*	Malaysia: Crocker, Tenom, Sabah	KUHE 53577	AB719248	AB719248	–	–	–	Hamidy et al. 2012
117	*Pelobatrachus kobayashii*	Malaysia: Gunung Kinabalu National Park, Sabah	UNIMAS 8148	KJ831313	–	–	–	–	Oberhummer et al. 2014
118	*Pelobatrachus ligayae*	Philippines: Palawan	KU KUH309095	KY022192	KY022192	–	KY022223	KY022159	Mahony et al. 2017

119	*Pelobatrachus nasutus*	Malaysia	FMNH 231281	KY022186	KY022186	–	KY022226	KY022162	Mahony et al. 2017
120	*Pelobatrachus stejnegeri*	Philippines: Mindanao	FMNH 250842	KY022190	KY022190	–	KY022227	KY022163	Mahony et al. 2017
	**Genus *Ophryophryne***			16S	12SV16S	COI	RAG-1	CXCR-4	
121	*Ophryophryne elfina*	Vietnam: Chu Yang Sin, Dak Lak	ZMMU NAP-02658	KY425389	KY425389	–	–	–	Poyarkov et al. 2017
122	*Ophryophryne gerti*	Vietnam: Tra Don Commune, Tra My, Quang Nam	AMNH 163668 / AMCC 106456	KY022201	KY022201	–	KY022231	KY022167	Mahony et al. 2017
123	*Ophryophryne hansi*	Vietnam: Thua Thien Hue	AMNH 169285	KY022204	KY022204	KX812155	KY022229	KY022165	Mahony et al. 2017; Chen et al. 2017
124	*Ophryophryne microstoma*	Vietnam: Van Ban, Lao Cai	AMNH 168682	KY022199	KY022199	–	KY022233	KY022169	Mahoney et al. 2017
125	*Ophryophryne poilani*	Vietnam: A Luoi, Thua Tien Hue	AMNH 169287	KY022202	KY022202	–	KY022232	KY022168	Mahony et al. 2017
126	*Ophryophryne synoria*	Cambodia: O'Reang, Mondolkiri	FMNH 262778	KY022198	KY022198	–	KY022235	KY022171	Mahony et al. 2017
	**Genus *Boulenophrys***			16S	12SV16S	COI	RAG-1	CXCR-4	
127	*Boulenophrys acuta*	China: Fengkai, Guangdong	SYS a002266	KJ579119	MF667834	MH406122	MH404890	–	Liu et al. 2018
128	*Boulenophrys angka*	Thailand: Doi Inthanon, Chiang Mai	KIZ 040591	MN508052	–	–	–	–	Wu et al. 2019
129	*Boulenophrys anlongensis*	China: Guizhou, Anlong	CIB AL20190531018	MT823184	–	MT823261	–	–	Li et al. 2020
130	*Boulenophrys baishanzuensis*	China: Zhejiang, Qingyuan	CIB QY20200719002	MW001151	–	MT998292	–	–	Wu et al. 2020
131	*Boulenophrys baolongensis*	China: Wushan, Chongqing	KIZ 019216	KX811813	–	KX812093	KX812202	–	Chen et al. 2017
132	*Boulenophrys binchuanensis*	China: Mt. Jizu, Yunnan	KIZ 019441	KX811849	–	KX812112	KX812219	–	Chen et al. 2017
133	*Boulenophrys binglingensis*	China: Mt. Wawu/Hongya, Sichuan	SYS a005313	MH406892	MH406625	MH406354	MH405137	–	Liu et al. 2018
134	*Boulenophrys boettgeri*	China: Mt. Wuyi, Fujian	SYS a004149	MF667878	MF667847	MH406247	MH405026	–	Liu et al. 2018
135	*Boulenophrys brachykolos*	China: Hong Kong	SYS a002258	KJ560403	MF667851	MH406120	MH404888	–	Liu et al. 2018
136	*Boulenophrys caobangensis*	Vietnam: Nguyen Binh, Cao Bang	IEBR 4385	LC483945	–	–	–	–	Nguyen et al. 2020
137	*Boulenophrys caudoprocta*	China: Sangzhi, Hunan	SYS a004293	MH406796	MH406529	MH406258	MH405037	–	Liu et al. 2018
138	*Boulenophrys changyangensis*	China: Changyang, Hubei	MT CY20220806005	PV768530	–	PV768629	–	–	Li et al. 2025
139	*Boulenophrys cheni*	China: Mt. Jinggang, Jiangxi	SYS a004050	MF667873	MF667840	MH406241	MH405020	–	Liu et al. 2018
140	*Boulenophrys chishuiensis*	China: Mt. Emei, Sichuan	SYS a005307	MH406888	MH406621	MH406350	MH405133	–	Liu et al. 2018
141	*Boulenophrys congjiangensis*	China: Mt. Jiuwan, Guangxi	SYS a005134	MH406880	MH406613	MH406342	MH405125	–	Liu et al. 2018
142	*Boulenophrys daiyunensis*	China: Mt. Daiyun, Fujian	SYS a001733	MH406643	MH406372	MH406079	MH404847	–	Liu et al. 2018
143	*Boulenophrys dalaolingensis*	China: Dalaoling Nature Reserve, Hubei	NNUYC240510	PV816347	–	PV814946	–	–	Wang et al. 2025
144	*Boulenophrys daoji*	China: Fenghua, Zhejiang	SYS a004089	MH406783	MH406516	MH406243	MH405022	–	Liu et al. 2018
145	*Boulenophrys daweimontis*	China: Mt. Dawei, Yunnan	KIZ 048997	KX811867	–	KX812125	KX812248	–	Chen et al. 2017
146	*Boulenophrys daxuemontis*	China: Junlian, Mt. Daxue, Sichuan	CIB JL20230630024	PV469383	–	PV480507	–	–	Liu et al. 2025
147	*Boulenophrys dongguanensis*	China: Dongguan, Guangdong	SYS a001974	MH406648	MH406377	MH406084	MH404852	–	Liu et al. 2018

148	*Boulenophrys dupanglingensis*	China: Dupangling National Nature Reserve, Hunan	HUNU 22SA05	OP548599	–	OP550328	–	–	Xiao et al. 2025
149	*Boulenophrys elongata*	China: Mt. Lianhua, Huizhou, Guangdong	GEP a155	OR601595	OR601600	OR597101	–	–	Zeng et al. 2024
150	*Boulenophrys fanjingmontis*	China: Mt. Fanjing, Guizhou	SYS a004350	MH406808	MH406541	MH406270	MH405049	–	Liu et al. 2018
151	*Boulenophrys fansipanensis*	Vietnam: Sapa, Lao Cai	VNMN 2018.01	MH514886	–	MW086544	–	–	Tapley et al. 2020
152	*Boulenophrys fengshunensis*	China: Mt. Tongguzhang, Guangdong	SYS a004724	MH406848	MH406581	MH406310	MH405091	–	Liu et al. 2018
153	*Boulenophrys frigida*	Vietnam: Bat Xat, Lao Cai	AMS R186131	MT364279	–	MW086550	–	–	Tapley et al. 2020
154	*Boulenophrys gaolanensis*	China: Gaolan Island, Zhuhai, Guangdong	SYS a009225	PQ217847	–	PQ218778	–	–	Song et al. 2024
155	*Boulenophrys gutu*	China: Mt. Hengshan, Nanyue, Hengyang, Hunan	CIB NY20240402004	PX369595	–	PX367389	–	–	Kuang et al. 2025
156	*Boulenophrys hengshanensis*	China: Mt. Hengshan, Nanyue, Hengyang, Hunan	CSUFT HS210608	ON209289	–	OQ910104	–	–	Qian et al. 2023
157	*Boulenophrys hoanglienensis*	Vietnam: Sapa, Lao Cai	VNMN 07034	MH514890	–	MW086547	–	–	Tapley et al. 2020
158	*Boulenophrys huangniushiensis*	China: Huangniushi station, Jiulianshan National Nature Reserve, Jiangxi	SYS a009331	PX121631	–	PX121627	–	–	Wang et al. 2025
159	*Boulenophrys* cf. *huangshanensis*	China: Mt. Huangshan, Anhui	SYS a002702	MF667882	MF667853	MH406160	MH404928	–	Liu et al. 2018
160	*Boulenophrys hungtai*	China: Jiedong, Guangdong	SYS a008576	OL635597	–	OL634864	–	–	Wang et al. 2022
161	*Boulenophrys insularis*	China: Nan'ao, Guangdong	SYS a002168	MH406662	MH406392	MH406102	MH404870	–	Liu et al. 2018
162	*Boulenophrys jiangi*	China: Youyang, Chongqing	KIZ 03076	KX811911	–	KX812148	KX812256	–	Chen et al. 2017
163	*Boulenophrys jingdongensis*	China: Mt. Wuliang, Yunnan	SYS a003928	MH406773	MH406506	MH406232	MH405009	–	Liu et al. 2018
164	*Boulenophrys jinggangensis*	China: Mt. Sifang, Hunan	SYS a004824	MH406857	MH406590	MH406319	MH405100	–	Liu et al. 2018
165	*Boulenophrys jiulianensis*	China: Mt. Nankun, Guangdong	SYS a004011	MH406778	MH406511	MH406237	MH405016	–	Liu et al. 2018
166	*Boulenophrys kuatunensis*	China: Mt. Wuyi, Jiangxi	SYS a003449	MF667881	MF667850	MH406206	MH404982	–	Liu et al. 2018
167	*Boulenophrys leishanensis*	China: Mt. Leigong, Guizhou	SYS a002214	MH406674	MH406404	MH406114	MH404882	–	Liu et al. 2018
168	*Boulenophrys liboensis*	China: Libo, Guizhou	GNUG:20150813001	MF285253	MF285242	–	–	–	Zhang et al. 2017
169	*Boulenophrys lichun*	China: Ningde, Fujian	GEP a214	PQ309138	–	PQ300669	–	–	Lin et al. 2024
170	*Boulenophrys lini*	China, Mt. Jinggang, Jiangxi	SYS a002382	KJ560414	KJ560439	MH406136	MH404904	–	Wang et al. 2014; Liu et al. 2018
171	*Boulenophrys lishuiensis*	China: Liandu, Zhejiang	SYS a008445	OQ180984	–	OQ180872	–	–	Lyu et al. 2023
172	*Boulenophrys lushanensis*	China: Lushan National Nature Reserve, Jiangxi	JXNU 21042803	PX096357	–	PX096339	–	–	Shen et al. 2025
173	*Boulenophrys lushuiensis*	China: Tengchong, Yunnan	SYS a003785	MH406756	MH406489	MH406215	MH404991	–	Liu et al. 2018
174	*Boulenophrys minor*	China: Dujiangyan, Sichuan	SYS a003212	MF667865	MF667828	MH406197	MH404972	–	Wang et al. 2014; Liu et al. 2018
175	*Boulenophrys mirabilis*	China: Lingui, Guangxi	SYS a002289	MH406681	MH406414	MH406127	MH404895	–	Liu et al. 2018
176	*Boulenophrys mufumontana*	China: Mt. Mufu, Hunan	SYS a006419	MK524107	–	MK524138	–	–	Wang et al. 2019
177	*Boulenophrys nankunensis*	China: Mt. Nankun, Guangdong	SYS a004503	MH406824	MH406557	MH406286	MH405065	–	Liu et al. 2018
178	*Boulenophrys nanlingensis*	China: Mt. Nanling, Guangdong	SYS a001962	MH406645	MH406374	MH406081	MH404849	–	Liu et al. 2018
179	*Boulenophrys nebulosa*	China: Mt. Lushan, Jiangxi	JXNU 21042833	PV984449	–	PV984465	–	–	Shen et al. 2025
180	*Boulenophrys obesa*	China: Fengkai, Guangdong	SYS a002275	KJ579123	MH406412	MH406125	MH404893	–	Li et al. 2014; Liu et al. 2018

181	*Boulenophrys ombrophila*	China: Renhua, Guangdong	SYS a001976	MH406650	MH406379	MH406086	MH404854	–	Liu et al. 2018
182	*Boulenophrys omeimontis*	China: Mt. Emei, Sichuan	SYS a005301	MH406887	MH406620	MH406349	MH405132	–	Liu et al. 2018
183	*Boulenophrys palpebralespinosa*	Laos: Phou Dendin, Phongsaly; Vietnam: Pu Hu, Thanh Hoa	FMNH 258098	KY022209	KY022209	–	KY022238	KY022174	Mahony et al. 2017
184	*Boulenophrys pepe*	China: Lianshan Bijiashan Nature Reserve, Lianshan Zhuang and Yao, Qingyuan, Guangdong	GEP a207	PQ131151	–	PQ130479	–	–	Wang et al. 2024
185	*Boulenophrys puningensis*	China: Puning, Guangdong	SYS a005770	OL635585	–	OL634853	–	–	Wang et al. 2022
186	*Boulenophrys qianbeiensis*	China: Kuankuoshui, Guizhou	KIZ YPX28756	KX811855	–	KX812120	KX812231	–	Chen et al. 2017
187	*Boulenophrys rubrimera*	China: Jinping, Yunnan	KIZ 020425	KX811871	–	KX812123	KX812245	–	Chen et al. 2017
188	*Boulenophrys sangzhiensis*	China: Sangzhi, Hunan	SYS a004313	MH406802	MH406535	MH406264	MH405043	–	Liu et al. 2018
189	*Boulenophrys sanmingensis*	China: Jiangle, Fujian	SYS a002499	MH406699	MH406432	MH406147	MH404915	–	Liu et al. 2018
190	*Boulenophrys shimentaina*	China: Yingde, Guangdong	SYS a002077	MH406655	MH406385	MH406092	MH404860	–	Liu et al. 2018
191	*Boulenophrys shuichengensis*	China: Shuicheng / Dafang, Guizhou	CIBDF202100812001	OK017489	–	OK012066	–	–	Qi et al. 2021
192	*Boulenophrys shunhuangensis*	China: Mt. Mao'er, Guangxi	SYS a002295	MH406684	MH406417	MH406130	MH404898	–	Liu et al. 2018
193	*Boulenophrys spinata*	China: Mt. Leigong, Guizhou	SYS a002226	MH406675	MH406405	MH406115	MH404883	–	Liu et al. 2018
194	*Boulenophrys tongboensis*	China: Mt. Tongbo, Jiangxi	SYS a003227	MH406744	MH406477	MH406201	MH404976	–	Liu et al. 2018
195	*Boulenophrys tuberogranulata*	China: Sangzhi, Hunan	SYS a004310	MH406801	MH406534	MH406263	MH405042	–	Liu et al. 2018
196	*Boulenophrys wugongensis*	China: Mt. Wugong, Jiangxi	SYS a004801	MH406854	MH406587	MH406316	MH405097	–	Liu et al. 2018
197	*Boluenophrys wuliangshanensis*	China: Mt. Wuliang, Yunnan	SYS a003924	MH406771	MH406504	MH406230	MH405007	–	Liu et al. 2018
198	*Boulenophrys wushanensis*	China: Shennongjia, Hubei; China: Mt. Guangwu, Sichuan	SYS a003009	MH406733	MH406466	MH406185	MH404960	–	Liu et al. 2018
199	*Boulenophrys xiangnanensis*	China: Shuangpai, Hunan	SYS a002874	MH406713	MH406446	MH406165	MH404938	–	Liu et al. 2018
200	*Boulenophrys xianjuensis*	China: Mt. Dapan, Zhejiang	SYS a002668	MH406704	MH406437	MH406154	MH404922	–	Liu et al. 2018
201	*Boulenophrys xuefengmontis*	China: Wugang, Hunan	SYS a004365	MH406814	MH406547	MH406276	MH405055	–	Liu et al. 2018
202	*Boulenophrys yangchunensis*	China: Yangchun Ehuangzhang Provincial Nature Reserve, Yangchun, Guangdong	GEP a170	PX600405	–	PX529371	–	–	Zhao et al. 2025
203	*Boulenophrys yangmingensis*	China: Shuangpai, Hunan	SYS a002888	MH406719	MH406452	MH406171	MH404944	–	Liu et al. 2018
204	*Boulenophrys yaoshanensis*	China: Mt. Dayao, Guangxi	SYS a004878	MH406863	MH406596	MH406325	MH405106	–	Liu et al. 2018
205	*Boulenophrys yezhongensis*	China: Shuicheng, Guizhou	CIB SC20240531005	PV469441	–	PV491551	–	–	Liu et al. 2025
206	*Boulenophrys yingdeensis*	China: Yingde, Guangdong	SYS a005447	MH406898	MH406631	MH406360	MH405143	–	Liu et al. 2018
207	*Boulenophrys youran*	China: Nanshan National Park, Chengbu Miao Autonomous, Shaoyang, Hunan	HUNL XB25061601	PX641336	–	–	–	–	Xiao et al. 2025
208	*Boulenophrys yunkaiensis*	China: Mt. Yunkai, Guangdong	SYS a004638	MH406844	MH406577	MH406306	MH405087	–	Liu et al. 2018
	**Genus *Sarawakiphrys***			16S	12SV16S	COI	RAG-1	CXCR-4	
209	*Sarawakiphrys dringi*	Malaysia: Gunung Mulu National Park, Sarawak	UNIMAS 8948	KJ831316	–	–	–	–	Oberhummer et al. 2014
	**Genus *Grillitschia***			16S	12SV16S	COI	RAG-1	CXCR-4	
210	*Grillitschia aceras*	Thailand: Khao Nan National Park, Nakhon Si Thammarat	KU KUH332489	KY022211	KY022211	x	KY022240	KY022176	Mahony et al. 2017
211	*Grillitschia longipes*	Malaysia: Genting highland	IABHU 21101	AB530656	–	–	–	–	Hasan et al. 2012
	**Genus *Atympanophrys***			16S	12SV16S	COI	RAG-1	CXCR-4	

212	*Atympanophrys gigantica*	China: Jingdong, Yunnan	SYS a003933	MH406775	MH406508	MH406234	MH405011	MH450010	Liu et al. 2018
213	*Atympanophrys nankiangensis*	China: Nanjiang, Sichuan	CIB XM835	EF397243	EF397243	–	EF397281	–	Fu et al. 2007
214	*Atympanophrys shapingensis*	China: Mt. Wawu, Sichuan	SYS a005310	MH406890	MH406623	MH406352	MH405135	–	Liu et al. 2018
215	*Atympanophrys wawuensis*	China: Mt. Wawu, Sichuan	SYS a005311	MH406891	MH406624	MH406353	MH405136	–	Liu et al. 2018
	**Genus *Brachytarsophrys***			16S	12SV16S	COI	RAG-1	CXCR-4	
216	*Brachytarsophrys carinense*	Myanmar: Khotama Camp, Yephyu, Dawei, Tanintharyi	CAS 243791	KY022197	KY022197	–	KY022219	KY022155	Mahony et al. 2017
217	*Brachytarsophrys chuannanensis*	China: Hejiang, Sichuan	SYS a004927	MH406902	MH406636	MH406365	MH405148	–	Liu et al. 2018
218	*Brachytarsophrys feae*	China: Jingdong, Yunnan	SYS a003913	MH406900	MH406634	MH406363	MH405146	MH450012	Liu et al. 2018
219	*Brachytarsophrys guilinensis*	China: Guilin, Guangxi	GXNU YU000904	PV819240	–	PV817820	–	–	Wu et al. 2025
220	*Brachytarsophrys intermedia*	Laos: Xe Sap National Biodiversity Conservation Area, Kaleum, Xe Kong	FMNH 258093	KY022196	KY022196	–	KY022221	KY022157	Mahony et al. 2017
221	*Brachytarsophrys orientalis*	China: Mt. Jiulian, Jiangxi	SYS a004225	OQ180989	OQ180955	MT162625	–	–	Li et al. 2020; Lyu et al. 2023
222	*Brachytarsophrys platyparietus*	China: Dayao, Yunnan	SYS a005919	OQ180990	OQ180956	MT162633	–	–	Li et al. 2020; Lyu et al. 2023
223	*Brachytarsophrys popei*	China: Yanling, Hunan	SYS a001864	KM504256	MH406632	MH406361	MH405144	–	Liu et al. 2018
224	*Brachytarsophrys qiannanensis*	China: Libo, Qiannan, Guizhou	CIB LB20210806054	OK104100	–	OK104053	–	–	Li et al. 2022
225	*Brachytarsophrys wenshanensis*	China: Wenshan National Nature Reserve, Xiaoqiaogou, Xichou, Wenshan, Yunnan	SWU0004072	PP153337	–	PP155195	–	–	He et al. 2024
226	*Brachytarsophrys wui*	China: Lengshui River, Mt. Foding, Shiqian, Guizhou	GIB1972746	PX635279	–	PX568570	–	–	Lyu et al. 2026
	**Subfamily Leptobrachiinae**								
227	*Leptobrachella alpina*	China: Jingdong, Yunnan	SYS a003927	MH406905	MH406639	MH406368	MH405151	–	Liu et al. 2018
228	*Leptobrachella laui*	China: Mt. Wutong, Guangdong	SYS a003471	MH406903	MH406637	MH406366	MH405149	–	Liu et al. 2018
229	*Leptobrachium boringii*	China: Mt. Emei, Sichuan	KIZ YPX37539	KX811930	–	KX812164	KX812282	–	Chen et al. 2017
230	*Leptobrachium sylheticum*	India: Trishna Wildlife Sanctuary, South Tripura, Tripura	SDBDU 2009.490	KY022304	KY022304	–	KY022347	KY022325	Mahony et al. 2017
	**Family Pelobatidae (Outgroup)**								
231	*Pelobates cultripes*	Spain	VUB 0510	AY364363	AY364341	–	AY364201	AY364171	Biju & Bossuyt 2003

Table S2. Localities, geographical coordinates, elevation, and species of *Jingophrys* recorded in the present study and in earlier publications. Localities are arranged longitudinally and organised by country of occurrence. Abbreviation: WLS, Wildlife Sanctuary.

No.	Locality	District / County	State / Province/ Region	Longitude (°E)	Latitude (°N)	Elevation (meters)	Species recorded	Reference
	**INDIA**							
1	Sessni, Eaglenest WLS	West Kameng District	Arunachal Pradesh State	92.4181	27.0474	1250	*Jingophrys vegrandis*	Athreya 2006
2	Sessa Village, Bhalukpong Forest Division	West Kameng District	Arunachal Pradesh State	92.5242	27.0992	1210	*Jingophrys vegrandis*	Present study
3	Sessa Village, Bhalukpong Forest Division	West Kameng District	Arunachal Pradesh State	92.5274	27.1011	1110	*Jingophrys vegrandis*	Mahony et al. 2013
4	Tajgi Village, Ziro Valley	Lower Subansiri District	Arunachal Pradesh State	93.8638	27.6402	1740	*Jingophrys taniitatii* **sp. nov.**<br>*Jingophrys tanogogo* **sp. nov.**<br>*Jingophrys wularsoni* **sp. nov.**	Present study
5	Manipolyang-Pange road	Lower Subansiri District	Arunachal Pradesh State	93.8689	27.5246	1780	*Jingophrys taniitatii* **sp. nov.**	Present study
6	Tarin Tibe Khole Pass, Tale Valley WLS	Lower Subansiri District	Arunachal Pradesh State	93.8805	27.5396	1945	*Jingophrys taniitatii* **sp. nov.**<br>*Jingophrys tanogogo* **sp. nov.**<br>*Jingophrys wularsoni* **sp. nov.**	Present study
7	Hari Village, Ziro Valley	Lower Subansiri District	Arunachal Pradesh State	93.8857	27.5999	1729	*Jingophrys tanogogo* **sp. nov.**	Present study
8	Pange, Tale Valley WLS	Lower Subansiri District	Arunachal Pradesh State	93.8946	27.5443	1925	*Jingophrys taniitatii* **sp. nov.**<br>*Jingophrys tanogogo* **sp. nov.**<br>*Jingophrys wularsoni* **sp. nov.**	Present study
9	Pange, Tale Valley WLS	Lower Subansiri District	Arunachal Pradesh State	93.8976	27.5477	1855	*Jingophrys wularsoni* **sp. nov.**	Saikia & Sinha 2018; Present study
10	Tasi Biddang, Tale Valley WLS	Lower Subansiri District	Arunachal Pradesh State	93.9167	27.5406	2121	*Jingophrys hrdy* **sp. nov.**<br>*Jingophrys wularsoni* **sp. nov.**	Present study
11	Tale Top, Tale Valley WLS	Lower Subansiri District	Arunachal Pradesh State	93.9539	27.5338	2344	*Jingophrys hrdy* **sp. nov.**	Present study
12	Kebang	East Siang District	Arunachal Pradesh State	94.9576	28.2773	305	*Jingophrys siangensis* **sp. nov.**	Present study
13	Yembung	East Siang District	Arunachal Pradesh State	94.9865	28.2588	314	*Jingophrys siangensis* **sp. nov.**	Present study
14	Pangin	East Siang District	Arunachal Pradesh State	94.9870	28.2628	304	*Jingophrys siangensis* **sp. nov.**	Present study
15	Tiwari Gaon	Lower Dibang Valley District	Arunachal Pradesh State	95.8067	28.2115	954	*Jingophrys* cf. *yeae*	Present study
16	Tiwari Gaon	Lower Dibang Valley District	Arunachal Pradesh State	95.8225	28.2193	1455	*Jingophrys* cf. *yeae*	Present study
17	Etalin	Dibang Valley District	Arunachal Pradesh State	95.9111	28.6186	714	*Jingophrys* cf. *yeae*	Present study
18	Anini	Dibang Valley District	Arunachal Pradesh State	95.9273	28.8097	1390	*Jingophrys* cf. *yeae*	Present study
19	Hayuliang-Chaglagom	Anjaw District	Arunachal Pradesh State	96.5491	28.1458	780	*Jingophrys* cf. *zhaoermii*	Present study
20	Kupha	Anjaw District	Arunachal Pradesh State	96.4651	28.0826	785	*Jingophrys* cf. *zhaoermii*	Present study
	**CHINA**							

21	Didong Village	Medog County	Tibet / Xizang Autonomous Region	95.1246	29.2251	670	*Jingophrys yeae*	Shi et al. 2020
22	Gelin	Medog County	Tibet / Xizang Autonomous Region	95.1667	29.1833	1530	*Jingophrys pachyproctus*	Hunag and Fei 1981; Shi et al. 2020
23	Beibeng Village	Medog County	Tibet / Xizang Autonomous Region	95.1856	29.2429	870	*Jingophrys yeae*	Shi et al. 2020
24	Gelin Village	Medog County	Tibet / Xizang Autonomous Region	95.2263	29.1518	2589.52	*Jingophrys zhaoermii*	Yuan et al. 2026
25	Maniwong	Medog County	Tibet / Xizang Autonomous Region	–	–	–	*Jingophrys yeae*	Li et al. 2010; Shi et al. 2020
26	Yarang Village	Medog County	Tibet / Xizang Autonomous Region	95.2813	29.2949	795	*Jingophrys yeae*	Li et al. 2010; Shi et al. 2020
27	Medog urban neighborhood	Medog County	Tibet / Xizang Autonomous Region	95.3132	29.3221	907	*Jingophrys yeae*	Shi et al. 2020
28	Yadong Village, Medog city suburb	Medog County	Tibet / Xizang Autonomous Region	95.3440	29.3265	1073	*Jingophrys yeae*	Shi et al. 2020
29	Bari Village	Medog County	Tibet / Xizang Autonomous Region	95.3602	29.3295	1780	*Jingophrys* cf. *pachyproctus* *Jingophrys yeae*	Shi et al. 2020
30	Renqingbeng Temple	Medog County	Tibet / Xizang Autonomous Region	95.3617	29.3048	2003	*Jingophrys* cf. *pachyproctus* *Jingophrys zhoui*	Shi et al. 2020
31	Xiaolangsu Village, Tongbiguan Town	Yingjiang County	Yunnan Province	97.5713	24.5009	700	*Jingophrys feii*	Yang et al. 2018; Lyu et al. 2023
32	Nabang Town	Yingjiang County	Yunnan Province	97.5995	24.7134	760	*Jingophrys feii*	Lyu et al. 2023
33	Husa	Dehong Dai and Jingpo Autonomous Prefecture	Yunnan Province	98.5356	24.2037	1675	*Jingophrys* cand. sp. A	Chen et al. 2017

Table S3. Uncorrected intra- and interspecific pairwise genetic p-distances among *Jingophrys* members, based on mitochondrial 16S rRNA. Values are reported as mean and standard deviation with minimum and maximum inside parentheses, and expressed as a percentage. The number of individuals for each taxon are referenced in Table S1.

***Jingophrys vegrandis* species group**												
	J. feii	*J. siangensis* **sp. nov.**	*J. tanogogo* **sp. nov.**	*J. vegrandis*	*J. yeae* sensu lato	*J. yeae* sensu stricto	*J.* cf. *yeae*	*J. zhaoermii* sensu lato	*J.* cf. *zhaoermii*	*J. zhaoermii* sensu stricto	*J. zhoui*	*J.* cand. sp. A
J. feii	0											
J. siangensis **sp. nov.**	8.1 ± 0.0<br>(8.1 – 8.1)	0										
J. tanogogo **sp. nov.**	8.7 ± 0.1<br>(8.7 – 8.9)	7.3 ± 0.1<br>(7.3 – 7.5)	0.1 ± 0.1<br>(0.0 – 0.2)									
J. vegrandis	7.2 ± 0.1<br>(7.1 – 7.3)	5.9 ± 0.1<br>(5.7 – 6.0)	4.3 ± 0.1<br>(4.1 – 4.6)	0.1 ± 0.1<br>(0.0 – 0.2)								
J. yeae sensu lato	8.1 ± 0.2<br>(7.7 – 8.3)	7.0 ± 0.2<br>(6.9 – 7.5)	5.7 ± 0.2<br>(5.3 – 6.5)	4.2 ± 0.4<br>(4.0 – 6.9)	0.7 ± 0.8<br>(0.0 – 2.0)							
J. yeae sensu stricto	8.2 ± 0.1<br>(8.1 – 8.3)	7.0 ± 0.1<br>(6.9 – 7.1)	5.6 ± 0.1<br>(5.3 – 5.9)	4.2 ± 0.4<br>(4.0 – 6.9)	–	0.05 ± 0.1<br>(0.0 – 0.4)						
J. cf. *yeae*	7.8 ± 0.1<br>(7.7 – 7.9)	7.3 ± 0.2<br>(7.1 – 7.5)	6.0 ± 0.3<br>(5.7 – 6.5)	4.4 ± 0.2<br>(4.2 – 4.6)	–	1.6 ± 0.1<br>(1.6 – 2.0)	0.6 ± 0.4<br>(0.0 – 1.0)					
J. zhaoermii sensu lato	6.7 ± 0.3<br>(6.3 – 6.9)	6.7 ± 0.0<br>(6.7 – 6.7)	4.6 ± 0.1<br>(4.5 – 4.7)	3.4 ± 0.2<br>(3.2 – 3.8)	4.8 ± 0.1<br>(4.6 – 5.2)	4.8 ± 0.1<br>(4.6 – 5.0)	4.9 ± 0.1<br>(4.8 – 5.2)	0.8 ± 0.8<br>(0.0 – 1.6)				
J. cf. *zhaoermii*	6.3 ± 0.0<br>(6.3 – 6.3)	6.7 ± 0.0<br>(6.7 – 6.7)	4.6 ± 0.1<br>(4.5 – 4.7)	3.7 ± 0.1<br>(3.6 – 3.8)	4.8 ± 0.1<br>(4.6 – 5.2)	4.8 ± 0.1<br>(4.6 – 5.0)	5.0 ± 0.1<br>(5.0 – 5.2)	–	0			
J. zhaoermii sensu stricto	6.9 ± 0.0<br>(6.9 – 6.9)	6.7 ± 0.0<br>(6.7 – 6.7)	4.6 ± 0.1<br>(4.5 – 4.7)	3.3 ± 0.1<br>(3.2 – 3.4)	–	4.8 ± 0.1<br>(4.6 – 5.0)	4.8 ± 0.1<br>(4.8 – 5.0)	–	1.6 ± 0.0<br>(1.6 – 1.6)	0		
J. zhoui	8.2 ± 0.1<br>(8.1 – 8.3)	8.2 ± 0.1<br>(8.1 – 8.3)	6.0 ± 0.1<br>(5.9 – 6.1)	4.6 ± 0.1<br>(4.5 – 4.7)	6.3 ± 0.1<br>(6.0 – 6.5)	6.3 ± 0.1<br>(6.0 – 6.5)	6.4 ± 0.1<br>(6.2 – 6.5)	5.0 ± 0.1<br>(4.8 – 5.1)	4.9 ± 0.1<br>(4.8 – 4.9)	5.1 ± 0.1<br>(4.9 – 5.1)	0	
J. cand. sp. A	6.7 ± 0.0<br>(6.7 – 6.7)	5.3 ± 0.0<br>(5.3 – 5.3)	6.9 ± 0.1<br>(6.9 – 7.1)	5.3 ± 0.1<br>(5.7 – 6.0)	6.4 ± 0.1<br>(6.2 – 6.8)	6.4 ± 0.1<br>(6.3 – 6.5)	6.4 ± 0.3<br>(6.2 – 6.8)	5.1 ± 0.0<br>(5.1 – 5.1)	5.1 ± 0.0<br>(5.1 – 5.1)	5.1 ± 0.0<br>(5.1 – 5.1)	6.6 ± 0.1<br>(6.5 – 6.7)	0
***Jingophrys pachyproctus* species group**												
	J. hrdy **sp. nov.**	*J. pachyproctus*	*J. taniitatii* **sp. nov.**	*J. wularsoni* **sp. nov.**	*J.* cand. sp. B							
J. hrdy **sp. nov.**	0.1 ± 0.1<br>(0.0 – 0.2)											
J. pachyproctus	7.7 ± 0.1<br>(7.5 – 7.9)	0.2 ± 0.2<br>(0.0 – 0.6)										
J. taniitatii **sp. nov.**	5.0 ± 0.1<br>(4.9 – 5.1)	8.5 ± 0.1<br>(8.3 – 8.7)	0									
J. wularsoni **sp. nov.**	6.8 ± 0.1<br>(6.7 – 6.9)	3.5 ± 0.1<br>(3.4 – 3.7)	8.3 ± 0.0<br>(8.3 – 8.3)	0								

J. cand. sp. B	5.6 ± 0.1<br>(5.5 – 5.7)	7.9 ± 0.1<br>(7.7 – 8.1)	4.4 ± 0.0<br>(4.4 – 4.4)	7.7 ± 0.0<br>(7.7 – 7.7)	–							
Jingophrys vegrandis **species group vs.** ***Jingophrys pachyproctus*** **species group**												
	J. hrdy **sp. nov.**	*J. pachyproctus*	*J. taniitatii* **sp. nov.**	*J. wularsoni* **sp. nov.**	*J.* cand. sp. B							
J. feii	10.1 ± 0.0<br>(10.1 – 10.1)	11.0 ± 0.2<br>(10.7 – 11.3)	10.3 ± 0.0<br>(10.3 – 10.3)	11.1 ± 0.0<br>(11.1 – 11.1)	11.9 ± 0.0<br>(11.9 – 11.9)							
J. siangensis **sp. nov.**	10.2 ± 0.1<br>(10.1 – 10.3)	10.0 ± 0.1<br>(9.9 – 10.3)	10.6 ± 0.0<br>(10.6 – 10.6)	9.7 ± 0.0<br>(9.7 – 9.7)	11.1 ± 0.0<br>(11.1 – 11.1)							
J. tanogogo **sp. nov.**	9.2 ± 0.1<br>(9.1 – 9.5)	10.3 ± 0.2<br>(10.1 – 10.9)	9.5 ± 0.0<br>(9.5 – 9.5)	9.7 ± 0.1<br>(9.7 – 9.9)	9.5 ± 0.1<br>(9.5 – 9.7)							
J. vegrandis	9.2 ± 0.2<br>(8.9 – 9.6)	9.8 ± 0.3<br>(9.3 – 10.4)	9.2 ± 0.2<br>(9.0 – 9.4)	9.4 ± 0.2<br>(9.1 – 9.6)	9.2 ± 0.2<br>(8.9 – 9.4)							
J. yeae sensu lato	9.6 ± 0.3<br>(9.3 – 10.1)	10.1 ± 0.2<br>(9.5 – 10.7)	10.4 ± 0.1<br>(10.4 – 10.6)	10.1 ± 0.1<br>(9.9 – 10.3)	10.2 ± 0.2<br>(10.0 – 10.6)							
J. yeae sensu stricto	9.4 ± 0.1<br>(9.3 – 9.7)	10.2 ± 0.2<br>(9.9 – 10.7)	10.4 ± 0.1<br>(10.4 – 10.6)	10.1 ± 0.1<br>(9.9 – 10.3)	10.2 ± 0.1<br>(10.0 – 10.4)							
J. cf. *yeae*	9.9 ± 0.1<br>(9.7 – 10.1)	10.1 ± 0.2<br>(9.5 – 10.5)	10.5 ± 0.1<br>(10.4 – 10.6)	10.1 ± 0.1<br>(9.9 – 10.1)	10.5 ± 0.1<br>(10.4 – 10.6)							
J. zhaoermii sensu lato	7.8 ± 0.6<br>(6.9 – 8.3)	9.6 ± 0.2<br>(9.3 – 10.1)	8.9 ± 0.1<br>(8.7 – 8.9)	8.6 ± 0.1<br>(8.5 – 8.8)	9.1 ± 0.0<br>(9.1 – 9.1)							
J. cf. *zhaoermii*	7.0 ± 0.1<br>(6.9 – 7.1)	9.7 ± 0.2<br>(9.5 – 10.1)	8.7 ± 0.0<br>(8.7 – 8.7)	8.8 ± 0.0<br>(8.8 – 8.8)	9.1 ± 0.0<br>(9.1 – 9.1)							
J. zhaoermii sensu stricto	8.2 ± 0.1<br>(8.1 – 8.3)	9.5 ± 0.2<br>(9.3 – 9.9)	8.9 ± 0.0<br>(8.9 – 8.9)	8.5 ± 0.0<br>(8.5 – 8.5)	9.1 ± 0.0<br>(9.1 – 9.1)							
J. zhoui	8.9 ± 0.1<br>(8.9 – 9.1)	10.6 ± 0.1<br>(10.4 – 10.9)	9.1 ± 0.0<br>(9.1 – 9.1)	10.3 ± 0.0<br>(10.3 – 10.3)	9.1 ± 0.0<br>(9.1 – 9.1)							
J. cand. sp. A	8.2 ± 0.1<br>(8.1 – 8.3)	9.4 ± 0.1<br>(9.3 – 9.7)	9.3 ± 0.0<br>(9.3 – 9.3)	8.1 ± 0.0<br>(8.1 – 8.1)	9.7 ± 0.0<br>(9.7 – 9.7)							

Table S4. Comparison among and statistical support for different models of ancestral range reconstructions from BioGeoBEARS. Abbreviations: Dispersal-Extinction-Cladogenesis (DEC); Dispersal-Vicariance Analysis LIKE (DIVALIKE); BayArea LIKE (BAYAREALIKE). Each model was tested with and without founder-event speciation (jump dispersal, J). The Log-Likelihood (LnL), corrected Akaike's Information Criterion (AICc), and AICc model weight were used for model comparison. The best-supported model is indicated in bold face.

Model	Log-Likelihood (LnL)	No. of parameters	Dispersal rate (*d*)	Extinction rate (*e*)	Jump dispersal rate (*j*)	AICc	AICc weight
DEC	-217.1	2	0.0055	1.0e-12	0	438.3	0.048
**DEC+J**	**-213.1**	**3**	**0.0047**	**1.0e-12**	**0.0043**	**432.3**	**0.95**
DIVALIKE	-235.3	2	0.0074	0.0007	0	474.7	5.8e-10
DIVALIKE+J	-224.4	3	0.0053	1.0e-12	0.0076	454.9	1.2e-05
BAYAREALIKE	-248.5	2	0.0043	0.044	0	501	1.1e-15
BAYAREALIKE+J	-235.5	3	0.0034	0.0092	0.01	477.1	1.7e-10

Table S5. Morphometric measurements (range, mean, and standard deviation) for *Jingophrys* species reported in the present study. All measurements are in millimeters (mm). Measurement acronyms are defined in the text.

	Jingophrys hrdy **sp. nov.**		*Jingophrys siangensis* **sp. nov.**		*Jingophrys taniitatii* **sp. nov.**		*Jingophrys tanogogo* **sp. nov.**			
	(males, n = 6)		(males, n = 6)		(males, n = 4)		(males, n = 8)		(females, n = 4)	
	Range	Mean ± SD	Range	Mean ± SD	Range	Mean ± SD	Range	Mean ± SD	Range	Mean ± SD
SVL	30.0–36.2	34.1.0±2.4	28.6–31.3	29.9±0.9	27.4–31.1	28.9.0±1.6	24.9–30.3	27.2±1.5	31.8–35.5	33.4±1.6
HL	10.4–12.4	11.6±0.7	9.5–11.1	10.4±0.5	10.2–11.3	10.7±0.5	8.5–10.4	9.0±0.7	10.1–10.8	10.5±0.3
HW	10.3–12.5	11.5±0.8	9.9–10.6	10.2±0.2	11.0–11.9	11.5±0.4	8.5–10.4	9.0±0.7	10.1–10.8	10.4±0.3
SL	4.1–5.0	4.4±0.3	4.1–4.6	4.3±0.2	3.5–4.1	3.8±0.3	3.1–3.9	3.4±0.2	3.9–4.0	4.0±0.1
EL	3.2–3.7	3.4±0.2	3.4–3.7	3.6±0.1	3.5–3.8	3.6±0.1	2.5–2.9	2.7±0.1	2.7–2.9	2.9±0.1
TYD	2.2–2.6	2.4±0.2	1.5–2.0	1.8±0.2	1.6–2.1	1.8±0.2	1.3–1.8	1.5±0.2	1.9–1.9	1.9±0.0
TYE	1.4–1.9	1.6±0.2	1.6–1.9	1.8±0.1	1.5–1.7	1.6±0.1	1.3–1.9	1.6±0.2	1.8–2.3	2.1±0.2
TYF	2.5–3.7	3.2±0.5	2.3–2.6	2.4±0.1	1.7–2.3	1.9±0.3	1.9–2.2	2.0±0.1	1.7–2.3	2.0±0.3
EN	2.1–2.4	2.2±0.2	1.6–2.1	1.9±0.2	1.4–1.9	1.6±0.2	1.5–2.0	1.7±0.2	1.6–2.0	1.9±0.2
NS	1.9–2.1	2.0±0.1	1.7–1.8	1.8±0.0	1.9–2.0	2.0±0.1	1.2–2.3	1.5±0.4	1.4–1.8	1.6±0.2
IUE	3.5–3.9	3.7±0.2	2.9–3.8	3.3±0.3	3.0–3.4	3.2±0.2	3.2–3.6	3.4±0.2	3.3–3.7	3.5±0.2
UEW	3.0–3.2	3.1±0.1	2.7–4.0	3.1±0.5	3.1–3.5	3.3±0.2	2.2–2.5	2.3±0.1	2.5–2.8	2.7±0.2
IN	3.4–3.9	3.6±0.2	3.2–3.7	3.4±0.2	3.3–3.7	3.4±0.2	2.4–3.1	2.7±0.2	2.8–3.3	3.0±0.2
SP	2.4–2.7	2.6±0.1	2.0–2.4	2.3±0.1	2.4–2.8	2.6±0.2	2.0–2.2	2.1±0.1	2.2–2.6	2.4±0.2
FAL	7.5–8.1	7.9±0.3	7.1–7.6	7.5±0.2	6.5–7.2	6.9±0.3	6.2–7.8	6.8±0.6	7.8–8.2	8.0±0.2
HAL	10.2–10.9	10.5±0.3	8.2–9.0	8.7±0.3	8.5–9.5	9.0±0.4	8.1–10.1	8.8±0.7	9.8–10.5	10.2±0.3
FIL	2.9–3.2	3.1±0.1	2.4–3.0	2.7±0.2	2.7–3.3	2.9±0.3	2.0–2.7	2.2±0.2	2.5–3.0	2.8±0.2
FIIL	4.3–4.9	4.7±0.2	3.7–4.2	4.0±0.2	3.5–4.1	3.8±0.3	3.7–4.8	4.1±0.4	4.8–5.5	5.1±0.3
FIIIL	7.3–7.8	7.5±0.2	5.4–6.4	6.0±0.4	5.7–6.6	6.0±0.4	5.1–6.5	5.7±0.5	6.8–7.4	7.2±0.3
FIVL	4.9–5.7	5.4±0.3	3.5–4.3	4.0±0.3	3.9–4.5	4.1±0.3	3.4–5.0	4.0±0.5	4.5–5.3	4.9±0.4
TL	15.6–17.5	16.5±0.6	13.6–14.6	14.1±0.4	14.5–15.7	15.1±0.6	13.1–14.6	13.3±0.5	13.1–15.2	14.5±1.0
SHL	15.7–17.6	16.7±0.6	14.4–16.4	15.7±0.8	15.3–17.9	16.3±1.1	13.1–15.7	13.6±0.9	14.9–16.8	15.8±0.9
FOL	15.5–17.7	16.4±0.7	11.4–13.5	12.7±0.8	13.5–14.0	13.7±0.2	10.9–12.6	11.4±0.6	13.1–13.8	13.5±0.4
TFOL	23.4–27.6	25.3±1.4	18.8–21.6	20.9±1.1	20.8–23.6	21.7±1.3	17.1–21.7	18.6±1.4	21.5–23.2	22.4±0.9
TIVW	0.5–0.7	0.6±0.1	0.9–1.2	1.1±0.1	0.5–0.9	0.7±0.2	0.1–0.5	0.4±0.1	0.4–0.5	0.5±0.1
TIVF	0.0–0.2	0.1±0.1	1.1–1.3	1.2±0.1	0.3–1.4	0.6±0.5	1.0–1.6	1.3±0.3	1.1–1.4	1.2±0.1

	Jingophrys vegrandis		*Jingophrys wularsoni* **sp. nov.**			*Jingophrys* cf. *yeae*		*Jingophrys* cf. *zhaoermii*		
	(males, n = 6)		(males, n = 9)		(female)	(males, n = 7)		(males, n = 3)		
	Range	Mean ± SD	Range	Mean ± SD	(N = 1)	Range	Mean±SD	Range	Mean±SD	
SVL	26.8–29.9	28.9±1.1	36.4–40.8	38.2±1.6	47.6	24.9–28.0	26.3.0±1.0	22.5–26.5	24.5±2.0	
HL	9.7–10.6	10.2±0.3	12.6–14.5	13.5±0.6	16.1	9.1–9.5	9.3±0.1	9.2–9.8	9.3±0.5	
HW	9.6–10.6	10.2±0.3	12.5–14.5	13.5±0.7	16.4	8.9–9.8	9.3±0.3	7.4–8.3	7.9±0.5	
SL	3.8–4.1	3.9±0.1	5.0–5.5	5.2±0.2	5.7	3.3–3.9	3.5±0.2	3.5–3.7	3.6±0.1	
EL	3.1–3.6	3.3±0.2	3.5–3.9	3.6±0.1	4.3	2.4–2.8	2.6±0.1	2.8–2.9	2.8±0.1	
TYD	1.8–2.0	2.0±0.1	1.6–1.9	1.8±0.1	2.5	1.6–1.9	1.8±0.1	1.3–1.6	1.4±0.2	
TYE	1.1–1.5	1.4±0.2	2.4–2.6	2.5±0.1	2.9	1.2–1.4	1.3±0.1	1.1–1.4	1.2±0.2	
TYF	1.6–2.3	1.9±0.3	3.3–4.2	3.7±0.3	4.5	1.7–2.6	2.2±0.3	2.1–2.6	2.3±0.3	
EN	1.5–2.0	1.7±0.2	2.0–2.4	2.2±0.1	2.2	1.5–1.6	1.6±0.0	1.2–1.3	1.2±0.1	
NS	1.7–2.0	1.9±0.1	2.4–2.7	2.5±0.1	2.7	1.6–1.9	1.7±0.1	1.7–2.1	2.0±0.2	
IUE	3.1–3.5	3.2±0.2	4.3–5.4	4.9±0.3	5.3	3.3–3.8	3.5±0.2	3.3–3.6	3.5±0.2	
UEW	3.0–3.4	3.2±0.1	3.3–3.7	3.6±0.1	4.1	2.3–2.6	2.5±0.1	2.3–2.4	2.3±0.1	
IN	3.1–3.3	3.3±0.1	4.0–4.4	4.2±0.1	4.5	2.8–3.0	2.9±0.1	2.6–3.2	3.0±0.3	
SP	2.2–2.5	2.3±0.1	2.5–3.2	2.7±0.2	3.0	2.0–2.9	2.3±0.3	1.7–2.2	2.0±0.3	
FAL	7.1–7.6	7.3±0.2	8.3–9.7	8.7±0.5	10.5	5.8–6.8	6.2±0.4	4.9–5.5	5.1±0.3	
HAL	9.0–9.4	9.3±0.2	11.0–11.9	11.2±0.3	13.1	7.5–8.5	7.7±0.3	7.4–8.0	7.7±0.3	
FIL	2.6–3.0	2.8±0.2	3.5–4.0	3.8±0.2	4.2	1.9–2.3	2.1±0.2	2.1–2.3	2.2±0.1	
FIIL	3.8–4.6	4.1±0.3	3.7–5.2	4.7±0.5	5.6	3.2–3.7	3.4±0.2	3.4–3.8	3.6±0.2	

FIIIL	6.1–6.6	6.4±0.2	7.7–8.9	8.2±0.5	9.5	4.7–5.5	5.2±0.3	5.3–5.5	5.4±0.1	
FIVL	3.6–4.6	4.1±0.3	5.3–6.4	6.0±0.4	6.7	3.0–3.6	3.2±0.2	3.8–4.0	3.9±0.1	
TL	13.7–14.6	14.3±0.4	17.9–20.3	18.6±0.9	21.0	12.0–13.1	12.4±0.5	12.0–13.3	12.6±0.7	
SHL	14.9–15.9	15.4±0.4	17.5–20.3	18.6±1.0	20.9	13.1–14.0	13.5±0.3	12.9–14.2	13.7±0.7	
FOL	12.0–12.7	12.4±0.3	16.4–17.9	17.0±0.5	20.2	11.0–12.9	11.5±0.7	11.1–12.3	11.8±0.6	
TFOL	19.7–21.7	21.1±0.7	26.2–29.3	27.5±1.2	31.3	17.8–20.1	18.6±0.8	16.9–18.9	18.0±1.0	
TIVW	0.4–0.8	0.6±0.1	0.8–0.9	0.9±0.1	0.8	0.5–0.6	0.5±0.1	0.4–0.5	0.5±0.1	
TIVF	0.9–1.2	1.1±0.1	NA	NA	NA	0.7–0.9	0.8±0.1	0.1–0.1	0.1±0.0	

Table S6. Principal Components Analysis of size-adjusted morphometric variables from males of the *Jingophrys vegrandis* and *Jingophrys pachyproctus* species groups. Values indicate factor loadings and percent variance explained by the five principal components (PC) with eigenvalues > 1. Bold font denotes variables with the highest loadings.

***Jingophrys vegrandis* species group**					
Variable	PC 1	PC 2	PC 3	PC 4	PC 5
SVL	-0.1705503	-0.334186	0.15879855	0.08521808	**-0.335301**
HL/SVL	**0.28602109**	-0.0585474	-0.2853002	0.0486209	-0.1189289
HW/SVL	0.07253603	-0.2569522	-0.1493203	**0.41483627**	0.30909778
SL/HL	-0.0347472	-0.2230303	0.04723474	**-0.452152**	0.16859932
EL/HL	0.01434925	-0.3191534	0.17196609	**-0.3473818**	-0.1070889
TYD/HL	-0.0794427	-0.2624176	-0.1533639	0.21565002	**0.30697985**
TYE/HL	-0.2010818	0.02641559	**0.30014487**	-0.1408283	0.16535711
EN/HL	-0.2486991	-0.1250035	0.26031771	-0.0376335	**0.30781916**
NS/HL	0.14444829	-0.0627273	-0.1339319	**-0.3639934**	0.22831477
IUE/HL	-0.039796	0.37432237	-0.0891351	-0.0711859	**0.43151447**
UEW/HL	0.02098548	**-0.3830015**	0.02904042	0.00838113	0.16403018
IN/HL	0.05337124	-0.2708518	-0.0927223	**-0.3483994**	0.07498852
FAL/SVL	-0.0504932	-0.2305715	**0.30752186**	0.27627158	0.30666197
HAL/SVL	0.22149725	0.1237943	**0.36885085**	0.14940119	0.09833018
FIL/SVL	0.25700096	**-0.2741174**	0.10360361	0.10179196	-0.2606619
FIIL/SVL	0.17469264	0.15264738	**0.40255849**	-0.0246909	0.07006198
FIII/SVL	**0.317071**	-0.0339112	0.21749604	0.08187434	-0.0028331
FIV/SVL	0.2646923	0.09418454	**0.33561239**	-0.0586874	-0.1091094
TL/SVL	**0.31181522**	0.09906266	0.13125276	-0.060663	0.11335438
ShL/SVL	**0.34638372**	-0.113053	-0.0622632	-0.0840464	0.07083079
FOL/SVL	**0.32329425**	0.04799012	-0.1594278	-0.1286308	0.19085446
TFOL/SVL	**0.32801279**	-0.1269987	-0.1127892	0.13910136	0.0896213
Eigenvalue	5.92427634	4.00382427	3.61191153	2.06882502	1.36287092
% Total variance	26.928529	18.199201	16.41778	9.40375	6.194868
Cumulative %	26.928529	45.12773	61.54551	70.94926	77.144128
***Jingophrys pachyproctus* species group**					
Variable	PC 1	PC 2	PC 3	PC 4	PC 5
SVL	-0.2464749	**0.3562355**	-0.0072742	-0.0997641	-0.2644385
HL/SVL	-0.0942475	-0.1684897	**0.22489445**	-0.0410466	0.1409598
HW/SVL	-0.0375682	**-0.3003843**	-0.0766205	0.0590046	0.09484804
SL/HL	-0.0174436	0.19797076	-0.154763	0.05103127	**0.22328996**
EL/HL	0.23806728	**-0.2696787**	-0.2648888	-0.0016134	0.2194621
TYD/HL	**0.76565436**	0.11463267	0.02657724	0.28603529	-0.4185095
TYE/HL	-0.3506888	-0.0145303	-0.4785564	**0.58803542**	-0.0326313
EN/HL	0.23661791	**0.58604901**	-0.2750983	0.06951661	0.46275913
NS/HL	-0.0459087	**-0.1635086**	-0.1213988	0.0090381	-0.0900161
IUE/HL	-0.1894768	0.20408607	-0.0094709	0.35286209	**-0.3848212**
UEW/HL	0.09471421	-0.1222223	-0.0866698	**0.14698377**	-0.0236998
IN/HL	0.03061582	0.02044696	-0.0064689	-0.0422048	**0.09905089**
FAL/SVL	-0.0478425	-0.1436778	0.18466436	**0.29936159**	-0.0134803
HAL/SVL	0.07650909	-0.0682011	0.07230691	**0.14837297**	0.12001194
FIL/SVL	-0.0551716	-0.0878561	0.00270874	**0.28483176**	0.27428624
FIIL/SVL	0.01073431	-0.0262997	**0.44673438**	0.29875505	0.25346872
FIII/SVL	0.02753262	0.06550429	0.14110011	**0.20499323**	0.17515887
FIV/SVL	-0.0810067	0.22223977	**0.4967169**	0.2183473	0.04859928
TL/SVL	0.03680465	**-0.136857**	0.04081082	0.08953947	-0.0483734
ShL/SVL	0.10229656	**-0.2942843**	-0.0889359	0.13114533	-0.0861553
FOL/SVL	0.14721738	-0.0748489	0.02531142	0.06891494	**0.20411933**
TFOL/SVL	0.08214289	-0.0607302	-0.0764055	0.01347918	**0.08790075**
Eigenvalue	9.66753	5.861913	3.647847	1.752606	0.00877491
% Total variance	39.788081	24.125529	15.013229	7.213095	3.611439
Cumulative %	39.788081	63.91361	78.92684	86.139934	89.751374

Table S7. Acoustic properties of calls of eight *Jingophrys* species reported from India in the present study, and of three additional species based on data from earlier publications. Values are reported as mean and standard deviation with minimum and maximum inside parantheses, when present.

Species	Temporal properties					Spectral properties		Calling height (m)	Calling site
	Call duration (ms)	Call rise time (ms)	Call fall time (ms)	Pulses/ call	Pulse rate (pulses/s)	Broad frequency peaks	Dominant frequency (kHz)		
J. feii [a]	132.6	–	–	14-20	–	–	4.74-4.91	–	–
J. hrdy **sp. nov.**	180.2 ± 5.4 (171.23–200.8)	57.4 ± 16.1 (24.95–89.43)	122.5 ± 16.1 (86.8–155)	23 (22–27)	127.48 ± 4.75 (120.01–138.5)	Three	3.23 ± 0.04 (3.14–3.36)	1–1.5	Bamboo twig and leaves
J. cf. *pachyproctus* [b]	746 ± 221 (491–889)	–	–	22-62	–	–	3.2 (3.2–3.3)	–	–
J. siangensis **sp. nov.**	59.7 ± 6.6 (49.3–72.0)	21.5 ± 3.8 (8.5–30.7)	38.1 ± 5.3 (31.1–51.6)	12 (10–15)	193.66 ± 9.74 (173.71–216.73)	Two	4.43 ±0.06 (4.31–4.57)	0.1–0.5	Leaf of shrub
J. taniitatti **sp. nov.**	54.5 ± 3.8 (4.9–60.7)	23.3 ± 1.97 (19.38–29.88)	31.2 ± 2.7 (22.8–35.1)	10 (9–11)	184.13 ± 8.92 (155.55–199.9)	Three	3.59 ± 0.08 (3.45–3.75)	0.5–1.5	Fallen tree trunk
J. tanogogo **sp. nov.**	31.1 ± 2.4 (26.3–36.6)	14.7 ± 0.8 (12.5–16.4)	16.5 ± 2.0 (12.8–21.0)	5 (5–6)	140.98 ± 12.15 (120.58–165.35)	Two	4.88 ± 0.1 (4.78–5.08)	0.5–2	Leaf of shrub
J. vegrandis	166.6 ± 6.0 (146.9–180.5)	59.7 ± 18.4 (37.0–165.0)	106.9 ± 17.9 (5.8–129.6)	12 (11–14)	71.03 ± 3.09 (66.65–77.8)	Two	5.46 ± 0.07 (5.21–5.51)	1.5–2	Leaf of shrub
J. wularsoni **sp. nov.**	51.7 ± 2.0 (46.3–56.6)	15.6 ± 0.9 (13.4–17.4)	36.1 ± 1.8 (32.1–40.5)	8 (7–9)	140.8 ± 3.95 (127.18–147.38)	One	2.71 ± 0.04 (2.63–2.8)	1–2	Leaf of shrub
J. cf. *yeae*	109.4 ± 7.5 (99.4–124.1)	39.2 ± 6.4 (27.4–56.1)	70.2 ± 8.6 (53.1–89.8)	12 (11–13)	102.55 ± 9.15 (90.41–116.84)	Two	4.61 ± 0.14 (4.35–4.82)	0.3–1	Leaf of shrub
J. yeae [b]	139 ± 39 (99–212)	–	–	7–12	–	–	4.7 (4.4–5.2)	–	–
J. cf. *zhaoermii*	78.9 ± 4.5 (67.9–87.6)	31.6 ±7.2 (12.8–39.9)	47.2 ± 7.8 (38.3–73.6)	8 (8-10)	99.46 ± 6.57 (90.38–108.81)	Two	4.57 ± 0.13 (4.31–4.78)	0.5–1	Leaf of shrub

[a] Data from Yang et al. (2018).
[b] Data from Shi et al. (2020).

Table S8. Principal Components Analysis (PCA) of six acoustic properties of male calls of members of the *Jingophrys vegrandis* and *Jingophrys pachyproctus* species groups. Values indicate factor loadings and percent variance explained by principal components (PC) 1–5. Bold font denotes variables with the highest loadings.

***Jingophrys vegrandis* species group**					
Variable	PC1	PC2	PC3	PC4	PC5
Call duration	**0.48104657**	0.12167333	-0.0643403	0.02248593	0.43393683
Call rise ime	**0.45787948**	-0.019613	-0.165184	**-0.7333114**	-0.474195
Call fall time	**0.45111821**	0.20929734	-0.0085556	**0.64976896**	-0.5747111
Number of pulses	0.36243334	**0.55497182**	0.41307187	-0.1113609	0.36403297
Pulse rate	-0.3950744	0.43298284	**0.55914127**	-0.1616393	-0.3519394
Dominant frequency	0.26075123	-0.6674861	**0.69658743**	0.03194692	-0.0127976
Eigenvalue	4.21881175	1.25700567	0.31514126	0.19032029	0.01816468
% Total variance	70.313529	20.950095	5.252354	3.172005	0.302745
Cumulative %	70.313529	91.263624	96.515978	99.687983	99.990728
***Jingophrys pachyproctus* species group**					
Variable	PC 1	PC 2	PC 3	PC 4	PC5
Call duration	**-0.4852819**	0.00078259	0.12378556	0.05626742	0.00016879
Call rise ime	-0.4514505	0.20914479	**-0.7793396**	0.21323897	-0.2743705
Call fall time	**-0.4739775**	-0.1056131	0.52955185	0.00611285	**-0.6053948**
Number of pulses	**-0.4720022**	0.17018072	0.21643173	0.30849019	**0.71134098**
Pulse rate	0.32698418	**0.56969791**	0.2078635	**0.67910957**	-0.2284753
Dominant frequency	-0.0795901	**0.76914663**	0.0826548	-0.6284662	0.00331641
Eigenvalue	4.23835661	1.61984762	0.10475842	0.03584063	0.00099085
% Total variance	70.639277	26.99746	1.745974	0.597344	0.016514
Cumulative %	70.639277	97.636737	99.382711	99.980055	99.996569

Table S9. Diagnostic characters for *Jingophrys* species recognised in the present study.

	***J. pachyproctus* group members**				***J. vegrandis* group members**						
Adult morphology											
**Character**	***J. hrdy* sp. nov.**	***J. pachyproctus*[1,2]**	***J. taniitatii* sp. nov.**	***J. wularsoni* sp. nov.**	***J. feii*[3]**	***J. siangensis* sp. nov.**	***J. tanogogo* sp. nov.**	***J. vegrandis***	***J. yeae*[1]**	***J. zhoui*[1]**	***J. zhaoermii*[4]**
Male SVL (mm)	30.0–36.2 (n = 6)	33.0–37.0 (n = 5)	27.4–31.1 (n = 4)	36.2–40.8 (n = 9)	24.3–25.1 (n = 4)	28.6–31.3 (n = 6)	24.9–30.3 (n = 8)	26.8–29.9 (n = 6)	24.9–28.0 (n = 6)	23.0 (n = 1)	34.5–35.4 (n = 2)
Snout shape (dorsal view)	Rounded	Blunt	Bluntly pointed	Rounded	Rounded	Bluntly pointed	Bluntly pointed	Rounded	Rounded	Rounded	Rounded
Dermal fringes on toes	Absent	Absent	Well developed	Absent	Weakly or well developed	Well developed	Well developed	Moderately to well developed	Absent	Absent or weakly developed	Absent
Toe tips (ventral view)	Raised ventrally	–	Raised ventrally	Raised ventrally	–	Raised ventrally	Not raised ventrally	Raised ventrally	Raised ventrally	Not raised ventrally	Not raised ventrally
Dorsal skin texture	Prominent tubercles posteriorly; "> <" shaped ridge present, vertically oriented	Sparsely granular	Prominently tuberculate	Sparsely granular	Granular with small dense tubercles	Prominent tubercles absent; weakly developed "> <" shaped ridge, vertically oriented	With or without small smooth granules	Shagreened to sparsely granular; weakly developed "> <" shaped ridge, vertically oriented	Shagreened to sparsely granular	Smooth	Relatively smooth and lacking tubercles
Lateral skin texture (flank and lateral surfaces)	Prominently tuberculate	Granular with scattered tubercles	Prominently tuberculate, with blackish-brown spots	Prominent large scattered tubercles	Prominently tuberculate	Shagreened with small scattered tubercles	Shagreened with small scattered tubercles	Scattered tubercles	Prominent tubercles surrounded with blackish-brown spots	Smooth	Prominently tuberculate
Dorsal colouration	Greyish brown with a light greenish-yellow tinge	Brown to dark brown	Uniformly greyish-brown	Light reddish brown with light greyish-brown blotches	Light grey-brown	Light brown to greyish brown	Light yellowish brown	Olive or reddish brown	Light greyish brown or greenish brown	Orange brown	Light brown with scattered orange-red tubercles
Ventral colour and markings	Belly greyish brown with prominent blackish-brown blotches	Greyish-brown to blackish brown	Light brown with blackish-brown blotches; blotches with or without intermingled dark grey spots	Greyish brown with blackish-brown blotches	Partial or complete maculation	Discontinuous bands on either side of the belly	Immaculate with two continuous broad dark grey bands extending on either side of the belly	Light silvery brown with scattered brown spots, and two broken dark-brownish bands on either side of the belly	Mottled grey with scattered dots and two broken dark-brownish bands on either side of the belly	Upper abdomen orange-brown, with faint orange dots, and lateral brown stripes on either side of the belly	Grey mottling with purplish-grey hue, scattered with small white blotches

Anterior and posterior surfaces of thighs	Light brown with prominent blackish-brown blotches	Orange	Light brown with greyish spots	Light orangish grey with blackish-brown blotches	Immaculate	Mottled and blotched with blackish brown	Immaculate	Mottled and blotched with dark brown	Larger blackish-brown blotches	Flesh brown with white spots	Light brown with smoky white speckling
Colour of finger and toe tips	Light orangish red	Light red	Pale grey	Light reddish orange	Pale grey with a yellowish tinge	Pale grey	Reddish orange	Orangish	Light orange-red	Light orange	Pale grey
Male calls											
Dominant frequency (kHz)	3.14–3.36	3.2–3.3[1]	3.45–3.75	2.63–2.8	4.74–4.91[3]	4.31–4.57	4.78–5.08	5.21–5.51	4.4–5.2[1]	–	–
Pulses/call	22–27	22–62[1]	9–11	7–9	14–20[3]	10–15	5–6	11–14	7–12[1]	–	–
Pulse rate (pulses/s)	120.0–138.5	–	155.6–199.9	127.2–147.4	–	173.7–216.7	120.6–165.4	66.7–77.8	–	–	–
Call duration (ms)	171.2–200.8	491–889[1]	4.9–60.7	46.3–56.6	132.6[3]	49.3–72.0	26.3–36.6	146.9–180.5	99–212[1]	–	–
Call rise time (ms)	24.95–89.43	–	19.38–29.88	13.4–17.4	–	8.5–30.7	12.5–16.4	37.0–165.0	–	–	–
Broad frequency peaks	Three	–	Three	One	–	Two	Two	Two	–	–	–

[1] Data from Shi et al. (2020).
[2] Data from Huang and Fei (1981).
[3] Data from Yang et al. (2018).
[4] Data from Yuan et al. (2026).
