# Supplementary material for: Two-fold hidden diversity of diminutive horned frogs (Megophryinae: Jingophrys) highlights overlooked in situ diversification in the Himalayas
Source: R Soc Open Sci. 2026 Nov 11;13(11):261030. doi: 10.1098/rsos.261030 (PMC13752532; doi:10.1098/rsos.261030)
Supplement: Supplementary file 1 [file rsos.261030.zip › rsos.261030/rsos261030_si_002.pdf]

Electronic Supplementary Material: Figures S1–S8

Two-fold hidden diversity of diminutive horned frogs (Megophryinae: *Jingophrys*) highlights overlooked *in situ* diversification in the Himalayas

Sonali Garg[1,3], S. D. Biju[1,2,3], Tage Tajo[3], Radhakrishna Upadhyaya K.[3], and James Hanken[1]
[1] Department of Organismic and Evolutionary Biology and Museum of Comparative Zoology and
[2] Radcliffe Institute for Advanced Study, Harvard University, Cambridge, Massachusetts, USA
[3] Systematics Lab, Department of Environmental Studies, University of Delhi, Delhi, India
Author for correspondence: Sonali Garg, e-mail: sonaligarg@fas.harvard.edu

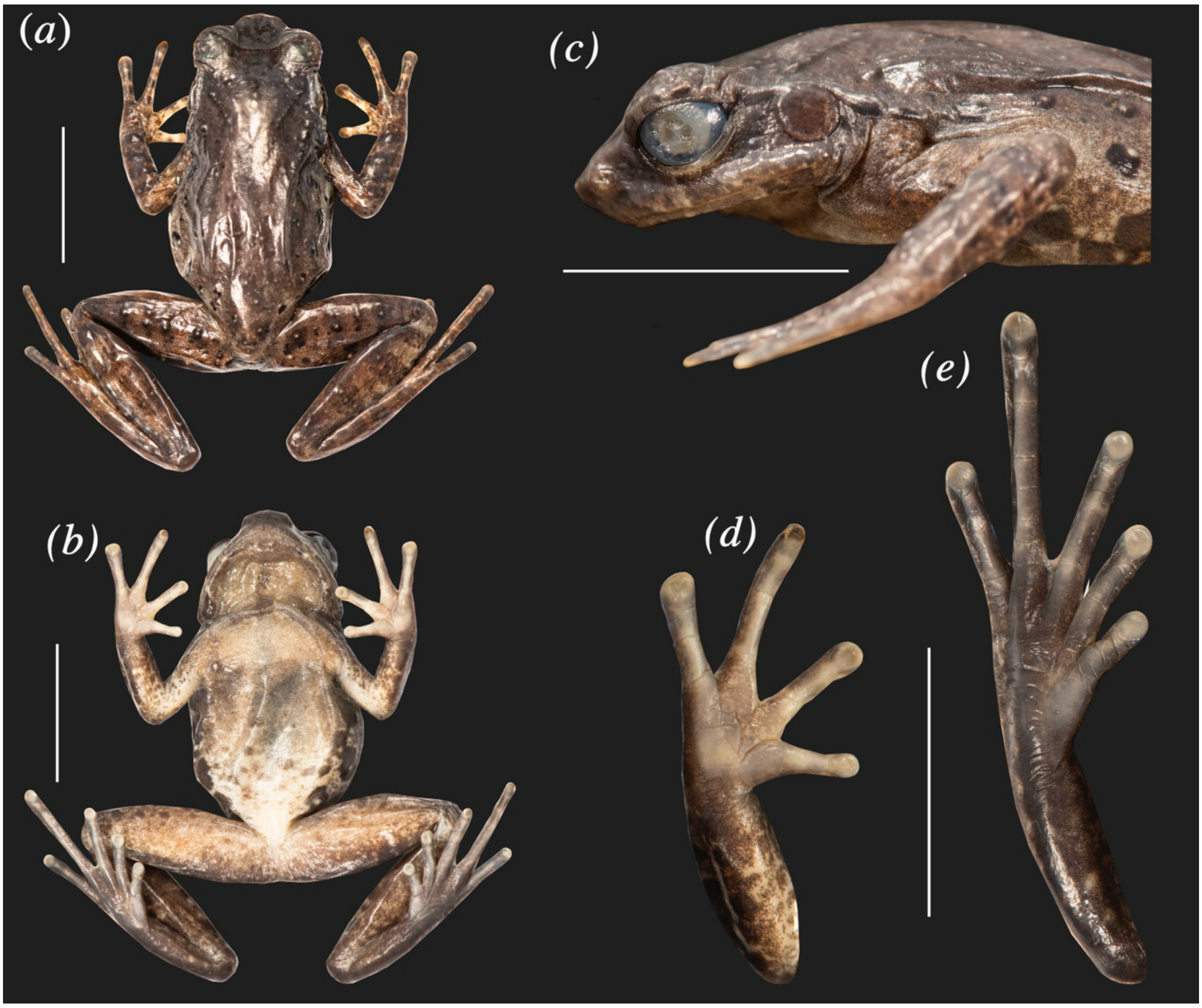

Figure S1. Holotype (ZSI A 16611, adult male) of *Jingophrys siangensis* sp. nov., in preservative. (*a*) Dorsal view, (*b*) ventral view, (*c*) lateral view of head, (*d*) ventral view of right hand, and (*e*) ventral view of right foot. Scale bars = 10 mm.

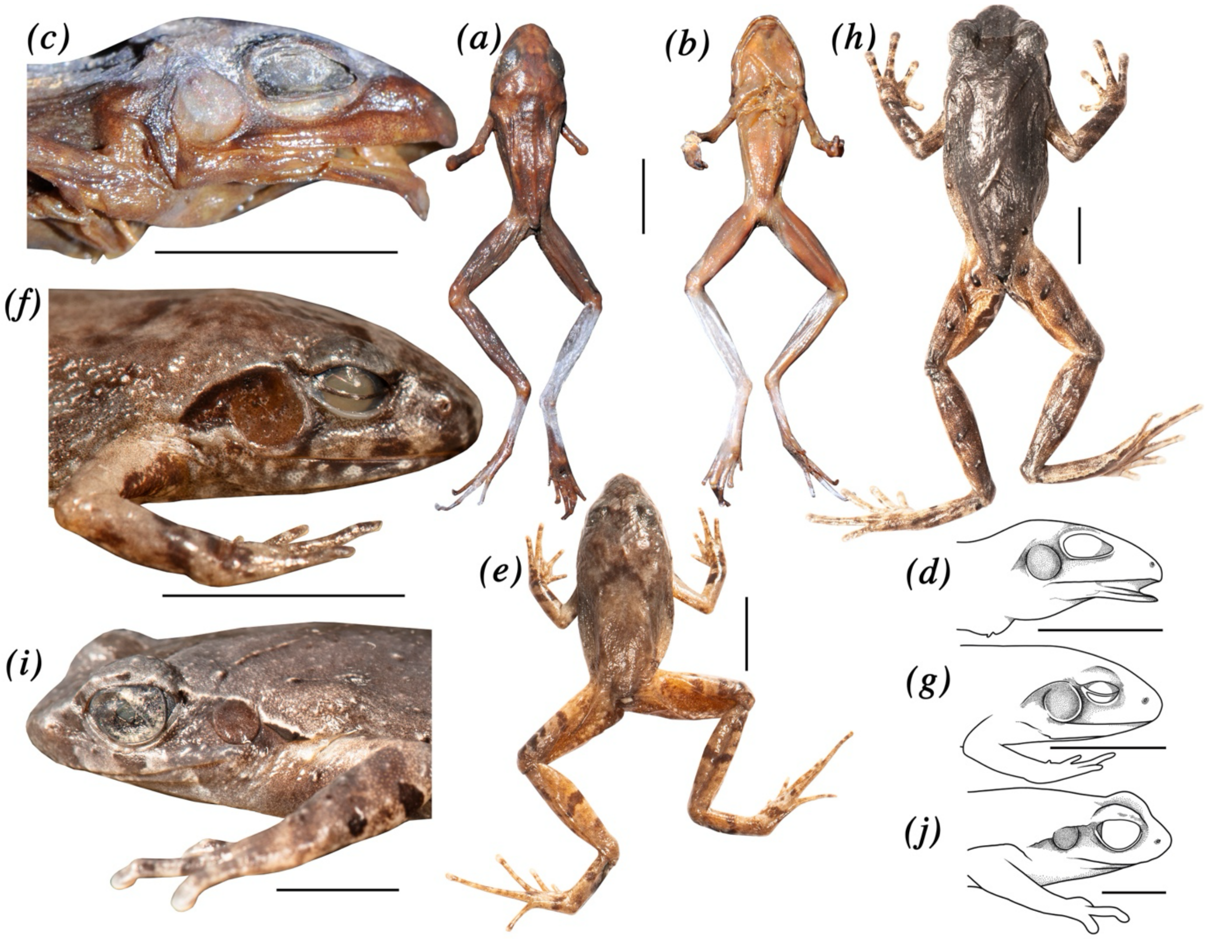

Figure S2. Morphological comparison of *Jingophrys siangensis* sp. nov. with *Liurana kempii*. (*a–d*) Holotype (ZSIC 17013) of *Megalophrys kempii* Annandale, 1912, in preservative: (*a*) dorsal view, (*b*) ventral view, (*c*) lateral view of head, and (*d*) enlarged view of tympanum and eye; (*e–g*) *Liurana* sp. (SDBDU 2022.6272) from Yembung, in the vicinity of the type locality of *Megalophrys kempii* Annandale, 1912 (= *Liurana kempii*): (*e*) dorsal view, (*f*) lateral view of head, and (*g*) enlarged view of tympanum and eye; (*h–j*) *Jingophrys siangensis* sp. nov. (paratype, ZSI A 16614) from Pangin, in the vicinity of the type locality of *Liurana kempii*: (*h*) dorsal view, (*i*) lateral view of head, and (*j*) enlarged view of tympanum and eye. Scale bars = 5 mm.

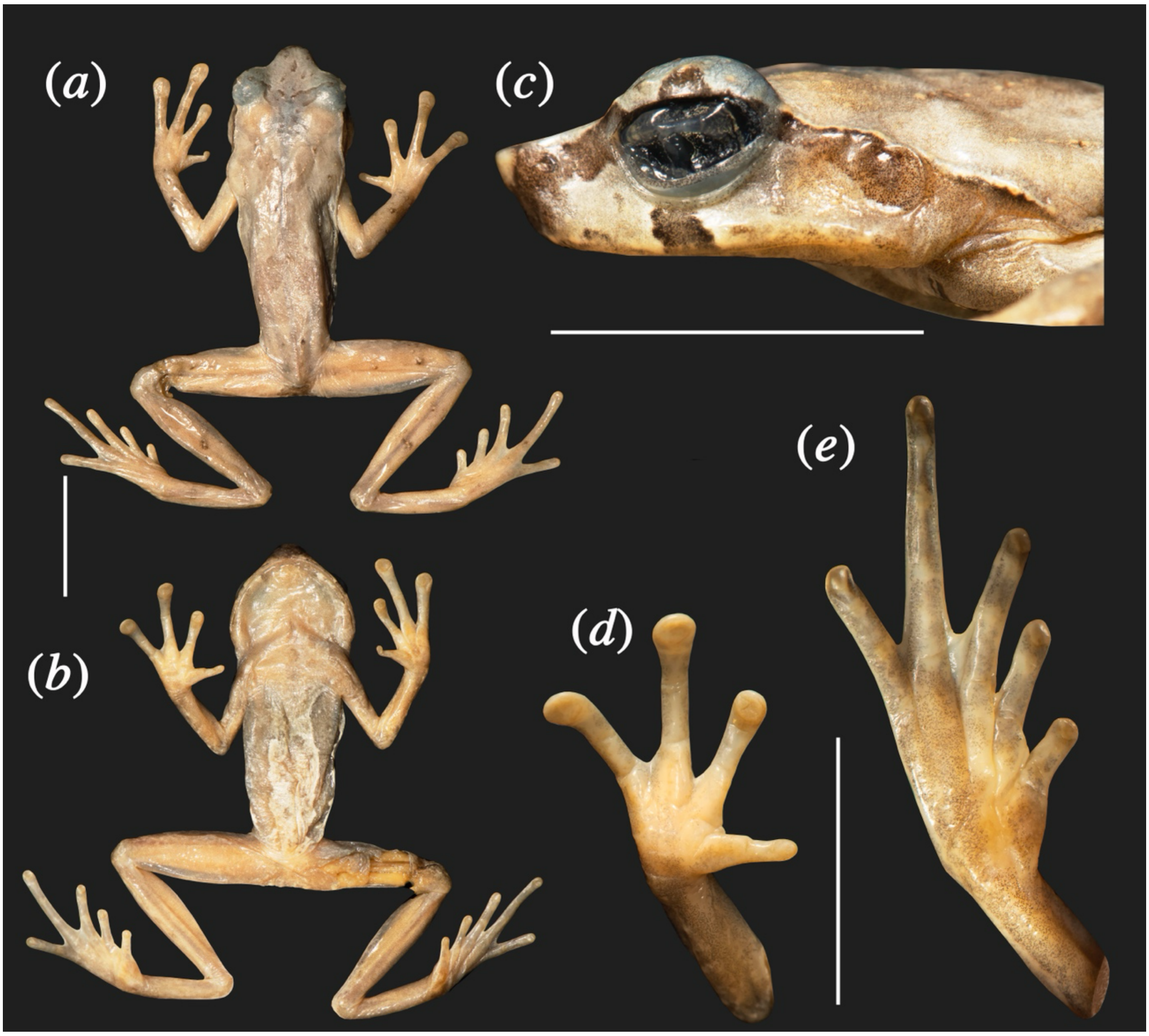

Figure S3. Holotype (ZSI A 16616, adult male) of *Jingophrys tanogogo* sp. nov., in preservative. (*a*) Dorsal view, (*b*) ventral view, (*c*) lateral view of head, (*d*) ventral view of right hand, and (*e*) ventral view of right foot. Scale bars = 10 mm.

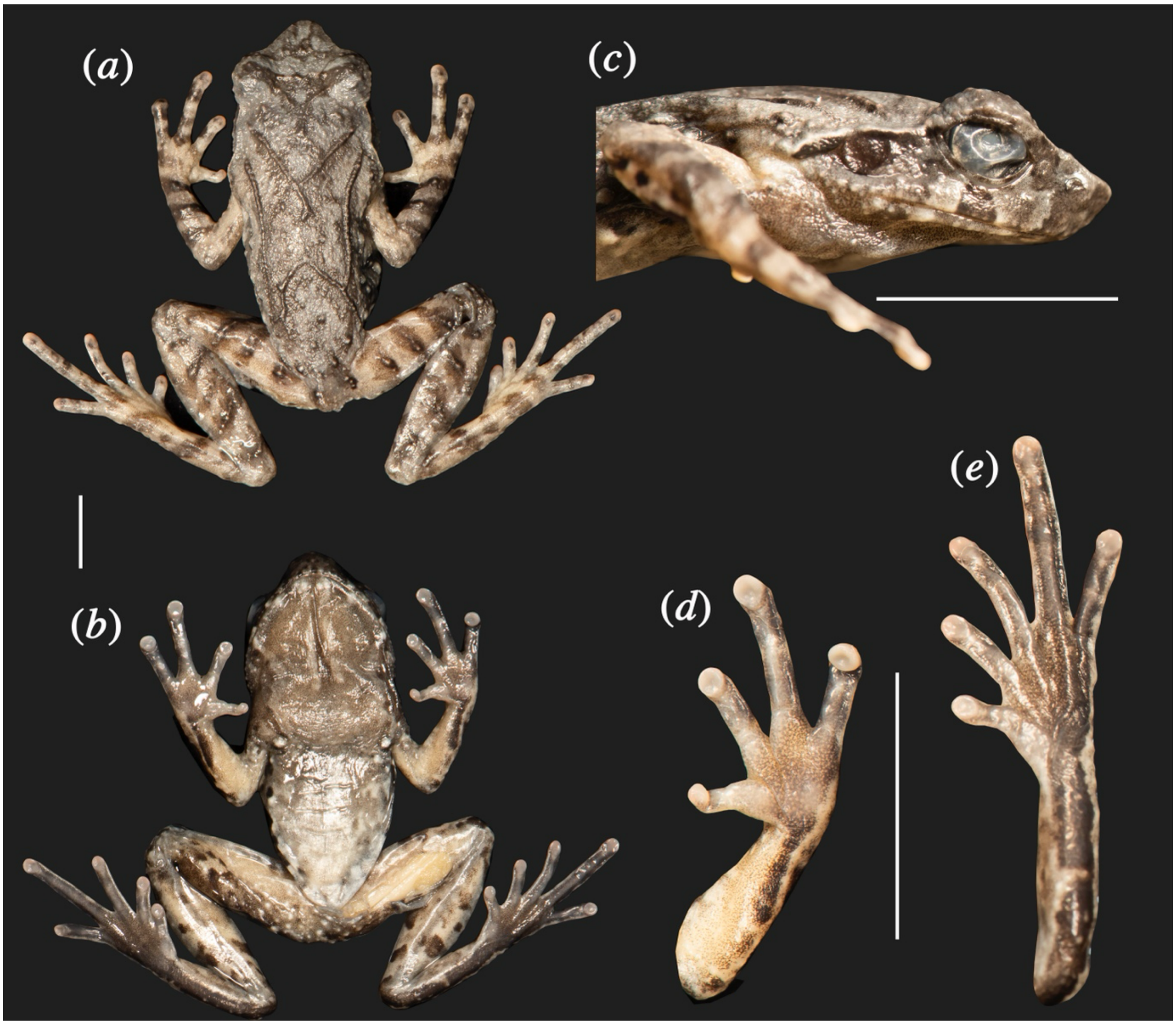

Figure S4. Adult male (SDBDU 2023.6379a) of *Jingophrys* cf. *yeae*, in preservative. (*a*) Dorsal view, (*b*) ventral view, (*c*) lateral view of head, (*d*) ventral view of left hand, and (*e*) ventral view of left foot. Scale bars = 10 mm.

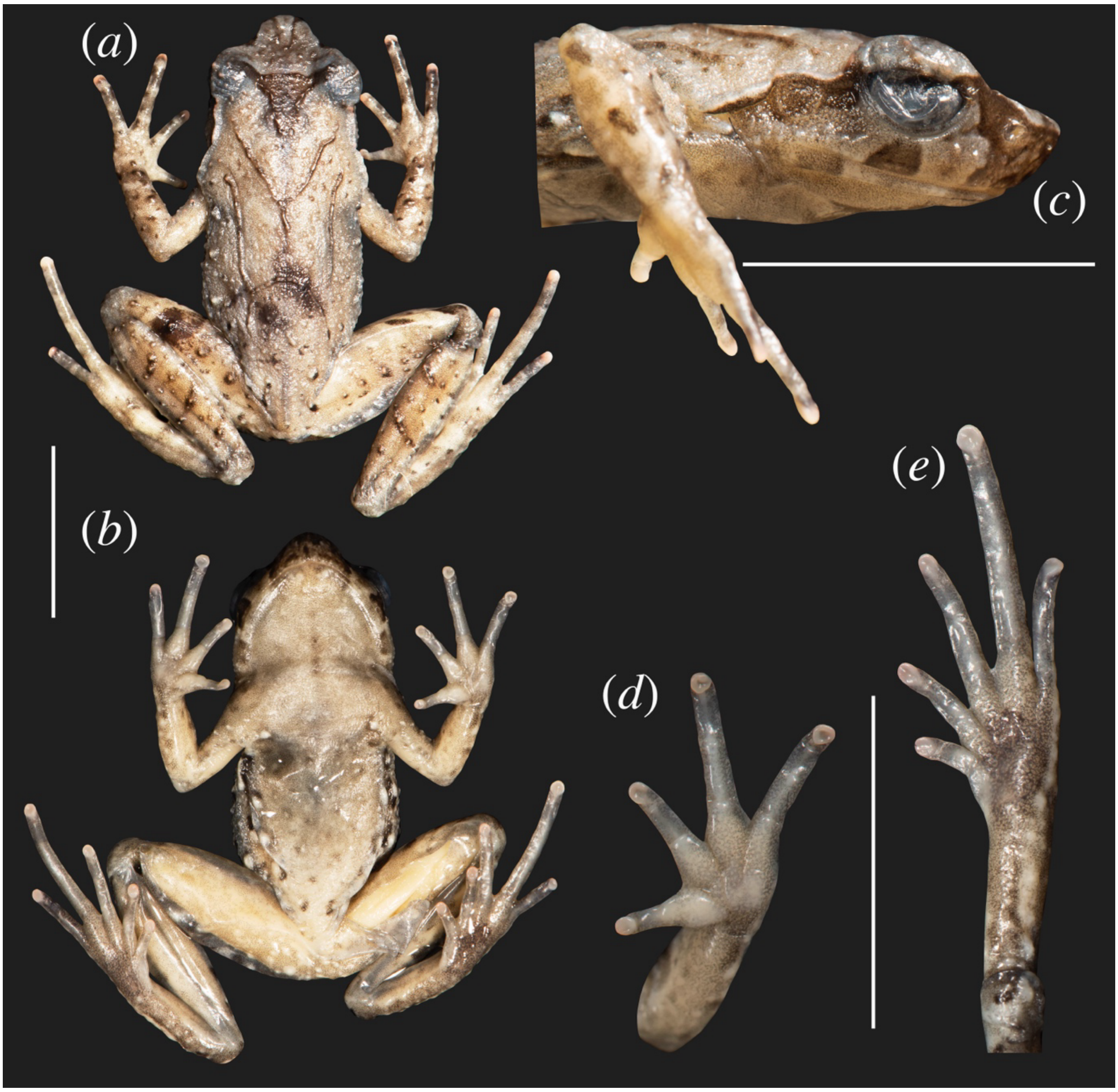

Figure S5. Adult male (SDBDU 2023.6376) of *Jingophrys* cf. *zhaoermii*, in preservative. (*a*) Dorsal view, (*b*) ventral view, (*c*) lateral view of head, (*d*) ventral view of left hand, and (*e*) ventral view of left foot. Scale bars = 10 mm.

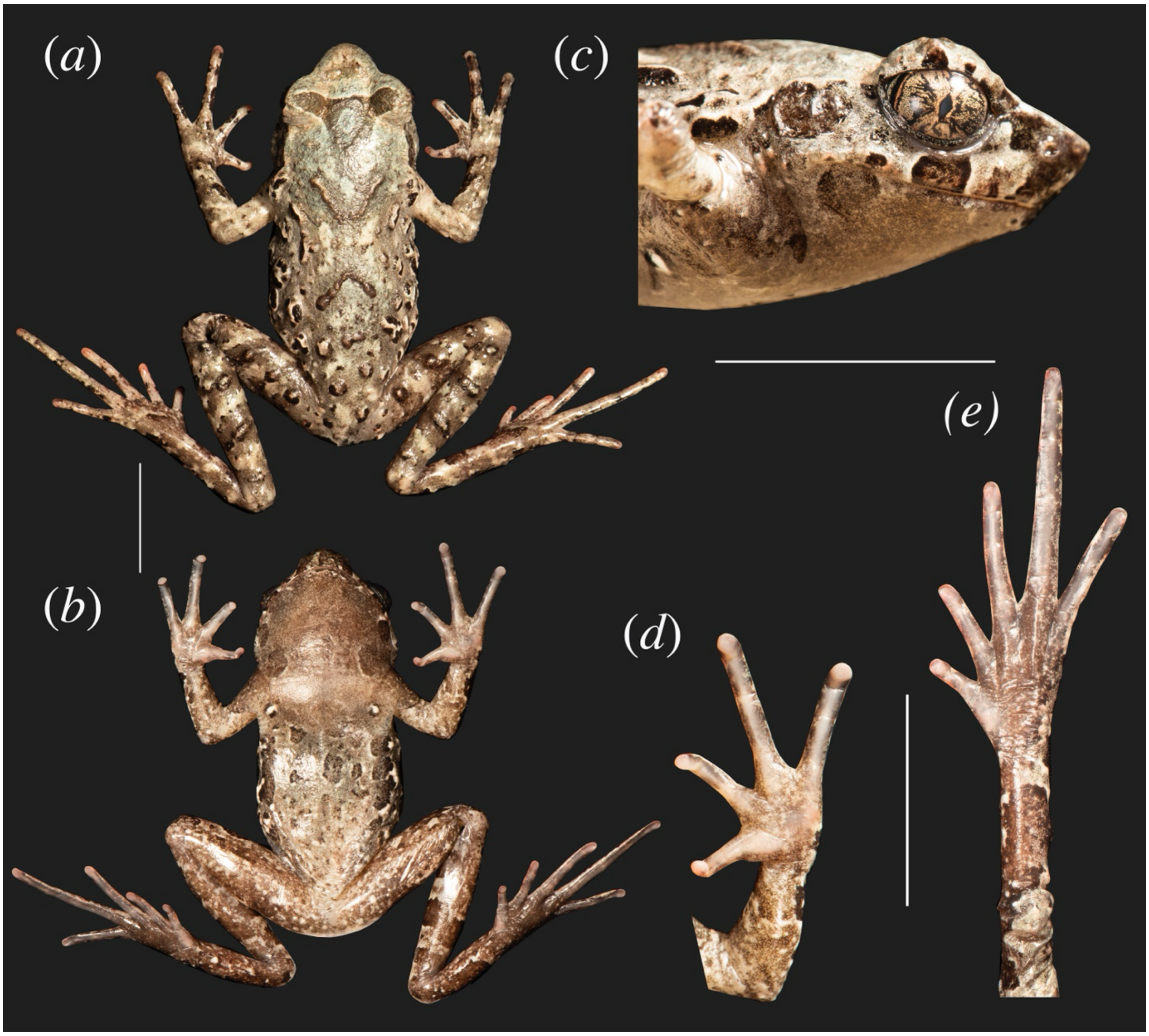

Figure S6. Holotype (ZSI A 16624, adult male) of *Jingophrys hrdy* sp. nov., in preservative. (*a*) Dorsal view, (*b*) ventral view, (*c*) lateral view of head, (*d*) ventral view of left hand, and (*e*) ventral view of left foot. Scale bars = 10 mm.

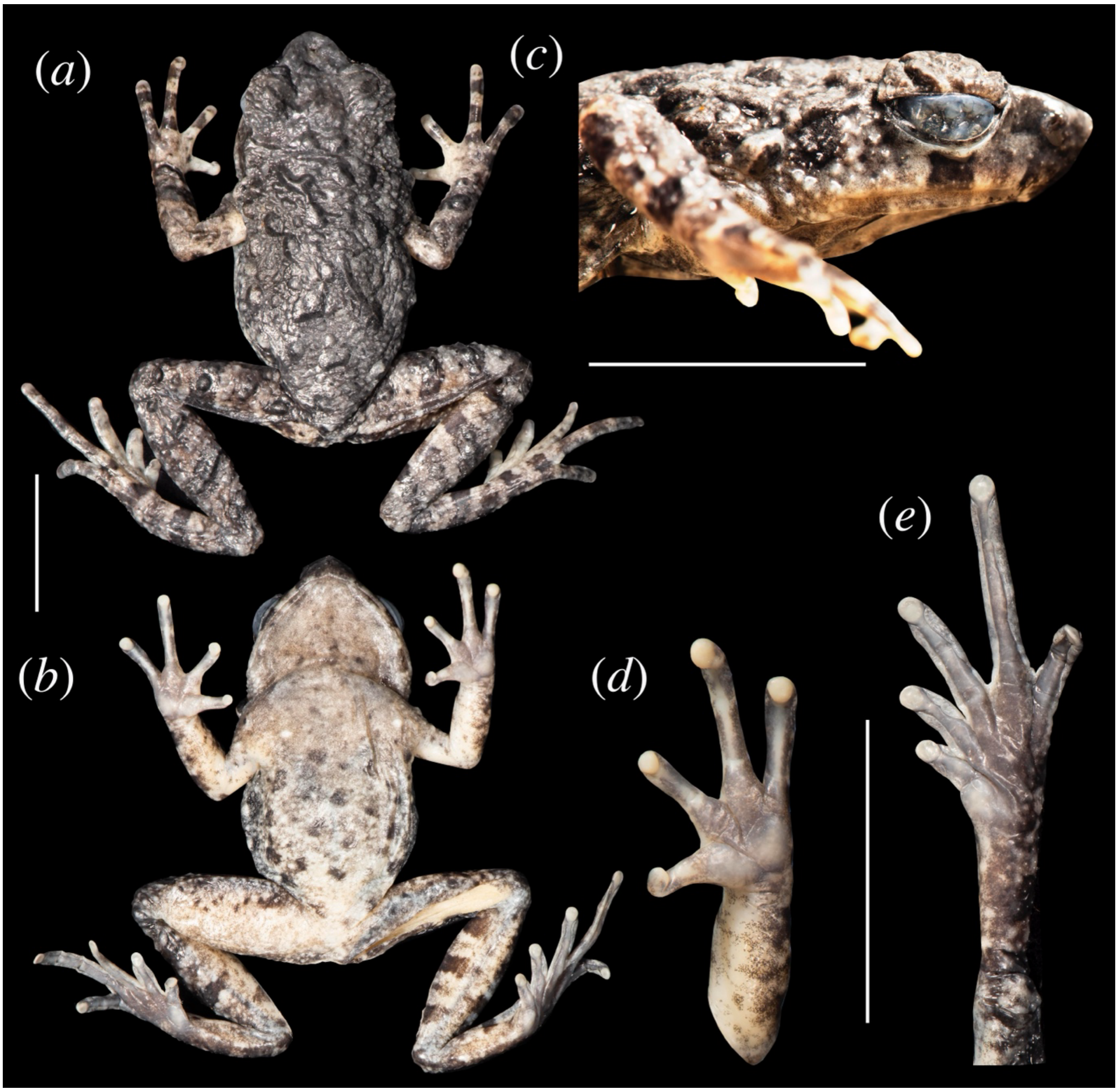

Figure S7. Holotype (ZSI A 16628, adult male) of *Jingophrys taniitatii* sp. nov., in preservative. (*a*) Dorsal view, (*b*) ventral view, (*c*) lateral view of head, (*d*) ventral view of left hand, (*e*) ventral view of left foot. Scale bars = 10 mm.

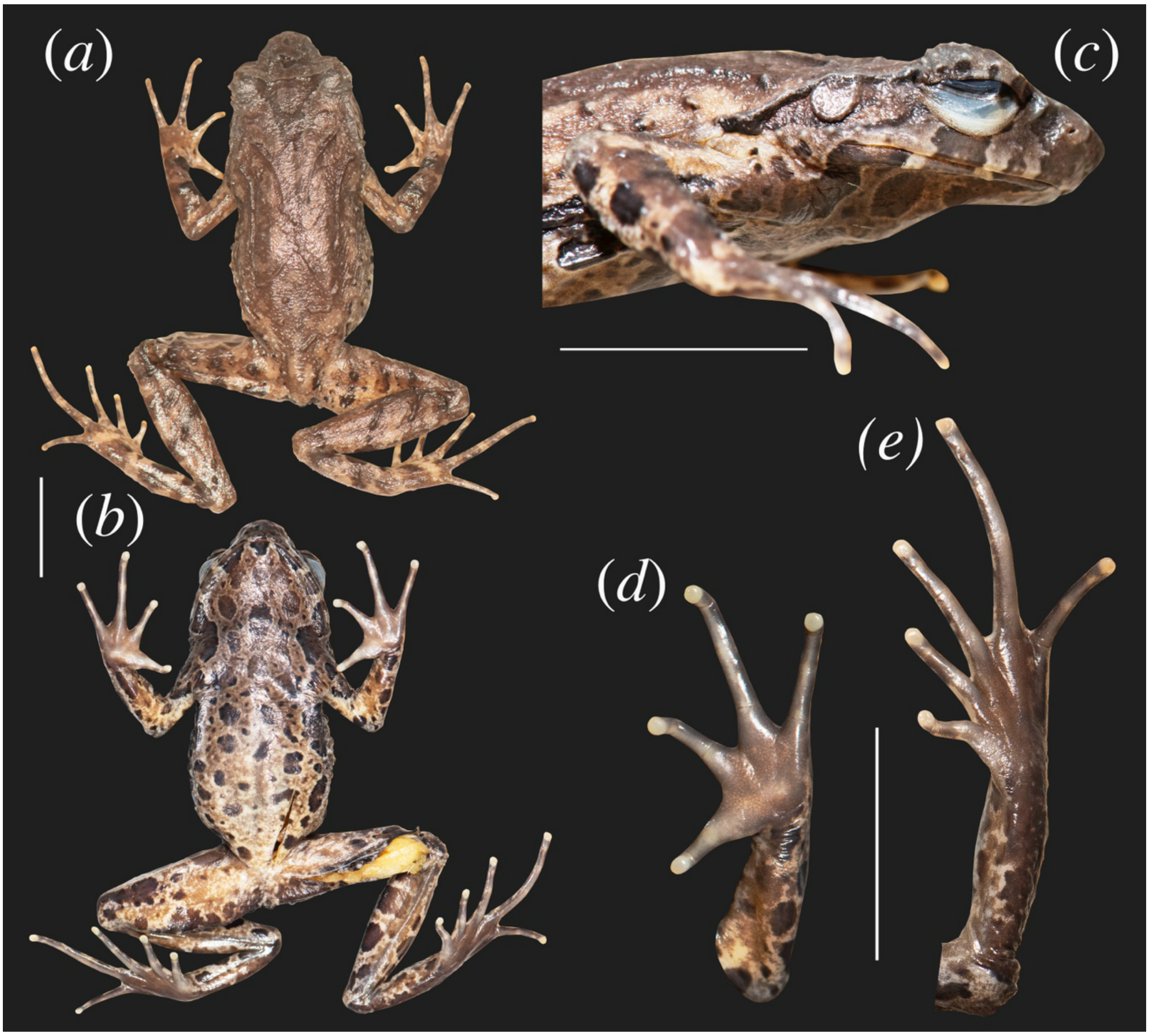

Figure S8. Holotype (ZSI A 16631, adult male) of *Jingophrys wularsoni* sp. nov., in preservative. (*a*) Dorsal view, (*b*) ventral view, (*c*) lateral view of head, (*d*) ventral view of left hand, and (*e*) ventral view of left foot. Scale bars = 10 mm.
